# Reference values for body composition in healthy urban Mexican children and adolescents

**DOI:** 10.1038/s41430-023-01352-1

**Published:** 2023-10-16

**Authors:** Lopez-Gonzalez Desiree, Jonathan C Wells, Partida-Gaytan Armando, Cortina-Borja Mario, Clark Patricia

**Affiliations:** 1 Clinical Epidemiology Research Unit, Hospital Infantil de México Federico Gómez, 2. Universidad Nacional Autonoma de México, Mexico City, Mexico; 2 Childhood Nutrition Research Centre. Population, Policy and Practice Research and Teaching Department, University College London Great Ormond Street Institute of Child Health, London, UK; 3 Associate Clinical Researcher, Clinical Research Direction, Hospital Infantil de México Federico Gómez, Mexico City, Mexico; 4 Population, Policy and Practice Research and Teaching Department, University College London Great Ormond Street Institute of Child Health, London, UK

**Keywords:** Paediatrics, Nutrition

## Abstract

**Introduction:**

Given the increasing incidence of chronic degenerative diseases related to changes in tissues, the availability of diagnostic tools with greater accuracy in the estimation of body composition (BC) has become necessary. Interpreting the BC values of individuals requires reference data obtained from a healthy population with the same ethnicity, to identify individuals at risk for the development of negative health outcomes.

**Objective:**

Generate reference values (RV) of body composition (BC) for Mexican children and adolescents.

**Methods:**

This was an urban-population-based cross-sectional study of healthy Mexican children and adolescents. BC estimations by anthropometry, multifrequency bioimpedance analysis (MF-BIA) and dual-energy X-ray absorptiometry (DXA) where used to create sex- and age-specific RV by means of generalized additive models for location, scale and shape (GAMLSS).

**Results:**

We assessed 2104 subjects, and after confirming a clinically and metabolically healthy status, we measured 1659 subjects aged 5–20 years, [806 females (49%) and 853 males (51%)] by anthropometry, MF-BIA and DXA to create sex- and age- smoothed reference centiles, lambda (L), mu (M), and sigma (S) values. We also built sex- and age-smoothed graphic curves for each variable of interest.

**Conclusions:**

We present valid RV and curves for BC variables estimated by anthropometry, MF-BIA and DXA from clinically and metabolically healthy urban Mexican children and adolescents. These RV are different from those reported for other populations, and therefore, should be used for clinical and research purposes involving urban Mexican children and adolescents.

## Introduction

Body mass index (BMI) is a simple and informative tool fundamental for the classification of nutritional status as malnutrition, healthy weight, overweight (OW) or obesity (OB) [1]. Although BMI shows good correlation with adiposity, it does not consider other tissues, and subjects of the same age, sex and BMI can vary twofold in their amount of body fat, due to differences in lean mass (LM) [2]. BMI does neither provide information on the distribution of such tissues among body regions, consequently, the clinical usefulness of BMI is limited [3].

The evaluation of the distribution of the tissues that conform the human body [i.e., body composition (BC)], is increasingly relevant in the assessment of health and disease. For clinical purposes, BC evaluates the proportions of fat mass (FM), total body water (TBW) [4], bone mineral content (BMC), and lean mass (LM) [5]. Different methods and devices can estimate BC through models that take into account the direct measurement and/or calculation of one to four or more components, and the context usually defines the ideal method to be used [5]. In clinical practice, a balance between quantity, accuracy and practicality is pursued. For research purposes, reference standards (i.e. the 4-component model) are usually preferred.

Evaluation of BC in the pediatric population is of great relevance due to the increasing prevalence of chronic degenerative diseases related to excess adipose tissue, as well as other clinical conditions where the distribution of human body compartments is altered [6, 7].

In Mexico, 30% of the children and adolescents are affected by OW/OB [8], without knowledge of their metabolic status, and reaches 70% for the adult population. Given the magnitude of the impact of OW/OB and their comorbidities in our population, it is likely that we should consider different approaches to improve sensitivity to the problem.

Characterizing BC and generating reference values (RV) for a particular pediatric population is useful for individual clinical evaluations, and informative from an anthropological perspective. Such RV should ideally characterize the healthy subjects of a particular population, therefore, they must be generated from a representative sample that meets strict selection criteria that define such healthy state [9]. Previous publications have stated relevant BC differences between different populations [e.g. Hispanics have shown higher values of FM compared to those of white and black groups in United States of America (USA)], hence the need for population-specific RV [10, 11].

The objective of this study was to create valid RV of BC for Mexican urban children and adolescents for anthropometry, multifrequency bioimpedance analysis (MF-BIA) and dual-energy X-ray absorptiometry (DXA).

## Methods

This was an urban population-based, cross-sectional study of clinically and metabolically healthy Mexican children and adolescents.

### Study subjects and recruitment

Considering the variability of BC variables for both sexes and across the age-span of interest, we estimated the sample size using the distribution of FM-data reported for the UK pediatric population [12] and stratified for age and sex, obtaining a sample size of 62 subjects per year of age and sex.

We performed a random, multistage and stratified sampling from the list of Mexico City’s primary and secondary schools registered at the Mexican Ministry of Public Education (*n* = 7511) [13]. Stratification factors included public/private sector, education level and administrative delegation. Fifteen schools were invited, and 13 agreed to participate. We also invited 15 preparatory schools of the National Autonomous University of Mexico of which 3 participated.

Written invitation was sent to parents between March 2015 and November 2019. Family members and friends who met inclusion criteria were also invited. During the study, 4 additional schools (2 elementary, 1 secondary, 1 preparatory) and one soccer- club were included.

Inclusion criteria consisted in individuals from 5 to 20 years of age confirmed as clinically and metabolically healthy by clinical history, examination and laboratory tests, Mexican ethnicity (i.e., the subject, parents and all four grandparents must have been born in Mexico, with Spanish as their maternal language), birth weight >2.5 kg, no history of chronic diseases, no intake of drugs known to modify bone mass (e.g., hormonal therapy, corticosteroids, antiepileptics, methotrexate, etc.), no clinical evidence of early puberty (defined as breast development in girls <8 years or pubic hair growth in boys <9 years), no history of ≥2 fractures, and no history of pregnancy or current pregnancy.

Informed consent for all parents or guardians, and informed assent for subjects 7 years and older where obtained. The study was executed in accordance with the Declaration of Helsinki, approved by our institution’s research, ethics and biosafety committees (HIM 2015-055), and all measurements were performed in our center.

#### Measurements

Each subject was clinically assessed to confirm their health status, register their sexual maturation based on Tanner’s criteria and collect relevant clinical and demographic data [14, 15].

### Anthropometric measurements

Anthropometry was performed with subjects wearing lightweight clothing, measuring weight and height (SECA® 284 scale stadiometer, Hamburg, Germany), BMI was calculated and corresponding *z*-scores were computed based on growth charts from the WHO [16, 17]. Waist and hip circumferences were measured according to the World Health Organization (WHO) standards. Mid upper arm circumferences (MUAC) (cm) of both arms were measured midway between the tip of the acromion and olecranon to the nearest cm with the arms hanging. Thigh circumferences were measured at the midpoint from the inguinal crease to the proximal pole of the patella, and calf circumferences were measured at the point of greatest circumference. All circumferences were measured with a SECA® 201 measuring tape. Skinfold (SF) thicknesses were measured in accordance with the Lohman technique following The International Society for the Advancement of Kinanthropometry (ISAK) recommendations measured at the triceps, thigh, and calf, twice at each site and on both sides of the body, using a caliper with a scale of 0–80 mm and precision of ±0.2 mm (Harpenden caliper, British Indicators Ltd, St Albans, UK) [18].

We used Slaughter equation to estimate BC values of FM percentage (FM%) and Lee’s-Poortmans equation to estimate skeletal muscle mass (SMM) [19, 20].

$$\begin{array}{l}{\rm{Males}}\,{\rm{Percentage}}\,{\rm{of}}\,{\rm{fat}}( \% )=0.735({\rm{triceps}}+{\rm{calf}})+1.0\\ {\rm{Females}}\,{\rm{Percentage}}\,{\rm{of}}\,{\rm{fat}}( \% )=0.610({\rm{triceps}}+{\rm{calf}})+5.1\end{array}$$

Total fat-mass = fraction of fat × weight (kg).

Total fat free mass = weight – total fat mass

With 8 h of fasting, serum glucose, insulin, total cholesterol, triglycerides, high-density lipoprotein (HDL) cholesterol, and low-density lipoprotein (LDL) cholesterol were assessed for each subject.

Subjects with BMI *z-*scores of +3 or −3; values of glucose ≥100 mg/dL, total cholesterol ≥200 mg/dL; C-HDL < 40 mg/dL or <45 mg/dL for post-pubertal girls; triglycerides ≥100 mg/dL for children under 10 years or ≥130 mg/dL for children 10–19 years; blood pressure ≥90th percentile by age, height and sex based on the Expert Panel on Integrated Guidelines for Cardiovascular Health and Risk Reduction in Children and Adolescents criteria [21]; and insulin resistance defined by HOMA-IR > 3.5 were not considered for the generation of reference values; as these abnormalities were considered as exclusion factors to ensure the healthy status of the sample.

### MF-BIA measurements

Three different MF-BIA devices were used: two for standing measurements using footplates and handgrips MF-BIA Handrail (SECA® mBCA 514, Hamburg, Germany) and MF-BIA paediatric prototype (SECA® paediatric prototype) with height-adjustable electrodes, and one for MF-BIA supine measurements (SECA® 525, Hamburg, Germany) with eight electrodes positioned two at each hand and foot. Measurements were performed in accordance with the manufacturers’ instructions.

Standing-position: For MF-BIA Handrail, each side of the handrail carries six electrodes, out of which two on each side are chosen depending on the participant’s height, arms should be held straight at 30 degrees from the body the minimum height to hold this position is 140 cm. In the paediatric prototype, two height-adjustable hand electrodes are available and subjects must hold them with the arms at an angle of 30 degrees from the body, this device does not require a specific height to generate valid measurements.

Supine-position BIA is designed for measurements in the supine position and can be operated using either 4 adhesive electrodes on the right side of the body (4e) or 8 electrodes (8e) on both sides of the body while the subject is lying supine on a non-conductive surface. Subjects should lye supine for 10 min before being measured.

The MF-BIA equations have been previously validated for Mexican children and adolescents [22].

### DXA measurements

Whole-body scans were performed using Lunar-iDXA instrumentation (GE Healthcare, Madison, WI, USA) following manufacturer’s instructions for subject positioning and recommendations from The International Society for Clinical Densitometry (ISCD) [23–25]. Daily calibration was performed with a phantom. We used ENCORE software version 15 to obtain BC values for the total body and surrogate regions: arms, trunk and legs.

Technical precision error was estimated based on repeated scans of an independent sample of 30 voluntary children and adolescents resulting in 0.005 g/cm^2^ root mean square standard deviation (RMS SD), with a Least Significant Change (LSC) of 0.014 g/cm^2^ at 95% confidence level which was acceptable according to ISCD criteria.

### Statistical analyses and establishment of RV

Descriptive statistics were used to characterize the sample. Subjects with incomplete data, and outliers for height, weight and BMI were eliminated.

FM, FFM and LM indexes (FMI, FFMI and LMI respectively) were calculated using the ratio of each component in kg and the height in m squared aiming to adjust for body size [26, 27].

For the BC estimations with MF-BIA we took the mean value obtained with the total measurements (from two or three devices) for each subject.

Generalized additive models for location, scale and shape (GAMLSS) were fitted by maximum likelihood in R language version 3.6.3 within the R-Studio platform, version 1.2.5033 [28]. The RV were defined by sex- and age-related centiles, for the age range 5 to 18 years. The 1st, 3rd, 5th, 15th, 25th, 50th, 75th, 85th, 95th, 97th and 99th centiles for each 0.5-year age group were estimated. Data from subjects in the age groups of 4 and 19–20 years were considered in the curve-fitting procedure because of their influence on the smoothing process, but specific RV for these age groups are not presented. Models were fitted based on the Box-Cox-Cole-Green (BCCG) distribution, fitting smooth curves defining parameters for location (median, *mu*, M), scale (coefficient of variation, *sigma*, S), and shape (skewness, *lambda*, L), as goodness-of-fit was not improved by fitting four-parameter distributions. Penalized p-splines [29] specified the smooth terms, with their degrees chosen by a generalized cross-validation criterion.

In the interest of comparing the RV proposed in this study with those of other populations, we performed graphical comparisons of BC, specifically FMI, LMI; estimated by DXA, two variables that were the most consistently reported among the different studies. These analyses included data from 1999–2004 USA NHANES (measured by DXA Hologic, but transformed to Lunar equivalents through a validated method) [30], from India (measured with DXA-Lunar during 2006–2010) [31], from UK (measured with DXA-Lunar during 2001–2011 [12] that although not previously published as FMI, LMI, these were provided by the author for this study).

All graphical representations were generated in GraphPad Prism version 9.3.1 (350) for Mac OS X, GraphPad Software, San Diego, California USA, www.graphpad.com.

## Results

A total of 2721 subjects called our center expressing interest to participate. Those identified as eligible were invited to our research center where all study assessments took place. We evaluated 2104 subjects (1073 males, 51%, and 1031 females, 49%). Data of weight, height and BMI of this sample paired by age and sex [32] and compared to that of the National Health and Nutrition Survey ENSANUT 2018 [8] showed differences that reached statistical significance only for a minority of age-specific subgroups providing supportive evidence of the representativeness of our sample (supplementary fig. 1).

For the generation of RV, 445 subjects (21%) had one or more exclusion criteria (i.e. metabolic alteration, see details in participants’ flowchart in Online supplementary material Fig. 2). A total of 1659 subjects (79% of the total sample), i.e., 806 females (49%) and 853 males (51%), composed the sample for the generation of BC RV. All 1659 subjects were measured by SF, DXA and supine MF-BIA. For the standing MF-BIA 1020 subjects were measured by the MF-BIA Handrail, and 1392 with the paediatric prototype. Intraclass correlation coefficient between the three different MF-BIA devices was 0.998 CI 95% (0.998 to 0.999, *P* < 0.001).

Table 1 describes the demographics, clinical and biochemical characteristics of the subjects by age group.

**Table 1:** Clinical and demographic characteristics of the subjects.

*n* = 1659	Young children *n* = 135 (8%)	Children *n* = 702 (42%)	Adolescents *n* = 611 (37%)	Young adults *n* = 211 (13%)
*Sex*				
Female *n* (%)	71 (53%)	304 (43%)	310 (51%)	121 (57%)
Male *n* (%)	64 (47%)	398 (57%)	301 (49%)	90 (43%)
Age (years)	5.4 ± 0.4	8.9 ± 1.7	15.1 ± 1.7	19.2 ± 0.9
Weight (kg)	19.5 ± 3.8	30.2 ± 9.4	54.5 ± 11.1	60.9 ± 12.2
Height (cm)	110.2 ± 5.9	130.9 ± 11.7	160.2 ± 8.9	164.4 ± 9
Height Z-score^WHO^	−0.23 ± 1.05	−0.30 ± 0.98	−0.53 ± 0.96	−0.54 ± 0.86
BMI (kg/m^2^)	16 ± 2.2	17.3 ± 3	21.1 ± 3.4	22.49 ± 3.7
BMI Z−score^WHO^	0.22 ± 1.32	0.11 ± 1.12	0.14 ± 1.02	-0.05 ± 1.05
Waist circumference (cm)	53.4 ± 5.3	60.5 ± 8.7	72.6 ± 8.1	76.3 ± 8.1
Systolic blood pressure (mmHg)	94 ± 7	97 ± 8	105 ± 9	110 ± 11
Diastolic blood pressure (mmHg)	59 ± 6	61 ± 6	65 ± 6	68 ± 6
Waist-height index	0.49 ± 0.04	0.46 ± 0.05	0.45 ± 0.05	0.47 ± 0.06
*Tanner´s puberal stage*				
I	135 (100%)	535 (76%)	12 (2%)	0
II	0	120 (17%)	36 (6%)	0
III	0	41 (6%)	140 (23%)	0
IV	0	6 (1%)	278 (46%)	28 (13%)
V	0	0	145 (24%)	183 (87%)
*BMI category*				
Healthy weight	94 (70%)	495 (71%)	455 (75%)	161 (76%)
Overweight	15 (11%)	99 (14%)	101 (17%)	28 (13%)
Obesity	14 (10%)	65 (9%)	29 (5%)	6 (3%)
Low weight	11 (9%)	43 (6%)	25 (4%)	16 (8%)

The values are expressed as mean ± standard deviation, or number and percentage. *BMI* body mass index, *WHO* world health organization. The Z-scores for height and BMI were calculated according the WHO standards.

BMI category according to WHO: Healthy weight: Z-score >-2 to <1, overweight: Z-score ≥1 to <2, obesity ≥2 to <3, low weight ≤ −2 to > −3.

Young children: 4.0–5.9 years, children: 6–11.9 years, adolescents 12–17.9 years and young adults 18–20.9 years.

### Reference values

**Fig. 1:**
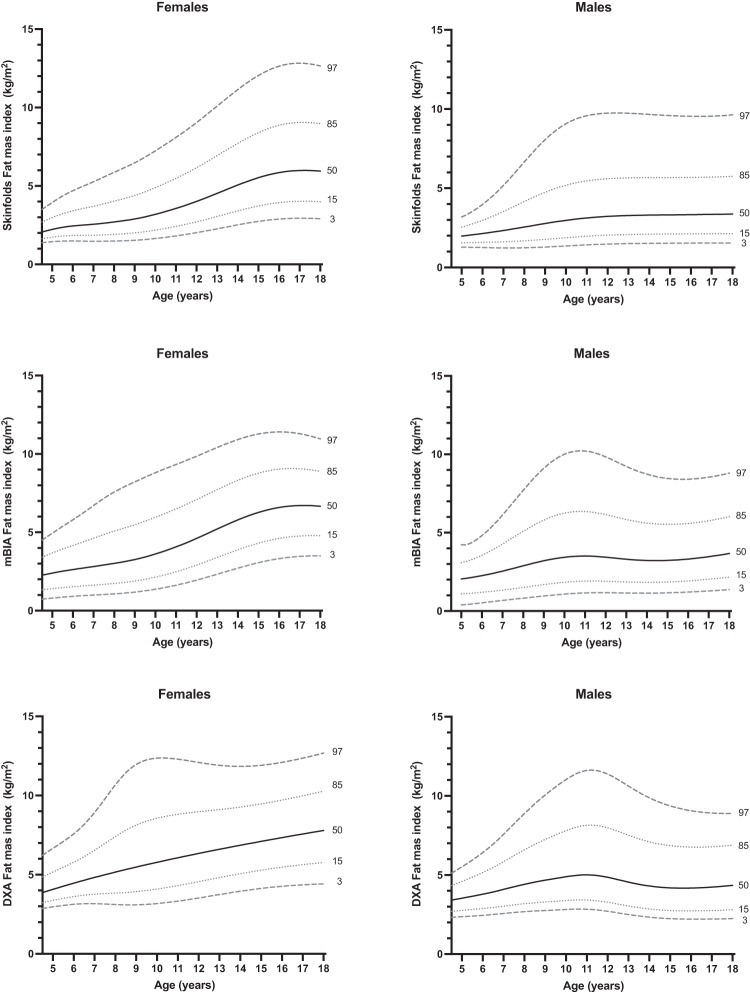
Percentile curves of fat mass index by skinfold thickness (upper), MF-BIA (middle) and DXA (lower) for females (left panels) and males (right panels).

**Fig. 2:**
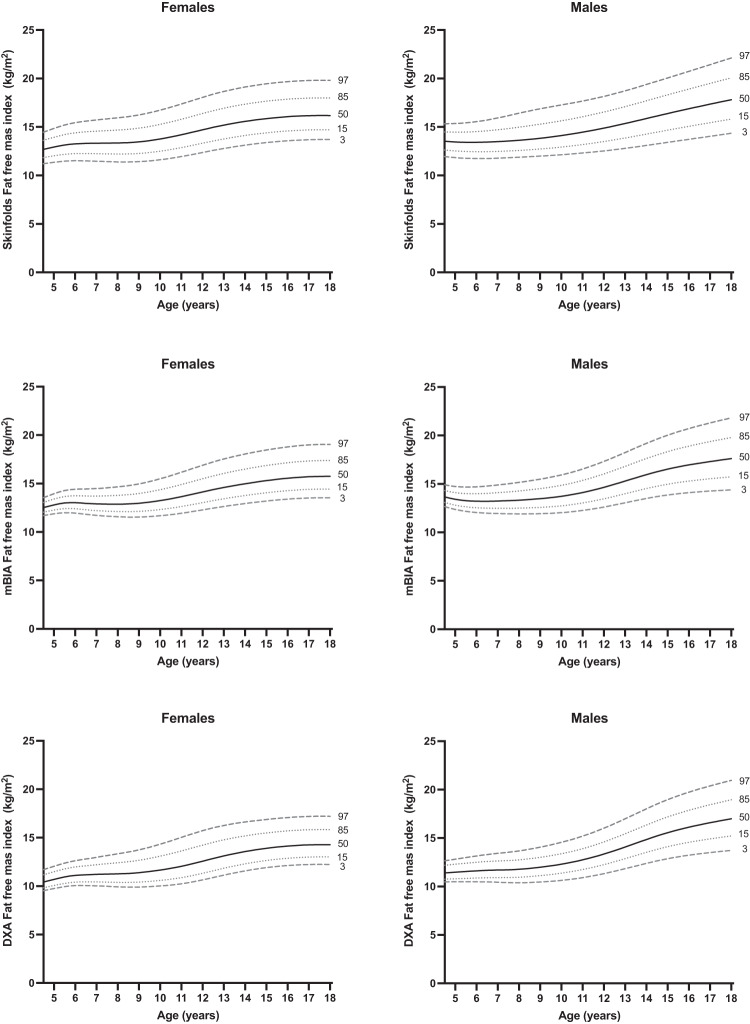
Percentile curves of fat-free mass index by skinfold thickness (upper), MF-BIA (middle) and DXA (lower) for females (left panels) and males (right panels).

The smoothed percentile curves of FMI and FFMI estimated by skinfolds thickness (SF), MF-BIA and DXA are shown in Figs. 1, and 2, the specific centiles of RV are presented in Tables 2 and 3.

**Table 2:** Smoothed percentiles for FMI for female and male 5 to 18 years, estimates by skinfolds thickness (upper), MF-BIA (middle) and DXA (lower).

SF FMI (kg/m^2^)													
Age	Female LMS parameters and percentile												
	L	S	1th	3th	5th	15th	25th	50th (M)	75th	85th	95th	97th	99th
5	−0.553	0.260	1.32	1.44	1.52	1.73	1.88	2.23	2.68	2.98	3.63	3.94	4.66
5.5	−0.518	0.279	1.35	1.48	1.56	1.80	1.97	2.36	2.88	3.23	3.98	4.35	5.20
6	−0.485	0.297	1.35	1.49	1.58	1.84	2.02	2.45	3.02	3.42	4.28	4.70	5.68
6.5	−0.455	0.314	1.33	1.48	1.58	1.85	2.05	2.51	3.13	3.56	4.52	4.99	6.09
7	−0.427	0.330	1.32	1.47	1.57	1.86	2.07	2.56	3.23	3.70	4.75	5.26	6.49
7.5	−0.401	0.345	1.31	1.47	1.57	1.88	2.10	2.62	3.35	3.86	5.00	5.56	6.91
8	−0.377	0.357	1.31	1.49	1.59	1.91	2.15	2.70	3.48	4.03	5.26	5.87	7.33
8.5	−0.355	0.367	1.32	1.50	1.61	1.95	2.20	2.79	3.62	4.20	5.51	6.16	7.72
9	−0.333	0.376	1.34	1.53	1.65	2.01	2.27	2.89	3.77	4.39	5.78	6.48	8.13
9.5	−0.313	0.382	1.38	1.58	1.71	2.08	2.36	3.03	3.96	4.62	6.10	6.84	8.58
10	−0.294	0.387	1.43	1.65	1.78	2.18	2.48	3.19	4.19	4.89	6.46	7.24	9.08
10.5	−0.275	0.391	1.50	1.73	1.87	2.30	2.61	3.37	4.43	5.18	6.84	7.66	9.59
11	−0.258	0.393	1.57	1.81	1.96	2.42	2.76	3.57	4.69	5.48	7.23	8.10	10.12
11.5	−0.241	0.394	1.65	1.91	2.07	2.56	2.92	3.78	4.98	5.81	7.65	8.56	10.66
12	−0.225	0.395	1.74	2.02	2.19	2.71	3.10	4.01	5.28	6.17	8.10	9.04	11.23
12.5	−0.210	0.395	1.84	2.14	2.32	2.88	3.29	4.27	5.61	6.54	8.57	9.56	11.83
13	−0.195	0.394	1.95	2.27	2.46	3.06	3.49	4.53	5.95	6.94	9.06	10.09	12.45
13.5	−0.181	0.394	2.05	2.39	2.60	3.24	3.70	4.80	6.30	7.33	9.56	10.63	13.07
14	−0.168	0.393	2.16	2.52	2.74	3.41	3.90	5.06	6.64	7.72	10.04	11.15	13.66
14.5	−0.154	0.393	2.26	2.64	2.87	3.58	4.09	5.31	6.96	8.08	10.48	11.63	14.21
15	−0.142	0.392	2.34	2.74	2.98	3.72	4.26	5.53	7.24	8.40	10.87	12.05	14.69
15.5	−0.129	0.392	2.41	2.83	3.08	3.85	4.41	5.71	7.47	8.67	11.20	12.39	15.07
16	−0.118	0.391	2.46	2.89	3.15	3.94	4.51	5.85	7.65	8.87	11.43	12.64	15.33
16.5	−0.106	0.391	2.49	2.93	3.19	4.00	4.58	5.94	7.77	9.00	11.57	12.79	15.47
17	−0.095	0.391	2.50	2.94	3.21	4.02	4.62	5.99	7.82	9.05	11.62	12.83	15.50
17.5	−0.084	0.391	2.49	2.93	3.20	4.02	4.61	5.99	7.81	9.04	11.59	12.78	15.41
18	−0.073	0.391	2.47	2.91	3.17	3.99	4.58	5.95	7.76	8.97	11.48	12.66	15.23
**Age**	**Male LMS parameters and percentile**												
	**L**	**S**	**1th**	**3th**	**5th**	**15th**	**25th**	**50th (M)**	**75th**	**85th**	**95th**	**97th**	**99th**
5	−0.188	0.235	1.15	1.28	1.35	1.55	1.69	1.98	2.33	2.55	2.99	3.19	3.63
5.5	−0.223	0.269	1.14	1.27	1.35	1.57	1.72	2.06	2.48	2.75	3.28	3.52	4.05
6	−0.255	0.304	1.12	1.26	1.34	1.58	1.76	2.14	2.65	2.98	3.66	3.98	4.68
6.5	−0.283	0.341	1.09	1.24	1.33	1.60	1.79	2.24	2.83	3.24	4.11	4.53	5.48
7	−0.307	0.376	1.08	1.23	1.33	1.62	1.83	2.33	3.04	3.53	4.63	5.18	6.46
7.5	−0.327	0.408	1.07	1.23	1.33	1.64	1.87	2.44	3.25	3.85	5.20	5.90	7.60
8	−0.343	0.435	1.07	1.24	1.34	1.68	1.93	2.55	3.47	4.16	5.79	6.66	8.83
8.5	−0.355	0.455	1.08	1.26	1.37	1.72	1.99	2.66	3.69	4.46	6.36	7.39	10.06
9	−0.364	0.470	1.10	1.29	1.40	1.77	2.05	2.77	3.88	4.74	6.88	8.06	11.19
9.5	−0.370	0.480	1.13	1.32	1.44	1.82	2.12	2.88	4.06	4.99	7.32	8.63	12.15
10	−0.374	0.485	1.16	1.36	1.48	1.87	2.18	2.97	4.21	5.19	7.67	9.07	12.88
10.5	−0.376	0.487	1.19	1.39	1.52	1.92	2.24	3.05	4.34	5.35	7.92	9.38	13.36
11	−0.376	0.486	1.22	1.42	1.55	1.97	2.29	3.12	4.43	5.46	8.09	9.58	13.63
11.5	−0.374	0.485	1.24	1.45	1.58	2.01	2.34	3.18	4.51	5.55	8.19	9.69	13.75
12	−0.372	0.482	1.26	1.47	1.61	2.04	2.37	3.22	4.56	5.60	8.25	9.74	13.76
12.5	−0.368	0.480	1.28	1.49	1.63	2.06	2.40	3.25	4.59	5.64	8.27	9.75	13.71
13	−0.364	0.478	1.29	1.51	1.64	2.08	2.41	3.28	4.62	5.66	8.27	9.73	13.63
13.5	−0.360	0.476	1.30	1.51	1.65	2.09	2.43	3.29	4.63	5.67	8.26	9.70	13.52
14	−0.356	0.474	1.30	1.52	1.66	2.10	2.44	3.30	4.64	5.67	8.24	9.66	13.41
14.5	−0.352	0.473	1.31	1.53	1.67	2.11	2.45	3.31	4.64	5.67	8.22	9.62	13.31
15	−0.347	0.472	1.31	1.53	1.67	2.11	2.45	3.32	4.65	5.67	8.20	9.59	13.22
15.5	−0.342	0.471	1.31	1.53	1.67	2.12	2.46	3.33	4.66	5.67	8.18	9.56	13.14
16	−0.338	0.471	1.31	1.54	1.68	2.12	2.47	3.33	4.66	5.68	8.18	9.54	13.08
16.5	−0.333	0.471	1.31	1.54	1.68	2.13	2.47	3.34	4.67	5.69	8.18	9.54	13.05
17	−0.328	0.471	1.31	1.54	1.68	2.13	2.48	3.35	4.69	5.70	8.19	9.55	13.05
17.5	−0.323	0.472	1.31	1.54	1.68	2.13	2.48	3.36	4.70	5.72	8.22	9.58	13.09
18	−0.319	0.473	1.31	1.54	1.68	2.14	2.49	3.37	4.72	5.75	8.27	9.64	13.17

MF-BIA FMI (kg/m^2^)													
Age	Female LMS parameters and percentile												
	L	S	1th	3th	5th	15th	25th	50th (M)	75th	85th	95th	97th	99th
5	0.463	0.457	0.56	0.80	0.95	1.41	1.72	2.40	3.20	3.68	4.57	4.95	5.70
5.5	0.381	0.468	0.62	0.86	1.01	1.48	1.80	2.52	3.40	3.93	4.95	5.38	6.26
6	0.307	0.478	0.67	0.92	1.07	1.53	1.87	2.62	3.57	4.16	5.30	5.80	6.83
6.5	0.242	0.489	0.72	0.96	1.11	1.58	1.93	2.72	3.74	4.39	5.68	6.24	7.44
7	0.189	0.502	0.75	1.00	1.15	1.63	1.99	2.82	3.91	4.63	6.07	6.71	8.09
7.5	0.149	0.512	0.79	1.03	1.19	1.68	2.05	2.92	4.08	4.86	6.45	7.17	8.73
8	0.121	0.517	0.82	1.07	1.23	1.74	2.11	3.02	4.25	5.08	6.79	7.57	9.28
8.5	0.104	0.518	0.86	1.12	1.28	1.80	2.19	3.13	4.41	5.28	7.08	7.91	9.74
9	0.095	0.513	0.92	1.18	1.35	1.89	2.29	3.26	4.59	5.48	7.35	8.22	10.11
9.5	0.092	0.505	0.99	1.27	1.44	2.00	2.42	3.42	4.79	5.71	7.63	8.52	10.45
10	0.095	0.493	1.07	1.37	1.55	2.14	2.58	3.62	5.02	5.96	7.91	8.80	10.75
10.5	0.104	0.479	1.17	1.49	1.68	2.30	2.76	3.83	5.27	6.22	8.18	9.07	11.01
11	0.118	0.463	1.29	1.62	1.83	2.48	2.96	4.07	5.53	6.49	8.44	9.33	11.23
11.5	0.136	0.446	1.42	1.78	2.00	2.69	3.19	4.33	5.82	6.78	8.72	9.59	11.44
12	0.157	0.429	1.56	1.95	2.19	2.91	3.43	4.61	6.12	7.09	9.01	9.86	11.66
12.5	0.180	0.412	1.72	2.13	2.39	3.15	3.69	4.91	6.44	7.41	9.30	10.14	11.88
13	0.205	0.395	1.88	2.33	2.59	3.40	3.96	5.21	6.76	7.73	9.60	10.42	12.11
13.5	0.231	0.380	2.05	2.52	2.81	3.65	4.23	5.51	7.07	8.03	9.88	10.68	12.32
14	0.256	0.366	2.22	2.72	3.01	3.89	4.49	5.79	7.36	8.32	10.14	10.92	12.51
14.5	0.282	0.353	2.38	2.90	3.21	4.11	4.73	6.05	7.62	8.57	10.36	11.12	12.67
15	0.307	0.342	2.52	3.06	3.38	4.31	4.94	6.27	7.84	8.78	10.53	11.27	12.77
15.5	0.332	0.332	2.65	3.21	3.53	4.48	5.11	6.45	8.01	8.93	10.65	11.37	12.82
16	0.357	0.323	2.75	3.32	3.66	4.61	5.25	6.59	8.12	9.03	10.71	11.41	12.81
16.5	0.382	0.315	2.82	3.41	3.74	4.71	5.34	6.67	8.18	9.07	10.70	11.38	12.73
17	0.407	0.308	2.87	3.46	3.80	4.77	5.40	6.71	8.19	9.06	10.64	11.29	12.59
17.5	0.432	0.302	2.90	3.49	3.83	4.79	5.42	6.71	8.15	8.99	10.52	11.15	12.39
18	0.456	0.297	2.91	3.50	3.84	4.79	5.41	6.67	8.08	8.89	10.36	10.96	12.15
**Age**	**Male LMS parameters and percentile**												
	**L**	**S**	**1th**	**3th**	**5th**	**15th**	**25th**	**50th (M)**	**75th**	**85th**	**95th**	**97th**	**99th**
5	0.951	0.453	0.15	0.39	0.57	1.10	1.43	2.04	2.69	3.08	3.86	4.23	5.07
5.5	0.777	0.480	0.19	0.45	0.62	1.14	1.48	2.14	2.87	3.28	4.04	4.36	5.00
6	0.622	0.505	0.27	0.53	0.69	1.19	1.54	2.26	3.08	3.56	4.45	4.81	5.54
6.5	0.488	0.530	0.36	0.61	0.76	1.26	1.61	2.39	3.33	3.90	4.96	5.41	6.32
7	0.377	0.552	0.44	0.68	0.84	1.34	1.70	2.55	3.61	4.28	5.57	6.13	7.28
7.5	0.290	0.571	0.51	0.75	0.91	1.42	1.81	2.71	3.91	4.69	6.23	6.92	8.36
8	0.223	0.584	0.57	0.82	0.98	1.51	1.91	2.89	4.21	5.09	6.89	7.70	9.45
8.5	0.171	0.591	0.64	0.89	1.05	1.60	2.02	3.05	4.49	5.47	7.51	8.45	10.51
9	0.129	0.594	0.70	0.96	1.13	1.69	2.13	3.21	4.74	5.80	8.05	9.11	11.45
9.5	0.096	0.594	0.76	1.02	1.20	1.77	2.22	3.34	4.94	6.07	8.48	9.64	12.20
10	0.070	0.590	0.81	1.08	1.26	1.84	2.29	3.43	5.08	6.25	8.78	10.00	12.74
10.5	0.049	0.585	0.85	1.13	1.30	1.89	2.34	3.49	5.16	6.34	8.94	10.19	13.03
11	0.035	0.579	0.88	1.16	1.33	1.91	2.37	3.51	5.17	6.35	8.95	10.21	13.08
11.5	0.026	0.572	0.90	1.17	1.34	1.92	2.36	3.49	5.12	6.28	8.84	10.08	12.91
12	0.022	0.566	0.90	1.17	1.34	1.90	2.34	3.43	5.02	6.15	8.63	9.83	12.57
12.5	0.023	0.560	0.90	1.16	1.33	1.88	2.31	3.37	4.91	6.00	8.38	9.53	12.15
13	0.027	0.553	0.89	1.15	1.32	1.86	2.27	3.31	4.79	5.84	8.12	9.23	11.72
13.5	0.033	0.547	0.89	1.14	1.31	1.84	2.25	3.26	4.70	5.71	7.90	8.95	11.32
14	0.041	0.540	0.89	1.14	1.30	1.83	2.23	3.23	4.63	5.61	7.72	8.73	10.99
14.5	0.050	0.534	0.89	1.15	1.31	1.84	2.24	3.22	4.60	5.55	7.59	8.56	10.73
15	0.060	0.527	0.90	1.16	1.33	1.85	2.25	3.23	4.59	5.53	7.52	8.46	10.54
15.5	0.072	0.521	0.92	1.18	1.35	1.88	2.29	3.26	4.62	5.54	7.49	8.41	10.43
16	0.083	0.514	0.94	1.21	1.38	1.92	2.33	3.32	4.67	5.59	7.51	8.41	10.38
16.5	0.095	0.509	0.96	1.24	1.42	1.97	2.39	3.38	4.74	5.66	7.57	8.45	10.38
17	0.108	0.503	0.99	1.28	1.46	2.03	2.45	3.47	4.84	5.76	7.66	8.54	10.44
17.5	0.121	0.498	1.02	1.32	1.50	2.09	2.53	3.56	4.95	5.88	7.78	8.66	10.54
18	0.134	0.493	1.06	1.36	1.55	2.16	2.61	3.67	5.08	6.02	7.93	8.80	10.68

DXA FMI (kg/m^2^)													
Age	Female LMS parameters and percentile												
	L	S	1th	3th	5th	15th	25th	50th (M)	75th	85th	95th	97th	99th
5	−1.106	0.202	2.79	2.97	3.07	3.37	3.59	4.07	4.72	5.16	6.16	6.66	7.89
5.5	−1.002	0.211	2.86	3.06	3.17	3.50	3.74	4.27	4.98	5.47	6.55	7.10	8.41
6	−0.908	0.223	2.92	3.13	3.25	3.62	3.88	4.46	5.25	5.79	6.98	7.57	9.01
6.5	−0.820	0.240	2.93	3.17	3.30	3.71	3.99	4.65	5.53	6.14	7.49	8.17	9.81
7	−0.740	0.263	2.91	3.17	3.32	3.77	4.09	4.83	5.84	6.54	8.12	8.93	10.89
7.5	−0.665	0.289	2.87	3.14	3.31	3.80	4.16	5.00	6.16	6.98	8.84	9.80	12.17
8	−0.595	0.314	2.81	3.11	3.29	3.83	4.23	5.16	6.47	7.41	9.57	10.68	13.46
8.5	−0.529	0.334	2.77	3.09	3.28	3.87	4.30	5.32	6.77	7.81	10.20	11.44	14.50
9	−0.466	0.348	2.76	3.10	3.30	3.93	4.39	5.48	7.03	8.13	10.66	11.96	15.14
9.5	−0.408	0.354	2.77	3.12	3.34	4.00	4.48	5.63	7.24	8.38	10.95	12.25	15.37
10	−0.352	0.355	2.80	3.17	3.40	4.09	4.59	5.78	7.42	8.57	11.11	12.36	15.32
10.5	−0.299	0.351	2.85	3.24	3.48	4.20	4.71	5.93	7.57	8.71	11.17	12.36	15.10
11	−0.248	0.344	2.92	3.33	3.57	4.31	4.84	6.07	7.71	8.82	11.17	12.29	14.82
11.5	−0.200	0.336	3.00	3.42	3.67	4.43	4.97	6.21	7.83	8.90	11.15	12.20	14.52
12	−0.153	0.327	3.09	3.52	3.78	4.56	5.11	6.34	7.93	8.98	11.11	12.09	14.22
12.5	−0.109	0.317	3.19	3.63	3.90	4.69	5.24	6.47	8.04	9.04	11.07	11.99	13.96
13	−0.066	0.307	3.28	3.74	4.02	4.82	5.38	6.60	8.14	9.11	11.04	11.91	13.74
13.5	−0.025	0.299	3.38	3.85	4.13	4.94	5.51	6.73	8.24	9.19	11.04	11.86	13.57
14	0.014	0.292	3.47	3.95	4.24	5.07	5.63	6.86	8.35	9.27	11.06	11.84	13.47
14.5	0.053	0.286	3.55	4.05	4.34	5.18	5.75	6.98	8.46	9.36	11.10	11.86	13.42
15	0.090	0.281	3.62	4.13	4.43	5.29	5.86	7.10	8.57	9.47	11.18	11.91	13.41
15.5	0.125	0.278	3.68	4.20	4.51	5.38	5.97	7.22	8.69	9.59	11.27	11.99	13.46
16	0.160	0.277	3.72	4.26	4.58	5.47	6.07	7.34	8.82	9.71	11.38	12.09	13.54
16.5	0.193	0.276	3.76	4.32	4.64	5.56	6.17	7.45	8.95	9.84	11.51	12.22	13.65
17	0.226	0.276	3.78	4.36	4.69	5.63	6.26	7.57	9.08	9.98	11.66	12.36	13.78
17.5	0.257	0.277	3.80	4.39	4.73	5.70	6.34	7.68	9.22	10.13	11.81	12.52	13.93
18	0.288	0.278	3.81	4.42	4.77	5.77	6.42	7.79	9.35	10.28	11.98	12.68	14.10
**Age**	**Male LMS parameters and percentile**												
	**L**	**S**	**1th**	**3th**	**5th**	**15th**	**25th**	**50th (M)**	**75th**	**85th**	**95th**	**97th**	**99th**
5	−0.234	0.233	2.20	2.36	2.46	2.76	2.99	3.54	4.22	4.60	5.25	5.51	6.02
5.5	−0.323	0.248	2.24	2.41	2.51	2.82	3.06	3.66	4.43	4.87	5.63	5.94	6.55
6	−0.401	0.264	2.29	2.45	2.55	2.88	3.13	3.79	4.65	5.16	6.05	6.42	7.14
6.5	−0.466	0.279	2.33	2.50	2.61	2.95	3.22	3.93	4.90	5.48	6.52	6.95	7.83
7	−0.518	0.294	2.39	2.57	2.68	3.03	3.32	4.09	5.18	5.84	7.06	7.58	8.62
7.5	−0.554	0.309	2.45	2.63	2.74	3.11	3.42	4.26	5.48	6.23	7.63	8.24	9.48
8	−0.574	0.323	2.50	2.68	2.80	3.19	3.51	4.41	5.75	6.59	8.19	8.88	10.33
8.5	−0.584	0.335	2.54	2.73	2.85	3.25	3.58	4.55	6.01	6.94	8.72	9.50	11.14
9	−0.586	0.347	2.57	2.76	2.88	3.30	3.65	4.67	6.24	7.25	9.20	10.06	11.88
9.5	−0.584	0.357	2.59	2.79	2.91	3.34	3.71	4.78	6.45	7.53	9.63	10.56	12.55
10	−0.580	0.366	2.62	2.82	2.95	3.39	3.77	4.89	6.66	7.80	10.04	11.03	13.15
10.5	−0.573	0.373	2.64	2.84	2.97	3.42	3.81	4.98	6.82	8.03	10.37	11.41	13.62
11	−0.563	0.379	2.63	2.84	2.97	3.42	3.82	5.01	6.91	8.14	10.55	11.61	13.87
11.5	−0.550	0.384	2.59	2.79	2.92	3.37	3.77	4.97	6.88	8.12	10.53	11.59	13.84
12	−0.532	0.388	2.51	2.71	2.84	3.28	3.67	4.85	6.75	7.98	10.34	11.37	13.54
12.5	−0.511	0.392	2.41	2.61	2.73	3.16	3.54	4.71	6.56	7.76	10.04	11.02	13.07
13	−0.486	0.394	2.32	2.50	2.62	3.04	3.41	4.55	6.36	7.51	9.69	10.62	12.53
13.5	−0.458	0.396	2.23	2.41	2.53	2.94	3.30	4.41	6.17	7.29	9.35	10.22	12.00
14	−0.428	0.398	2.16	2.34	2.45	2.85	3.21	4.31	6.02	7.10	9.06	9.88	11.53
14.5	−0.395	0.399	2.10	2.28	2.39	2.79	3.15	4.23	5.91	6.95	8.82	9.59	11.12
15	−0.359	0.399	2.07	2.24	2.36	2.75	3.11	4.19	5.84	6.85	8.64	9.36	10.79
15.5	−0.322	0.398	2.05	2.22	2.34	2.74	3.09	4.17	5.81	6.79	8.51	9.19	10.52
16	−0.283	0.398	2.03	2.21	2.33	2.73	3.09	4.17	5.80	6.76	8.41	9.06	10.30
16.5	−0.242	0.397	2.03	2.21	2.33	2.74	3.10	4.20	5.82	6.76	8.35	8.97	10.14
17	−0.201	0.395	2.03	2.22	2.34	2.76	3.13	4.24	5.85	6.78	8.33	8.91	10.02
17.5	−0.160	0.394	2.04	2.23	2.35	2.78	3.16	4.29	5.91	6.83	8.33	8.89	9.94
18	−0.119	0.393	2.06	2.25	2.37	2.81	3.20	4.35	5.98	6.89	8.35	8.89	9.90

**Table 3:** Smoothed percentiles for FFMI for female and male 5 to 18 years, estimates by skinfolds thickness (upper), MF-BIA (middle) and DXA (lower).

SF FFMI (kg/m^2^)													
Age	Female LMS parameters and percentile												
	L	S	1th	3th	5th	15th	25th	50th (M)	75th	85th	95th	97th	99th
5	−0.477	0.071	11.02	11.35	11.53	12.02	12.32	12.92	13.57	13.93	14.58	14.84	15.35
5.5	−0.522	0.075	11.12	11.47	11.66	12.18	12.50	13.13	13.82	14.21	14.91	15.19	15.75
6	−0.563	0.078	11.16	11.52	11.72	12.25	12.59	13.26	13.98	14.39	15.14	15.44	16.04
6.5	−0.600	0.081	11.13	11.50	11.71	12.26	12.61	13.30	14.06	14.50	15.28	15.60	16.24
7	−0.635	0.084	11.09	11.46	11.67	12.24	12.60	13.32	14.11	14.56	15.39	15.73	16.40
7.5	−0.668	0.086	11.04	11.43	11.64	12.22	12.59	13.33	14.15	14.62	15.48	15.84	16.55
8	−0.698	0.089	11.00	11.40	11.61	12.21	12.59	13.35	14.19	14.68	15.57	15.94	16.69
8.5	−0.727	0.091	10.99	11.39	11.61	12.22	12.60	13.38	14.25	14.76	15.68	16.07	16.84
9	−0.754	0.093	11.02	11.42	11.65	12.27	12.66	13.46	14.35	14.88	15.84	16.24	17.05
9.5	−0.779	0.095	11.09	11.50	11.73	12.36	12.77	13.59	14.51	15.05	16.04	16.46	17.30
10	−0.804	0.096	11.20	11.62	11.85	12.50	12.91	13.76	14.71	15.26	16.30	16.73	17.61
10.5	−0.827	0.098	11.34	11.77	12.01	12.67	13.09	13.96	14.94	15.51	16.58	17.03	17.95
11	−0.849	0.099	11.51	11.95	12.19	12.87	13.30	14.19	15.20	15.79	16.90	17.37	18.32
11.5	−0.869	0.099	11.71	12.15	12.40	13.09	13.54	14.45	15.48	16.09	17.23	17.71	18.70
12	−0.890	0.100	11.91	12.37	12.62	13.32	13.78	14.71	15.76	16.39	17.56	18.06	19.07
12.5	−0.909	0.100	12.12	12.58	12.84	13.55	14.01	14.96	16.04	16.68	17.87	18.38	19.41
13	−0.927	0.100	12.31	12.78	13.04	13.76	14.23	15.19	16.28	16.93	18.14	18.66	19.72
13.5	−0.945	0.099	12.49	12.96	13.22	13.95	14.42	15.39	16.50	17.16	18.38	18.91	19.98
14	−0.962	0.099	12.65	13.12	13.39	14.12	14.60	15.57	16.69	17.35	18.59	19.12	20.20
14.5	−0.979	0.099	12.79	13.26	13.53	14.27	14.75	15.73	16.85	17.52	18.77	19.30	20.39
15	−0.995	0.098	12.91	13.39	13.65	14.39	14.87	15.86	16.99	17.66	18.91	19.45	20.55
15.5	−1.010	0.098	13.01	13.49	13.76	14.50	14.98	15.97	17.10	17.77	19.04	19.58	20.68
16	−1.025	0.097	13.10	13.58	13.84	14.59	15.07	16.06	17.19	17.87	19.13	19.68	20.79
17	−1.054	0.097	13.20	13.68	13.95	14.69	15.18	16.17	17.30	17.98	19.25	19.79	20.91
17.5	−1.067	0.097	13.23	13.70	13.97	14.71	15.19	16.18	17.31	17.99	19.27	19.81	20.93
18	−1.081	0.097	13.23	13.71	13.97	14.71	15.19	16.18	17.31	17.99	19.26	19.80	20.93
**Age**	**Male LMS parameters and percentile**												
	**L**	**S**	**1th**	**3th**	**5th**	**15th**	**25th**	**50th (M)**	**75th**	**85th**	**95th**	**97th**	**99th**
5	−0.268	0.069	11.49	11.84	12.03	12.53	12.84	13.45	14.10	14.46	15.10	15.36	15.86
5.5	−0.449	0.072	11.42	11.77	11.96	12.47	12.79	13.42	14.09	14.47	15.15	15.42	15.96
6	−0.707	0.074	11.40	11.74	11.93	12.45	12.77	13.42	14.12	14.52	15.25	15.54	16.13
6.5	−1.030	0.077	11.41	11.75	11.94	12.45	12.78	13.44	14.17	14.60	15.39	15.71	16.37
7	−1.382	0.079	11.45	11.78	11.96	12.48	12.81	13.48	14.25	14.71	15.56	15.93	16.67
7.5	−1.693	0.081	11.50	11.82	12.01	12.52	12.85	13.55	14.35	14.83	15.76	16.17	17.01
8	−1.907	0.083	11.55	11.88	12.06	12.58	12.91	13.62	14.46	14.97	15.97	16.41	17.36
8.5	−2.001	0.085	11.61	11.94	12.12	12.65	12.99	13.72	14.59	15.12	16.18	16.65	17.67
9	−1.978	0.087	11.66	12.00	12.19	12.73	13.08	13.83	14.73	15.28	16.38	16.87	17.94
9.5	−1.857	0.089	11.71	12.06	12.26	12.82	13.19	13.96	14.88	15.45	16.57	17.07	18.15
10	−1.673	0.091	11.77	12.14	12.34	12.92	13.31	14.11	15.05	15.64	16.76	17.26	18.33
10.5	−1.453	0.093	11.83	12.22	12.44	13.05	13.45	14.28	15.25	15.84	16.97	17.46	18.50
11	−1.220	0.095	11.90	12.31	12.54	13.18	13.60	14.46	15.46	16.05	17.19	17.67	18.69
11.5	−0.991	0.096	11.98	12.41	12.66	13.33	13.77	14.66	15.68	16.29	17.42	17.90	18.89
12	−0.778	0.098	12.07	12.53	12.79	13.50	13.95	14.88	15.93	16.54	17.68	18.15	19.12
12.5	−0.590	0.100	12.16	12.65	12.93	13.67	14.15	15.11	16.18	16.81	17.95	18.43	19.38
13	−0.432	0.101	12.27	12.79	13.08	13.86	14.36	15.36	16.46	17.09	18.25	18.72	19.68
13.5	−0.303	0.103	12.40	12.94	13.24	14.06	14.58	15.61	16.74	17.39	18.56	19.04	20.00
14	−0.201	0.104	12.53	13.10	13.41	14.26	14.80	15.87	17.03	17.70	18.89	19.38	20.34
14.5	−0.120	0.106	12.66	13.25	13.58	14.47	15.02	16.13	17.32	18.00	19.22	19.72	20.69
15	−0.057	0.107	12.80	13.41	13.75	14.67	15.24	16.38	17.61	18.31	19.55	20.06	21.05
15.5	−0.010	0.108	12.93	13.57	13.92	14.87	15.46	16.63	17.90	18.61	19.88	20.40	21.41
16	0.020	0.110	13.07	13.72	14.09	15.06	15.67	16.88	18.17	18.91	20.21	20.74	21.77
17	0.030	0.112	13.34	14.03	14.41	15.44	16.08	17.35	18.72	19.49	20.87	21.42	22.52
17.5	0.012	0.114	13.49	14.19	14.58	15.63	16.28	17.58	18.98	19.78	21.19	21.77	22.90
18	−0.016	0.115	13.63	14.35	14.74	15.81	16.48	17.81	19.25	20.07	21.52	22.12	23.29

MF-BIA FFMI (kg/m^2^)													
Age	Female LMS parameters and percentile												
	L	S	1th	3th	5th	15th	25th	50th (M)	75th	85th	95th	97th	99th
5	−1.862	0.042	12.14	12.36	12.48	12.81	13.01	13.41	13.85	14.10	14.55	14.74	15.10
5.5	−1.825	0.046	11.94	12.17	12.29	12.63	12.85	13.27	13.74	14.00	14.48	14.67	15.06
6	−1.790	0.049	11.81	12.05	12.18	12.54	12.77	13.21	13.70	13.98	14.49	14.70	15.11
6.5	−1.759	0.053	11.73	11.98	12.12	12.50	12.74	13.21	13.72	14.02	14.56	14.78	15.22
7	−1.730	0.056	11.68	11.95	12.09	12.49	12.74	13.24	13.78	14.10	14.66	14.90	15.36
7.5	−1.703	0.059	11.64	11.92	12.07	12.49	12.75	13.27	13.85	14.18	14.77	15.02	15.51
8	−1.678	0.062	11.62	11.90	12.06	12.50	12.77	13.32	13.92	14.27	14.90	15.16	15.67
8.5	−1.654	0.065	11.61	11.91	12.08	12.53	12.82	13.39	14.02	14.39	15.04	15.32	15.86
9	−1.632	0.068	11.61	11.93	12.10	12.58	12.88	13.48	14.14	14.52	15.21	15.49	16.06
9.5	−1.611	0.071	11.64	11.97	12.15	12.64	12.96	13.58	14.27	14.67	15.39	15.69	16.29
10	−1.591	0.074	11.69	12.03	12.22	12.74	13.07	13.72	14.44	14.86	15.62	15.93	16.55
10.5	−1.572	0.077	11.77	12.13	12.33	12.87	13.21	13.90	14.65	15.09	15.88	16.21	16.87
11	−1.554	0.079	11.89	12.26	12.47	13.04	13.40	14.11	14.91	15.37	16.20	16.54	17.23
11.5	−1.536	0.081	12.03	12.43	12.64	13.24	13.61	14.37	15.20	15.68	16.55	16.91	17.64
12	−1.520	0.083	12.20	12.62	12.84	13.47	13.86	14.65	15.53	16.03	16.95	17.33	18.08
12.5	−1.504	0.084	12.39	12.83	13.07	13.72	14.13	14.96	15.88	16.41	17.37	17.77	18.57
13	−1.488	0.085	12.60	13.05	13.30	13.99	14.42	15.29	16.26	16.81	17.82	18.24	19.08
13.5	−1.474	0.086	12.80	13.28	13.54	14.26	14.72	15.63	16.64	17.22	18.28	18.72	19.59
14	−1.459	0.087	13.00	13.50	13.77	14.52	15.00	15.96	17.01	17.62	18.73	19.19	20.10
14.5	−1.446	0.087	13.17	13.69	13.98	14.76	15.26	16.26	17.36	17.99	19.15	19.63	20.58
15	−1.432	0.088	13.32	13.86	14.15	14.97	15.49	16.52	17.67	18.33	19.53	20.03	21.02
15.5	−1.420	0.088	13.43	13.99	14.30	15.14	15.68	16.76	17.94	18.63	19.87	20.39	21.41
16	−1.407	0.088	13.52	14.10	14.42	15.29	15.85	16.96	18.19	18.90	20.18	20.72	21.77
17	−1.383	0.089	13.65	14.26	14.60	15.53	16.12	17.31	18.61	19.37	20.73	21.29	22.42
17.5	−1.372	0.089	13.70	14.33	14.68	15.64	16.24	17.46	18.80	19.58	20.98	21.56	22.71
18	−1.361	0.089	13.74	14.39	14.75	15.73	16.36	17.61	18.99	19.79	21.23	21.82	23.00
**Age**	**Male LMS parameters and percentile**												
	**L**	**S**	**1th**	**3th**	**5th**	**15th**	**25th**	**50th (M)**	**75th**	**85th**	**95th**	**97th**	**99th**
5	−1.634	0.046	11.69	11.88	11.98	12.27	12.44	12.80	13.18	13.40	13.79	13.95	14.27
5.5	−1.534	0.049	11.78	11.99	12.11	12.41	12.61	12.99	13.42	13.66	14.10	14.28	14.64
6	−1.442	0.052	11.73	11.95	12.07	12.40	12.61	13.02	13.48	13.74	14.22	14.42	14.81
6.5	−1.358	0.055	11.60	11.83	11.95	12.30	12.52	12.96	13.44	13.72	14.23	14.45	14.87
7	−1.280	0.058	11.48	11.72	11.85	12.21	12.44	12.90	13.41	13.71	14.26	14.48	14.94
7.5	−1.207	0.061	11.39	11.64	11.77	12.15	12.39	12.88	13.42	13.73	14.32	14.56	15.05
8	−1.139	0.064	11.32	11.58	11.72	12.11	12.37	12.88	13.45	13.78	14.40	14.66	15.18
8.5	−1.076	0.066	11.27	11.54	11.69	12.10	12.36	12.90	13.50	13.85	14.51	14.78	15.35
9	−1.015	0.069	11.27	11.55	11.70	12.13	12.41	12.97	13.60	13.98	14.67	14.97	15.57
9.5	−0.958	0.072	11.30	11.60	11.76	12.21	12.50	13.09	13.75	14.15	14.89	15.21	15.85
10	−0.904	0.074	11.38	11.68	11.85	12.32	12.62	13.24	13.95	14.37	15.16	15.50	16.19
10.5	−0.853	0.077	11.47	11.79	11.97	12.46	12.78	13.43	14.17	14.62	15.46	15.82	16.56
11	−0.804	0.079	11.60	11.93	12.12	12.63	12.96	13.65	14.43	14.90	15.79	16.17	16.96
11.5	−0.757	0.082	11.76	12.10	12.30	12.83	13.17	13.89	14.71	15.20	16.13	16.53	17.37
12	−0.712	0.084	11.93	12.29	12.48	13.04	13.39	14.13	14.99	15.50	16.47	16.89	17.77
12.5	−0.669	0.086	12.10	12.47	12.67	13.24	13.61	14.37	15.26	15.79	16.79	17.23	18.14
13	−0.628	0.089	12.26	12.64	12.85	13.43	13.81	14.59	15.50	16.05	17.08	17.53	18.47
13.5	−0.588	0.091	12.41	12.80	13.01	13.61	13.99	14.80	15.72	16.28	17.34	17.80	18.76
14	−0.550	0.093	12.55	12.94	13.16	13.77	14.16	14.98	15.92	16.49	17.58	18.05	19.02
14.5	−0.513	0.095	12.68	13.08	13.30	13.92	14.32	15.15	16.11	16.69	17.79	18.27	19.26
15	−0.477	0.098	12.80	13.20	13.42	14.05	14.46	15.30	16.28	16.86	17.98	18.46	19.47
15.5	−0.443	0.100	12.90	13.31	13.53	14.17	14.58	15.44	16.42	17.02	18.15	18.64	19.65
16	−0.409	0.102	12.99	13.40	13.63	14.27	14.69	15.55	16.55	17.15	18.29	18.79	19.81
17	−0.345	0.106	13.09	13.51	13.75	14.40	14.83	15.70	16.72	17.33	18.49	18.99	20.03
17.5	−0.315	0.108	13.11	13.53	13.77	14.43	14.85	15.74	16.75	17.37	18.53	19.03	20.08
18	−0.285	0.111	13.11	13.53	13.77	14.43	14.86	15.74	16.76	17.38	18.54	19.04	20.09

DXA FFMI (kg/m^2^)													
Age	Female LMS parameters and percentile												
	L	S	1th	3th	5th	15th	25th	50th (M)	75th	85th	95th	97th	99th
5	−2.411	0.058	9.63	9.75	9.83	10.07	10.25	10.70	11.22	11.48	11.90	12.05	12.33
5.5	−2.315	0.060	9.81	9.95	10.03	10.28	10.48	10.95	11.49	11.77	12.21	12.38	12.68
6	−2.227	0.062	9.91	10.06	10.14	10.41	10.62	11.11	11.69	11.98	12.45	12.63	12.95
6.5	−2.146	0.066	9.90	10.06	10.15	10.44	10.65	11.17	11.78	12.10	12.61	12.80	13.16
7	−2.071	0.070	9.86	10.03	10.13	10.43	10.66	11.21	11.86	12.21	12.76	12.97	13.37
7.5	−2.002	0.075	9.80	9.98	10.09	10.41	10.66	11.25	11.94	12.32	12.93	13.16	13.61
8	−1.937	0.080	9.74	9.93	10.04	10.39	10.65	11.27	12.01	12.41	13.08	13.34	13.83
8.5	−1.876	0.084	9.69	9.90	10.02	10.38	10.66	11.31	12.10	12.52	13.24	13.52	14.06
9	−1.818	0.088	9.69	9.91	10.03	10.42	10.70	11.39	12.22	12.67	13.44	13.74	14.33
9.5	−1.763	0.092	9.72	9.95	10.08	10.49	10.79	11.51	12.38	12.86	13.69	14.02	14.66
10	−1.712	0.096	9.78	10.02	10.16	10.59	10.91	11.66	12.58	13.09	13.98	14.33	15.03
10.5	−1.663	0.100	9.86	10.11	10.26	10.72	11.05	11.84	12.81	13.35	14.29	14.68	15.43
11	−1.616	0.102	9.98	10.25	10.40	10.88	11.23	12.05	13.06	13.63	14.63	15.04	15.84
11.5	−1.571	0.104	10.15	10.42	10.59	11.08	11.45	12.30	13.35	13.93	14.97	15.40	16.24
12	−1.528	0.104	10.36	10.65	10.82	11.33	11.70	12.58	13.64	14.24	15.30	15.73	16.59
12.5	−1.487	0.103	10.59	10.89	11.07	11.59	11.97	12.85	13.92	14.52	15.59	16.02	16.88
13	−1.447	0.101	10.83	11.14	11.32	11.85	12.23	13.12	14.18	14.78	15.83	16.26	17.11
13.5	−1.409	0.099	11.06	11.37	11.55	12.09	12.47	13.36	14.41	15.00	16.04	16.46	17.29
14	−1.372	0.097	11.26	11.58	11.76	12.31	12.69	13.57	14.61	15.19	16.21	16.62	17.44
14.5	−1.337	0.095	11.44	11.76	11.95	12.49	12.88	13.75	14.78	15.35	16.35	16.76	17.56
15	−1.303	0.093	11.59	11.91	12.10	12.65	13.04	13.90	14.92	15.49	16.48	16.88	17.67
15.5	−1.270	0.092	11.71	12.03	12.22	12.78	13.17	14.03	15.04	15.61	16.59	16.99	17.77
16	−1.238	0.092	11.79	12.13	12.32	12.88	13.27	14.14	15.14	15.70	16.68	17.08	17.86
17	−1.177	0.091	11.89	12.23	12.43	13.00	13.39	14.26	15.26	15.82	16.80	17.20	17.98
17.5	−1.147	0.091	11.90	12.25	12.44	13.02	13.41	14.28	15.28	15.84	16.82	17.22	18.00
18	−1.119	0.091	11.89	12.24	12.44	13.02	13.41	14.28	15.28	15.84	16.82	17.21	18.00
**Age**	**Male LMS parameters and percentile**												
	**L**	**S**	**1th**	**3th**	**5th**	**15th**	**25th**	**50th (M)**	**75th**	**85th**	**95th**	**97th**	**99th**
5	−2.030	0.057	10.36	10.48	10.56	10.79	10.99	11.47	12.03	12.30	12.69	12.82	13.05
5.5	−1.874	0.060	10.34	10.48	10.57	10.83	11.04	11.54	12.12	12.40	12.83	12.98	13.26
6	−1.732	0.063	10.32	10.48	10.58	10.88	11.09	11.61	12.20	12.50	12.98	13.15	13.47
6.5	−1.601	0.065	10.28	10.47	10.58	10.91	11.14	11.67	12.27	12.58	13.10	13.30	13.67
7	−1.479	0.068	10.22	10.44	10.56	10.92	11.16	11.70	12.31	12.64	13.20	13.42	13.85
7.5	−1.366	0.070	10.16	10.40	10.54	10.92	11.18	11.72	12.34	12.68	13.29	13.53	14.02
8	−1.261	0.073	10.11	10.39	10.54	10.95	11.22	11.77	12.39	12.75	13.40	13.67	14.21
8.5	−1.161	0.075	10.11	10.41	10.57	11.02	11.31	11.87	12.49	12.86	13.56	13.85	14.45
9	−1.068	0.077	10.13	10.46	10.64	11.12	11.42	11.99	12.62	13.01	13.74	14.06	14.72
9.5	−0.979	0.079	10.18	10.53	10.73	11.24	11.55	12.13	12.78	13.18	13.96	14.30	15.00
10	−0.895	0.081	10.25	10.63	10.84	11.38	11.70	12.30	12.96	13.38	14.20	14.56	15.31
10.5	−0.815	0.083	10.34	10.76	10.98	11.55	11.89	12.51	13.19	13.62	14.48	14.86	15.66
11	−0.739	0.086	10.48	10.91	11.15	11.75	12.11	12.75	13.46	13.91	14.80	15.20	16.05
11.5	−0.666	0.087	10.64	11.10	11.35	11.99	12.36	13.03	13.76	14.24	15.17	15.59	16.48
12	−0.596	0.089	10.83	11.33	11.59	12.26	12.65	13.35	14.11	14.60	15.58	16.02	16.95
12.5	−0.530	0.091	11.05	11.57	11.85	12.56	12.97	13.70	14.50	15.01	16.03	16.49	17.46
13	−0.465	0.093	11.29	11.84	12.13	12.87	13.30	14.07	14.91	15.44	16.51	16.99	17.99
13.5	−0.404	0.095	11.54	12.11	12.42	13.20	13.65	14.46	15.33	15.89	17.01	17.50	18.54
14	−0.344	0.097	11.78	12.39	12.71	13.53	14.00	14.85	15.76	16.35	17.50	18.02	19.09
14.5	−0.287	0.099	12.01	12.64	12.98	13.84	14.33	15.22	16.17	16.78	17.98	18.51	19.62
15	−0.231	0.100	12.21	12.87	13.22	14.11	14.63	15.55	16.55	17.18	18.42	18.96	20.10
15.5	−0.177	0.102	12.39	13.07	13.43	14.36	14.89	15.85	16.89	17.54	18.82	19.37	20.53
16	−0.125	0.104	12.53	13.23	13.61	14.57	15.12	16.12	17.19	17.87	19.17	19.74	20.91
17	−0.026	0.107	12.76	13.51	13.90	14.92	15.51	16.58	17.73	18.44	19.80	20.38	21.58
17.5	0.022	0.109	12.86	13.62	14.03	15.07	15.68	16.79	17.97	18.70	20.08	20.67	21.88
18	0.068	0.110	12.95	13.73	14.15	15.22	15.85	16.99	18.21	18.95	20.36	20.96	22.18

Detailed specific centiles of RV for anthropometric, MF-BIA and DXA variables are shown in part the supplementary material (Tables 2–44).

Specific RV for BMC for our sample have been previously published [32].

The three methods showed similar patterns of data distribution and differences in BC based on sex were evident. Compared with that of males, BC of females showed a significantly greater amount and proportion of FM components, in contrast males showed a significantly greater amount and proportion of LM and BMC components. Data’s graphical representation made evident a greater amount of FM observed in females, with a positive inflection starting at 8–9 y age. In contrast, males showed greater amounts of LM, with a positive inflection starting at the beginning of adolescence. Data was also analyzed by Tanner pubertal stage (1–5), where females showed increasing amounts of FM and LM in Tanner stages 1 to 4 and relative stability upon reaching stage 5, while males showed increasing amounts of LM and relatively stable amounts of FM in all Tanner stages (data shown in supplementary fig. 3).

**Fig. 3:**
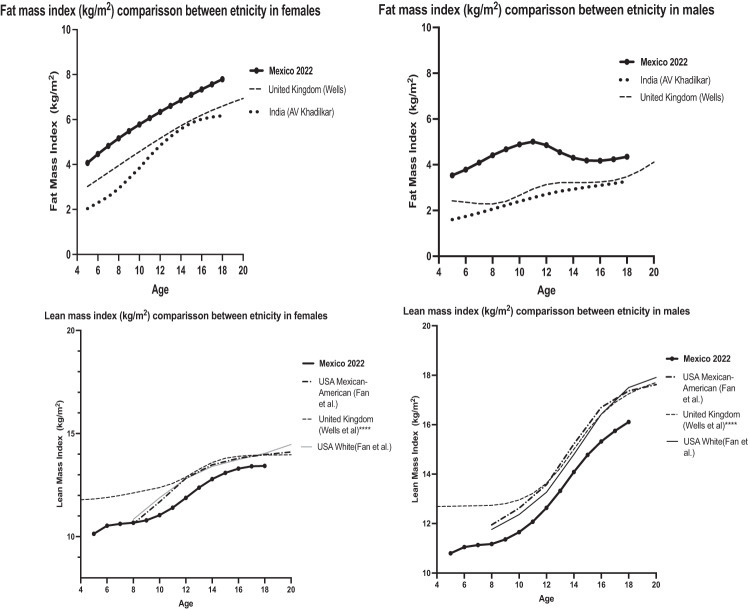
Comparison between the behavior of reference values published in other studies, estimated by DXA (Lunar). Upper: FMI, Lower: LMI.

The comparisons between our RV of FMI, LMI and FM% estimated by DXA with those previously published in other studies using the same device (Lunar) and reported or made available to us in the same format are illustrated in Fig. 3 and supplementary fig. 4.

Our data showed similar values of FM% to those from Mexican-Americans [30] but FMI significantly higher than those of UK [12] and India [31], Kruskal–Wallis statistics for females 8.6 (*P* value = 0.013); for males statistics 28.2 (*P* value < 0.001). Regarding LMI our data showed lower values than those reported for USA and UK, Kruskal–Wallis statistics for females 7.6 (*P* value = 0.022); for males statistics 6.5 (*P* value = 0.039). No other comparisons were made because of differences in methods and or reporting format of data.

## Discussion

The availability of population-specific RV is important because variations in the growth of the human body among different populations are influenced mainly by genetic, endocrine, physiological, environmental, and cultural factors over time [33]. These differences are recognized by functional and adaptive growth patterns, and their description has been clinically relevant not only to physically characterize different populations but also to allow the identification of members of a particular population that present atypical or extreme variations from the specific-population pattern and their possible association with a health outcome [34].

In this study we report valid RV of BC by anthropometry, mBIA and DXA for the urban Mexican pediatric population. We provide values for the 1st, 3rd, 5th, 15th, 25th, 50th, 75th, 85th, 95th, 97th and 99th centiles at 0.25 years intervals for subjects aged 5 to 18 years, for each sex and for each variable, along with *lambda* (L), *mu* (M) and *sigma* (S) values with corresponding formulas to enable Z-scores estimations for clinical use. We also provide sex and age-smoothed centile graphs, and as supplementary material RV for other related variables. We believe these RV provided for the three most relevant methods used in the clinical assessment of BC, will facilitate and increase the accuracy of its assessment in several clinical and research contexts. Despite each method provides precise estimates of BC, as it has been previously published they are not interchangeable between them, therefore this study addresses the need of such population-specific RV for each method [22, 35–39].

As a result of this study, we have been able to characterize the BC of the population of interest and generate RV that will allow the identification of subjects with abnormal (net, relative and/or adjusted) BC values and facilitate their classification. Immediate clinical applicability lies in the increased accuracy of individual evaluation of subjects with particular clinical conditions, where knowledge and quantification of BC is relevant (e.g., associations between low LM, cancer prognosis, chemotherapy tolerance, prognosis with acute events requiring hospitalization, prostration [35, 36], relationships between increased FM content and insulin resistance) for whichever of the three methods is used [33]. The availability of RV for the three methods is especially relevant for our country, where the reference standard for clinical purposes (DXA) is available only in few centers, but mBIA and anthropometry are so countrywide, and because neither of mBIA nor anthropometry expose subjects to radiation, these may be more appropriate for those subjects who require close monitoring and multiple measurements of BC (i.e., athletes, individuals participating in nutritional interventions, exercise interventions, pharmacological interventions, etc.).

As our data has shown, the BC of the healthy Mexican urban pediatric population characterizes preschoolers and school-age children of both sexes as having similar total body weight and BC compartment distribution (i.e., FM and LM). In adolescence, despite maintaining similar total body weights, females show significantly more FM and less LM. In early adulthood, the difference in total body weight becomes evident, and the differences in BC are accentuated, with females, having a lower total body weight, greater amount of FM, and a lower amount of LM. These differences increase after the pubertal growth spurt until reaching their maximum discrepancy in late adolescence and adulthood. Similar data behaviors and sex-differences, although of different magnitudes have been previously described for other populations [34, 40, 41].

Comparisons of our data with that of previously reported RV of BC for other populations made patent significant differences. Our data showed higher values of the FM compartment than those for IND [31], and UK [12] and values of the LM compartment lower than those for the UK, and USA [30]. Comparisons were limited mainly because of significant heterogeneity of available data of RV of BC estimated by DXA previously published, including some studies reporting results as FMI and FFMI while others %FM or FM, and the use of different DXA devices (Lunar vs Hologic) which are not interchangeable. Besides ethnicity other potential factors may explain reported differences, such as the decade data was collected, sampling methods, criteria to define the healthy status of the sample, etc. The investigation of the determinants of these differences is deeper, more complex, and not within the scope of this study. However, such differences justify the need for specific values for each population to improve accuracy of the evaluations of individuals belonging to such particular population.

The characterization of the BC of the studied sample makes evident that a significant accumulation of adipose tissue (FM) occurs without leaving a clear signal in BMI and prior to the development of metabolic alterations defining an altered state of health. Although BMI correlates well with adiposity and is a tool with adequate clinical performance at the population level to identify subjects with OW/OB, it has important limitations in individual clinical evaluations. BMI can and often misclassify athletes, subjects with edema, and subjects with the accumulation of tissues other than fat as being OW/OB; BMI can also classify subjects with sarcopenia or sarcopenic obesity as subjects with a healthy weight. All these clinical conditions can be better evaluated by BC. Another important limitation of BMI is the low sensitivity to changes in BC. The most frequent example is a subject correctly identified by BMI as OW/OB who initiates a nutritional intervention coupled with physical activity that results in an increase in LM and a decrease in FM without significant changes in weight and, therefore minimal changes in BMI. In the individual clinical evaluation of these subjects, evaluating BC adds accuracy, sensitivity to change and clinical value in decision-making.

RV should ideally characterize healthy subjects of the population of interest and summarize data on those characteristics that represent such healthy state, which is not necessarily “normal” in a population. If we consider the estimated prevalence of 30% OW/OB in children and adolescents of Mexico and 70% for its adult population and should generate RV based on the distribution of BMI at the population level, we could most likely end up normalizing OW/OB. In other words, the distribution of measured values of physical characteristics of a population is not necessarily similar to the distribution of the values measured and related to a state of health. Therefore, we believe that a strength of this study is the stringent criteria (clinical and metabolic) applied to define the healthy status of our sample.

Our urban-population-based recruitment approach may be considered a limitation for the representativeness for the whole Mexican children and adolescent population of the country. However, comparison of our data to that of ENSANUT (which is considered representative of the population of whole country) identified significant differences only for five of the 17 groups studied. Therefore, we believe there is positive evidence to support the clinical adoption of our RV instead of those of other populations as an improvement of current practice applicable to the clinical assessment of BC in Mexican children and adolescents.

### Study limitations

As previously discussed, the RV published here are representative of the urban children and adolescent population of Mexico City and Metropolitan Area, a region that accounts for approximately 20% of the population of the country and where the best living conditions have been estimated (i.e., indices of life expectancy, literacy, school enrolment, education level, GDP per capita, human development, and degree of human development) [42]. This is relevant because, based on ENSANUT data, there are significant differences between the population in the north of the country and those in the southeast, i.e., the population in the north has a higher prevalence of OW/OB, and the population in the southeast has a higher prevalence of malnutrition [32]. It is important for this study to be validated in populations from different regions of the country, rural environments, and indigenous populations. However, it is also important to recognize that by increasing the representativeness of these populations in the RV, there is a risk of modifying their clinical performance. Specifically, increasing the representativeness would allow the measurement values for populations that have a higher prevalence of some undesirable clinical health conditions (OW, OB, and malnutrition) as well as less adequate social conditions.

Another relative limitation is the cross-sectional nature of the study, which does not allow characterization of the growth patterns of the studied population. The RV and the corresponding smoothed curves are based on GAMLSS for the data and interpolation equations to generate these values and patterns. However, GAMLSS together with its predecessor method, i.e., LMS, are the standard methods used worldwide for the generation of reference values of this nature.

### Future research

We considered relevant to model RV and their corresponding smoothed curves for the sub-compartments of FM estimated by DXA (i.e. truncal fat mass (tkFM) [43], android fat mass (aFM) and gynoid fat mass (gFM) values) [44]. Despite these parameters have not yet been clinically validated as biomarkers related to specific risks for negative health outcomes, typical distributions of two types of adipose have [1]. Adipose tissue that accumulates preferentially at the central level (i.e., abdomen) and is usually metabolically active (i.e., adipokine-secreting) has been related to insulin resistance as well as other negative health outcomes. This central distribution is more frequent in males, which is why it is known as android fat [2]. Conversely, peripheral adipose tissue functions mainly as an energy store with a peripheral distribution (buttocks and thighs) unrelated to negative health outcomes, occurs more frequently in the female sex and is referred to as gynoid fat.

Finally, RV should not be seen as static but with variation at the inter- and intrapopulation levels. These variations can be attributed to regional, genetic, dietary and physical activity influences as well as the effects of different exposures over time [45]. In the last 30 years, there has been a significant increase in the mean weight and BMI of children [42–44] and adults [46]. Similarly, in the last 100 years, there have been great variations in the mean height of almost all populations, with large differences among them [4, 46]. This justifies the need to update, adjust and recalculate reference values periodically and thus increase their functionality. The RV presented in this study may be seen as a valid starting point of what we visualize should improve the clinical assessment of BC and foster significant growth in this area of knowledge involving the Mexican children and adolescent population. An early potential exploration lies in the questioning of the BMI cut-off points currently adopted to define OW or OB and whether we should continue to categorize our population based on this single criterion.

## Conclusions

We report valid BC reference data for the urban Mexican pediatric population. There are important differences in the BC of Mexican children and adolescents compared with other populations that justify the need for these RV and that merit further investigation. The measurement of BC provides more clinical information on nutritional status than BMI alone; these RV are different from those reported for other populations, and therefore, should be used for clinical and research purposes involving Mexican children and adolescents.

## Supplementary information

Supplementary Material

## Data Availability

The data that support the results of this study are not publicly available because they contain information that could compromise the privacy of the pediatric participants who participated in the research. For more information, you can contact the corresponding author.
