# Supplementary material for: Reference values for body composition in healthy urban Mexican children and adolescents
Source: Eur J Clin Nutr. 2023 Oct 16;78(11):979–94. doi: 10.1038/s41430-023-01352-1 (PMC11537948; doi:10.1038/s41430-023-01352-1)

Reference values of body composition for healthy urban Mexican children and adolescents. “Online Supplementary Material”

Figure 1. BMI distribution comparison by sex and year of age between our sample (red) with that reported in the 2018 Mexican National Health and Nutrition Survey (ENSANUT 2018) (blue).
By T-student test

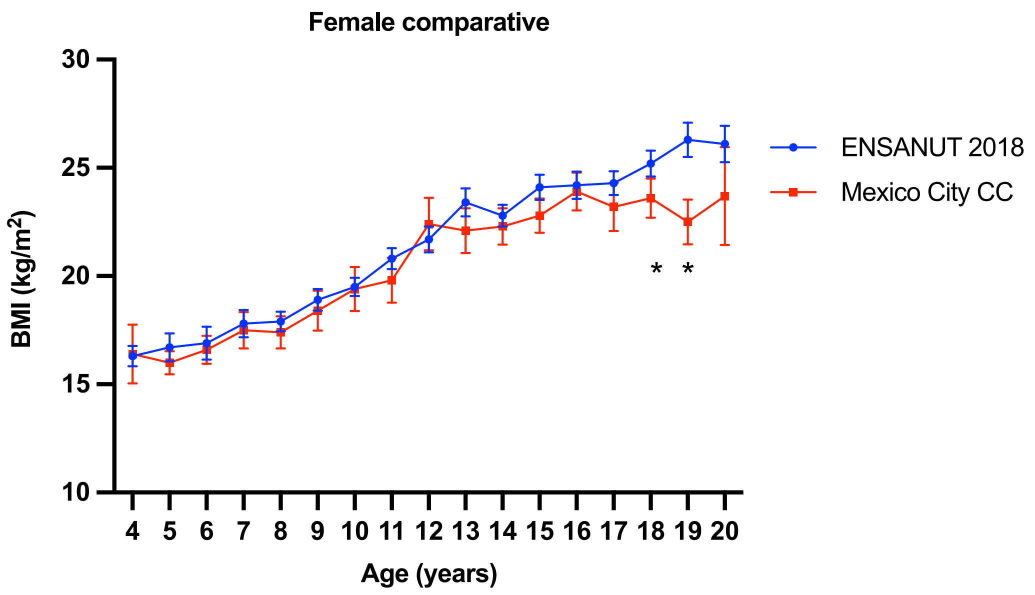

* Age group with mean BMI differences, P value <0.05

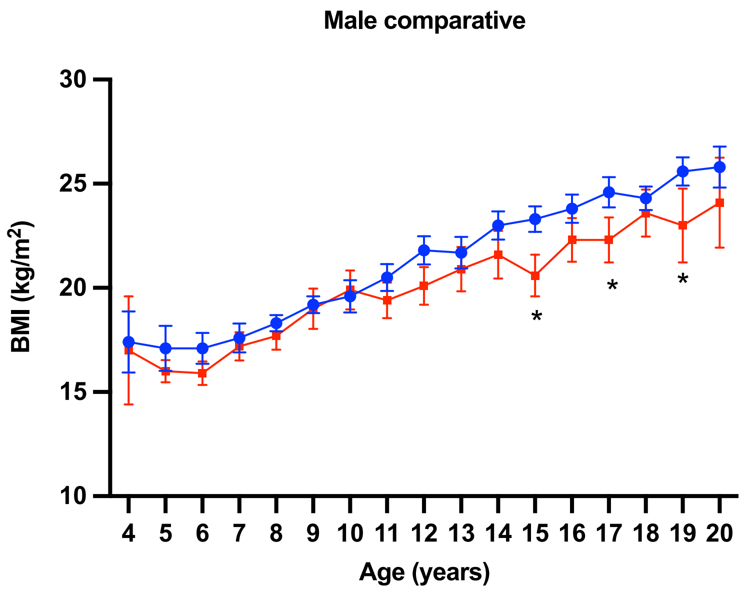

* Age group with mean BMI differences, P value <0.05

Figure 2. Participants flowchart.

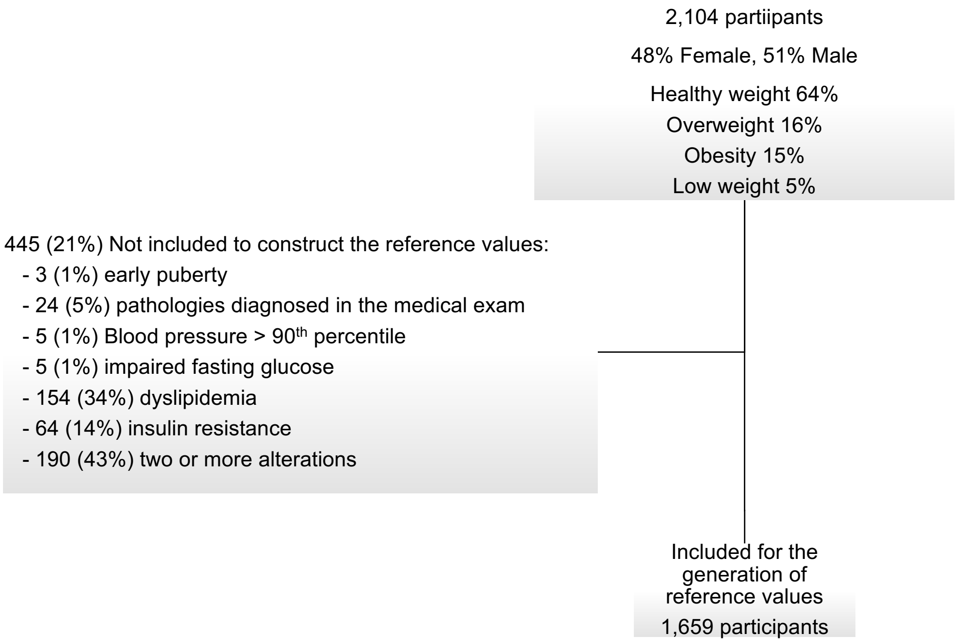

Supplementary table 1. Sample size by age and sex groups.

Age group	Female (n)	Male (n)
4	7	7
5	64	57
6	51	56
7	52	81
8	54	78
9	44	67
10	45	59
11	58	57
12	39	50
13	44	49
14	55	49
15	53	61
16	65	49
17	54	43
18	54	43
19	47	30
20	20	17

Body composition centiles values by age and gender for Mexican subjects (5 to 18 years).

Part 1. Anthropometry: raw values.

Supplementary Table 2. Smoothed percentiles for waist circumference according to WHO methodology, for female and male 5 to 18 years.

Waist circumference (cm) (WHO methodology)

Age (years)	Female LMS parameters and centiles													Male LMS parameters and centiles												
	L	S	1th	3th	5th	15th	25th	50th (M)	75th	85th	95th	97th	99th	L	S	1th	3th	5th	15th	25th	50th (M)	75th	85th	95th	97th	99th
5	-2.976	0.079	43.87	44.92	45.52	47.21	48.35	50.8	53.83	55.79	59.86	61.79	66.25	-2.867	0.059	44.76	45.63	46.12	47.49	48.38	50.22	52.38	53.69	56.23	57.35	59.72
5.5	-2.979	0.082	44.88	45.98	46.6	48.39	49.59	52.2	55.45	57.57	62.02	64.16	69.18	-2.826	0.064	45.73	46.68	47.21	48.71	49.7	51.76	54.18	55.68	58.61	59.92	62.72
6	-2.976	0.085	45.65	46.79	47.45	49.32	50.58	53.34	56.8	59.08	63.91	66.27	71.88	-2.788	0.07	46.52	47.56	48.15	49.8	50.89	53.2	55.96	57.68	61.11	62.66	66.06
6.5	-2.967	0.088	46.16	47.35	48.03	49.98	51.3	54.2	57.87	60.31	65.55	68.14	74.41	-2.753	0.078	47.13	48.27	48.91	50.74	51.96	54.56	57.73	59.75	63.84	65.75	70.01
7	-2.949	0.092	46.5	47.73	48.44	50.47	51.85	54.89	58.79	61.39	67.07	69.91	76.91	-2.721	0.086	47.59	48.83	49.54	51.56	52.92	55.86	59.51	61.89	66.84	69.2	74.68
7.5	-2.921	0.096	46.78	48.05	48.79	50.9	52.34	55.52	59.63	62.4	68.5	71.6	79.34	-2.691	0.095	47.99	49.33	50.1	52.31	53.81	57.1	61.26	64.03	69.96	72.88	79.91
8	-2.881	0.099	47.12	48.43	49.19	51.38	52.87	56.19	60.5	63.42	69.91	73.23	81.63	-2.662	0.103	48.4	49.83	50.66	53.04	54.67	58.28	62.95	66.1	73.05	76.58	85.39
8.5	-2.829	0.101	47.6	48.96	49.75	52.01	53.56	57.01	61.5	64.55	71.36	74.86	83.76	-2.636	0.111	48.82	50.34	51.21	53.75	55.5	59.42	64.56	68.1	76.09	80.26	91.04
9	-2.767	0.103	48.26	49.67	50.48	52.82	54.43	58	62.65	65.81	72.86	76.49	85.7	-2.611	0.117	49.27	50.86	51.78	54.47	56.33	60.51	66.09	69.98	78.95	83.75	96.5
9.5	-2.694	0.104	49.05	50.5	51.34	53.76	55.42	59.11	63.9	67.16	74.38	78.08	87.42	-2.587	0.121	49.82	51.47	52.42	55.22	57.17	61.57	67.48	71.65	81.38	86.66	100.97
10	-2.608	0.105	49.9	51.41	52.27	54.78	56.49	60.29	65.2	68.52	75.84	79.56	88.84	-2.565	0.123	50.49	52.18	53.16	56.04	58.05	62.59	68.71	73.04	83.19	88.73	103.81
10.5	-2.511	0.105	50.8	52.35	53.25	55.83	57.6	61.49	66.5	69.86	77.19	80.87	89.91	-2.544	0.123	51.27	52.99	54	56.93	58.97	63.58	69.8	74.18	84.42	89.98	105.08
11	-2.405	0.105	51.76	53.36	54.29	56.95	58.76	62.74	67.81	71.19	78.47	82.06	90.73	-2.523	0.122	52.12	53.87	54.89	57.85	59.9	64.54	70.76	75.12	85.22	90.66	105.23
11.5	-2.291	0.105	52.8	54.46	55.41	58.15	60	64.06	69.18	72.55	79.71	83.19	91.41	-2.504	0.12	53.02	54.79	55.81	58.78	60.84	65.48	71.64	75.93	85.76	90.98	104.75
12	-2.171	0.104	53.91	55.63	56.61	59.42	61.32	65.43	70.58	73.94	80.94	84.28	92.01	-2.485	0.117	53.95	55.72	56.75	59.73	61.78	66.39	72.46	76.65	86.12	91.08	103.93
12.5	-2.045	0.102	55.04	56.8	57.82	60.7	62.63	66.81	71.96	75.28	82.1	85.3	92.54	-2.468	0.114	54.89	56.67	57.7	60.67	62.72	67.27	73.23	77.29	86.37	91.04	102.91
13	-1.915	0.101	56.11	57.94	58.97	61.92	63.89	68.11	73.26	76.53	83.15	86.21	92.99	-2.451	0.11	55.86	57.63	58.65	61.61	63.64	68.13	73.95	77.88	86.53	90.91	101.8
13.5	-1.782	0.099	57.1	58.97	60.03	63.04	65.04	69.29	74.42	77.64	84.06	86.97	93.34	-2.434	0.106	56.82	58.59	59.61	62.55	64.55	68.97	74.64	78.43	86.65	90.76	100.75
14	-1.649	0.098	57.97	59.89	60.97	64.03	66.06	70.33	75.42	78.59	84.8	87.59	93.57	-2.418	0.103	57.76	59.53	60.54	63.46	65.45	69.79	75.31	78.98	86.81	90.67	99.89
14.5	-1.516	0.096	58.7	60.67	61.77	64.88	66.93	71.21	76.26	79.37	85.38	88.04	93.68	-2.403	0.1	58.67	60.44	61.45	64.35	66.31	70.59	75.99	79.54	87.05	90.7	99.3
15	-1.383	0.095	59.31	61.31	62.44	65.59	67.65	71.94	76.93	79.98	85.8	88.34	93.67	-2.388	0.098	59.53	61.3	62.31	65.19	67.14	71.37	76.67	80.13	87.39	90.87	98.99
15.5	-1.253	0.094	59.8	61.84	62.98	66.17	68.24	72.52	77.46	80.44	86.07	88.51	93.56	-2.374	0.096	60.35	62.11	63.12	66	67.94	72.14	77.37	80.76	87.82	91.18	98.94
16	-1.124	0.092	60.18	62.25	63.41	66.62	68.7	72.97	77.84	80.75	86.21	88.55	93.34	-2.36	0.094	61.11	62.88	63.89	66.77	68.71	72.89	78.07	81.42	88.35	91.63	99.13
16.5	-0.996	0.091	60.45	62.55	63.72	66.96	69.04	73.29	78.09	80.94	86.22	88.45	93.01	-2.347	0.093	61.81	63.59	64.61	67.5	69.44	73.62	78.79	82.12	88.97	92.19	99.54
17	-0.871	0.09	60.63	62.75	63.94	67.18	69.27	73.48	78.21	80.99	86.1	88.25	92.59	-2.334	0.092	62.46	64.25	65.28	68.19	70.14	74.34	79.52	82.85	89.68	92.89	100.17
17.5	-0.747	0.089	60.73	62.87	64.06	67.32	69.4	73.58	78.23	80.94	85.88	87.95	92.08	-2.321	0.092	63.05	64.86	65.9	68.84	70.81	75.05	80.27	83.62	90.49	93.71	101.01
18	-0.626	0.088	60.76	62.92	64.12	67.38	69.45	73.6	78.17	80.81	85.6	87.58	91.53	-2.309	0.093	63.59	65.42	66.47	69.45	71.45	75.74	81.03	84.42	91.38	94.65	102.04

Supplementary Table 3. Smoothed percentiles for waist circumference according to NIH methodology, for female and male 5 to 18 years.

Waist circumference (cm) (NIH methodology)

Age (years)	Female LMS parameters and centiles													Male LMS parameters and centiles												
	L	S	1th	3th	5th	15th	25th	50th (M)	75th	85th	95th	97th	99th	L	S	1th	3th	5th	15th	25th	50th (M)	75th	85th	95th	97th	99th
5	-3.418	0.083	44.85	45.9	46.51	48.25	49.44	52.05	55.41	57.67	62.64	65.16	71.47	-2.778	0.059	45.78	46.35	46.7	47.8	48.66	50.81	53.38	54.7	56.76	57.51	58.89
5.5	-3.23	0.086	45.91	47.04	47.68	49.53	50.8	53.57	57.12	59.5	64.68	67.29	73.75	-2.741	0.066	46.79	47.44	47.83	49.07	50.04	52.48	55.43	56.98	59.44	60.36	62.08
6	-3.058	0.089	46.73	47.92	48.6	50.57	51.9	54.83	58.57	61.07	66.5	69.21	75.87	-2.707	0.073	47.66	48.38	48.82	50.21	51.29	54.05	57.44	59.26	62.22	63.36	65.51
6.5	-2.899	0.092	47.24	48.5	49.22	51.3	52.71	55.82	59.78	62.43	68.16	71.02	78.02	-2.675	0.081	48.37	49.18	49.67	51.22	52.43	55.53	59.44	61.58	65.16	66.57	69.3
7	-2.753	0.097	47.54	48.87	49.64	51.84	53.34	56.64	60.86	63.69	69.82	72.88	80.39	-2.646	0.089	48.98	49.87	50.41	52.13	53.48	56.95	61.41	63.92	68.25	70	73.47
7.5	-2.617	0.102	47.77	49.18	49.99	52.32	53.91	57.42	61.92	64.94	71.51	74.8	82.89	-2.619	0.098	49.52	50.5	51.09	52.98	54.46	58.3	63.35	66.26	71.44	73.59	77.98
8	-2.489	0.106	48.09	49.58	50.43	52.89	54.57	58.28	63.06	66.26	73.24	76.74	85.34	-2.594	0.106	50.05	51.1	51.74	53.79	55.39	59.59	65.22	68.54	74.64	77.25	82.71
8.5	-2.369	0.11	48.6	50.15	51.05	53.64	55.41	59.32	64.33	67.7	75.02	78.67	87.61	-2.57	0.113	50.56	51.69	52.38	54.58	56.3	60.83	67.02	70.76	77.81	80.91	87.62
9	-2.256	0.112	49.28	50.92	51.86	54.57	56.43	60.51	65.75	69.25	76.82	80.58	89.7	-2.548	0.119	51.1	52.3	53.04	55.36	57.2	62.03	68.72	72.85	80.83	84.44	92.46
9.5	-2.149	0.114	50.11	51.83	52.81	55.64	57.58	61.83	67.26	70.87	78.63	82.45	91.61	-2.526	0.124	51.73	52.98	53.75	56.19	58.11	63.19	70.27	74.7	83.44	87.47	96.61
10	-2.048	0.115	51.05	52.84	53.86	56.81	58.82	63.22	68.81	72.51	80.38	84.22	93.31	-2.506	0.126	52.45	53.76	54.56	57.09	59.07	64.3	71.63	76.25	85.45	89.74	99.59
10.5	-1.952	0.116	52.08	53.93	54.99	58.05	60.13	64.66	70.37	74.12	82.02	85.83	94.72	-2.487	0.126	53.27	54.62	55.44	58.03	60.06	65.39	72.83	77.53	86.93	91.32	101.44
11	-1.86	0.115	53.21	55.13	56.23	59.38	61.51	66.14	71.93	75.71	83.55	87.29	95.86	-2.469	0.125	54.14	55.52	56.36	59.01	61.06	66.44	73.91	78.62	88.03	92.41	102.48
11.5	-1.772	0.114	54.47	56.45	57.58	60.81	62.99	67.69	73.52	77.28	85	88.62	96.8	-2.451	0.124	55.05	56.46	57.31	59.99	62.07	67.46	74.9	79.58	88.87	93.18	103.01
12	-1.688	0.112	55.84	57.87	59.03	62.32	64.54	69.29	75.11	78.83	86.37	89.86	97.62	-2.435	0.122	55.98	57.41	58.27	60.99	63.07	68.45	75.82	80.42	89.51	93.7	103.17
12.5	-1.607	0.109	57.24	59.31	60.5	63.85	66.09	70.86	76.66	80.32	87.65	90.99	98.33	-2.419	0.119	56.94	58.39	59.26	61.99	64.08	69.41	76.66	81.16	89.97	93.99	102.98
13	-1.53	0.107	58.6	60.71	61.91	65.31	67.56	72.35	78.1	81.69	88.8	92.01	98.95	-2.403	0.115	57.93	59.39	60.27	63	65.08	70.36	77.44	81.79	90.26	94.08	102.52
13.5	-1.455	0.104	59.86	62.01	63.23	66.66	68.92	73.7	79.39	82.92	89.81	92.89	99.47	-2.389	0.112	58.94	60.41	61.29	64.02	66.09	71.28	78.16	82.36	90.44	94.05	101.92
14	-1.383	0.102	61	63.17	64.4	67.86	70.13	74.9	80.52	83.98	90.67	93.62	99.88	-2.374	0.108	59.96	61.43	62.32	65.04	67.08	72.17	78.86	82.9	90.6	94	101.33
14.5	-1.314	0.1	61.99	64.19	65.43	68.91	71.18	75.93	81.48	84.87	91.37	94.21	100.19	-2.36	0.105	60.95	62.43	63.32	66.03	68.05	73.05	79.54	83.44	90.79	94.01	100.88
15	-1.247	0.098	62.84	65.06	66.31	69.8	72.07	76.79	82.27	85.59	91.91	94.66	100.38	-2.347	0.102	61.91	63.4	64.28	66.99	68.99	73.91	80.23	84	91.07	94.14	100.63
15.5	-1.182	0.096	63.55	65.78	67.04	70.53	72.8	77.49	82.9	86.16	92.31	94.97	100.47	-2.334	0.099	62.83	64.32	65.21	67.91	69.9	74.75	80.93	84.6	91.44	94.38	100.58
16	-1.119	0.094	64.11	66.36	67.62	71.11	73.38	78.04	83.37	86.56	92.56	95.13	100.43	-2.322	0.097	63.7	65.2	66.09	68.79	70.77	75.57	81.65	85.24	91.9	94.76	100.74
16.5	-1.058	0.093	64.54	66.79	68.06	71.54	73.8	78.42	83.68	86.81	92.66	95.16	100.26	-2.31	0.095	64.51	66.03	66.92	69.63	71.61	76.38	82.38	85.92	92.46	95.26	101.11
17	-0.999	0.092	64.84	67.1	68.36	71.84	74.08	78.66	83.84	86.92	92.62	95.04	99.97	-2.298	0.094	65.27	66.8	67.7	70.43	72.41	77.17	83.13	86.64	93.12	95.89	101.66
17.5	-0.942	0.09	65.04	67.3	68.56	72.02	74.25	78.79	83.89	86.9	92.46	94.81	99.58	-2.287	0.093	65.97	67.52	68.44	71.18	73.18	77.94	83.9	87.4	93.87	96.63	102.38
18	-0.886	0.089	65.15	67.41	68.66	72.12	74.33	78.81	83.84	86.79	92.22	94.5	99.12	-2.276	0.093	66.63	68.2	69.12	71.9	73.92	78.7	84.68	88.2	94.7	97.48	103.26

Supplementary Table 4. Smoothed percentiles for hip circumference, for female and male 5 to 18 years.

Hip circumference (cm)

Age (years)	Female LMS parameters and centiles													Male LMS parameters and centiles												
	L	S	1th	3th	5th	15th	25th	50th (M)	75th	85th	95th	97th	99th	L	S	1th	3th	5th	15th	25th	50th (M)	75th	85th	95th	97th	99th
5	-3.385	0.069	50.25	51.29	51.89	53.58	54.71	57.14	60.12	62.03	65.98	67.84	72.11	-2.142	0.061	49.41	50.47	51.06	52.69	53.75	55.9	58.35	59.81	62.56	63.73	66.16
5.5	-3.161	0.071	51.3	52.41	53.04	54.83	56.03	58.58	61.69	63.68	67.74	69.64	73.94	-2.014	0.065	50.56	51.71	52.35	54.14	55.29	57.64	60.33	61.94	64.95	66.25	68.93
6	-2.957	0.072	52.26	53.43	54.1	55.98	57.24	59.9	63.14	65.2	69.36	71.28	75.59	-1.896	0.068	51.61	52.86	53.56	55.5	56.75	59.32	62.26	64.02	67.33	68.76	71.7
6.5	-2.768	0.074	53.16	54.41	55.11	57.1	58.42	61.23	64.63	66.77	71.09	73.07	77.47	-1.788	0.073	52.58	53.94	54.7	56.8	58.16	60.96	64.18	66.11	69.75	71.33	74.58
7	-2.594	0.078	54.04	55.37	56.13	58.26	59.67	62.67	66.3	68.59	73.19	75.3	79.97	-1.688	0.078	53.48	54.95	55.78	58.06	59.54	62.6	66.13	68.25	72.27	74.01	77.63
7.5	-2.432	0.082	54.85	56.28	57.1	59.39	60.92	64.17	68.1	70.59	75.59	77.88	82.97	-1.595	0.083	54.34	55.93	56.82	59.3	60.91	64.25	68.11	70.44	74.88	76.8	80.82
8	-2.28	0.087	55.59	57.13	58.01	60.48	62.13	65.64	69.91	72.6	78.03	80.53	86.06	-1.508	0.087	55.21	56.92	57.88	60.55	62.3	65.92	70.11	72.66	77.5	79.61	84.02
8.5	-2.138	0.091	56.38	58.02	58.96	61.61	63.38	67.14	71.72	74.61	80.43	83.11	89.04	-1.426	0.092	56.12	57.95	58.97	61.84	63.71	67.59	72.11	74.85	80.08	82.36	87.13
9	-2.004	0.095	57.29	59.04	60.04	62.86	64.74	68.75	73.61	76.69	82.86	85.69	91.93	-1.348	0.095	57.08	59.02	60.11	63.15	65.14	69.27	74.08	77	82.58	85.01	90.09
9.5	-1.877	0.098	58.34	60.2	61.26	64.26	66.25	70.5	75.64	78.89	85.37	88.33	94.83	-1.275	0.098	58.1	60.14	61.29	64.49	66.59	70.95	76.01	79.08	84.95	87.5	92.83
10	-1.756	0.1	59.51	61.49	62.61	65.78	67.89	72.37	77.77	81.17	87.93	91	97.7	-1.206	0.1	59.17	61.31	62.51	65.87	68.06	72.6	77.88	81.08	87.17	89.81	95.32
10.5	-1.641	0.102	60.83	62.91	64.08	67.42	69.63	74.32	79.94	83.46	90.42	93.56	100.36	-1.14	0.101	60.3	62.52	63.77	67.25	69.53	74.23	79.68	82.97	89.22	91.92	97.54
11	-1.532	0.102	62.41	64.58	65.81	69.28	71.58	76.42	82.19	85.77	92.79	95.93	102.67	-1.077	0.101	61.47	63.76	65.05	68.65	70.99	75.82	81.39	84.74	91.08	93.82	99.47
11.5	-1.427	0.1	64.35	66.6	67.87	71.44	73.79	78.72	84.53	88.11	95.03	98.09	104.59	-1.017	0.101	62.68	65.04	66.36	70.04	72.43	77.35	83	86.39	92.76	95.49	101.12
12	-1.327	0.097	66.62	68.93	70.23	73.86	76.23	81.17	86.92	90.42	97.12	100.04	106.16	-0.96	0.1	63.94	66.35	67.7	71.44	73.86	78.83	84.51	87.9	94.23	96.93	102.48
12.5	-1.232	0.093	69.05	71.39	72.71	76.35	78.73	83.62	89.24	92.62	99.01	101.76	107.45	-0.905	0.098	65.26	67.71	69.07	72.85	75.28	80.27	85.92	89.27	95.51	98.16	103.56
13	-1.139	0.088	71.43	73.78	75.1	78.74	81.09	85.9	91.36	94.6	100.66	103.24	108.52	-0.852	0.095	66.64	69.1	70.48	74.26	76.69	81.65	87.23	90.52	96.61	99.18	104.39
13.5	-1.051	0.084	73.59	75.95	77.27	80.88	83.2	87.9	93.19	96.31	102.05	104.48	109.39	-0.801	0.092	68.05	70.52	71.89	75.66	78.08	82.97	88.45	91.67	97.57	100.05	105.05
14	-0.965	0.08	75.45	77.81	79.12	82.7	84.99	89.6	94.72	97.72	103.19	105.48	110.09	-0.751	0.089	69.45	71.92	73.28	77.03	79.42	84.24	89.6	92.72	98.43	100.81	105.59
14.5	-0.883	0.077	76.98	79.33	80.64	84.19	86.45	90.98	95.98	98.88	104.14	106.33	110.7	-0.704	0.086	70.82	73.27	74.63	78.35	80.71	85.44	90.67	93.71	99.22	101.51	106.08
15	-0.803	0.075	78.17	80.53	81.83	85.38	87.62	92.1	97	99.84	104.94	107.05	111.25	-0.658	0.083	72.12	74.57	75.92	79.59	81.92	86.57	91.68	94.63	99.96	102.16	106.54
15.5	-0.726	0.074	79.06	81.44	82.75	86.3	88.55	93	97.85	100.64	105.65	107.71	111.79	-0.614	0.081	73.36	75.79	77.13	80.77	83.06	87.64	92.63	95.5	100.67	102.79	107.01
16	-0.651	0.073	79.69	82.1	83.42	87	89.24	93.7	98.54	101.31	106.26	108.29	112.3	-0.571	0.078	74.51	76.93	78.27	81.87	84.14	88.65	93.54	96.34	101.37	103.42	107.49
16.5	-0.579	0.073	80.06	82.5	83.84	87.45	89.72	94.2	99.04	101.8	106.73	108.74	112.71	-0.53	0.076	75.59	78.01	79.33	82.91	85.16	89.61	94.42	97.17	102.07	104.07	108.02
17	-0.509	0.073	80.2	82.67	84.02	87.68	89.97	94.48	99.34	102.11	107.03	109.03	112.98	-0.489	0.075	76.6	79.01	80.33	83.89	86.13	90.53	95.28	97.98	102.78	104.74	108.59
17.5	-0.441	0.074	80.13	82.64	84.02	87.72	90.03	94.59	99.48	102.26	107.18	109.18	113.12	-0.45	0.073	77.55	79.96	81.28	84.83	87.05	91.43	96.12	98.79	103.52	105.44	109.22
18	-0.375	0.075	79.9	82.45	83.85	87.6	89.94	94.55	99.48	102.27	107.21	109.22	113.15	-0.412	0.072	78.44	80.85	82.18	85.73	87.95	92.3	96.96	99.6	104.27	106.17	109.88

Supplementary Table 5. Smoothed percentiles for skinfold thickness right triceps, for female and male 5 to 18 years.

Right triceps skinfold (mm)

Age (years)	Female LMS parameters and centiles													Male LMS parameters and centiles												
	L	S	1th	3th	5th	15th	25th	50th (M)	75th	85th	95th	97th	99th	L	S	1th	3th	5th	15th	25th	50th (M)	75th	85th	95th	97th	99th
5	-0.6	0.214	5.71	6.16	6.42	7.17	7.69	8.84	10.28	11.22	13.13	14.02	15.99	-0.49	0.27	4.78	5.25	5.53	6.36	6.94	8.26	9.99	11.16	13.62	14.8	17.48
5.5	-0.522	0.233	5.78	6.28	6.57	7.42	8.01	9.31	10.97	12.05	14.28	15.32	17.61	-0.479	0.285	4.6	5.08	5.36	6.21	6.81	8.18	10.01	11.25	13.91	15.2	18.17
6	-0.451	0.252	5.8	6.36	6.68	7.63	8.29	9.77	11.66	12.9	15.46	16.65	19.3	-0.47	0.299	4.53	5.02	5.31	6.19	6.82	8.26	10.21	11.54	14.44	15.86	19.16
6.5	-0.385	0.272	5.79	6.4	6.75	7.81	8.55	10.21	12.35	13.76	16.68	18.05	21.09	-0.462	0.311	4.56	5.08	5.38	6.31	6.97	8.52	10.63	12.08	15.28	16.86	20.56
7	-0.324	0.292	5.75	6.41	6.8	7.97	8.78	10.63	13.03	14.62	17.93	19.47	22.91	-0.454	0.322	4.67	5.22	5.54	6.53	7.24	8.91	11.21	12.81	16.36	18.12	22.31
7.5	-0.268	0.311	5.7	6.42	6.84	8.11	9.01	11.04	13.7	15.46	19.13	20.84	24.65	-0.448	0.333	4.82	5.39	5.74	6.8	7.56	9.37	11.87	13.62	17.55	19.53	24.24
8	-0.215	0.326	5.67	6.44	6.89	8.26	9.23	11.44	14.33	16.25	20.22	22.07	26.16	-0.442	0.342	4.96	5.57	5.93	7.05	7.87	9.81	12.51	14.43	18.74	20.92	26.18
8.5	-0.165	0.336	5.67	6.48	6.97	8.43	9.47	11.83	14.91	16.94	21.13	23.08	27.34	-0.437	0.351	5.08	5.71	6.1	7.28	8.15	10.21	13.1	15.17	19.86	22.25	28.03
9	-0.118	0.342	5.71	6.57	7.08	8.63	9.73	12.21	15.43	17.53	21.85	23.83	28.14	-0.433	0.359	5.16	5.82	6.22	7.46	8.36	10.53	13.6	15.8	20.83	23.41	29.7
9.5	-0.073	0.342	5.8	6.71	7.25	8.86	10.01	12.58	15.88	18.02	22.35	24.32	28.57	-0.428	0.367	5.2	5.88	6.3	7.57	8.51	10.77	13.98	16.3	21.63	24.38	31.13
10	-0.031	0.338	5.95	6.89	7.46	9.13	10.31	12.94	16.27	18.41	22.68	24.6	28.7	-0.425	0.374	5.21	5.9	6.33	7.63	8.6	10.92	14.26	16.67	22.26	25.16	32.31
10.5	0.009	0.331	6.13	7.12	7.7	9.43	10.63	13.29	16.62	18.73	22.89	24.75	28.66	-0.421	0.38	5.18	5.88	6.31	7.63	8.61	10.99	14.41	16.91	22.7	25.73	33.22
11	0.047	0.323	6.34	7.36	7.96	9.73	10.95	13.64	16.94	19.02	23.06	24.84	28.56	-0.418	0.386	5.11	5.81	6.24	7.57	8.56	10.96	14.45	16.99	22.95	26.06	33.83
11.5	0.084	0.316	6.55	7.6	8.22	10.03	11.28	13.98	17.26	19.3	23.23	24.95	28.5	-0.415	0.392	5.02	5.71	6.14	7.47	8.46	10.87	14.38	16.96	23.02	26.21	34.18
12	0.118	0.309	6.75	7.84	8.47	10.33	11.59	14.31	17.58	19.59	23.44	25.1	28.52	-0.412	0.397	4.9	5.59	6.01	7.33	8.32	10.73	14.25	16.85	22.97	26.21	34.35
12.5	0.152	0.303	6.94	8.06	8.71	10.61	11.89	14.64	17.9	19.89	23.67	25.29	28.6	-0.41	0.402	4.79	5.47	5.88	7.19	8.17	10.57	14.1	16.7	22.88	26.15	34.42
13	0.184	0.299	7.11	8.27	8.94	10.87	12.18	14.96	18.23	20.21	23.94	25.52	28.76	-0.407	0.407	4.68	5.35	5.76	7.06	8.03	10.42	13.94	16.56	22.78	26.09	34.48
13.5	0.215	0.295	7.27	8.46	9.15	11.13	12.46	15.27	18.56	20.53	24.23	25.79	28.96	-0.405	0.412	4.58	5.25	5.66	6.94	7.91	10.29	13.81	16.44	22.7	26.05	34.57
14	0.245	0.292	7.41	8.64	9.35	11.38	12.73	15.58	18.89	20.87	24.54	26.09	29.21	-0.403	0.416	4.5	5.16	5.57	6.84	7.81	10.18	13.72	16.35	22.68	26.07	34.72
14.5	0.274	0.29	7.54	8.8	9.53	11.61	12.99	15.89	19.22	21.2	24.87	26.4	29.49	-0.401	0.42	4.43	5.09	5.49	6.77	7.73	10.11	13.65	16.31	22.7	26.13	34.93
15	0.302	0.288	7.66	8.96	9.71	11.84	13.25	16.19	19.55	21.54	25.2	26.73	29.8	-0.399	0.424	4.38	5.03	5.43	6.71	7.67	10.05	13.62	16.29	22.76	26.24	35.19
15.5	0.329	0.287	7.76	9.1	9.87	12.06	13.5	16.48	19.88	21.88	25.55	27.08	30.12	-0.397	0.428	4.33	4.99	5.39	6.66	7.62	10.02	13.61	16.31	22.85	26.39	35.51
16	0.355	0.286	7.86	9.24	10.03	12.27	13.74	16.77	20.21	22.23	25.91	27.44	30.47	-0.396	0.431	4.3	4.95	5.35	6.63	7.59	10	13.62	16.35	22.98	26.58	35.87
16.5	0.38	0.285	7.94	9.36	10.17	12.47	13.97	17.06	20.54	22.58	26.28	27.81	30.84	-0.394	0.434	4.27	4.92	5.32	6.6	7.57	9.99	13.64	16.4	23.13	26.79	36.26
17	0.404	0.285	8.01	9.47	10.31	12.66	14.2	17.34	20.87	22.93	26.65	28.18	31.22	-0.393	0.438	4.25	4.9	5.3	6.59	7.56	10	13.68	16.47	23.29	27.01	36.67
17.5	0.428	0.285	8.07	9.58	10.44	12.85	14.42	17.62	21.2	23.28	27.03	28.57	31.61	-0.391	0.441	4.23	4.88	5.29	6.57	7.55	10.01	13.72	16.55	23.46	27.25	37.09
18	0.451	0.286	8.12	9.67	10.55	13.02	14.63	17.9	21.53	23.64	27.42	28.96	32.02	-0.39	0.444	4.21	4.87	5.27	6.56	7.55	10.02	13.77	16.63	23.64	27.49	37.51

Supplementary Table 6. Smoothed percentiles for skinfold thickness right thigh, for female and male 5 to 18 years.

Right thigh skinfold (mm)

Age (years)	Female LMS parameters and centiles													Male LMS parameters and centiles												
	L	S	1th	3th	5th	15th	25th	50th (M)	75th	85th	95th	97th	99th	L	S	1th	3th	5th	15th	25th	50th (M)	75th	85th	95th	97th	99th
5	0.705	0.353	3.69	5.14	5.96	8.25	9.71	12.6	15.71	17.45	20.51	21.73	24.1	0.082	0.283	5.49	6.05	6.38	7.44	8.24	10.19	12.57	13.85	15.99	16.82	18.37
5.5	0.486	0.352	4.67	5.97	6.72	8.88	10.3	13.26	16.6	18.55	22.08	23.54	26.42	0.039	0.304	5.37	5.95	6.3	7.41	8.26	10.37	13	14.45	16.91	17.87	19.71
6	0.309	0.351	5.43	6.65	7.36	9.45	10.87	13.89	17.45	19.61	23.62	25.32	28.76	-0.001	0.325	5.33	5.93	6.3	7.47	8.37	10.66	13.58	15.22	18.06	19.18	21.36
6.5	0.177	0.349	6.03	7.21	7.91	9.97	11.4	14.5	18.27	20.6	25.07	27	30.99	-0.037	0.344	5.39	6.02	6.41	7.66	8.64	11.14	14.41	16.29	19.58	20.91	23.51
7	0.083	0.348	6.52	7.69	8.39	10.46	11.9	15.09	19.04	21.53	26.41	28.55	33.05	-0.071	0.361	5.57	6.25	6.67	8.02	9.08	11.85	15.53	17.7	21.56	23.15	26.28
7.5	0.019	0.347	6.93	8.11	8.81	10.91	12.38	15.66	19.78	22.41	27.63	29.96	34.9	-0.102	0.377	5.84	6.56	7.01	8.47	9.63	12.69	16.84	19.33	23.85	25.72	29.48
8	-0.022	0.346	7.29	8.49	9.2	11.33	12.84	16.21	20.48	23.24	28.75	31.23	36.54	-0.132	0.389	6.11	6.88	7.36	8.93	10.18	13.51	18.13	20.94	26.14	28.32	32.76
8.5	-0.048	0.345	7.61	8.83	9.56	11.74	13.28	16.74	21.16	24.02	29.78	32.39	37.99	-0.159	0.4	6.36	7.17	7.68	9.33	10.66	14.24	19.28	22.4	28.26	30.76	35.88
9	-0.062	0.345	7.9	9.14	9.89	12.12	13.7	17.26	21.81	24.77	30.74	33.45	39.28	-0.185	0.409	6.56	7.4	7.92	9.64	11.04	14.81	20.22	23.61	30.07	32.87	38.68
9.5	-0.071	0.344	8.16	9.44	10.21	12.5	14.12	17.77	22.45	25.49	31.64	34.44	40.48	-0.21	0.416	6.69	7.54	8.08	9.85	11.29	15.2	20.89	24.51	31.52	34.59	41.02
10	-0.079	0.343	8.42	9.73	10.52	12.86	14.52	18.26	23.07	26.19	32.53	35.41	41.64	-0.233	0.422	6.76	7.62	8.16	9.95	11.42	15.43	21.32	25.13	32.58	35.89	42.91
10.5	-0.09	0.342	8.69	10.03	10.83	13.22	14.92	18.75	23.67	26.89	33.41	36.39	42.83	-0.255	0.428	6.75	7.61	8.15	9.95	11.41	15.46	21.48	25.41	33.22	36.73	44.25
11	-0.105	0.342	8.96	10.32	11.14	13.58	15.31	19.22	24.27	27.57	34.3	37.38	44.07	-0.277	0.432	6.67	7.51	8.05	9.81	11.26	15.28	21.34	25.33	33.37	37.03	44.94
11.5	-0.123	0.341	9.24	10.62	11.44	13.93	15.69	19.68	24.86	28.25	35.19	38.39	45.35	-0.297	0.436	6.5	7.33	7.84	9.56	10.98	14.92	20.91	24.9	33.05	36.8	44.99
12	-0.139	0.34	9.5	10.91	11.75	14.27	16.07	20.14	25.43	28.91	36.07	39.37	46.59	-0.316	0.439	6.29	7.08	7.57	9.23	10.6	14.41	20.27	24.22	32.36	36.14	44.52
12.5	-0.152	0.34	9.76	11.19	12.04	14.61	16.43	20.58	25.99	29.56	36.91	40.31	47.77	-0.335	0.441	6.05	6.81	7.28	8.87	10.18	13.85	19.53	23.4	31.46	35.25	43.72
13	-0.159	0.339	10	11.45	12.32	14.93	16.79	21.02	26.54	30.18	37.7	41.18	48.83	-0.353	0.443	5.82	6.54	7	8.52	9.77	13.3	18.79	22.56	30.51	34.29	42.81
13.5	-0.159	0.339	10.21	11.7	12.58	15.25	17.14	21.45	27.07	30.78	38.43	41.98	49.77	-0.37	0.444	5.63	6.32	6.75	8.21	9.41	12.8	18.12	21.8	29.64	33.4	41.96
14	-0.153	0.338	10.41	11.92	12.83	15.55	17.48	21.87	27.59	31.36	39.11	42.7	50.56	-0.386	0.444	5.47	6.13	6.55	7.95	9.11	12.39	17.56	21.16	28.9	32.65	41.26
14.5	-0.141	0.338	10.58	12.13	13.06	15.84	17.81	22.29	28.09	31.91	39.73	43.34	51.22	-0.402	0.444	5.35	5.99	6.4	7.76	8.88	12.06	17.11	20.64	28.32	32.06	40.74
15	-0.121	0.337	10.73	12.32	13.27	16.12	18.14	22.7	28.58	32.43	40.29	43.89	51.73	-0.418	0.444	5.26	5.88	6.28	7.6	8.7	11.8	16.75	20.23	27.86	31.62	40.39
15.5	-0.094	0.337	10.85	12.49	13.47	16.39	18.45	23.1	29.06	32.93	40.79	44.37	52.1	-0.433	0.443	5.19	5.81	6.19	7.49	8.56	11.6	16.46	19.91	27.52	31.29	40.19
16	-0.06	0.336	10.94	12.63	13.64	16.64	18.76	23.5	29.52	33.41	41.22	44.75	52.32	-0.447	0.443	5.14	5.75	6.13	7.4	8.45	11.43	16.23	19.65	27.25	31.05	40.08
16.5	-0.017	0.336	11	12.75	13.79	16.89	19.06	23.89	29.97	33.86	41.6	45.06	52.42	-0.461	0.442	5.11	5.7	6.07	7.32	8.36	11.3	16.04	19.44	27.04	30.87	40.06
17	0.033	0.335	11.02	12.84	13.91	17.11	19.34	24.27	30.4	34.29	41.92	45.3	52.42	-0.475	0.441	5.08	5.67	6.04	7.27	8.29	11.19	15.88	19.26	26.89	30.75	40.1
17.5	0.089	0.335	11	12.9	14.02	17.33	19.62	24.65	30.83	34.69	42.2	45.49	52.34	-0.488	0.44	5.07	5.65	6.01	7.23	8.23	11.1	15.76	19.12	26.77	30.68	40.2
18	0.15	0.334	10.95	12.93	14.1	17.53	19.9	25.03	31.24	35.09	42.46	45.65	52.24	-0.501	0.439	5.06	5.63	5.99	7.2	8.19	11.03	15.65	19.01	26.69	30.64	40.34

Supplementary Table 7. Smoothed percentiles for skinfold thickness right calf, for female and male 5 to 18 years.

Right calf skinfold (mm)

Age (years)	Female LMS parameters and centiles													Male LMS parameters and centiles												
	L	S	1th	3th	5th	15th	25th	50th (M)	75th	85th	95th	97th	99th	L	S	1th	3th	5th	15th	25th	50th (M)	75th	85th	95th	97th	99th
5	-0.577	0.217	5.35	5.77	6.02	6.74	7.23	8.32	9.69	10.58	12.4	13.24	15.1	-0.271	0.209	4.67	5.05	5.27	5.9	6.34	7.31	8.47	9.17	10.47	11.02	12.16
5.5	-0.494	0.256	4.97	5.44	5.72	6.53	7.1	8.39	10.05	11.15	13.46	14.55	17.01	-0.271	0.242	4.45	4.85	5.09	5.78	6.28	7.41	8.81	9.66	11.26	11.95	13.37
6	-0.422	0.291	4.67	5.18	5.48	6.38	7.02	8.48	10.41	11.72	14.49	15.82	18.85	-0.271	0.275	4.27	4.69	4.94	5.7	6.25	7.56	9.23	10.26	12.21	13.06	14.83
6.5	-0.36	0.322	4.42	4.96	5.29	6.27	6.97	8.59	10.77	12.26	15.47	17.02	20.59	-0.27	0.309	4.14	4.58	4.85	5.67	6.28	7.78	9.77	11.01	13.4	14.45	16.66
7	-0.305	0.352	4.21	4.78	5.13	6.18	6.95	8.74	11.18	12.87	16.53	18.32	22.44	-0.27	0.344	4.05	4.51	4.79	5.68	6.36	8.08	10.43	11.92	14.84	16.14	18.9
7.5	-0.257	0.379	4.05	4.66	5.03	6.17	7	8.96	11.68	13.56	17.67	19.69	24.34	-0.27	0.377	3.98	4.47	4.76	5.72	6.47	8.41	11.15	12.92	16.45	18.03	21.44
8	-0.214	0.397	3.99	4.64	5.03	6.25	7.15	9.28	12.22	14.27	18.75	20.93	25.96	-0.27	0.406	3.95	4.45	4.76	5.78	6.59	8.75	11.9	13.96	18.13	20.02	24.14
8.5	-0.175	0.406	4.03	4.72	5.14	6.44	7.4	9.67	12.8	14.97	19.67	21.95	27.16	-0.269	0.431	3.95	4.47	4.79	5.87	6.73	9.11	12.65	15.01	19.81	22.01	26.82
9	-0.14	0.408	4.16	4.89	5.34	6.72	7.74	10.14	13.42	15.67	20.49	22.81	28.03	-0.269	0.45	3.98	4.52	4.86	5.98	6.9	9.47	13.39	16.01	21.41	23.9	29.37
9.5	-0.108	0.404	4.36	5.14	5.61	7.08	8.15	10.66	14.06	16.37	21.25	23.56	28.73	-0.269	0.465	4.04	4.58	4.93	6.1	7.07	9.82	14.06	16.93	22.85	25.6	31.65
10	-0.079	0.398	4.6	5.43	5.93	7.48	8.61	11.23	14.72	17.07	21.98	24.27	29.34	-0.269	0.476	4.09	4.65	5.01	6.22	7.23	10.12	14.64	17.71	24.08	27.04	33.58
10.5	-0.052	0.39	4.86	5.74	6.27	7.91	9.09	11.8	15.39	17.77	22.68	24.96	29.93	-0.269	0.484	4.14	4.71	5.08	6.31	7.36	10.36	15.1	18.33	25.04	28.17	35.09
11	-0.027	0.383	5.12	6.06	6.62	8.33	9.56	12.37	16.04	18.45	23.38	25.64	30.53	-0.269	0.49	4.17	4.75	5.12	6.38	7.44	10.52	15.41	18.76	25.72	28.97	36.17
11.5	-0.004	0.377	5.39	6.37	6.96	8.75	10.03	12.94	16.69	19.13	24.08	26.33	31.17	-0.268	0.494	4.18	4.76	5.13	6.4	7.48	10.6	15.59	19.01	26.13	29.45	36.83
12	0.017	0.372	5.64	6.67	7.29	9.16	10.49	13.49	17.33	19.81	24.8	27.05	31.86	-0.268	0.496	4.16	4.74	5.12	6.39	7.47	10.61	15.63	19.08	26.29	29.66	37.13
12.5	0.037	0.368	5.87	6.95	7.6	9.54	10.92	14.02	17.95	20.48	25.52	27.78	32.58	-0.268	0.498	4.13	4.71	5.08	6.35	7.42	10.55	15.57	19.03	26.24	29.62	37.13
13	0.055	0.365	6.08	7.2	7.88	9.9	11.33	14.51	18.54	21.11	26.22	28.49	33.31	-0.268	0.499	4.08	4.66	5.03	6.28	7.35	10.46	15.44	18.86	26.03	29.4	36.88
13.5	0.072	0.364	6.25	7.42	8.12	10.22	11.69	14.97	19.09	21.71	26.89	29.19	34.03	-0.268	0.498	4.03	4.6	4.97	6.21	7.26	10.33	15.24	18.62	25.7	29.03	36.43
14	0.088	0.363	6.39	7.6	8.32	10.49	12	15.38	19.59	22.26	27.52	29.84	34.72	-0.268	0.497	3.98	4.54	4.9	6.13	7.17	10.18	15	18.32	25.27	28.54	35.82
14.5	0.104	0.363	6.49	7.74	8.49	10.71	12.27	15.73	20.04	22.76	28.1	30.45	35.38	-0.268	0.495	3.93	4.48	4.84	6.05	7.07	10.02	14.73	17.97	24.76	27.96	35.08
15	0.118	0.365	6.56	7.84	8.61	10.89	12.49	16.04	20.44	23.21	28.63	31.01	35.99	-0.267	0.492	3.88	4.43	4.78	5.97	6.97	9.85	14.44	17.59	24.2	27.3	34.23
15.5	0.132	0.366	6.6	7.91	8.7	11.04	12.67	16.29	20.78	23.61	29.1	31.52	36.56	-0.267	0.488	3.83	4.37	4.72	5.89	6.87	9.68	14.12	17.18	23.58	26.58	33.29
16	0.144	0.369	6.61	7.95	8.75	11.14	12.82	16.51	21.08	23.96	29.54	31.98	37.08	-0.267	0.482	3.79	4.32	4.66	5.81	6.77	9.51	13.81	16.75	22.92	25.81	32.27
16.5	0.157	0.372	6.59	7.96	8.78	11.22	12.93	16.7	21.35	24.27	29.93	32.41	37.56	-0.267	0.477	3.75	4.28	4.62	5.74	6.68	9.33	13.48	16.32	22.24	25.02	31.2
17	0.168	0.375	6.57	7.96	8.79	11.28	13.01	16.85	21.58	24.54	30.28	32.79	38	-0.267	0.47	3.72	4.24	4.57	5.68	6.59	9.17	13.16	15.89	21.56	24.22	30.12
17.5	0.179	0.378	6.52	7.94	8.79	11.31	13.08	16.98	21.79	24.79	30.61	33.15	38.41	-0.267	0.464	3.69	4.2	4.53	5.61	6.5	9	12.85	15.47	20.9	23.44	29.08
18	0.19	0.381	6.47	7.91	8.77	11.34	13.13	17.09	21.97	25.02	30.91	33.48	38.8	-0.267	0.457	3.66	4.17	4.49	5.55	6.42	8.85	12.56	15.07	20.28	22.7	28.08

Supplementary Table 8. Smoothed percentiles for sum of skinfold thickness (mean triceps + mean calf), for female and male 5 to 18 years.

Sum of skinfolds (triceps + calf) (mm)

Age (years)	Female LMS parameters and centiles													Male LMS parameters and centiles												
	L	S	1th	3th	5th	15th	25th	50th (M)	75th	85th	95th	97th	99th	L	S	1th	3th	5th	15th	25th	50th (M)	75th	85th	95th	97th	99th
5	-0.897	0.204	11.92	12.73	13.21	14.59	15.56	17.72	20.53	22.41	26.44	28.4	32.95	-0.443	0.221	9.51	10.17	10.56	11.76	12.64	14.76	17.43	19	21.86	23.05	25.46
5.5	-0.765	0.227	11.63	12.53	13.05	14.6	15.68	18.11	21.31	23.46	28.09	30.33	35.56	-0.432	0.245	9.47	10.18	10.61	11.93	12.91	15.32	18.43	20.29	23.73	25.18	28.16
6	-0.652	0.247	11.37	12.35	12.92	14.62	15.81	18.52	22.08	24.49	29.65	32.16	37.97	-0.421	0.268	9.44	10.21	10.67	12.12	13.21	15.93	19.52	21.7	25.8	27.55	31.19
6.5	-0.554	0.27	11.03	12.08	12.71	14.57	15.89	18.9	22.89	25.6	31.44	34.28	40.85	-0.412	0.291	9.45	10.28	10.78	12.36	13.56	16.62	20.74	23.28	28.14	30.25	34.68
7	-0.468	0.299	10.58	11.73	12.41	14.46	15.93	19.31	23.85	26.97	33.73	37.03	44.71	-0.403	0.313	9.5	10.38	10.92	12.63	13.96	17.37	22.08	25.03	30.76	33.29	38.64
7.5	-0.391	0.329	10.14	11.38	12.12	14.38	16	19.79	24.95	28.52	36.32	40.15	49.08	-0.395	0.335	9.54	10.48	11.06	12.91	14.35	18.13	23.46	26.85	33.54	36.53	42.94
8	-0.323	0.353	9.84	11.17	11.97	14.42	16.2	20.37	26.1	30.06	38.73	42.98	52.86	-0.388	0.356	9.58	10.57	11.19	13.16	14.72	18.86	24.81	28.66	36.35	39.83	47.37
8.5	-0.263	0.367	9.75	11.17	12.03	14.66	16.57	21.06	27.2	31.44	40.63	45.09	55.36	-0.381	0.374	9.62	10.65	11.3	13.39	15.06	19.54	26.09	30.39	39.08	43.06	51.76
9	-0.207	0.371	9.88	11.39	12.3	15.1	17.13	21.86	28.27	32.65	42	46.48	56.62	-0.374	0.391	9.64	10.72	11.39	13.59	15.36	20.15	27.26	31.98	41.62	46.07	55.9
9.5	-0.157	0.367	10.22	11.82	12.79	15.74	17.86	22.77	29.31	33.7	42.93	47.28	56.95	-0.368	0.404	9.66	10.77	11.46	13.75	15.6	20.66	28.27	33.36	43.85	48.74	59.58
10	-0.112	0.357	10.72	12.42	13.44	16.52	18.72	23.74	30.31	34.65	43.6	47.74	56.8	-0.362	0.415	9.66	10.79	11.51	13.86	15.78	21.06	29.08	34.48	45.7	50.95	62.65
10.5	-0.07	0.345	11.33	13.11	14.18	17.38	19.64	24.75	31.3	35.56	44.18	48.1	56.56	-0.356	0.424	9.63	10.79	11.52	13.92	15.89	21.34	29.68	35.33	47.1	52.63	64.99
11	-0.031	0.333	11.97	13.85	14.96	18.27	20.59	25.76	32.28	36.46	44.79	48.52	56.48	-0.35	0.431	9.58	10.75	11.48	13.92	15.92	21.49	30.05	35.87	48.03	53.75	66.56
11.5	0.005	0.323	12.62	14.58	15.74	19.16	21.54	26.79	33.3	37.42	45.53	49.12	56.7	-0.345	0.436	9.5	10.67	11.41	13.86	15.88	21.51	30.21	36.13	48.51	54.35	67.42
12	0.038	0.314	13.24	15.29	16.49	20.03	22.47	27.81	34.35	38.44	46.41	49.91	57.22	-0.34	0.44	9.4	10.56	11.3	13.75	15.77	21.43	30.19	36.15	48.62	54.5	67.65
12.5	0.069	0.308	13.82	15.95	17.2	20.86	23.37	28.81	35.41	39.5	47.4	50.84	57.98	-0.335	0.442	9.28	10.44	11.17	13.61	15.63	21.28	30.03	35.98	48.43	54.29	67.39
13	0.097	0.303	14.33	16.55	17.85	21.63	24.21	29.77	36.45	40.58	48.46	51.87	58.91	-0.331	0.443	9.17	10.32	11.05	13.47	15.46	21.08	29.78	35.69	48.02	53.81	66.74
13.5	0.124	0.3	14.76	17.07	18.42	22.33	24.98	30.67	37.46	41.63	49.54	52.95	59.95	-0.326	0.444	9.07	10.21	10.92	13.32	15.3	20.87	29.48	35.32	47.47	53.16	65.82
14	0.15	0.299	15.11	17.51	18.9	22.93	25.66	31.49	38.41	42.63	50.61	54.03	61.03	-0.322	0.443	8.98	10.1	10.81	13.19	15.15	20.66	29.16	34.91	46.83	52.39	64.74
14.5	0.173	0.299	15.38	17.86	19.3	23.45	26.26	32.24	39.29	43.58	51.65	55.09	62.11	-0.318	0.441	8.9	10.01	10.72	13.07	15	20.45	28.83	34.47	46.14	51.56	63.56
15	0.196	0.299	15.57	18.12	19.61	23.89	26.77	32.89	40.09	44.45	52.62	56.1	63.17	-0.314	0.439	8.84	9.94	10.63	12.96	14.87	20.25	28.5	34.03	45.42	50.7	62.33
15.5	0.217	0.301	15.68	18.31	19.84	24.24	27.2	33.47	40.81	45.24	53.53	57.04	64.17	-0.31	0.437	8.78	9.87	10.56	12.86	14.75	20.06	28.17	33.6	44.71	49.83	61.08
16	0.237	0.302	15.73	18.44	20.01	24.53	27.56	33.97	41.46	45.96	54.37	57.93	65.13	-0.306	0.434	8.74	9.82	10.5	12.78	14.65	19.88	27.86	33.17	43.99	48.97	59.84
16.5	0.256	0.305	15.73	18.51	20.11	24.75	27.85	34.4	42.04	46.62	55.15	58.75	66.03	-0.303	0.431	8.7	9.78	10.45	12.7	14.55	19.72	27.56	32.76	43.3	48.12	58.63
17	0.274	0.308	15.68	18.53	20.17	24.92	28.09	34.78	42.57	47.23	55.88	59.53	66.88	-0.299	0.428	8.67	9.74	10.41	12.63	14.46	19.56	27.27	32.36	42.63	47.31	57.47
17.5	0.291	0.311	15.6	18.51	20.19	25.04	28.29	35.12	43.05	47.79	56.57	60.26	67.7	-0.296	0.425	8.65	9.7	10.37	12.57	14.38	19.42	27	31.98	42	46.54	56.37
18	0.307	0.314	15.49	18.46	20.18	25.14	28.46	35.42	43.49	48.31	57.22	60.96	68.48	-0.293	0.422	8.62	9.67	10.33	12.52	14.31	19.28	26.74	31.63	41.41	45.82	55.34

Supplementary Table 9. Smoothed percentiles for mid-upper right arm circumference (MUAC), for female and male 5 to 18 years.

Right mid-upper arm circumference (cm)

Age (years)	Female LMS parameters and centiles													Male LMS parameters and centiles													
	L	S	1th	3th	5th	15th	25th	50th (M)	75th	85th	95th	97th	99th	L	S	1th	3th	5th	15th	25th	50th (M)	75th	85th	95th	97th	99th
5	-2.651	0.082	14.19	14.55	14.76	15.34	15.73	16.57	17.6	18.26	19.6	20.23	21.66	-2.043	0.067	13.88	14.21	14.39	14.89	15.22	15.88	16.65	17.11	17.99	18.36	19.15
5.5	-2.5	0.087	14.54	14.93	15.15	15.79	16.21	17.12	18.24	18.96	20.44	21.13	22.69	-1.867	0.076	14.11	14.49	14.7	15.28	15.66	16.46	17.37	17.93	18.99	19.45	20.42
6	-2.362	0.091	14.81	15.23	15.47	16.15	16.61	17.59	18.8	19.58	21.18	21.93	23.64	-1.707	0.086	14.32	14.74	14.98	15.65	16.08	17	18.06	18.72	19.97	20.53	21.69
6.5	-2.236	0.096	14.98	15.43	15.68	16.41	16.91	17.96	19.27	20.11	21.85	22.67	24.52	-1.559	0.094	14.5	14.98	15.24	15.99	16.48	17.51	18.73	19.47	20.92	21.55	22.9
7	-2.118	0.102	15.09	15.57	15.84	16.62	17.15	18.29	19.69	20.6	22.49	23.37	25.4	-1.422	0.102	14.67	15.19	15.49	16.32	16.86	18.01	19.37	20.2	21.83	22.55	24.08
7.5	-2.009	0.107	15.19	15.69	15.98	16.82	17.38	18.59	20.1	21.08	23.12	24.08	26.29	-1.295	0.11	14.82	15.39	15.71	16.62	17.22	18.49	19.99	20.92	22.73	23.54	25.25
8	-1.907	0.112	15.29	15.83	16.14	17.02	17.61	18.91	20.52	21.57	23.75	24.78	27.15	-1.176	0.118	14.95	15.57	15.93	16.92	17.58	18.96	20.61	21.63	23.62	24.5	26.39
8.5	-1.811	0.117	15.45	16.01	16.34	17.27	17.9	19.26	20.97	22.08	24.39	25.48	27.99	-1.064	0.125	15.08	15.75	16.13	17.21	17.92	19.42	21.21	22.32	24.49	25.45	27.49
9	-1.721	0.12	15.66	16.25	16.59	17.57	18.24	19.67	21.47	22.63	25.05	26.19	28.8	-0.958	0.131	15.21	15.93	16.34	17.49	18.26	19.88	21.81	23	25.32	26.35	28.53
9.5	-1.635	0.123	15.92	16.54	16.9	17.93	18.62	20.13	22	23.22	25.73	26.91	29.59	-0.858	0.137	15.35	16.12	16.56	17.79	18.61	20.33	22.38	23.65	26.1	27.18	29.47
10	-1.554	0.125	16.22	16.87	17.24	18.32	19.04	20.61	22.55	23.81	26.39	27.6	30.33	-0.764	0.14	15.53	16.34	16.8	18.11	18.97	20.78	22.93	24.25	26.79	27.91	30.26
10.5	-1.477	0.126	16.54	17.22	17.61	18.73	19.48	21.1	23.11	24.4	27.03	28.25	30.98	-0.674	0.143	15.74	16.59	17.08	18.44	19.34	21.23	23.45	24.81	27.41	28.54	30.9
11	-1.404	0.126	16.91	17.62	18.02	19.18	19.95	21.62	23.68	24.99	27.64	28.86	31.57	-0.588	0.143	15.99	16.88	17.38	18.8	19.73	21.68	23.95	25.32	27.94	29.07	31.42
11.5	-1.333	0.126	17.33	18.06	18.48	19.67	20.47	22.18	24.26	25.59	28.24	29.45	32.11	-0.506	0.143	16.27	17.2	17.72	19.18	20.14	22.12	24.42	25.8	28.4	29.52	31.82
12	-1.266	0.124	17.79	18.55	18.98	20.2	21.02	22.76	24.87	26.2	28.83	30.03	32.62	-0.427	0.141	16.6	17.55	18.08	19.58	20.55	22.56	24.86	26.24	28.81	29.9	32.13
12.5	-1.201	0.122	18.28	19.06	19.5	20.76	21.59	23.35	25.47	26.8	29.4	30.57	33.08	-0.352	0.138	16.96	17.94	18.48	20	20.99	23	25.29	26.65	29.16	30.22	32.37
13	-1.139	0.12	18.77	19.57	20.02	21.29	22.14	23.92	26.04	27.35	29.91	31.05	33.47	-0.279	0.135	17.36	18.36	18.91	20.44	21.43	23.44	25.7	27.03	29.47	30.49	32.54
13.5	-1.08	0.118	19.23	20.04	20.5	21.79	22.64	24.44	26.55	27.85	30.36	31.47	33.8	-0.21	0.13	17.8	18.8	19.36	20.9	21.89	23.89	26.1	27.4	29.75	30.73	32.68
14	-1.022	0.115	19.64	20.46	20.93	22.24	23.1	24.89	26.99	28.28	30.74	31.81	34.06	-0.143	0.126	18.25	19.26	19.83	21.37	22.35	24.33	26.5	27.76	30.03	30.97	32.83
14.5	-0.967	0.113	20	20.84	21.31	22.63	23.49	25.29	27.37	28.64	31.04	32.09	34.26	-0.078	0.122	18.7	19.72	20.29	21.83	22.81	24.76	26.9	28.12	30.32	31.22	33
15	-0.913	0.111	20.32	21.16	21.64	22.97	23.83	25.62	27.69	28.94	31.29	32.31	34.4	-0.015	0.118	19.14	20.17	20.74	22.29	23.26	25.2	27.3	28.49	30.63	31.5	33.21
15.5	-0.861	0.109	20.58	21.44	21.91	23.25	24.11	25.9	27.95	29.17	31.48	32.47	34.5	0.045	0.115	19.57	20.61	21.18	22.73	23.71	25.63	27.7	28.88	30.96	31.81	33.46
16	-0.811	0.108	20.8	21.66	22.14	23.47	24.34	26.12	28.15	29.35	31.61	32.57	34.54	0.104	0.113	19.97	21.03	21.6	23.17	24.14	26.06	28.11	29.27	31.32	32.15	33.76
16.5	-0.762	0.106	20.96	21.83	22.31	23.65	24.51	26.28	28.28	29.47	31.68	32.61	34.52	0.161	0.111	20.35	21.42	22.01	23.58	24.56	26.48	28.53	29.68	31.7	32.51	34.1
17	-0.715	0.104	21.08	21.95	22.43	23.76	24.62	26.37	28.35	29.52	31.68	32.59	34.44	0.216	0.11	20.7	21.79	22.39	23.98	24.97	26.9	28.95	30.09	32.1	32.91	34.47
17.5	-0.669	0.103	21.16	22.02	22.5	23.83	24.68	26.42	28.37	29.51	31.62	32.51	34.3	0.27	0.109	21.03	22.14	22.75	24.37	25.37	27.32	29.37	30.52	32.52	33.32	34.88
18	-0.625	0.102	21.2	22.06	22.54	23.86	24.71	26.42	28.34	29.46	31.52	32.39	34.12	0.322	0.108	21.34	22.47	23.09	24.74	25.76	27.73	29.79	30.95	32.95	33.75	35.3

Supplementary Table 10. Smoothed percentiles for girth right thigh, for female and male 5 to 18 years.

Right thigh circumference (cm)

Age (years)	Female LMS parameters and centiles													Male LMS parameters and centiles													
	L	S	1th	3th	5th	15th	25th	50th (M)	75th	85th	95th	97th	99th	L	S	1th	3th	5th	15th	25th	50th (M)	75th	85th	95th	97th	99th
5	-2.572	0.089	26.49	27.21	27.62	28.79	29.58	31.28	33.39	34.76	37.6	38.95	42.06	-0.952	0.08	25.36	26.15	26.59	27.79	28.56	30.1	31.82	32.82	34.65	35.42	36.95
5.5	-2.353	0.088	27.43	28.19	28.62	29.84	30.66	32.4	34.53	35.89	38.64	39.92	42.78	-0.826	0.084	26.11	26.97	27.45	28.77	29.61	31.3	33.17	34.26	36.25	37.07	38.74
6	-2.153	0.087	28.27	29.06	29.51	30.79	31.64	33.43	35.59	36.95	39.66	40.89	43.59	-0.71	0.088	26.77	27.72	28.25	29.68	30.59	32.43	34.46	35.64	37.79	38.69	40.48
6.5	-1.97	0.088	28.94	29.78	30.25	31.59	32.48	34.35	36.57	37.96	40.69	41.92	44.58	-0.604	0.093	27.36	28.39	28.96	30.52	31.51	33.51	35.71	36.99	39.32	40.28	42.21
7	-1.8	0.09	29.44	30.34	30.85	32.27	33.21	35.19	37.54	38.99	41.83	43.1	45.83	-0.505	0.098	27.86	28.98	29.6	31.3	32.37	34.54	36.93	38.32	40.83	41.87	43.95
7.5	-1.641	0.095	29.83	30.79	31.34	32.87	33.88	36	38.51	40.06	43.09	44.44	47.31	-0.414	0.103	28.29	29.5	30.17	32.01	33.18	35.53	38.12	39.62	42.33	43.46	45.7
8	-1.493	0.1	30.14	31.18	31.76	33.41	34.5	36.79	39.49	41.16	44.41	45.85	48.93	-0.328	0.108	28.67	29.98	30.71	32.69	33.95	36.48	39.26	40.87	43.78	44.99	47.38
8.5	-1.354	0.105	30.44	31.55	32.19	33.96	35.14	37.6	40.51	42.3	45.78	47.33	50.6	-0.248	0.112	29.05	30.45	31.23	33.35	34.7	37.4	40.36	42.07	45.16	46.44	48.96
9	-1.223	0.11	30.77	31.97	32.65	34.56	35.82	38.46	41.58	43.49	47.21	48.84	52.31	-0.172	0.115	29.44	30.93	31.76	34.01	35.43	38.28	41.41	43.21	46.44	47.77	50.41
9.5	-1.099	0.115	31.15	32.44	33.17	35.21	36.55	39.38	42.69	44.73	48.66	50.38	54.02	-0.1	0.118	29.88	31.44	32.31	34.67	36.17	39.14	42.39	44.26	47.6	48.97	51.67
10	-0.981	0.119	31.58	32.95	33.73	35.9	37.33	40.33	43.84	45.98	50.09	51.89	55.66	-0.032	0.119	30.37	32	32.91	35.36	36.9	39.98	43.31	45.22	48.63	50.02	52.76
10.5	-0.869	0.122	32.06	33.51	34.33	36.64	38.15	41.3	44.97	47.2	51.46	53.31	57.17	0.033	0.119	30.91	32.6	33.53	36.06	37.65	40.79	44.18	46.11	49.54	50.94	53.68
11	-0.762	0.123	32.66	34.19	35.06	37.47	39.05	42.33	46.13	48.43	52.77	54.65	58.52	0.095	0.118	31.5	33.24	34.19	36.77	38.39	41.57	44.99	46.93	50.37	51.76	54.48
11.5	-0.66	0.123	33.43	35.04	35.94	38.45	40.09	43.47	47.35	49.67	54.03	55.9	59.73	0.154	0.116	32.12	33.89	34.87	37.49	39.13	42.34	45.77	47.71	51.12	52.5	55.18
12	-0.563	0.121	34.39	36.05	36.99	39.57	41.25	44.69	48.59	50.91	55.22	57.05	60.77	0.21	0.114	32.76	34.57	35.56	38.21	39.86	43.08	46.51	48.44	51.81	53.17	55.82
12.5	-0.469	0.118	35.46	37.17	38.13	40.77	42.47	45.93	49.81	52.1	56.31	58.08	61.65	0.264	0.112	33.43	35.26	36.26	38.93	40.59	43.81	47.22	49.13	52.46	53.79	56.39
13	-0.379	0.114	36.57	38.32	39.29	41.96	43.67	47.12	50.95	53.19	57.27	58.97	62.37	0.316	0.11	34.13	35.97	36.98	39.66	41.31	44.52	47.9	49.78	53.05	54.36	56.89
13.5	-0.292	0.11	37.63	39.4	40.38	43.07	44.78	48.2	51.97	54.15	58.09	59.72	62.96	0.367	0.107	34.84	36.7	37.7	40.38	42.03	45.22	48.55	50.4	53.61	54.89	57.36
14	-0.209	0.107	38.58	40.38	41.37	44.06	45.77	49.16	52.86	54.99	58.8	60.36	63.45	0.415	0.104	35.56	37.42	38.43	41.1	42.74	45.9	49.18	51	54.15	55.4	57.8
14.5	-0.128	0.104	39.41	41.22	42.22	44.92	46.62	49.99	53.63	55.71	59.41	60.92	63.89	0.461	0.101	36.28	38.15	39.15	41.81	43.44	46.56	49.8	51.59	54.67	55.89	58.23
15	-0.05	0.101	40.09	41.93	42.94	45.65	47.35	50.7	54.29	56.33	59.94	61.41	64.28	0.506	0.099	36.99	38.85	39.86	42.51	44.12	47.21	50.4	52.16	55.18	56.37	58.66
15.5	0.025	0.1	40.65	42.5	43.52	46.25	47.95	51.28	54.84	56.85	60.39	61.82	64.61	0.55	0.096	37.68	39.54	40.54	43.18	44.79	47.85	51	52.73	55.7	56.86	59.1
16	0.098	0.098	41.08	42.95	43.98	46.72	48.42	51.75	55.28	57.26	60.74	62.14	64.86	0.592	0.094	38.34	40.21	41.21	43.84	45.44	48.48	51.59	53.3	56.22	57.37	59.56
16.5	0.168	0.097	41.37	43.27	44.3	47.05	48.76	52.07	55.57	57.53	60.95	62.32	64.98	0.633	0.092	38.97	40.84	41.85	44.48	46.07	49.09	52.18	53.87	56.75	57.88	60.04
17	0.237	0.096	41.55	43.46	44.49	47.25	48.96	52.25	55.72	57.65	61.01	62.35	64.94	0.673	0.091	39.58	41.46	42.46	45.09	46.68	49.69	52.76	54.44	57.28	58.4	60.53
17.5	0.303	0.095	41.62	43.54	44.58	47.34	49.04	52.32	55.74	57.64	60.94	62.25	64.79	0.711	0.089	40.15	42.04	43.05	45.69	47.28	50.28	53.34	55	57.83	58.94	61.05
18	0.368	0.094	41.62	43.54	44.58	47.34	49.03	52.28	55.67	57.54	60.77	62.06	64.53	0.748	0.088	40.7	42.6	43.62	46.27	47.86	50.87	53.92	55.57	58.38	59.48	61.57

Supplementary Table 11. Smoothed percentiles for girth right calf, for female and male 5 to 18 years.

Right calf circumference (cm)

Age (years)	Female LMS parameters and centiles													Male LMS parameters and centiles												
	L	S	1th	3th	5th	15th	25th	50th (M)	75th	85th	95th	97th	99th	L	S	1th	3th	5th	15th	25th	50th (M)	75th	85th	95th	97th	99th
5	-2.768	0.067	19.36	19.79	20.02	20.69	21.12	22.04	23.13	23.81	25.14	25.73	27.02	-1.624	0.062	19.07	19.51	19.75	20.42	20.85	21.71	22.67	23.24	24.28	24.71	25.6
5.5	-2.65	0.069	19.82	20.26	20.51	21.21	21.68	22.65	23.8	24.52	25.92	26.55	27.91	-1.489	0.067	19.44	19.93	20.2	20.94	21.41	22.37	23.44	24.07	25.23	25.72	26.71
6	-2.543	0.071	20.16	20.63	20.89	21.64	22.13	23.15	24.37	25.13	26.61	27.28	28.72	-1.366	0.072	19.78	20.32	20.61	21.43	21.95	23.01	24.19	24.89	26.18	26.73	27.83
6.5	-2.444	0.075	20.38	20.88	21.16	21.95	22.47	23.56	24.87	25.68	27.29	28.01	29.57	-1.253	0.077	20.1	20.68	21.01	21.9	22.47	23.64	24.94	25.72	27.14	27.75	28.97
7	-2.353	0.08	20.57	21.1	21.4	22.25	22.81	24	25.42	26.31	28.08	28.88	30.64	-1.148	0.083	20.4	21.03	21.39	22.36	22.99	24.27	25.71	26.56	28.14	28.81	30.16
7.5	-2.268	0.086	20.79	21.36	21.68	22.6	23.21	24.5	26.06	27.05	29.02	29.93	31.92	-1.05	0.088	20.68	21.37	21.76	22.82	23.51	24.91	26.49	27.43	29.17	29.9	31.4
8	-2.188	0.091	21.05	21.66	22	22.99	23.64	25.04	26.75	27.83	30.02	31.03	33.28	-0.958	0.094	20.97	21.72	22.13	23.29	24.04	25.56	27.28	28.3	30.2	31	32.64
8.5	-2.114	0.096	21.34	21.99	22.35	23.4	24.11	25.61	27.45	28.63	31.02	32.13	34.61	-0.873	0.098	21.27	22.08	22.53	23.77	24.57	26.21	28.07	29.16	31.21	32.07	33.83
9	-2.043	0.1	21.68	22.36	22.75	23.86	24.61	26.21	28.17	29.43	31.99	33.19	35.87	-0.792	0.102	21.61	22.47	22.95	24.27	25.12	26.86	28.83	30	32.16	33.07	34.93
9.5	-1.976	0.103	22.08	22.8	23.21	24.38	25.16	26.85	28.92	30.25	32.96	34.23	37.07	-0.715	0.104	21.99	22.89	23.39	24.78	25.68	27.51	29.57	30.79	33.04	33.99	35.92
10	-1.913	0.105	22.53	23.29	23.71	24.93	25.75	27.51	29.68	31.07	33.89	35.21	38.16	-0.642	0.106	22.4	23.34	23.87	25.32	26.25	28.15	30.28	31.54	33.85	34.82	36.79
10.5	-1.853	0.106	23.03	23.81	24.25	25.52	26.37	28.19	30.43	31.86	34.76	36.11	39.12	-0.573	0.106	22.84	23.82	24.36	25.86	26.83	28.78	30.96	32.24	34.59	35.58	37.56
11	-1.796	0.105	23.57	24.38	24.84	26.14	27.01	28.88	31.17	32.62	35.55	36.9	39.9	-0.507	0.106	23.3	24.31	24.87	26.41	27.4	29.39	31.6	32.9	35.26	36.25	38.23
11.5	-1.741	0.104	24.19	25.02	25.49	26.82	27.7	29.6	31.89	33.34	36.25	37.58	40.5	-0.444	0.105	23.77	24.81	25.38	26.95	27.95	29.97	32.2	33.5	35.86	36.84	38.8
12	-1.688	0.101	24.87	25.71	26.19	27.53	28.43	30.32	32.6	34.03	36.86	38.14	40.92	-0.384	0.103	24.25	25.3	25.89	27.48	28.49	30.52	32.76	34.05	36.39	37.36	39.28
12.5	-1.638	0.098	25.58	26.42	26.9	28.25	29.14	31.02	33.25	34.64	37.36	38.58	41.19	-0.326	0.101	24.74	25.8	26.39	28	29.02	31.05	33.27	34.55	36.85	37.8	39.68
13	-1.59	0.094	26.26	27.11	27.59	28.93	29.81	31.66	33.83	35.17	37.77	38.91	41.35	-0.271	0.099	25.23	26.3	26.9	28.51	29.52	31.54	33.74	35	37.25	38.18	40.01
13.5	-1.543	0.09	26.9	27.75	28.22	29.55	30.42	32.23	34.34	35.62	38.09	39.17	41.43	-0.217	0.096	25.71	26.79	27.39	28.99	30.01	32.01	34.17	35.41	37.61	38.51	40.28
14	-1.498	0.086	27.48	28.32	28.79	30.1	30.96	32.72	34.77	36	38.35	39.36	41.48	-0.166	0.094	26.19	27.27	27.86	29.46	30.46	32.44	34.57	35.78	37.93	38.8	40.51
14.5	-1.455	0.083	27.98	28.81	29.28	30.57	31.41	33.14	35.13	36.32	38.56	39.53	41.53	-0.116	0.091	26.63	27.71	28.31	29.9	30.89	32.84	34.93	36.12	38.21	39.05	40.71
15	-1.413	0.08	28.38	29.21	29.68	30.96	31.79	33.49	35.43	36.58	38.75	39.68	41.58	-0.068	0.089	27.05	28.13	28.72	30.3	31.28	33.21	35.26	36.42	38.45	39.28	40.88
15.5	-1.373	0.078	28.69	29.52	29.98	31.26	32.08	33.76	35.67	36.8	38.91	39.81	41.65	-0.021	0.086	27.44	28.51	29.1	30.66	31.64	33.54	35.55	36.69	38.67	39.47	41.03
16	-1.334	0.077	28.91	29.74	30.2	31.47	32.29	33.96	35.85	36.97	39.04	39.92	41.72	0.024	0.084	27.79	28.86	29.44	31	31.96	33.84	35.82	36.93	38.87	39.65	41.16
16.5	-1.296	0.077	29.03	29.86	30.32	31.6	32.42	34.09	35.96	37.07	39.13	40	41.77	0.067	0.083	28.11	29.18	29.76	31.3	32.26	34.11	36.06	37.15	39.05	39.81	41.29
17	-1.259	0.077	29.05	29.89	30.35	31.64	32.46	34.13	36	37.11	39.15	40.02	41.77	0.11	0.081	28.4	29.46	30.04	31.58	32.53	34.36	36.29	37.36	39.23	39.97	41.42
17.5	-1.223	0.077	29	29.84	30.31	31.6	32.43	34.1	35.98	37.09	39.13	39.99	41.74	0.151	0.08	28.66	29.73	30.31	31.84	32.78	34.6	36.5	37.56	39.4	40.13	41.55
18	-1.189	0.078	28.89	29.74	30.21	31.51	32.34	34.02	35.91	37.02	39.06	39.93	41.67	0.191	0.079	28.91	29.97	30.55	32.08	33.02	34.83	36.71	37.76	39.57	40.29	41.69

Supplementary Table 12. Smoothed percentiles for FM% by anthropometry, for female and male 5 to 18 years.

Total body fat mass percentage by anthropometry (Slaugter equation)

Age	Female LMS parameters and centiles													Male LMS parameters and centiles												
	L	S	1th	3th	5th	15th	25th	50th (M)	75th	85th	95th	97th	99th	L	S	1th	3th	5th	15th	25th	50th (M)	75th	85th	95th	97th	99th
5	-1.355	0.137	12.3	12.67	12.89	13.61	14.17	15.63	17.5	18.54	20.3	20.99	22.33	-0.664	0.214	7.84	8.42	8.76	9.75	10.44	12.01	14	15.29	17.91	19.11	21.77
5.5	-1.212	0.153	12.34	12.76	13.02	13.84	14.49	16.16	18.33	19.55	21.67	22.52	24.19	-0.638	0.233	7.8	8.38	8.73	9.77	10.52	12.28	14.57	16.04	19.01	20.37	23.36
6	-1.08	0.17	12.16	12.63	12.93	13.85	14.57	16.43	18.87	20.28	22.75	23.75	25.76	-0.614	0.252	7.79	8.39	8.75	9.86	10.67	12.64	15.27	16.95	20.33	21.88	25.26
6.5	-0.961	0.186	11.88	12.41	12.74	13.75	14.55	16.59	19.29	20.87	23.68	24.85	27.2	-0.593	0.272	7.82	8.45	8.82	10	10.88	13.09	16.1	18.03	21.9	23.67	27.54
7	-0.855	0.202	11.62	12.19	12.55	13.66	14.52	16.75	19.69	21.43	24.58	25.91	28.59	-0.573	0.292	7.89	8.54	8.93	10.18	11.14	13.6	17.04	19.25	23.68	25.7	30.12
7.5	-0.76	0.216	11.43	12.06	12.44	13.65	14.58	16.97	20.15	22.04	25.51	26.97	29.97	-0.554	0.31	7.97	8.64	9.06	10.38	11.41	14.13	18.01	20.52	25.54	27.84	32.86
8	-0.675	0.227	11.33	12.02	12.43	13.73	14.74	17.28	20.66	22.68	26.41	28	31.25	-0.536	0.327	8.06	8.75	9.18	10.58	11.69	14.66	18.98	21.78	27.41	29.98	35.61
8.5	-0.594	0.236	11.31	12.05	12.49	13.89	14.96	17.64	21.19	23.31	27.24	28.9	32.33	-0.52	0.342	8.14	8.86	9.31	10.77	11.95	15.17	19.91	23	29.21	32.04	38.25
9	-0.516	0.241	11.38	12.18	12.66	14.15	15.28	18.09	21.77	23.97	28.03	29.75	33.28	-0.504	0.355	8.22	8.96	9.42	10.95	12.19	15.63	20.75	24.1	30.85	33.93	40.68
9.5	-0.44	0.244	11.57	12.42	12.94	14.53	15.72	18.65	22.45	24.71	28.86	30.62	34.19	-0.489	0.366	8.27	9.03	9.51	11.1	12.39	16.01	21.47	25.05	32.27	35.57	42.8
10	-0.365	0.245	11.83	12.75	13.3	14.99	16.25	19.3	23.19	25.49	29.7	31.47	35.05	-0.475	0.375	8.3	9.08	9.57	11.2	12.55	16.32	22.05	25.82	33.42	36.9	44.52
10.5	-0.291	0.244	12.12	13.11	13.7	15.49	16.81	19.96	23.92	26.25	30.48	32.24	35.79	-0.462	0.382	8.3	9.1	9.6	11.27	12.65	16.54	22.47	26.38	34.28	37.9	45.83
11	-0.217	0.243	12.41	13.47	14.1	15.99	17.37	20.62	24.63	26.97	31.2	32.95	36.45	-0.449	0.387	8.27	9.08	9.59	11.29	12.7	16.67	22.73	26.74	34.85	38.57	46.72
11.5	-0.147	0.241	12.71	13.83	14.5	16.49	17.93	21.26	25.32	27.67	31.89	33.63	37.09	-0.437	0.392	8.21	9.03	9.55	11.27	12.69	16.71	22.85	26.92	35.15	38.93	47.22
12	-0.082	0.24	13.01	14.2	14.91	17.01	18.5	21.92	26.03	28.39	32.62	34.36	37.8	-0.426	0.395	8.12	8.95	9.47	11.21	12.64	16.68	22.85	26.94	35.23	39.03	47.38
12.5	-0.026	0.238	13.32	14.59	15.33	17.53	19.08	22.59	26.76	29.15	33.41	35.15	38.59	-0.415	0.397	8.02	8.85	9.38	11.12	12.56	16.6	22.75	26.83	35.12	38.93	47.28
13	0.021	0.237	13.64	14.98	15.76	18.07	19.68	23.27	27.51	29.93	34.25	36.01	39.48	-0.404	0.399	7.9	8.74	9.27	11.01	12.45	16.47	22.58	26.64	34.88	38.68	47
13.5	0.057	0.236	13.97	15.38	16.2	18.6	20.27	23.95	28.26	30.73	35.12	36.91	40.44	-0.394	0.4	7.78	8.62	9.15	10.9	12.33	16.32	22.38	26.4	34.57	38.34	46.6
14	0.08	0.236	14.29	15.77	16.63	19.13	20.84	24.61	29	31.52	36	37.83	41.44	-0.384	0.401	7.67	8.51	9.03	10.78	12.2	16.16	22.15	26.13	34.22	37.95	46.14
14.5	0.093	0.235	14.6	16.14	17.03	19.61	21.37	25.22	29.69	32.26	36.84	38.72	42.43	-0.374	0.402	7.55	8.39	8.92	10.66	12.08	16.01	21.92	25.85	33.86	37.55	45.67
15	0.094	0.235	14.88	16.47	17.39	20.03	21.84	25.75	30.29	32.91	37.61	39.54	43.36	-0.365	0.403	7.44	8.28	8.81	10.55	11.96	15.85	21.69	25.57	33.49	37.15	45.2
15.5	0.087	0.235	15.12	16.74	17.67	20.37	22.21	26.17	30.77	33.44	38.26	40.25	44.2	-0.356	0.403	7.33	8.18	8.7	10.44	11.85	15.7	21.46	25.29	33.13	36.76	44.74
16	0.073	0.236	15.28	16.93	17.88	20.62	22.47	26.47	31.12	33.83	38.76	40.81	44.89	-0.347	0.404	7.23	8.07	8.6	10.34	11.73	15.55	21.24	25.02	32.79	36.38	44.31
16.5	0.053	0.236	15.38	17.04	18	20.76	22.63	26.64	31.32	34.07	39.11	41.21	45.43	-0.339	0.405	7.12	7.97	8.5	10.23	11.63	15.4	21.02	24.77	32.46	36.03	43.91
17	0.028	0.237	15.41	17.07	18.03	20.8	22.67	26.68	31.37	34.16	39.3	41.46	45.81	-0.331	0.405	7.02	7.87	8.4	10.13	11.52	15.26	20.82	24.52	32.15	35.69	43.53
17.5	0.001	0.238	15.37	17.03	17.99	20.75	22.61	26.61	31.31	34.12	39.35	41.56	46.06	-0.323	0.406	6.92	7.77	8.3	10.03	11.42	15.13	20.62	24.29	31.86	35.39	43.19
18	-0.028	0.239	15.27	16.93	17.88	20.63	22.48	26.45	31.14	33.97	39.29	41.55	46.19	-0.315	0.407	6.82	7.68	8.21	9.94	11.32	15	20.43	24.07	31.59	35.1	42.88

Supplementary Table 13. Smoothed percentiles for SMM by anthropometry, for female and male 5 to 18 years.

Skeletal muscle mass (kg) by anthropometry (POORTMANS equation)

Age	Female LMS parameters and centiles													Male LMS parameters and centiles												
	L	S	1th	3th	5th	15th	25th	50th (M)	75th	85th	95th	97th	99th	L	S	1th	3th	5th	15th	25th	50th (M)	75th	85th	95th	97th	99th
5	-0.8	0.148	4.78	5.04	5.18	5.6	5.88	6.47	7.18	7.62	8.48	8.87	9.68	-1.441	0.127	6.97	7.27	7.43	7.91	8.23	8.92	9.78	10.33	11.44	11.96	13.12
5.5	-0.752	0.158	5.14	5.44	5.61	6.1	6.42	7.12	7.96	8.48	9.51	9.97	10.96	-1.326	0.128	7.4	7.72	7.9	8.42	8.77	9.51	10.42	11	12.16	12.69	13.86
6	-0.709	0.168	5.44	5.78	5.97	6.52	6.9	7.69	8.66	9.27	10.47	11.01	12.17	-1.222	0.134	7.77	8.12	8.32	8.89	9.28	10.11	11.12	11.77	13.06	13.65	14.95
6.5	-0.669	0.179	5.7	6.08	6.29	6.91	7.33	8.23	9.33	10.03	11.41	12.04	13.39	-1.126	0.143	8.09	8.49	8.71	9.36	9.79	10.73	11.88	12.62	14.1	14.78	16.28
7	-0.632	0.189	5.96	6.37	6.61	7.3	7.76	8.78	10.02	10.81	12.4	13.11	14.67	-1.037	0.153	8.41	8.85	9.11	9.83	10.32	11.38	12.69	13.52	15.22	16	17.71
7.5	-0.598	0.197	6.25	6.71	6.97	7.73	8.25	9.38	10.78	11.67	13.46	14.28	16.05	-0.955	0.162	8.73	9.22	9.5	10.31	10.86	12.05	13.53	14.47	16.39	17.28	19.23
8	-0.565	0.205	6.58	7.09	7.38	8.21	8.79	10.04	11.59	12.58	14.58	15.49	17.47	-0.877	0.171	9.07	9.61	9.92	10.82	11.43	12.75	14.4	15.45	17.6	18.59	20.78
8.5	-0.535	0.21	6.94	7.49	7.8	8.72	9.35	10.72	12.42	13.51	15.71	16.71	18.89	-0.805	0.179	9.42	10.02	10.36	11.35	12.02	13.49	15.31	16.48	18.86	19.96	22.37
9	-0.507	0.215	7.34	7.93	8.28	9.28	9.96	11.45	13.31	14.51	16.9	18	20.38	-0.736	0.186	9.79	10.44	10.81	11.9	12.64	14.26	16.27	17.56	20.19	21.39	24.04
9.5	-0.48	0.218	7.8	8.45	8.82	9.91	10.66	12.29	14.31	15.61	18.22	19.4	21.98	-0.671	0.193	10.17	10.88	11.29	12.48	13.3	15.07	17.27	18.69	21.55	22.87	25.75
10	-0.454	0.221	8.33	9.03	9.44	10.63	11.44	13.21	15.41	16.82	19.64	20.92	23.7	-0.61	0.199	10.58	11.36	11.8	13.1	13.99	15.92	18.32	19.85	22.95	24.37	27.46
10.5	-0.43	0.222	8.92	9.68	10.13	11.42	12.3	14.22	16.59	18.11	21.14	22.51	25.48	-0.551	0.204	11.03	11.87	12.36	13.77	14.73	16.81	19.4	21.05	24.36	25.87	29.14
11	-0.407	0.222	9.58	10.41	10.89	12.29	13.24	15.3	17.86	19.48	22.71	24.17	27.3	-0.496	0.207	11.53	12.44	12.96	14.48	15.51	17.75	20.5	22.26	25.75	27.33	30.74
11.5	-0.384	0.22	10.32	11.21	11.73	13.23	14.25	16.47	19.19	20.91	24.32	25.85	29.13	-0.442	0.208	12.08	13.05	13.61	15.23	16.33	18.71	21.62	23.45	27.09	28.72	32.2
12	-0.363	0.218	11.11	12.07	12.62	14.23	15.32	17.67	20.55	22.36	25.93	27.52	30.91	-0.391	0.206	12.69	13.73	14.32	16.04	17.2	19.69	22.72	24.61	28.33	29.98	33.49
12.5	-0.343	0.214	11.91	12.94	13.53	15.23	16.38	18.86	21.87	23.75	27.44	29.08	32.55	-0.342	0.202	13.38	14.47	15.1	16.9	18.11	20.7	23.8	25.72	29.46	31.11	34.57
13	-0.323	0.21	12.7	13.78	14.41	16.2	17.4	19.98	23.1	25.04	28.81	30.48	33.99	-0.295	0.196	14.14	15.29	15.94	17.81	19.06	21.71	24.85	26.78	30.48	32.09	35.46
13.5	-0.304	0.205	13.45	14.59	15.24	17.1	18.35	21.01	24.21	26.19	30.01	31.69	35.21	-0.25	0.189	14.96	16.15	16.83	18.76	20.03	22.72	25.86	27.78	31.41	32.98	36.22
14	-0.286	0.201	14.16	15.34	16.01	17.93	19.22	21.96	25.21	27.21	31.07	32.75	36.26	-0.207	0.182	15.8	17.03	17.73	19.7	21	23.71	26.84	28.73	32.29	33.81	36.92
14.5	-0.269	0.197	14.81	16.03	16.72	18.7	20.02	22.81	26.1	28.12	31.99	33.67	37.15	-0.165	0.175	16.62	17.89	18.61	20.62	21.94	24.66	27.78	29.65	33.12	34.6	37.59
15	-0.252	0.193	15.41	16.66	17.37	19.4	20.74	23.57	26.89	28.92	32.78	34.45	37.91	-0.124	0.169	17.39	18.7	19.43	21.48	22.82	25.56	28.68	30.53	33.94	35.38	38.29
15.5	-0.235	0.189	15.95	17.22	17.95	20	21.36	24.22	27.56	29.59	33.44	35.1	38.51	-0.085	0.165	18.09	19.43	20.18	22.27	23.63	26.4	29.53	31.37	34.76	36.18	39.03
16	-0.22	0.185	16.4	17.69	18.43	20.51	21.88	24.75	28.1	30.12	33.94	35.58	38.94	-0.047	0.163	18.68	20.06	20.84	22.98	24.37	27.19	30.35	32.2	35.59	37	39.83
16.5	-0.204	0.182	16.76	18.07	18.81	20.9	22.28	25.16	28.49	30.49	34.26	35.88	39.18	-0.01	0.162	19.18	20.6	21.4	23.61	25.04	27.92	31.15	33.03	36.45	37.87	40.72
17	-0.189	0.179	17.03	18.34	19.09	21.18	22.56	25.42	28.72	30.7	34.41	35.99	39.22	0.026	0.163	19.57	21.05	21.88	24.17	25.64	28.62	31.93	33.86	37.36	38.81	41.7
17.5	-0.175	0.176	17.21	18.52	19.27	21.35	22.72	25.55	28.81	30.75	34.38	35.93	39.07	0.061	0.165	19.86	21.41	22.27	24.66	26.19	29.28	32.72	34.71	38.33	39.82	42.79
18	-0.161	0.173	17.31	18.62	19.36	21.42	22.77	25.56	28.76	30.67	34.21	35.71	38.76	0.095	0.168	20.07	21.69	22.6	25.09	26.69	29.92	33.5	35.58	39.34	40.89	43.97

Supplementary Table 14. Smoothed percentiles for FFM by anthropometry, for female and male 5 to 18 years.

Total body fat free mass by anthropometry (by Slaugter equation)

Age	Female LMS parameters and centiles													Male LMS parameters and centiles												
	L	S	1th	3th	5th	15th	25th	50th (M)	75th	85th	95th	97th	99th	L	S	1th	3th	5th	15th	25th	50th (M)	75th	85th	95th	97th	99th
5	-0.324	0.110	11.31	11.84	12.14	12.94	13.45	14.47	15.60	16.25	17.44	17.93	18.90	0.629	0.119	11.57	12.32	12.73	13.80	14.45	15.70	16.98	17.68	18.89	19.36	20.27
5.5	-0.347	0.109	12.30	12.87	13.18	14.05	14.59	15.70	16.92	17.62	18.90	19.43	20.49	0.978	0.122	11.87	12.77	13.25	14.48	15.21	16.58	17.95	18.68	19.92	20.40	21.31
6	-0.368	0.109	13.16	13.76	14.10	15.01	15.59	16.76	18.05	18.80	20.15	20.72	21.84	0.931	0.126	12.48	13.44	13.95	15.27	16.06	17.55	19.04	19.84	21.19	21.72	22.72
6.5	-0.388	0.115	13.72	14.38	14.74	15.75	16.39	17.69	19.14	19.98	21.52	22.17	23.45	0.523	0.129	13.39	14.31	14.82	16.15	16.97	18.54	20.19	21.10	22.67	23.29	24.49
7	-0.405	0.129	14.02	14.77	15.18	16.34	17.08	18.61	20.33	21.35	23.23	24.02	25.62	-0.035	0.132	14.42	15.28	15.76	17.07	17.90	19.56	21.38	22.43	24.32	25.09	26.63
7.5	-0.422	0.143	14.34	15.17	15.64	16.95	17.80	19.57	21.59	22.80	25.07	26.03	28.00	-0.550	0.135	15.40	16.22	16.69	17.98	18.83	20.57	22.59	23.80	26.07	27.04	29.04
8	-0.438	0.148	14.92	15.81	16.32	17.73	18.65	20.57	22.79	24.12	26.63	27.70	29.91	-0.949	0.138	16.34	17.15	17.61	18.91	19.78	21.62	23.83	25.21	27.91	29.12	31.70
8.5	-0.452	0.145	15.80	16.73	17.25	18.70	19.65	21.63	23.90	25.27	27.83	28.93	31.18	-1.178	0.141	17.22	18.04	18.51	19.85	20.76	22.71	25.12	26.66	29.76	31.19	34.35
9	-0.466	0.141	16.81	17.76	18.30	19.79	20.77	22.79	25.11	26.50	29.11	30.22	32.50	-1.210	0.143	18.03	18.90	19.39	20.82	21.78	23.87	26.45	28.12	31.51	33.08	36.59
9.5	-0.479	0.141	17.77	18.78	19.34	20.93	21.96	24.11	26.57	28.05	30.83	32.02	34.46	-1.063	0.146	18.80	19.74	20.28	21.83	22.87	25.11	27.86	29.61	33.12	34.73	38.24
10	-0.492	0.148	18.62	19.72	20.34	22.07	23.21	25.58	28.33	30.00	33.14	34.49	37.27	-0.797	0.148	19.55	20.61	21.22	22.93	24.07	26.49	29.39	31.19	34.70	36.26	39.58
10.5	-0.503	0.158	19.41	20.61	21.30	23.23	24.51	27.19	30.34	32.26	35.93	37.52	40.84	-0.453	0.149	20.30	21.51	22.20	24.13	25.39	28.02	31.06	32.89	36.34	37.83	40.87
11	-0.515	0.168	20.27	21.59	22.34	24.48	25.91	28.92	32.49	34.69	38.93	40.79	44.69	-0.074	0.150	21.03	22.45	23.24	25.44	26.84	29.70	32.88	34.73	38.11	39.51	42.32
11.5	-0.525	0.172	21.33	22.75	23.57	25.88	27.42	30.69	34.60	37.02	41.72	43.79	48.15	0.282	0.151	21.78	23.45	24.37	26.87	28.43	31.53	34.85	36.74	40.07	41.42	44.06
12	-0.536	0.170	22.65	24.14	24.99	27.40	29.01	32.43	36.50	39.02	43.90	46.05	50.58	0.571	0.151	22.63	24.57	25.62	28.43	30.16	33.49	36.98	38.91	42.25	43.58	46.13
12.5	-0.545	0.162	24.15	25.66	26.53	28.97	30.60	34.03	38.09	40.58	45.39	47.50	51.91	0.766	0.151	23.65	25.85	27.03	30.12	32.00	35.57	39.23	41.23	44.64	45.98	48.53
13	-0.555	0.151	25.67	27.18	28.04	30.46	32.06	35.40	39.32	41.71	46.27	48.25	52.37	0.857	0.150	24.93	27.32	28.60	31.94	33.95	37.73	41.58	43.66	47.19	48.57	51.20
13.5	-0.564	0.139	27.15	28.63	29.47	31.82	33.36	36.56	40.27	42.50	46.73	48.55	52.29	0.853	0.149	26.48	28.99	30.33	33.83	35.94	39.92	43.95	46.14	49.85	51.30	54.06
14	-0.573	0.128	28.52	29.96	30.77	33.03	34.49	37.52	40.98	43.05	46.92	48.57	51.95	0.775	0.147	28.21	30.76	32.13	35.72	37.90	42.03	46.26	48.57	52.50	54.04	56.98
14.5	-0.581	0.117	29.73	31.12	31.90	34.05	35.45	38.30	41.53	43.45	47.00	48.51	51.56	0.654	0.146	29.95	32.49	33.86	37.50	39.72	43.97	48.38	50.80	54.96	56.61	59.76
15	-0.589	0.109	30.73	32.08	32.83	34.90	36.23	38.94	41.99	43.78	47.09	48.47	51.28	0.515	0.144	31.58	34.08	35.44	39.08	41.32	45.67	50.22	52.75	57.14	58.89	62.26
15.5	-0.597	0.104	31.51	32.82	33.55	35.56	36.85	39.47	42.39	44.10	47.24	48.56	51.20	0.375	0.143	33.04	35.49	36.84	40.46	42.71	47.12	51.79	54.42	59.02	60.86	64.44
16	-0.605	0.101	32.03	33.34	34.06	36.05	37.32	39.90	42.77	44.45	47.53	48.81	51.40	0.246	0.141	34.31	36.72	38.05	41.64	43.89	48.34	53.12	55.82	60.60	62.54	66.32
16.5	-0.613	0.101	32.34	33.65	34.38	36.38	37.66	40.25	43.13	44.82	47.91	49.21	51.81	0.128	0.140	35.43	37.80	39.11	42.68	44.93	49.40	54.26	57.03	61.97	63.99	67.95
17	-0.620	0.102	32.47	33.79	34.53	36.56	37.87	40.50	43.45	45.17	48.34	49.66	52.33	0.019	0.138	36.45	38.78	40.08	43.62	45.86	50.36	55.28	58.12	63.21	65.30	69.43
17.5	-0.627	0.104	32.45	33.80	34.56	36.63	37.96	40.66	43.69	45.47	48.74	50.11	52.88	-0.080	0.137	37.40	39.70	40.98	44.49	46.74	51.25	56.24	59.13	64.36	66.51	70.81
18	-0.634	0.107	32.33	33.71	34.48	36.60	37.96	40.73	43.85	45.69	49.07	50.50	53.38	-0.171	0.136	38.28	40.56	41.82	45.32	47.56	52.09	57.13	60.08	65.43	67.66	72.10

Supplementary Table 15. Smoothed percentiles for FM by anthropometry, for female and male 5 to 18 years.

	SF FM (kg)																									
Age	Female LMS parameters and centiles													Male LMS parameters and centiles												
	L	S	1th	3th	5th	15th	25th	50th (M)	75th	85th	95th	97th	99th	L	S	1th	3th	5th	15th	25th	50th (M)	75th	85th	95th	97th	99th
5	-0.479	0.291	1.1	1.3	1.42	1.73	1.93	2.34	2.79	3.07	3.58	3.79	4.24	0.345	0.271	1.39	1.53	1.62	1.88	2.06	2.49	3.06	3.45	4.29	4.7	5.65
5.5	-0.45	0.312	1.2	1.4	1.52	1.85	2.08	2.57	3.14	3.5	4.16	4.44	5.03	0.177	0.306	1.5	1.67	1.78	2.08	2.31	2.82	3.51	4	5.05	5.57	6.78
6	-0.423	0.332	1.26	1.47	1.59	1.97	2.23	2.81	3.54	4	4.91	5.32	6.17	0.032	0.342	1.59	1.78	1.89	2.24	2.5	3.09	3.91	4.49	5.75	6.38	7.87
6.5	-0.399	0.351	1.32	1.54	1.68	2.09	2.39	3.08	3.99	4.59	5.85	6.43	7.71	-0.089	0.379	1.64	1.85	1.98	2.37	2.66	3.33	4.27	4.93	6.42	7.17	8.95
7	-0.376	0.369	1.38	1.62	1.77	2.22	2.56	3.37	4.49	5.27	6.99	7.83	9.76	-0.184	0.416	1.69	1.92	2.06	2.5	2.81	3.57	4.63	5.39	7.11	7.98	10.07
7.5	-0.355	0.385	1.46	1.72	1.87	2.37	2.75	3.68	5.05	6.04	8.34	9.52	12.39	-0.255	0.449	1.77	2.02	2.17	2.65	3	3.84	5.05	5.91	7.88	8.88	11.3
8	-0.336	0.398	1.55	1.82	2	2.54	2.97	4.03	5.65	6.88	9.86	11.47	15.55	-0.302	0.477	1.86	2.14	2.3	2.83	3.22	4.16	5.51	6.49	8.71	9.85	12.6
8.5	-0.317	0.408	1.65	1.95	2.13	2.74	3.21	4.41	6.3	7.77	11.49	13.56	19.07	-0.331	0.499	1.97	2.27	2.45	3.03	3.46	4.51	6.01	7.09	9.58	10.85	13.92
9	-0.3	0.415	1.77	2.09	2.29	2.95	3.47	4.81	6.97	8.69	13.13	15.68	22.66	-0.348	0.515	2.1	2.43	2.64	3.27	3.75	4.9	6.57	7.77	10.52	11.93	15.33
9.5	-0.283	0.42	1.89	2.24	2.46	3.18	3.75	5.23	7.64	9.58	14.69	17.67	25.97	-0.354	0.526	2.27	2.64	2.87	3.57	4.1	5.38	7.23	8.57	11.61	13.17	16.9
10	-0.268	0.423	2.03	2.4	2.64	3.42	4.04	5.66	8.3	10.44	16.1	19.42	28.73	-0.353	0.532	2.48	2.89	3.14	3.93	4.52	5.95	8.01	9.49	12.85	14.56	18.65
10.5	-0.253	0.425	2.17	2.57	2.83	3.67	4.34	6.09	8.94	11.25	17.33	20.88	30.82	-0.346	0.534	2.72	3.18	3.46	4.33	4.99	6.58	8.86	10.49	14.19	16.06	20.51
11	-0.239	0.425	2.31	2.74	3.02	3.92	4.63	6.5	9.54	11.98	18.36	22.06	32.29	-0.337	0.532	2.99	3.5	3.81	4.78	5.51	7.27	9.78	11.57	15.61	17.64	22.45
11.5	-0.225	0.424	2.45	2.91	3.21	4.17	4.92	6.9	10.09	12.63	19.21	22.98	33.27	-0.325	0.529	3.29	3.85	4.19	5.27	6.07	8.01	10.77	12.73	17.12	19.31	24.46
12	-0.212	0.423	2.59	3.08	3.39	4.4	5.2	7.27	10.59	13.21	19.91	23.71	33.91	-0.314	0.525	3.61	4.23	4.61	5.8	6.68	8.81	11.83	13.96	18.7	21.05	26.56
12.5	-0.2	0.421	2.72	3.24	3.56	4.63	5.46	7.62	11.04	13.72	20.5	24.29	34.36	-0.304	0.52	3.95	4.63	5.05	6.36	7.33	9.66	12.93	15.24	20.33	22.85	28.69
13	-0.188	0.418	2.85	3.39	3.73	4.84	5.71	7.94	11.44	14.17	20.99	24.78	34.7	-0.296	0.514	4.3	5.05	5.51	6.93	7.99	10.51	14.05	16.53	21.97	24.63	30.79
13.5	-0.176	0.416	2.98	3.54	3.9	5.04	5.94	8.23	11.81	14.57	21.44	25.21	35.03	-0.291	0.508	4.65	5.46	5.96	7.5	8.64	11.36	15.15	17.79	23.55	26.36	32.81
14	-0.165	0.413	3.1	3.68	4.05	5.23	6.15	8.5	12.14	14.94	21.84	25.61	35.35	-0.289	0.502	4.99	5.87	6.4	8.06	9.28	12.18	16.2	18.99	25.05	27.98	34.7
14.5	-0.155	0.411	3.22	3.82	4.19	5.4	6.34	8.74	12.43	15.26	22.2	25.98	35.68	-0.288	0.497	5.31	6.24	6.81	8.57	9.87	12.94	17.18	20.1	26.41	29.45	36.38
15	-0.144	0.408	3.32	3.93	4.32	5.56	6.52	8.95	12.7	15.56	22.54	26.32	36	-0.287	0.493	5.59	6.57	7.17	9.03	10.39	13.61	18.03	21.06	27.58	30.71	37.79
15.5	-0.134	0.406	3.42	4.04	4.43	5.7	6.67	9.15	12.94	15.83	22.86	26.65	36.33	-0.287	0.489	5.83	6.85	7.48	9.41	10.83	14.17	18.73	21.85	28.52	31.7	38.89
16	-0.125	0.404	3.5	4.14	4.54	5.82	6.82	9.33	13.17	16.09	23.16	26.97	36.66	-0.286	0.487	6.01	7.07	7.71	9.71	11.17	14.6	19.25	22.43	29.19	32.4	39.62
16.5	-0.115	0.401	3.57	4.22	4.63	5.94	6.95	9.5	13.39	16.34	23.47	27.29	37.01	-0.283	0.485	6.13	7.21	7.87	9.91	11.4	14.88	19.59	22.79	29.57	32.79	39.98
17	-0.106	0.4	3.63	4.29	4.71	6.04	7.07	9.66	13.6	16.58	23.78	27.64	37.42	-0.281	0.484	6.19	7.29	7.96	10.02	11.52	15.02	19.75	22.94	29.69	32.87	39.97
17.5	-0.097	0.398	3.68	4.36	4.78	6.14	7.18	9.81	13.81	16.83	24.12	28.03	37.91	-0.278	0.483	6.2	7.3	7.97	10.04	11.54	15.03	19.73	22.89	29.55	32.68	39.63
18	-0.089	0.396	3.72	4.41	4.84	6.23	7.29	9.96	14.03	17.1	24.5	28.47	38.54	-0.274	0.483	6.16	7.26	7.93	9.98	11.47	14.93	19.57	22.68	29.2	32.26	39.03

Part 2. Body composition by multifrequency bioimpedance.

Supplementary Table 16. Smoothed percentiles for mBIA total FFM, for female and male 5 to 18 years.

BIA Fat free mass (kg)

Age	Female LMS parameters and centiles													Male LMS parameters and centiles												
	L	S	1th	3th	5th	15th	25th	50th (M)	75th	85th	95th	97th	99th	L	S	1th	3th	5th	15th	25th	50th (M)	75th	85th	95th	97th	99th
5	-1.339	0.103	12.02	12.45	12.7	13.39	13.85	14.8	15.93	16.62	17.95	18.54	19.78	-0.548	0.102	12.49	12.82	13.01	13.62	14.09	15.22	16.49	17.11	18.05	18.39	18.98
5.5	-1.263	0.1	12.87	13.33	13.59	14.31	14.79	15.78	16.93	17.63	18.96	19.55	20.77	-0.509	0.096	13.35	13.71	13.93	14.58	15.05	16.17	17.41	18.04	19	19.35	19.99
6	-1.193	0.099	13.49	13.98	14.25	15.02	15.51	16.55	17.74	18.47	19.84	20.44	21.68	-0.474	0.094	14.21	14.62	14.86	15.57	16.07	17.21	18.48	19.12	20.15	20.54	21.25
6.5	-1.129	0.102	13.94	14.46	14.76	15.57	16.11	17.22	18.5	19.28	20.76	21.4	22.73	-0.442	0.094	15	15.47	15.73	16.51	17.05	18.23	19.54	20.23	21.35	21.77	22.58
7	-1.07	0.108	14.4	14.97	15.29	16.19	16.77	18	19.42	20.29	21.94	22.66	24.15	-0.412	0.095	15.69	16.21	16.51	17.36	17.93	19.18	20.56	21.29	22.51	22.99	23.89
7.5	-1.015	0.116	14.92	15.55	15.9	16.9	17.56	18.93	20.54	21.52	23.39	24.22	25.93	-0.385	0.098	16.28	16.86	17.19	18.13	18.76	20.1	21.57	22.37	23.73	24.27	25.29
8	-0.963	0.123	15.48	16.18	16.57	17.68	18.41	19.94	21.75	22.86	24.99	25.93	27.9	-0.359	0.104	16.87	17.53	17.9	18.95	19.65	21.13	22.76	23.66	25.2	25.81	26.99
8.5	-0.914	0.13	16.08	16.84	17.28	18.5	19.31	21.01	23.02	24.26	26.66	27.72	29.95	-0.334	0.112	17.47	18.22	18.64	19.83	20.61	22.27	24.11	25.14	26.92	27.63	29.01
9	-0.869	0.137	16.73	17.56	18.04	19.38	20.27	22.15	24.39	25.77	28.46	29.65	32.16	-0.311	0.12	18.05	18.89	19.36	20.69	21.57	23.44	25.52	26.68	28.72	29.54	31.14
9.5	-0.825	0.144	17.42	18.34	18.86	20.33	21.32	23.4	25.88	27.42	30.42	31.76	34.58	-0.29	0.128	18.67	19.59	20.11	21.58	22.56	24.63	26.96	28.27	30.56	31.48	33.29
10	-0.784	0.15	18.18	19.18	19.75	21.37	22.45	24.74	27.49	29.2	32.54	34.03	37.19	-0.269	0.135	19.36	20.36	20.93	22.54	23.62	25.93	28.53	29.99	32.55	33.58	35.6
10.5	-0.745	0.155	19.02	20.11	20.73	22.5	23.68	26.18	29.2	31.07	34.75	36.38	39.86	-0.249	0.142	20.09	21.18	21.8	23.57	24.77	27.35	30.27	31.91	34.77	35.93	38.19
11	-0.708	0.157	20.03	21.2	21.87	23.77	25.04	27.74	30.97	32.98	36.91	38.66	42.37	-0.231	0.151	20.86	22.05	22.73	24.68	26.01	28.9	32.2	34.05	37.27	38.57	41.11
11.5	-0.673	0.156	21.24	22.48	23.19	25.2	26.54	29.37	32.76	34.85	38.92	40.71	44.5	-0.213	0.159	21.73	23.02	23.76	25.9	27.37	30.61	34.32	36.4	40.01	41.46	44.3
12	-0.638	0.151	22.61	23.91	24.65	26.74	28.12	31.03	34.47	36.58	40.65	42.43	46.16	-0.196	0.165	22.78	24.17	24.97	27.3	28.91	32.49	36.61	38.91	42.89	44.49	47.59
12.5	-0.606	0.144	24.04	25.38	26.14	28.27	29.67	32.6	36.03	38.11	42.08	43.81	47.38	-0.179	0.168	24.14	25.61	26.47	28.96	30.7	34.58	39.05	41.52	45.79	47.48	50.78
13	-0.574	0.136	25.41	26.77	27.53	29.68	31.09	34	37.37	39.4	43.23	44.87	48.26	-0.164	0.165	25.87	27.42	28.32	30.94	32.77	36.87	41.58	44.16	48.58	50.33	53.7
13.5	-0.544	0.129	26.62	28	28.77	30.91	32.31	35.18	38.48	40.44	44.12	45.68	48.88	-0.148	0.159	27.92	29.52	30.45	33.16	35.06	39.3	44.14	46.77	51.23	52.98	56.33
14	-0.515	0.124	27.65	29.03	29.8	31.94	33.32	36.16	39.37	41.28	44.82	46.31	49.35	-0.134	0.152	30.13	31.76	32.71	35.48	37.41	41.72	46.61	49.25	53.66	55.38	58.65
14.5	-0.487	0.119	28.48	29.86	30.63	32.76	34.14	36.94	40.1	41.96	45.4	46.85	49.77	-0.12	0.144	32.27	33.92	34.88	37.67	39.61	43.94	48.81	51.41	55.73	57.39	60.55
15	-0.46	0.116	29.11	30.5	31.28	33.42	34.79	37.58	40.71	42.55	45.92	47.34	50.19	-0.106	0.136	34.18	35.83	36.79	39.57	41.51	45.82	50.64	53.19	57.38	58.99	62.02
15.5	-0.434	0.115	29.58	30.99	31.77	33.92	35.31	38.11	41.24	43.07	46.42	47.83	50.64	-0.093	0.13	35.77	37.42	38.37	41.13	43.06	47.34	52.08	54.57	58.65	60.2	63.11
16	-0.408	0.115	29.91	31.34	32.13	34.31	35.71	38.54	41.69	43.54	46.9	48.31	51.13	-0.08	0.126	37.02	38.66	39.6	42.35	44.27	48.52	53.21	55.66	59.63	61.14	63.96
16.5	-0.384	0.115	30.12	31.57	32.38	34.59	36.01	38.87	42.07	43.93	47.33	48.74	51.58	-0.068	0.123	38	39.63	40.57	43.32	45.23	49.48	54.16	56.58	60.5	61.98	64.74
17	-0.36	0.116	30.23	31.7	32.52	34.77	36.21	39.11	42.35	44.23	47.67	49.1	51.96	-0.056	0.122	38.75	40.39	41.34	44.1	46.03	50.32	55.04	57.47	61.39	62.86	65.59
17.5	-0.337	0.117	30.25	31.74	32.57	34.86	36.32	39.26	42.54	44.44	47.92	49.36	52.25	-0.044	0.122	39.35	41.01	41.97	44.77	46.74	51.12	55.93	58.4	62.37	63.85	66.6
18	-0.314	0.118	30.2	31.71	32.56	34.87	36.34	39.32	42.63	44.56	48.07	49.52	52.44	-0.033	0.123	39.85	41.54	42.52	45.4	47.41	51.93	56.89	59.43	63.49	65.01	67.82

Supplementary Table 17. Smoothed percentiles for mBIA total FM, for female and male 5 to 18 years.

Mf-BIA Total FM (kg)																										
Age	Female LMS parameters and centiles													Male LMS parameters and centiles												
	L	S	1th	3th	5th	15th	25th	50th (M)	75th	85th	95th	97th	99th	L	S	1th	3th	5th	15th	25th	50th (M)	75th	85th	95th	97th	99th
5	-0.045	0.591	0.66	0.84	0.97	1.37	1.68	2.5	3.74	4.66	6.76	7.83	10.34	0.975	0.491	0.22	0.52	0.73	1.4	1.83	2.68	3.55	4.02	4.82	5.13	5.72
5.5	-0.048	0.541	0.84	1.06	1.2	1.64	1.99	2.86	4.13	5.05	7.1	8.11	10.47	0.759	0.51	0.24	0.56	0.78	1.43	1.88	2.78	3.77	4.34	5.32	5.71	6.47
6	-0.05	0.506	1.03	1.27	1.42	1.92	2.29	3.22	4.54	5.47	7.53	8.53	10.82	0.573	0.53	0.36	0.68	0.88	1.53	1.98	2.95	4.08	4.75	5.97	6.48	7.49
6.5	-0.05	0.496	1.16	1.43	1.61	2.15	2.56	3.57	5.01	6.02	8.22	9.29	11.74	0.421	0.552	0.5	0.82	1.02	1.66	2.13	3.19	4.5	5.32	6.87	7.54	8.89
7	-0.048	0.515	1.23	1.53	1.71	2.32	2.79	3.93	5.58	6.75	9.34	10.61	13.51	0.307	0.575	0.63	0.95	1.15	1.82	2.33	3.52	5.08	6.09	8.08	8.96	10.8
7.5	-0.043	0.551	1.24	1.56	1.77	2.45	2.98	4.3	6.26	7.67	10.85	12.42	16.09	0.227	0.598	0.74	1.07	1.29	2.02	2.58	3.94	5.79	7.03	9.57	10.72	13.19
8	-0.037	0.586	1.24	1.59	1.82	2.57	3.17	4.69	6.98	8.66	12.5	14.44	18.98	0.173	0.617	0.85	1.21	1.44	2.24	2.86	4.41	6.59	8.08	11.23	12.69	15.88
8.5	-0.028	0.606	1.28	1.66	1.91	2.74	3.4	5.11	7.7	9.63	14.04	16.27	21.54	0.133	0.63	0.96	1.35	1.61	2.48	3.17	4.92	7.43	9.19	12.98	14.77	18.75
9	-0.018	0.609	1.38	1.8	2.07	2.98	3.71	5.58	8.43	10.53	15.34	17.76	23.44	0.1	0.637	1.09	1.52	1.8	2.75	3.5	5.43	8.28	10.3	14.72	16.85	21.64
9.5	-0.007	0.596	1.54	2.01	2.31	3.31	4.11	6.14	9.19	11.41	16.43	18.93	24.75	0.072	0.639	1.23	1.69	1.99	3.02	3.84	5.94	9.08	11.34	16.36	18.8	24.37
10	0.006	0.575	1.77	2.29	2.62	3.73	4.59	6.77	9.98	12.28	17.41	19.92	25.7	0.049	0.636	1.38	1.87	2.2	3.29	4.17	6.43	9.82	12.29	17.82	20.54	26.79
10.5	0.019	0.55	2.05	2.63	3	4.21	5.15	7.47	10.81	13.17	18.33	20.82	26.46	0.031	0.63	1.53	2.05	2.39	3.55	4.47	6.86	10.46	13.08	19.01	21.94	28.73
11	0.033	0.523	2.38	3.03	3.44	4.77	5.78	8.24	11.7	14.1	19.25	21.7	27.17	0.019	0.622	1.66	2.21	2.57	3.77	4.73	7.21	10.95	13.69	19.87	22.94	30.06
11.5	0.047	0.495	2.79	3.51	3.97	5.41	6.5	9.09	12.66	15.09	20.21	22.61	27.9	0.013	0.613	1.77	2.34	2.71	3.95	4.94	7.48	11.3	14.09	20.38	23.51	30.75
12	0.061	0.466	3.27	4.07	4.57	6.14	7.3	10.03	13.7	16.15	21.22	23.56	28.66	0.014	0.604	1.86	2.45	2.83	4.1	5.11	7.69	11.54	14.33	20.61	23.72	30.9
12.5	0.076	0.438	3.81	4.7	5.25	6.94	8.17	11.02	14.76	17.22	22.23	24.52	29.42	0.02	0.594	1.93	2.54	2.93	4.22	5.25	7.85	11.7	14.47	20.65	23.69	30.67
13	0.091	0.412	4.41	5.38	5.97	7.77	9.07	12.02	15.81	18.27	23.2	25.42	30.14	0.031	0.583	2	2.62	3.02	4.34	5.38	8	11.83	14.56	20.59	23.53	30.22
13.5	0.106	0.388	5.02	6.07	6.7	8.6	9.95	12.97	16.8	19.24	24.08	26.22	30.75	0.045	0.572	2.07	2.71	3.12	4.47	5.53	8.16	11.96	14.64	20.5	23.32	29.7
14	0.121	0.368	5.61	6.73	7.39	9.37	10.76	13.84	17.68	20.09	24.81	26.89	31.24	0.062	0.56	2.14	2.8	3.23	4.61	5.69	8.34	12.11	14.75	20.43	23.14	29.2
14.5	0.136	0.35	6.16	7.33	8.02	10.06	11.48	14.6	18.42	20.8	25.41	27.42	31.6	0.081	0.548	2.22	2.91	3.35	4.78	5.87	8.55	12.3	14.89	20.41	23.01	28.77
15	0.15	0.336	6.63	7.84	8.55	10.64	12.09	15.22	19.01	21.36	25.87	27.82	31.85	0.102	0.536	2.32	3.03	3.49	4.96	6.08	8.78	12.52	15.08	20.43	22.93	28.41
15.5	0.165	0.325	7	8.24	8.97	11.1	12.56	15.7	19.47	21.79	26.21	28.11	32.02	0.124	0.524	2.42	3.16	3.64	5.16	6.3	9.05	12.78	15.3	20.51	22.92	28.15
16	0.18	0.318	7.26	8.53	9.27	11.43	12.9	16.05	19.81	22.1	26.45	28.31	32.13	0.147	0.512	2.52	3.31	3.8	5.38	6.55	9.34	13.08	15.57	20.66	22.99	28
16.5	0.194	0.313	7.42	8.7	9.45	11.63	13.11	16.26	20.01	22.28	26.58	28.41	32.15	0.17	0.5	2.64	3.47	3.98	5.62	6.83	9.67	13.42	15.89	20.88	23.14	27.95
17	0.208	0.311	7.47	8.77	9.53	11.72	13.2	16.36	20.09	22.35	26.59	28.4	32.09	0.195	0.49	2.77	3.63	4.17	5.87	7.12	10.02	13.79	16.25	21.16	23.36	28.02
17.5	0.222	0.311	7.44	8.75	9.51	11.71	13.2	16.36	20.08	22.32	26.54	28.33	31.97	0.219	0.479	2.9	3.81	4.38	6.14	7.43	10.39	14.21	16.66	21.51	23.67	28.19
18	0.236	0.312	7.35	8.66	9.42	11.64	13.13	16.29	20	22.25	26.44	28.22	31.83	0.243	0.47	3.03	3.99	4.59	6.43	7.76	10.79	14.65	17.1	21.91	24.03	28.45

Supplementary Table 18. Smoothed percentiles for mBIA total body water (TBW), for female and male 5 to 18 years.

BIA total body water (l)																										
Age	Female LMS parameters and percentile													Male LMS parameters and percentile												
	L	S	1th	3th	5th	15th	25th	50th (M)	75th	85th	95th	97th	99th	L	S	1th	3th	5th	15th	25th	50th (M)	75th	85th	95th	97th	99th
5	-1.345	0.121	8.37	8.71	8.9	9.46	9.83	10.62	11.58	12.19	13.39	13.94	15.13	0.835	0.11	8.88	9.19	9.37	9.93	10.35	11.33	12.32	12.75	13.34	13.53	13.86
5.5	-1.241	0.114	9.03	9.39	9.6	10.17	10.55	11.35	12.31	12.9	14.04	14.55	15.64	0.564	0.105	9.5	9.83	10.02	10.6	11.01	11.97	12.96	13.41	14.06	14.29	14.68
6	-1.146	0.11	9.55	9.93	10.14	10.74	11.14	11.96	12.93	13.52	14.66	15.16	16.21	0.293	0.103	10.14	10.5	10.7	11.31	11.74	12.7	13.71	14.21	14.95	15.21	15.68
6.5	-1.058	0.112	9.93	10.34	10.57	11.21	11.63	12.51	13.53	14.16	15.36	15.88	16.98	0.007	0.103	10.77	11.15	11.37	12.02	12.47	13.45	14.52	15.05	15.91	16.22	16.8
7	-0.977	0.118	10.28	10.73	10.98	11.68	12.15	13.12	14.25	14.94	16.27	16.84	18.05	-0.266	0.105	11.34	11.76	12	12.68	13.15	14.17	15.3	15.89	16.87	17.25	17.96
7.5	-0.901	0.126	10.66	11.15	11.43	12.22	12.73	13.82	15.09	15.87	17.37	18.03	19.4	-0.487	0.108	11.86	12.3	12.56	13.29	13.79	14.87	16.09	16.75	17.89	18.33	19.19
8	-0.83	0.133	11.07	11.61	11.92	12.79	13.37	14.58	16	16.88	18.56	19.3	20.85	-0.645	0.113	12.37	12.85	13.13	13.92	14.46	15.64	16.98	17.73	19.04	19.57	20.61
8.5	-0.764	0.139	11.52	12.12	12.46	13.42	14.05	15.39	16.96	17.93	19.79	20.61	22.33	-0.72	0.12	12.89	13.41	13.71	14.58	15.17	16.47	17.98	18.83	20.35	20.97	22.2
9	-0.701	0.145	12.01	12.67	13.04	14.09	14.79	16.26	17.99	19.05	21.1	22	23.88	-0.689	0.127	13.38	13.95	14.28	15.24	15.89	17.34	19.02	19.98	21.69	22.39	23.79
9.5	-0.642	0.15	12.55	13.27	13.67	14.83	15.59	17.2	19.1	20.27	22.52	23.5	25.56	-0.557	0.133	13.87	14.5	14.87	15.93	16.65	18.26	20.12	21.17	23.03	23.78	25.27
10	-0.586	0.156	13.12	13.9	14.35	15.61	16.45	18.22	20.3	21.58	24.04	25.11	27.36	-0.356	0.139	14.38	15.09	15.5	16.68	17.49	19.28	21.33	22.46	24.43	25.21	26.74
10.5	-0.532	0.16	13.74	14.6	15.08	16.46	17.37	19.29	21.55	22.94	25.59	26.75	29.17	-0.12	0.145	14.91	15.7	16.16	17.49	18.4	20.4	22.66	23.87	25.93	26.74	28.27
11	-0.481	0.16	14.48	15.4	15.93	17.4	18.37	20.42	22.82	24.28	27.08	28.29	30.82	0.122	0.151	15.44	16.34	16.86	18.36	19.39	21.63	24.1	25.4	27.55	28.37	29.91
11.5	-0.432	0.157	15.38	16.35	16.9	18.45	19.47	21.59	24.07	25.56	28.4	29.62	32.14	0.343	0.155	16	17.03	17.62	19.31	20.46	22.96	25.65	27.04	29.26	30.1	31.65
12	-0.386	0.15	16.42	17.42	17.99	19.58	20.62	22.76	25.23	26.71	29.48	30.66	33.08	0.524	0.158	16.69	17.84	18.49	20.36	21.63	24.38	27.27	28.73	31.03	31.88	33.44
12.5	-0.341	0.14	17.51	18.53	19.1	20.7	21.74	23.86	26.27	27.7	30.35	31.46	33.73	0.648	0.158	17.6	18.85	19.56	21.58	22.95	25.89	28.95	30.46	32.82	33.68	35.24
13	-0.298	0.131	18.55	19.57	20.15	21.73	22.75	24.82	27.15	28.51	31.01	32.06	34.16	0.706	0.154	18.83	20.14	20.88	22.99	24.42	27.49	30.65	32.21	34.6	35.47	37.04
13.5	-0.257	0.122	19.48	20.5	21.07	22.63	23.62	25.64	27.87	29.16	31.52	32.5	34.45	0.701	0.148	20.33	21.65	22.4	24.55	26	29.13	32.36	33.94	36.36	37.23	38.81
14	-0.217	0.115	20.25	21.27	21.83	23.36	24.34	26.29	28.44	29.68	31.91	32.84	34.67	0.646	0.14	21.98	23.28	24.02	26.15	27.6	30.74	33.99	35.58	38.02	38.9	40.49
14.5	-0.178	0.11	20.87	21.88	22.44	23.95	24.91	26.82	28.9	30.1	32.24	33.12	34.86	0.558	0.133	23.58	24.84	25.57	27.65	29.08	32.19	35.43	37.02	39.47	40.35	41.95
15	-0.141	0.107	21.33	22.34	22.9	24.4	25.35	27.24	29.29	30.46	32.55	33.4	35.08	0.453	0.127	25	26.22	26.92	28.95	30.34	33.4	36.61	38.2	40.64	41.53	43.14
15.5	-0.105	0.105	21.67	22.68	23.24	24.75	25.71	27.59	29.62	30.78	32.85	33.69	35.35	0.345	0.121	26.19	27.36	28.04	30.01	31.37	34.36	37.54	39.12	41.55	42.44	44.06
16	-0.07	0.105	21.89	22.92	23.49	25.02	25.98	27.88	29.92	31.09	33.16	34	35.65	0.243	0.117	27.12	28.26	28.92	30.83	32.16	35.11	38.26	39.82	42.26	43.15	44.77
16.5	-0.037	0.105	22.02	23.07	23.64	25.2	26.18	28.1	30.17	31.35	33.43	34.28	35.94	0.153	0.114	27.85	28.96	29.6	31.48	32.79	35.71	38.84	40.41	42.85	43.75	45.38
17	-0.004	0.106	22.07	23.14	23.73	25.32	26.31	28.26	30.36	31.56	33.67	34.52	36.2	0.077	0.112	28.42	29.52	30.15	32.01	33.31	36.23	39.38	40.96	43.42	44.32	45.98
17.5	0.028	0.108	22.07	23.16	23.75	25.37	26.38	28.37	30.5	31.7	33.84	34.71	36.4	0.012	0.112	28.89	29.97	30.61	32.46	33.77	36.71	39.9	41.5	44.01	44.93	46.62
18	0.059	0.109	22.01	23.12	23.73	25.37	26.39	28.41	30.57	31.79	33.95	34.82	36.53	-0.046	0.113	29.29	30.38	31.01	32.88	34.2	37.19	40.45	42.09	44.66	45.61	47.34

Supplementary Table 19. Smoothed percentiles for mBIA Truncal Fat Mass (kg), for female and male 5 to 18 years.

BIA Trunkal FM (kg)

Age (years)	Female LMS parameters and centiles													Male LMS parameters and centiles												
	L	S	1th	3th	5th	15th	25th	50th (M)	75th	85th	95th	97th	99th	L	S	1th	3th	5th	15th	25th	50th (M)	75th	85th	95th	97th	99th
5	-0.578	0.365	0.96	1.08	1.15	1.36	1.53	1.92	2.51	2.94	4.02	4.61	6.18	0.573	0.385	0.47	0.62	0.71	0.96	1.14	1.54	1.98	2.23	2.63	2.79	3.09
5.5	-0.517	0.378	0.99	1.12	1.19	1.43	1.61	2.04	2.69	3.17	4.32	4.95	6.6	0.416	0.405	0.55	0.7	0.79	1.07	1.26	1.72	2.25	2.55	3.08	3.29	3.71
6	-0.462	0.391	1.03	1.16	1.25	1.51	1.71	2.19	2.91	3.44	4.71	5.39	7.14	0.274	0.426	0.63	0.78	0.87	1.17	1.38	1.9	2.53	2.91	3.59	3.87	4.43
6.5	-0.412	0.407	1.06	1.21	1.31	1.6	1.82	2.36	3.16	3.75	5.17	5.92	7.84	0.152	0.45	0.69	0.85	0.95	1.26	1.5	2.08	2.83	3.3	4.17	4.55	5.32
7	-0.365	0.424	1.1	1.26	1.37	1.69	1.94	2.54	3.44	4.11	5.69	6.54	8.65	0.053	0.475	0.75	0.92	1.02	1.36	1.61	2.26	3.15	3.72	4.85	5.35	6.4
7.5	-0.321	0.441	1.13	1.31	1.43	1.79	2.06	2.74	3.74	4.49	6.25	7.18	9.5	-0.022	0.501	0.8	0.97	1.08	1.44	1.72	2.45	3.48	4.17	5.6	6.26	7.69
8	-0.28	0.455	1.16	1.37	1.49	1.89	2.19	2.94	4.05	4.87	6.8	7.81	10.31	-0.075	0.525	0.84	1.03	1.14	1.53	1.83	2.63	3.82	4.65	6.41	7.25	9.13
8.5	-0.242	0.464	1.21	1.43	1.57	2	2.33	3.16	4.37	5.26	7.33	8.41	11.02	-0.11	0.546	0.89	1.08	1.2	1.61	1.94	2.82	4.17	5.13	7.24	8.28	10.67
9	-0.206	0.468	1.27	1.52	1.67	2.14	2.51	3.4	4.72	5.68	7.88	9	11.69	-0.132	0.564	0.93	1.13	1.26	1.7	2.05	3.01	4.51	5.6	8.07	9.31	12.21
9.5	-0.172	0.469	1.36	1.63	1.8	2.32	2.72	3.71	5.13	6.15	8.47	9.64	12.4	-0.143	0.577	0.97	1.18	1.32	1.79	2.17	3.21	4.85	6.06	8.86	10.28	13.67
10	-0.139	0.466	1.48	1.78	1.96	2.55	2.99	4.07	5.61	6.7	9.15	10.36	13.18	-0.146	0.586	1.01	1.23	1.38	1.88	2.29	3.4	5.17	6.5	9.58	11.16	14.96
10.5	-0.108	0.461	1.62	1.95	2.16	2.81	3.3	4.48	6.14	7.31	9.88	11.13	14	-0.14	0.592	1.05	1.29	1.45	1.98	2.41	3.59	5.49	6.9	10.21	11.91	16.01
11	-0.079	0.454	1.79	2.16	2.39	3.11	3.65	4.94	6.73	7.97	10.66	11.95	14.87	-0.129	0.595	1.09	1.34	1.51	2.07	2.54	3.79	5.79	7.28	10.76	12.54	16.82
11.5	-0.051	0.446	1.98	2.39	2.65	3.44	4.03	5.43	7.36	8.67	11.47	12.79	15.76	-0.111	0.596	1.12	1.4	1.58	2.18	2.67	3.99	6.08	7.63	11.22	13.05	17.4
12	-0.024	0.436	2.19	2.65	2.93	3.8	4.45	5.96	8.01	9.39	12.29	13.65	16.64	-0.089	0.594	1.16	1.45	1.64	2.28	2.8	4.19	6.36	7.96	11.61	13.44	17.78
12.5	0.002	0.425	2.42	2.92	3.23	4.19	4.88	6.51	8.67	10.11	13.09	14.47	17.49	-0.063	0.59	1.2	1.51	1.71	2.39	2.93	4.39	6.63	8.25	11.92	13.74	17.98
13	0.027	0.414	2.65	3.2	3.54	4.57	5.32	7.04	9.31	10.8	13.84	15.24	18.25	-0.035	0.584	1.24	1.57	1.79	2.5	3.08	4.59	6.88	8.53	12.17	13.96	18.05
13.5	0.051	0.403	2.89	3.49	3.85	4.96	5.75	7.56	9.91	11.44	14.53	15.93	18.92	-0.005	0.577	1.28	1.64	1.86	2.62	3.22	4.79	7.13	8.78	12.37	14.11	18.04
14	0.074	0.392	3.13	3.77	4.16	5.33	6.17	8.06	10.47	12.03	15.14	16.53	19.49	0.027	0.568	1.33	1.7	1.94	2.74	3.37	4.99	7.36	9.01	12.54	14.22	17.96
14.5	0.096	0.382	3.36	4.04	4.45	5.68	6.55	8.51	10.97	12.54	15.65	17.04	19.95	0.061	0.558	1.37	1.77	2.02	2.87	3.53	5.2	7.59	9.23	12.68	14.3	17.86
15	0.117	0.372	3.57	4.29	4.71	6	6.9	8.89	11.39	12.97	16.06	17.42	20.28	0.096	0.548	1.41	1.83	2.11	2.99	3.68	5.4	7.82	9.44	12.81	14.37	17.74
15.5	0.138	0.363	3.76	4.5	4.94	6.26	7.18	9.21	11.72	13.29	16.34	17.68	20.46	0.133	0.538	1.45	1.9	2.19	3.12	3.84	5.61	8.04	9.65	12.93	14.43	17.63
16	0.158	0.354	3.91	4.67	5.13	6.47	7.4	9.44	11.94	13.49	16.49	17.79	20.49	0.171	0.528	1.49	1.97	2.27	3.26	4.01	5.82	8.26	9.85	13.06	14.49	17.54
16.5	0.177	0.346	4.02	4.8	5.26	6.62	7.56	9.59	12.06	13.59	16.51	17.77	20.38	0.21	0.518	1.52	2.04	2.36	3.39	4.17	6.02	8.48	10.06	13.18	14.57	17.48
17	0.196	0.339	4.1	4.89	5.35	6.72	7.65	9.67	12.09	13.58	16.41	17.63	20.13	0.248	0.509	1.56	2.1	2.44	3.53	4.33	6.23	8.7	10.27	13.33	14.67	17.45
17.5	0.215	0.333	4.15	4.94	5.4	6.76	7.68	9.67	12.04	13.49	16.22	17.39	19.78	0.286	0.5	1.59	2.16	2.52	3.66	4.5	6.44	8.92	10.48	13.48	14.78	17.47
18	0.232	0.326	4.17	4.96	5.42	6.76	7.68	9.62	11.93	13.33	15.96	17.08	19.36	0.323	0.492	1.62	2.23	2.61	3.8	4.67	6.65	9.15	10.7	13.65	14.92	17.52

Supplementary Table 20. Smoothed percentiles for mBIA Appendicular Skeletal Muscle Mass (kg), for female and male 5 to 18 years.

BIA ASMM (kg)

Age (years)	Female LMS parameters and percentile													Male LMS parameters and percentile												
	L	S	1th	3th	5th	15th	25th	50th (M)	75th	85th	95th	97th	99th	L	S	1th	3th	5th	15th	25th	50th (M)	75th	85th	95th	97th	99th
5	-0.974	0.165	3.05	3.22	3.32	3.61	3.8	4.23	4.76	5.1	5.8	6.13	6.85	-0.337	0.165	3.22	3.44	3.56	3.91	4.14	4.62	5.17	5.5	6.13	6.4	6.95
5.5	-0.892	0.154	3.39	3.57	3.68	3.98	4.19	4.63	5.16	5.5	6.17	6.47	7.13	-0.286	0.15	3.62	3.85	3.98	4.34	4.57	5.05	5.6	5.93	6.53	6.79	7.31
6	-0.816	0.149	3.66	3.85	3.97	4.29	4.5	4.96	5.51	5.85	6.52	6.82	7.45	-0.24	0.142	4.01	4.25	4.39	4.77	5.02	5.51	6.08	6.41	7.02	7.27	7.79
6.5	-0.747	0.151	3.85	4.06	4.19	4.53	4.77	5.26	5.84	6.21	6.92	7.23	7.9	-0.197	0.139	4.36	4.62	4.77	5.17	5.43	5.96	6.55	6.89	7.53	7.79	8.32
7	-0.683	0.158	4.03	4.26	4.4	4.79	5.04	5.59	6.24	6.65	7.44	7.79	8.54	-0.158	0.139	4.65	4.93	5.09	5.53	5.81	6.37	7	7.37	8.04	8.32	8.88
7.5	-0.623	0.168	4.22	4.49	4.64	5.07	5.37	5.99	6.73	7.19	8.1	8.5	9.36	-0.121	0.143	4.89	5.2	5.38	5.85	6.16	6.78	7.47	7.88	8.61	8.92	9.53
8	-0.567	0.177	4.43	4.73	4.9	5.39	5.72	6.42	7.27	7.8	8.83	9.3	10.28	-0.087	0.151	5.13	5.47	5.67	6.2	6.55	7.24	8.02	8.47	9.3	9.65	10.33
8.5	-0.514	0.186	4.67	5	5.19	5.74	6.11	6.9	7.85	8.45	9.61	10.13	11.24	-0.055	0.16	5.37	5.76	5.98	6.58	6.97	7.76	8.65	9.17	10.12	10.51	11.3
9	-0.465	0.193	4.92	5.29	5.5	6.11	6.53	7.41	8.47	9.14	10.45	11.03	12.27	-0.024	0.168	5.63	6.07	6.31	6.99	7.43	8.32	9.32	9.91	10.98	11.43	12.33
9.5	-0.418	0.201	5.2	5.61	5.85	6.53	6.98	7.97	9.16	9.9	11.36	12.01	13.39	0.005	0.175	5.92	6.4	6.68	7.43	7.91	8.9	10.02	10.67	11.87	12.36	13.36
10	-0.373	0.207	5.51	5.96	6.22	6.97	7.48	8.57	9.9	10.72	12.34	13.06	14.58	0.032	0.181	6.25	6.78	7.07	7.9	8.44	9.54	10.77	11.49	12.82	13.37	14.48
10.5	-0.331	0.211	5.85	6.35	6.64	7.47	8.03	9.23	10.68	11.58	13.35	14.13	15.78	0.058	0.188	6.58	7.16	7.49	8.41	9.01	10.23	11.6	12.41	13.89	14.5	15.74
11	-0.291	0.212	6.27	6.81	7.13	8.03	8.63	9.93	11.49	12.45	14.33	15.16	16.89	0.083	0.195	6.92	7.57	7.94	8.96	9.63	10.99	12.53	13.43	15.09	15.78	17.16
11.5	-0.252	0.207	6.77	7.35	7.69	8.65	9.29	10.66	12.29	13.29	15.22	16.06	17.82	0.106	0.202	7.29	8.02	8.42	9.56	10.3	11.82	13.53	14.54	16.39	17.16	18.71
12	-0.215	0.198	7.35	7.97	8.32	9.33	10	11.4	13.06	14.07	15.98	16.81	18.52	0.129	0.207	7.75	8.54	8.99	10.24	11.06	12.73	14.61	15.72	17.75	18.6	20.29
12.5	-0.18	0.187	7.97	8.61	8.98	10.01	10.69	12.11	13.75	14.74	16.61	17.4	19.03	0.151	0.206	8.34	9.2	9.69	11.04	11.92	13.72	15.74	16.93	19.09	19.99	21.79
13	-0.146	0.175	8.58	9.24	9.61	10.65	11.33	12.74	14.35	15.31	17.1	17.86	19.39	0.172	0.2	9.11	10.03	10.54	11.98	12.9	14.79	16.9	18.13	20.36	21.29	23.13
13.5	-0.113	0.165	9.13	9.8	10.17	11.22	11.9	13.28	14.86	15.78	17.49	18.21	19.65	0.192	0.19	10.02	10.98	11.52	13.01	13.96	15.9	18.05	19.29	21.54	22.47	24.31
14	-0.082	0.156	9.61	10.28	10.66	11.7	12.37	13.74	15.27	16.16	17.8	18.49	19.85	0.211	0.179	11	11.99	12.55	14.07	15.04	16.99	19.14	20.38	22.6	23.51	25.31
14.5	-0.051	0.149	10	10.68	11.05	12.1	12.76	14.11	15.61	16.49	18.07	18.73	20.04	0.23	0.167	11.95	12.93	13.53	15.06	16.03	17.98	20.1	21.31	23.48	24.36	26.11
15	-0.022	0.145	10.3	10.99	11.37	12.41	13.08	14.42	15.91	16.77	18.32	18.96	20.24	0.248	0.158	12.8	13.82	14.38	15.91	16.88	18.8	20.87	22.06	24.16	25.02	26.69
15.5	0.007	0.143	10.52	11.22	11.6	12.66	13.33	14.68	16.17	17.02	18.57	19.2	20.46	0.265	0.149	13.5	14.52	15.08	16.6	17.55	19.44	21.47	22.63	24.67	25.49	27.11
16	0.034	0.142	10.68	11.38	11.78	12.85	13.53	14.9	16.4	17.26	18.81	19.45	20.71	0.282	0.143	14.05	15.07	15.63	17.13	18.08	19.94	21.93	23.06	25.05	25.85	27.41
16.5	0.061	0.143	10.77	11.49	11.89	12.99	13.68	15.07	16.59	17.47	19.03	19.68	20.95	0.299	0.139	14.48	15.5	16.06	17.56	18.5	20.35	22.33	23.44	25.39	26.18	27.72
17	0.087	0.144	10.81	11.55	11.96	13.07	13.78	15.2	16.74	17.63	19.22	19.87	21.16	0.314	0.137	14.81	15.84	16.4	17.92	18.87	20.73	22.71	23.83	25.78	26.57	28.1
17.5	0.112	0.146	10.81	11.56	11.98	13.12	13.84	15.28	16.85	17.75	19.37	20.03	21.33	0.33	0.137	15.06	16.12	16.69	18.25	19.21	21.11	23.12	24.26	26.24	27.04	28.6
18	0.136	0.148	10.78	11.54	11.97	13.13	13.86	15.32	16.92	17.83	19.47	20.13	21.45	0.345	0.139	15.26	16.35	16.94	18.55	19.55	21.5	23.58	24.75	26.8	27.62	29.22

Supplementary Table 21. Smoothed percentiles for mBIA phase angle at 50kHz in supine position, for female and male 5 to 18 years.

BIA supine Phase angle 50 kHz (∘)

Age (years)	Female LMS parameters and centiles													Male LMS parameters andd centiles												
	L	S	1th	3th	5th	15th	25th	50th (M)	75th	85th	95th	97th	99th	L	S	1th	3th	5th	15th	25th	50th (M)	75th	85th	95th	97th	99th
5	-1.019	0.072	4.14	4.25	4.32	4.49	4.6	4.83	5.07	5.22	5.48	5.59	5.8	-0.99	0.095	3.71	3.84	3.92	4.12	4.26	4.53	4.84	5.03	5.37	5.52	5.82
5.5	-1.155	0.073	4.16	4.28	4.34	4.52	4.63	4.86	5.11	5.26	5.53	5.65	5.88	-0.816	0.091	3.86	4	4.08	4.29	4.42	4.7	5	5.18	5.51	5.65	5.93
6	-1.278	0.074	4.18	4.3	4.37	4.55	4.66	4.89	5.15	5.3	5.59	5.71	5.95	-0.656	0.087	4.01	4.15	4.23	4.44	4.58	4.85	5.15	5.33	5.64	5.77	6.04
6.5	-1.386	0.075	4.2	4.32	4.39	4.57	4.68	4.92	5.18	5.34	5.63	5.76	6.01	-0.509	0.085	4.12	4.26	4.34	4.56	4.7	4.97	5.26	5.43	5.74	5.86	6.11
7	-1.475	0.076	4.22	4.34	4.4	4.59	4.7	4.94	5.21	5.37	5.68	5.81	6.07	-0.373	0.082	4.18	4.33	4.41	4.62	4.76	5.03	5.32	5.49	5.78	5.9	6.14
7.5	-1.545	0.077	4.24	4.35	4.42	4.6	4.72	4.96	5.24	5.4	5.72	5.85	6.12	-0.246	0.081	4.22	4.36	4.44	4.66	4.8	5.06	5.35	5.51	5.8	5.91	6.14
8	-1.596	0.078	4.25	4.37	4.43	4.62	4.74	4.98	5.26	5.43	5.75	5.89	6.17	-0.128	0.079	4.26	4.41	4.49	4.71	4.85	5.12	5.4	5.56	5.84	5.95	6.17
8.5	-1.629	0.079	4.26	4.38	4.45	4.63	4.75	5	5.29	5.46	5.79	5.93	6.22	-0.016	0.079	4.32	4.47	4.56	4.78	4.92	5.18	5.47	5.63	5.9	6.01	6.23
9	-1.648	0.08	4.28	4.4	4.47	4.65	4.78	5.03	5.32	5.49	5.83	5.97	6.27	0.089	0.078	4.36	4.52	4.6	4.83	4.97	5.24	5.52	5.68	5.96	6.07	6.28
9.5	-1.654	0.08	4.3	4.42	4.49	4.68	4.81	5.06	5.36	5.54	5.87	6.02	6.33	0.188	0.078	4.37	4.54	4.62	4.85	4.99	5.27	5.55	5.71	5.98	6.09	6.3
10	-1.647	0.081	4.33	4.45	4.52	4.72	4.84	5.1	5.4	5.59	5.93	6.08	6.39	0.282	0.079	4.37	4.53	4.62	4.85	5	5.27	5.56	5.72	5.99	6.1	6.31
10.5	-1.627	0.082	4.37	4.49	4.56	4.76	4.89	5.15	5.46	5.64	5.99	6.15	6.46	0.371	0.08	4.36	4.53	4.62	4.86	5.01	5.29	5.57	5.73	6.01	6.12	6.32
11	-1.594	0.082	4.41	4.53	4.61	4.81	4.94	5.21	5.52	5.71	6.06	6.22	6.54	0.457	0.081	4.36	4.54	4.63	4.88	5.03	5.32	5.61	5.77	6.05	6.16	6.37
11.5	-1.548	0.083	4.45	4.58	4.66	4.86	4.99	5.27	5.59	5.78	6.14	6.3	6.62	0.538	0.083	4.38	4.56	4.66	4.92	5.07	5.37	5.67	5.84	6.12	6.23	6.45
12	-1.491	0.083	4.5	4.63	4.71	4.92	5.06	5.34	5.66	5.85	6.22	6.38	6.71	0.617	0.084	4.42	4.61	4.71	4.98	5.14	5.44	5.76	5.93	6.22	6.33	6.55
12.5	-1.423	0.084	4.55	4.69	4.77	4.98	5.12	5.41	5.74	5.94	6.31	6.47	6.8	0.692	0.086	4.47	4.67	4.78	5.06	5.23	5.54	5.87	6.05	6.35	6.46	6.69
13	-1.348	0.085	4.6	4.74	4.82	5.04	5.19	5.48	5.82	6.02	6.39	6.55	6.89	0.763	0.088	4.54	4.76	4.87	5.16	5.34	5.67	6.01	6.19	6.5	6.62	6.85
13.5	-1.267	0.085	4.65	4.8	4.88	5.11	5.25	5.55	5.89	6.1	6.48	6.64	6.97	0.833	0.089	4.64	4.86	4.98	5.29	5.47	5.82	6.17	6.36	6.68	6.81	7.05
14	-1.183	0.086	4.7	4.85	4.93	5.16	5.31	5.62	5.97	6.17	6.56	6.72	7.05	0.9	0.09	4.74	4.98	5.1	5.42	5.62	5.98	6.34	6.54	6.87	7	7.25
14.5	-1.096	0.086	4.74	4.9	4.98	5.22	5.37	5.68	6.03	6.24	6.63	6.79	7.13	0.964	0.091	4.84	5.09	5.22	5.56	5.76	6.13	6.51	6.71	7.05	7.19	7.44
15	-1.01	0.087	4.78	4.94	5.03	5.27	5.43	5.74	6.1	6.31	6.7	6.86	7.19	1.026	0.092	4.93	5.19	5.32	5.68	5.88	6.27	6.66	6.87	7.21	7.35	7.6
15.5	-0.925	0.087	4.81	4.98	5.07	5.31	5.47	5.8	6.16	6.37	6.76	6.92	7.25	1.086	0.092	5.01	5.28	5.42	5.78	5.99	6.39	6.79	7	7.35	7.49	7.75
16	-0.842	0.088	4.84	5.01	5.1	5.35	5.52	5.84	6.21	6.42	6.81	6.98	7.31	1.145	0.092	5.08	5.35	5.5	5.87	6.09	6.49	6.9	7.11	7.47	7.61	7.87
16.5	-0.763	0.088	4.86	5.03	5.13	5.39	5.55	5.88	6.25	6.47	6.86	7.02	7.35	1.201	0.092	5.13	5.42	5.57	5.95	6.17	6.58	6.99	7.21	7.57	7.71	7.97
17	-0.688	0.088	4.88	5.05	5.15	5.41	5.58	5.92	6.29	6.51	6.9	7.06	7.39	1.256	0.092	5.19	5.48	5.63	6.02	6.24	6.66	7.08	7.3	7.66	7.8	8.06
17.5	-0.617	0.089	4.89	5.07	5.17	5.44	5.61	5.95	6.32	6.54	6.93	7.09	7.42	1.309	0.093	5.23	5.53	5.68	6.08	6.31	6.73	7.15	7.37	7.74	7.88	8.14
18	-0.549	0.089	4.9	5.08	5.18	5.45	5.63	5.97	6.35	6.56	6.96	7.12	7.44	1.361	0.093	5.27	5.57	5.73	6.13	6.37	6.8	7.22	7.44	7.8	7.95	8.21

Supplementary Table 22. Smoothed percentiles for mBIA phase angle in standing position, for female and male 5 to 18 years.

BIA standing Phase angle 50 kHz (∘)

Age (years)	Female LMS parameters and centiles													Male LMS parameters and centiles												
	L	S	1th	3th	5th	15th	25th	50th (M)	75th	85th	95th	97th	99th	L	S	1th	3th	5th	15th	25th	50th (M)	75th	85th	95th	97th	99th
5	-0.599	0.077	3.61	3.73	3.79	3.96	4.07	4.28	4.51	4.64	4.88	4.98	5.17	-0.917	0.111	3.17	3.3	3.37	3.58	3.71	3.99	4.31	4.51	4.88	5.03	5.36
5.5	-0.721	0.078	3.63	3.74	3.81	3.98	4.09	4.3	4.54	4.67	4.92	5.02	5.22	-0.757	0.102	3.35	3.48	3.55	3.76	3.89	4.16	4.47	4.65	4.99	5.13	5.42
6	-0.834	0.079	3.64	3.76	3.82	3.99	4.1	4.32	4.56	4.7	4.96	5.06	5.27	-0.61	0.095	3.51	3.65	3.72	3.93	4.06	4.32	4.61	4.79	5.1	5.23	5.48
6.5	-0.939	0.08	3.66	3.77	3.83	4.01	4.12	4.34	4.59	4.73	4.99	5.1	5.32	-0.475	0.09	3.63	3.77	3.84	4.05	4.18	4.44	4.72	4.88	5.17	5.29	5.53
7	-1.036	0.081	3.67	3.78	3.85	4.02	4.13	4.36	4.61	4.76	5.03	5.14	5.37	-0.351	0.087	3.7	3.84	3.91	4.12	4.24	4.5	4.77	4.93	5.21	5.32	5.54
7.5	-1.122	0.082	3.68	3.79	3.86	4.03	4.14	4.37	4.63	4.78	5.06	5.17	5.41	-0.234	0.085	3.73	3.87	3.94	4.15	4.28	4.53	4.79	4.95	5.21	5.32	5.54
8	-1.196	0.083	3.69	3.8	3.87	4.04	4.15	4.38	4.64	4.8	5.08	5.21	5.45	-0.126	0.084	3.76	3.9	3.97	4.18	4.3	4.55	4.82	4.97	5.23	5.34	5.54
8.5	-1.259	0.083	3.7	3.81	3.88	4.05	4.17	4.4	4.66	4.82	5.11	5.24	5.49	-0.024	0.083	3.78	3.92	4	4.21	4.34	4.58	4.85	5	5.26	5.36	5.56
9	-1.311	0.084	3.71	3.82	3.89	4.07	4.18	4.42	4.68	4.84	5.14	5.27	5.54	0.073	0.083	3.8	3.94	4.02	4.23	4.36	4.61	4.87	5.02	5.27	5.38	5.58
9.5	-1.353	0.085	3.72	3.84	3.9	4.08	4.2	4.44	4.71	4.87	5.18	5.31	5.58	0.164	0.083	3.79	3.94	4.02	4.23	4.36	4.61	4.88	5.02	5.28	5.38	5.57
10	-1.385	0.086	3.74	3.86	3.92	4.1	4.22	4.46	4.74	4.9	5.21	5.35	5.63	0.25	0.083	3.78	3.93	4.01	4.22	4.35	4.61	4.87	5.02	5.27	5.37	5.57
10.5	-1.405	0.086	3.76	3.88	3.94	4.12	4.24	4.49	4.77	4.94	5.25	5.39	5.68	0.333	0.084	3.76	3.92	4	4.22	4.35	4.61	4.88	5.02	5.28	5.38	5.57
11	-1.415	0.087	3.78	3.9	3.97	4.15	4.27	4.52	4.8	4.97	5.3	5.44	5.73	0.411	0.086	3.75	3.91	4	4.22	4.36	4.62	4.9	5.05	5.3	5.41	5.6
11.5	-1.413	0.087	3.8	3.92	3.99	4.18	4.3	4.55	4.84	5.01	5.34	5.49	5.78	0.486	0.088	3.76	3.92	4.01	4.24	4.39	4.66	4.94	5.09	5.36	5.46	5.66
12	-1.401	0.088	3.83	3.95	4.02	4.21	4.33	4.58	4.88	5.05	5.39	5.53	5.84	0.557	0.09	3.77	3.94	4.04	4.28	4.43	4.71	5	5.16	5.43	5.54	5.74
12.5	-1.38	0.089	3.85	3.98	4.05	4.24	4.36	4.62	4.92	5.1	5.44	5.58	5.89	0.626	0.092	3.8	3.98	4.08	4.34	4.49	4.79	5.09	5.25	5.54	5.65	5.86
13	-1.353	0.089	3.88	4	4.07	4.27	4.4	4.66	4.96	5.14	5.48	5.63	5.94	0.692	0.094	3.85	4.04	4.14	4.41	4.58	4.88	5.2	5.37	5.66	5.77	5.99
13.5	-1.321	0.09	3.9	4.03	4.1	4.3	4.43	4.69	5	5.18	5.53	5.68	5.99	0.756	0.096	3.91	4.12	4.22	4.51	4.68	5	5.32	5.5	5.8	5.92	6.14
14	-1.286	0.09	3.93	4.06	4.13	4.33	4.46	4.73	5.04	5.23	5.58	5.73	6.04	0.817	0.097	3.99	4.2	4.31	4.61	4.78	5.12	5.45	5.63	5.94	6.06	6.29
14.5	-1.248	0.091	3.95	4.08	4.16	4.36	4.49	4.77	5.08	5.27	5.62	5.78	6.09	0.876	0.097	4.07	4.29	4.4	4.71	4.89	5.23	5.58	5.76	6.08	6.2	6.43
15	-1.209	0.091	3.98	4.11	4.18	4.39	4.53	4.8	5.12	5.31	5.67	5.82	6.14	0.933	0.097	4.14	4.37	4.49	4.8	4.99	5.34	5.69	5.88	6.2	6.32	6.55
15.5	-1.169	0.092	4	4.13	4.21	4.42	4.56	4.84	5.16	5.35	5.71	5.86	6.18	0.989	0.097	4.21	4.45	4.57	4.89	5.08	5.43	5.79	5.98	6.3	6.42	6.66
16	-1.13	0.092	4.02	4.15	4.23	4.44	4.58	4.87	5.19	5.38	5.75	5.9	6.22	1.042	0.096	4.28	4.52	4.64	4.97	5.16	5.52	5.87	6.06	6.38	6.51	6.74
16.5	-1.091	0.093	4.04	4.17	4.25	4.47	4.61	4.9	5.22	5.42	5.78	5.94	6.26	1.094	0.095	4.34	4.58	4.71	5.04	5.23	5.59	5.95	6.14	6.46	6.59	6.82
17	-1.054	0.093	4.05	4.19	4.27	4.49	4.63	4.92	5.25	5.45	5.82	5.97	6.3	1.144	0.094	4.4	4.64	4.77	5.1	5.3	5.66	6.02	6.21	6.53	6.65	6.89
17.5	-1.019	0.093	4.07	4.21	4.29	4.51	4.66	4.95	5.28	5.48	5.85	6.01	6.33	1.193	0.093	4.45	4.7	4.83	5.16	5.36	5.72	6.08	6.27	6.59	6.71	6.94
18	-0.985	0.094	4.08	4.23	4.31	4.53	4.68	4.97	5.31	5.51	5.88	6.04	6.36	1.24	0.093	4.5	4.75	4.88	5.22	5.42	5.78	6.14	6.33	6.65	6.77	7

Supplementary Table 23. Smoothed percentiles for mBIA resistance at 50kHz in supine position, for female and male 5 to 18 years.

BIA supine Resistance 50 kHz (Ω)

Age (years)	Female LMS parameters and centiles													Male LMS parameters and centiles												
	L	S	1th	3th	5th	15th	25th	50th (M)	75th	85th	95th	97th	99th	L	S	1th	3th	5th	15th	25th	50th (M)	75th	85th	95th	97th	99th
5	-0.793	0.074	712.9	734.3	746	778	798.2	838.5	882.6	908	953.8	972.7	1010.2	0.673	0.084	655.5	683.8	699	738.5	762.4	807.5	853.4	878.4	921	937.7	969.4
5.5	-0.535	0.08	692.9	715.8	728.5	762.7	784.2	826.9	873.3	899.8	947.3	966.8	1005.3	0.588	0.086	649.7	678	693.2	733	757.2	803	849.9	875.5	919.3	936.5	969.4
6	-0.328	0.085	680.3	705	718.6	755.3	778.3	823.8	872.8	900.7	950.3	970.6	1010.4	0.51	0.088	644	672.4	687.6	727.7	752	798.5	846.3	872.5	917.5	935.2	969.1
6.5	-0.158	0.09	673.1	699.9	714.5	754	778.7	827.2	879.3	908.8	961	982.2	1023.7	0.439	0.089	639.3	667.6	682.9	723.2	747.8	794.9	843.5	870.3	916.4	934.7	969.7
7	-0.017	0.095	664.8	693.6	709.3	751.6	777.9	829.5	884.6	915.7	970.5	992.7	1035.9	0.372	0.091	636.9	665.3	680.6	721.3	746.1	793.9	843.5	871	918.3	937.1	973.2
7.5	0.102	0.1	654.6	685.3	702	746.8	774.7	829.2	887	919.6	976.7	999.6	1044.4	0.31	0.093	635.9	664.4	679.8	720.8	746	794.6	845.2	873.3	922	941.3	978.6
8	0.203	0.105	643.7	676	693.6	740.6	769.8	826.7	886.9	920.6	979.5	1003.1	1049	0.253	0.094	633.4	662	677.5	718.7	744.1	793.3	844.8	873.5	923.3	943.1	981.5
8.5	0.289	0.109	632.8	666.5	684.8	733.7	763.9	822.6	884.4	918.9	979	1003	1049.6	0.198	0.095	628.6	657	672.5	713.8	739.3	788.9	841	870.1	920.8	941.1	980.4
9	0.364	0.111	622.4	657.1	675.9	726.1	757.1	816.9	879.7	914.7	975.3	999.5	1046.3	0.147	0.097	622.4	650.8	666.2	707.4	733	782.7	835.3	864.7	916.2	936.9	977
9.5	0.429	0.113	612.8	648.2	667.4	718.4	749.8	810.3	873.5	908.6	969.3	993.4	1039.9	0.099	0.098	615.7	643.9	659.2	700.3	725.9	775.7	828.7	858.4	910.5	931.5	972.3
10	0.487	0.114	604.2	640	659.5	711	742.5	803.3	866.4	901.4	961.7	985.6	1031.6	0.053	0.099	608.1	636	651.2	692.1	717.6	767.5	820.6	850.6	903.2	924.5	965.9
10.5	0.537	0.115	596.7	632.8	652.4	704	735.6	796.2	859	893.6	953.2	976.8	1022	0.009	0.101	599.2	626.8	641.8	682.4	707.8	757.5	810.7	840.8	893.8	915.3	957.2
11	0.583	0.115	590.3	626.6	646.1	697.7	729.2	789.3	851.5	885.7	944.3	967.5	1011.9	-0.033	0.102	588.8	615.9	630.8	671	696.1	745.5	798.6	828.7	881.9	903.5	945.7
11.5	0.623	0.115	585	621.2	640.7	692.1	723.3	782.9	844.3	877.9	935.6	958.3	1001.8	-0.073	0.103	576.9	603.6	618.3	657.8	682.6	731.6	784.4	814.4	867.6	889.2	931.6
12	0.659	0.114	580.6	616.6	636.1	687	718	776.9	837.4	870.5	927.1	949.4	991.9	-0.111	0.104	563.9	590	604.4	643.3	667.7	716.1	768.4	798.2	851.2	872.8	915.3
12.5	0.692	0.113	576.8	612.7	632	682.6	713.2	771.5	831.1	863.6	919.2	941	982.7	-0.147	0.105	550.1	575.6	589.7	627.8	651.8	699.5	751.2	780.7	833.4	854.9	897.4
13	0.721	0.112	573.6	609.3	628.5	678.7	709	766.5	825.3	857.3	911.9	933.3	974.1	-0.182	0.106	535.9	560.9	574.6	612	635.5	682.4	733.4	762.6	814.9	836.4	878.6
13.5	0.748	0.112	570.8	606.4	625.4	675.1	705.2	762	820	851.5	905.2	926.2	966.2	-0.216	0.107	522.2	546.5	559.9	596.5	619.6	665.7	716	745	796.8	818.1	860.2
14	0.773	0.111	568.4	603.7	622.6	672	701.8	758	815.2	846.2	899.1	919.7	959.1	-0.249	0.108	509.4	533.2	546.3	582.2	604.9	650.3	699.9	728.6	780	801.2	843.2
14.5	0.795	0.11	566.2	601.4	620.2	669.1	698.6	754.3	810.8	841.5	893.5	913.9	952.6	-0.28	0.109	498.2	521.5	534.4	569.6	592	636.7	685.9	714.2	765.4	786.6	828.5
15	0.816	0.11	564.3	599.2	617.9	666.6	695.8	750.9	806.8	837.1	888.5	908.5	946.7	-0.31	0.11	488.8	511.7	524.4	559.1	581.1	625.4	674.1	702.4	753.4	774.6	816.6
15.5	0.835	0.109	562.5	597.3	615.9	664.2	693.2	747.9	803.2	833.1	883.9	903.7	941.3	-0.34	0.111	481.2	503.7	516.2	550.5	572.3	616.2	664.8	692.9	744	765.2	807.4
16	0.852	0.108	560.8	595.5	614	662	690.9	745.1	799.9	829.5	879.7	899.2	936.4	-0.368	0.112	475.1	497.3	509.7	543.7	565.4	609	657.5	685.6	736.8	758.1	800.6
16.5	0.868	0.108	559.4	593.9	612.3	660.1	688.7	742.5	796.8	826.2	875.8	895.2	931.9	-0.396	0.113	470.1	492.2	504.5	538.2	559.8	603.3	651.7	679.9	731.4	752.8	795.7
17	0.883	0.107	558	592.4	610.7	658.3	686.8	740.2	794.1	823.1	872.3	891.5	927.8	-0.423	0.114	466	487.9	500.1	533.6	555.1	598.6	647	675.4	727.1	748.7	792.1
17.5	0.897	0.107	556.8	591.1	609.3	656.6	684.9	738	791.5	820.3	869.1	888.1	924	-0.449	0.114	462.4	484.2	496.3	529.7	551.1	594.6	643.1	671.6	723.7	745.5	789.3
18	0.91	0.107	555.6	589.8	608	655.1	683.3	736	789.1	817.8	866.1	884.9	920.5	-0.474	0.115	459.2	480.8	492.9	526.2	547.6	591	639.6	668.2	720.7	742.7	787.1

Supplementary Table 24. Smoothed percentiles for mBIA resistance / height in supine position, for female and male 5 to 18 years.

BIA supine Resistance (Ω) / height (cm)

Age (years)	Female LMS parameters and centiles													Male LMS parameters and centiles												
	L	S	1th	3th	5th	15th	25th	50th (M)	75th	85th	95th	97th	99th	L	S	1th	3th	5th	15th	25th	50th (M)	75th	85th	95th	97th	99th
5	0.605	0.106	6.443	6.804	6.998	7.509	7.819	8.411	9.02	9.354	9.925	10.151	10.581	0.408	0.1	5.864	6.158	6.316	6.737	6.995	7.49	8.006	8.291	8.784	8.98	9.357
5.5	0.576	0.105	6.248	6.592	6.778	7.266	7.564	8.131	8.717	9.038	9.588	9.805	10.221	0.334	0.101	5.669	5.95	6.103	6.508	6.757	7.239	7.743	8.023	8.508	8.702	9.075
6	0.549	0.106	6.07	6.404	6.585	7.06	7.35	7.904	8.477	8.791	9.331	9.545	9.954	0.266	0.102	5.469	5.739	5.885	6.277	6.518	6.987	7.479	7.754	8.232	8.423	8.793
6.5	0.525	0.108	5.883	6.214	6.393	6.866	7.155	7.709	8.282	8.597	9.14	9.355	9.767	0.204	0.103	5.286	5.547	5.689	6.068	6.304	6.761	7.245	7.516	7.989	8.179	8.547
7	0.502	0.113	5.668	6	6.181	6.657	6.949	7.51	8.093	8.414	8.968	9.188	9.61	0.146	0.105	5.144	5.398	5.537	5.909	6.141	6.593	7.073	7.343	7.816	8.006	8.376
7.5	0.481	0.119	5.427	5.764	5.946	6.43	6.728	7.3	7.897	8.228	8.799	9.026	9.463	0.092	0.107	5.022	5.272	5.409	5.778	6.007	6.458	6.939	7.211	7.689	7.881	8.258
8	0.461	0.126	5.178	5.519	5.704	6.197	6.501	7.088	7.702	8.043	8.635	8.87	9.324	0.042	0.11	4.88	5.127	5.262	5.627	5.855	6.305	6.788	7.061	7.545	7.741	8.124
8.5	0.443	0.133	4.933	5.278	5.465	5.966	6.276	6.877	7.508	7.859	8.471	8.714	9.186	-0.005	0.113	4.716	4.958	5.092	5.452	5.679	6.126	6.609	6.884	7.372	7.571	7.96
9	0.425	0.139	4.703	5.049	5.237	5.743	6.057	6.667	7.311	7.67	8.298	8.549	9.034	-0.05	0.116	4.552	4.79	4.921	5.277	5.501	5.946	6.429	6.705	7.198	7.399	7.795
9.5	0.408	0.145	4.495	4.839	5.028	5.533	5.847	6.46	7.11	7.474	8.11	8.364	8.859	-0.092	0.119	4.397	4.63	4.759	5.11	5.332	5.774	6.257	6.534	7.031	7.234	7.636
10	0.393	0.148	4.32	4.658	4.844	5.342	5.653	6.26	6.906	7.268	7.903	8.158	8.653	-0.132	0.122	4.241	4.469	4.595	4.941	5.16	5.599	6.081	6.358	6.858	7.064	7.472
10.5	0.378	0.149	4.182	4.51	4.69	5.175	5.478	6.071	6.702	7.057	7.68	7.93	8.417	-0.17	0.125	4.08	4.303	4.427	4.766	4.982	5.416	5.894	6.172	6.673	6.88	7.292
11	0.364	0.148	4.082	4.397	4.569	5.035	5.325	5.894	6.501	6.842	7.441	7.682	8.15	-0.206	0.127	3.915	4.131	4.251	4.582	4.794	5.22	5.694	5.969	6.469	6.676	7.09
11.5	0.35	0.144	4.017	4.316	4.479	4.92	5.195	5.733	6.307	6.629	7.196	7.423	7.866	-0.241	0.13	3.746	3.955	4.071	4.392	4.598	5.014	5.478	5.75	6.244	6.45	6.862
12	0.337	0.138	3.981	4.262	4.415	4.829	5.087	5.591	6.128	6.429	6.958	7.171	7.584	-0.274	0.132	3.579	3.779	3.891	4.2	4.398	4.801	5.253	5.517	6.001	6.204	6.61
12.5	0.324	0.132	3.963	4.227	4.371	4.759	5	5.471	5.971	6.252	6.744	6.942	7.326	-0.306	0.132	3.417	3.608	3.714	4.009	4.2	4.586	5.021	5.277	5.746	5.942	6.338
13	0.312	0.125	3.956	4.205	4.341	4.705	4.931	5.373	5.841	6.103	6.563	6.747	7.105	-0.336	0.132	3.265	3.446	3.547	3.827	4.008	4.376	4.791	5.036	5.486	5.675	6.057
13.5	0.301	0.12	3.953	4.189	4.318	4.663	4.877	5.295	5.736	5.983	6.416	6.589	6.925	-0.366	0.132	3.127	3.298	3.394	3.659	3.83	4.18	4.574	4.807	5.236	5.417	5.782
14	0.29	0.116	3.95	4.176	4.3	4.63	4.834	5.232	5.653	5.889	6.301	6.466	6.785	-0.394	0.13	3.009	3.171	3.261	3.512	3.674	4.006	4.38	4.601	5.01	5.182	5.53
14.5	0.279	0.113	3.943	4.162	4.281	4.6	4.798	5.182	5.589	5.816	6.213	6.372	6.68	-0.421	0.129	2.913	3.067	3.153	3.392	3.546	3.861	4.218	4.429	4.819	4.983	5.316
15	0.268	0.111	3.932	4.145	4.262	4.573	4.766	5.141	5.538	5.76	6.147	6.302	6.603	-0.448	0.127	2.841	2.988	3.07	3.299	3.446	3.748	4.09	4.292	4.666	4.824	5.144
15.5	0.258	0.11	3.916	4.126	4.241	4.548	4.738	5.107	5.498	5.716	6.098	6.251	6.548	-0.473	0.125	2.789	2.93	3.01	3.23	3.372	3.663	3.993	4.188	4.549	4.702	5.011
16	0.249	0.11	3.899	4.107	4.221	4.524	4.713	5.079	5.467	5.683	6.062	6.214	6.509	-0.498	0.124	2.752	2.89	2.967	3.18	3.318	3.601	3.921	4.11	4.462	4.61	4.911
16.5	0.239	0.11	3.884	4.091	4.204	4.506	4.693	5.057	5.443	5.659	6.037	6.188	6.483	-0.522	0.122	2.726	2.86	2.935	3.143	3.278	3.553	3.865	4.051	4.394	4.539	4.833
17	0.23	0.11	3.875	4.08	4.193	4.493	4.679	5.043	5.427	5.642	6.019	6.171	6.464	-0.545	0.121	2.705	2.836	2.909	3.113	3.245	3.514	3.82	4.002	4.338	4.481	4.77
17.5	0.221	0.11	3.871	4.076	4.188	4.487	4.672	5.034	5.418	5.633	6.009	6.16	6.453	-0.568	0.12	2.686	2.815	2.887	3.087	3.216	3.48	3.781	3.959	4.29	4.43	4.715
18	0.212	0.109	3.874	4.078	4.189	4.487	4.672	5.033	5.415	5.629	6.005	6.156	6.448	-0.59	0.119	2.669	2.795	2.866	3.061	3.188	3.448	3.743	3.919	4.244	4.383	4.663

Supplementary Table 25. Smoothed percentiles for mBIA resistance at 50kHz in standing position, for female and male 5 to 18 years.

BIA standing Resistance 50 kHz (Ω)

Age (years)	Female LMS parameters and centiles													Male LMS parameters and centiles												
	L	S	1th	3th	5th	15th	25th	50th (M)	75th	85th	95th	97th	99th	L	S	1th	3th	5th	15th	25th	50th (M)	75th	85th	95th	97th	99th
5	-0.862	0.078	764.4	787.9	800.9	836.4	858.9	904	953.8	982.6	1034.9	1056.6	1100	-0.294	0.086	711	737.3	751.8	790.8	815.2	863.4	915.4	944.9	997.4	1018.8	1060.9
5.5	-0.715	0.082	749.8	774.5	788.1	825.3	848.8	895.9	947.7	977.6	1031.7	1054.1	1098.8	-0.268	0.088	702.3	728.9	743.5	783	807.8	856.6	909.2	939.2	992.4	1014.1	1056.6
6	-0.58	0.086	735.9	761.7	776	814.9	839.5	888.6	942.4	973.4	1029.3	1052.3	1098.2	-0.244	0.089	693.8	720.7	735.5	775.5	800.5	849.9	903.1	933.4	987.2	1009.1	1052.1
6.5	-0.457	0.09	722.2	749.3	764.3	804.9	830.6	881.9	937.8	970	1027.8	1051.6	1098.7	-0.222	0.091	686.4	713.7	728.6	769	794.3	844.2	898	928.6	983	1005.2	1048.7
7	-0.343	0.095	708.3	736.8	752.5	795.2	822.1	875.7	934	967.5	1027.5	1052.1	1100.7	-0.201	0.093	681.1	708.7	723.8	764.7	790.3	840.8	895.3	926.3	981.4	1003.8	1047.8
7.5	-0.236	0.1	694.3	724.3	740.8	785.6	813.9	869.9	930.9	965.8	1028.1	1053.6	1104	-0.182	0.094	677.3	705.2	720.6	762	788	839.2	894.4	925.8	981.6	1004.3	1048.8
8	-0.137	0.105	680.4	711.9	729.3	776.3	805.9	864.6	928.2	964.5	1029.2	1055.6	1107.6	-0.165	0.095	672.9	701.2	716.7	758.6	784.8	836.6	892.5	924.2	980.6	1003.5	1048.6
8.5	-0.043	0.109	667.3	700.3	718.5	767.6	798.5	859.6	925.6	963.1	1029.8	1057	1110.4	-0.148	0.097	666.9	695.3	711	753.1	779.6	831.8	888.1	920.1	976.9	1000	1045.3
9	0.045	0.113	655.5	689.9	708.8	759.8	791.8	854.9	922.8	961.3	1029.5	1057.2	1111.4	-0.132	0.098	659.5	688.1	703.8	746.2	772.8	825.3	881.8	913.9	971	994.2	1039.7
9.5	0.129	0.117	645.4	680.9	700.4	753	785.8	850.5	919.6	958.8	1027.8	1055.7	1110.2	-0.117	0.099	651.3	680	695.7	738.2	764.9	817.4	874.1	906.3	963.5	986.7	1032.3
10	0.208	0.119	637.1	673.6	693.6	747.3	780.7	846.3	916.1	955.4	1024.5	1052.3	1106.5	-0.103	0.1	642.1	670.7	686.4	728.9	755.6	808.2	864.8	897	954.2	977.4	1023
10.5	0.283	0.119	630.8	668	688.3	742.7	776.4	842.3	912.1	951.2	1019.6	1047	1100.3	-0.089	0.101	631.7	660.2	675.9	718.3	744.9	797.3	853.8	885.9	942.9	966	1011.5
11	0.355	0.119	626.6	664.1	684.6	739.2	772.9	838.5	907.6	946.1	1013.2	1040	1091.9	-0.077	0.102	620.1	648.5	664.1	706.3	732.7	784.9	841.1	873	929.6	952.6	997.8
11.5	0.423	0.118	624.2	661.9	682.3	736.7	770.2	834.9	902.7	940.3	1005.6	1031.5	1081.7	-0.064	0.103	607.6	635.8	651.3	693.1	719.4	771.1	826.9	858.5	914.7	937.5	982.3
12	0.489	0.115	623.4	660.9	681.2	735	768	831.5	897.5	934.1	997.1	1022.1	1070.2	-0.052	0.104	594.6	622.5	637.8	679.3	705.3	756.5	811.7	843	898.6	921.2	965.5
12.5	0.552	0.113	623.6	660.8	680.9	733.9	766.2	828.2	892.4	927.7	988.4	1012.4	1058.4	-0.041	0.105	581.4	609	624.2	665.2	690.9	741.6	796.2	827.2	882.2	904.5	948.3
13	0.612	0.11	624.4	661.1	680.9	733	764.7	825.1	887.3	921.4	979.8	1002.8	1046.9	-0.03	0.106	568.6	595.9	610.9	651.5	676.9	727	781	811.6	866	888	931.3
13.5	0.671	0.108	625.1	661.4	681	732.1	763.2	822.1	882.5	915.4	971.8	993.9	1036.1	-0.02	0.107	556.5	583.6	598.4	638.6	663.7	713.3	766.7	797	850.8	872.6	915.4
14	0.727	0.105	625.5	661.5	680.7	731.2	761.6	819.2	878	910	964.5	985.8	1026.5	-0.01	0.108	545.7	572.4	587.2	626.9	651.8	701	753.9	783.9	837.1	858.7	901
14.5	0.781	0.104	625.3	661	680.1	729.9	759.9	816.5	873.9	905.1	958.1	978.8	1018.1	0	0.109	536.2	562.8	577.4	616.9	641.6	690.4	742.9	772.7	825.5	846.9	888.9
15	0.833	0.102	624.4	660	679	728.4	758.1	813.8	870.3	900.8	952.5	972.7	1011	0.009	0.109	528.3	554.7	569.3	608.5	633.1	681.7	733.9	763.5	816	837.3	879.1
15.5	0.884	0.101	622.9	658.5	677.5	726.6	756.1	811.3	866.9	897	947.7	967.5	1005	0.018	0.11	521.7	548.1	562.6	601.7	626.3	674.7	726.7	756.2	808.6	829.8	871.4
16	0.933	0.101	620.9	656.6	675.6	724.7	754	808.8	863.9	893.6	943.6	963	999.8	0.027	0.111	516.3	542.7	557.2	596.3	620.8	669.1	721.1	750.5	802.8	824	865.5
16.5	0.98	0.1	618.6	654.5	673.5	722.7	751.9	806.5	861.1	890.4	939.8	959	995.2	0.036	0.112	511.8	538.1	552.6	591.7	616.2	664.6	716.5	746	798.2	819.4	860.9
17	1.026	0.1	616.2	652.3	671.4	720.6	749.8	804.2	858.4	887.5	936.3	955.3	991	0.044	0.113	507.8	534.2	548.7	587.9	612.4	660.8	712.8	742.3	794.5	815.7	857.2
17.5	1.071	0.1	613.8	650.1	669.3	718.6	747.8	802	855.9	884.7	933.1	951.8	987	0.052	0.113	504.3	530.7	545.2	584.4	609	657.5	709.5	739.1	791.3	812.5	854.1
18	1.114	0.1	611.4	647.9	667.2	716.6	745.8	799.8	853.4	882.1	929.9	948.4	983.2	0.06	0.114	501	527.5	542	581.3	605.9	654.4	706.6	736.1	788.5	809.7	851.3

Supplementary Table 26. Smoothed percentiles for mBIA resistance / height in standing position, for female and male 5 to 18 years.

BIA standing Resistance (Ω) / height (cm)

Age (years)	Female LMS parameters modeled centiles													Male LMS parameters modeled centiles												
	L	S	1th	3th	5th	15th	25th	50th (M)	75th	85th	95th	97th	99th	L	S	1th	3th	5th	15th	25th	50th (M)	75th	85th	95th	97th	99th
5	0.605	0.106	6.33	6.62	6.78	7.22	7.49	8.02	8.59	8.91	9.48	9.71	10.16	0.164	0.105	6.08	6.38	6.54	6.98	7.26	7.8	8.36	8.68	9.24	9.47	9.9
5.5	0.576	0.105	6.09	6.38	6.53	6.95	7.21	7.73	8.29	8.6	9.16	9.39	9.83	0.122	0.107	5.96	6.26	6.42	6.86	7.13	7.67	8.24	8.57	9.13	9.36	9.8
6	0.549	0.106	5.85	6.13	6.28	6.69	6.94	7.45	7.99	8.3	8.85	9.07	9.5	0.084	0.109	5.74	6.03	6.19	6.62	6.89	7.42	7.98	8.3	8.86	9.09	9.53
6.5	0.525	0.108	5.64	5.9	6.05	6.45	6.69	7.19	7.72	8.02	8.56	8.78	9.21	0.049	0.111	5.5	5.78	5.94	6.36	6.62	7.13	7.69	8	8.56	8.79	9.23
7	0.502	0.113	5.46	5.72	5.86	6.25	6.5	6.98	7.51	7.81	8.34	8.56	8.98	0.018	0.113	5.32	5.59	5.75	6.16	6.41	6.92	7.47	7.78	8.34	8.56	9
7.5	0.481	0.119	5.31	5.57	5.71	6.09	6.34	6.82	7.34	7.64	8.18	8.39	8.82	-0.011	0.115	5.2	5.47	5.62	6.03	6.29	6.79	7.34	7.65	8.21	8.44	8.88
8	0.461	0.126	5.15	5.4	5.55	5.93	6.17	6.65	7.18	7.48	8.01	8.23	8.67	-0.037	0.117	5.08	5.35	5.5	5.9	6.16	6.67	7.21	7.53	8.09	8.32	8.77
8.5	0.443	0.133	4.97	5.23	5.37	5.75	5.99	6.47	6.99	7.29	7.83	8.05	8.49	-0.061	0.12	4.93	5.19	5.34	5.74	5.99	6.5	7.05	7.36	7.92	8.15	8.61
9	0.425	0.139	4.79	5.04	5.18	5.56	5.8	6.28	6.8	7.1	7.65	7.87	8.31	-0.084	0.122	4.76	5.03	5.17	5.56	5.81	6.31	6.86	7.17	7.73	7.96	8.42
9.5	0.408	0.145	4.62	4.87	5.01	5.38	5.62	6.09	6.62	6.92	7.46	7.69	8.13	-0.105	0.125	4.6	4.86	5	5.39	5.63	6.12	6.66	6.98	7.54	7.77	8.22
10	0.393	0.148	4.45	4.69	4.83	5.2	5.43	5.9	6.42	6.73	7.27	7.5	7.94	-0.125	0.127	4.44	4.69	4.83	5.21	5.45	5.94	6.47	6.78	7.33	7.56	8.02
10.5	0.378	0.149	4.28	4.52	4.65	5.01	5.24	5.71	6.22	6.52	7.06	7.29	7.74	-0.144	0.129	4.28	4.53	4.66	5.03	5.27	5.74	6.26	6.57	7.12	7.34	7.8
11	0.364	0.148	4.1	4.33	4.46	4.82	5.04	5.5	6.01	6.3	6.84	7.06	7.51	-0.162	0.13	4.12	4.35	4.48	4.84	5.07	5.53	6.04	6.34	6.88	7.1	7.55
11.5	0.35	0.144	3.93	4.15	4.28	4.62	4.84	5.29	5.78	6.07	6.6	6.82	7.25	-0.178	0.131	3.95	4.17	4.3	4.65	4.87	5.31	5.81	6.1	6.62	6.83	7.27
12	0.337	0.138	3.76	3.98	4.1	4.43	4.64	5.07	5.55	5.83	6.34	6.55	6.98	-0.194	0.131	3.78	3.99	4.11	4.44	4.66	5.08	5.56	5.83	6.34	6.55	6.96
12.5	0.324	0.132	3.6	3.81	3.92	4.24	4.45	4.86	5.32	5.59	6.08	6.29	6.7	-0.209	0.131	3.61	3.82	3.93	4.24	4.45	4.85	5.31	5.57	6.05	6.25	6.65
13	0.312	0.125	3.46	3.66	3.77	4.07	4.26	4.66	5.1	5.35	5.82	6.02	6.41	-0.224	0.131	3.46	3.65	3.76	4.06	4.25	4.64	5.07	5.32	5.78	5.97	6.35
13.5	0.301	0.12	3.33	3.52	3.62	3.91	4.1	4.47	4.89	5.14	5.58	5.77	6.14	-0.237	0.13	3.32	3.5	3.61	3.89	4.07	4.44	4.85	5.09	5.53	5.71	6.08
14	0.29	0.116	3.23	3.4	3.5	3.78	3.96	4.31	4.71	4.94	5.37	5.54	5.9	-0.25	0.129	3.21	3.39	3.48	3.76	3.93	4.28	4.67	4.9	5.32	5.49	5.84
14.5	0.279	0.113	3.14	3.31	3.41	3.67	3.84	4.18	4.56	4.78	5.19	5.36	5.69	-0.263	0.127	3.12	3.29	3.39	3.65	3.82	4.15	4.53	4.75	5.15	5.32	5.66
15	0.268	0.111	3.08	3.25	3.34	3.59	3.75	4.08	4.45	4.66	5.05	5.21	5.53	-0.275	0.126	3.06	3.23	3.32	3.57	3.73	4.06	4.43	4.64	5.03	5.19	5.52
15.5	0.258	0.11	3.04	3.2	3.29	3.53	3.69	4.01	4.36	4.57	4.94	5.09	5.41	-0.286	0.125	3.02	3.18	3.27	3.52	3.68	4	4.35	4.56	4.94	5.1	5.42
16	0.249	0.11	3.01	3.17	3.25	3.49	3.65	3.95	4.3	4.5	4.86	5.01	5.31	-0.297	0.124	3	3.16	3.24	3.49	3.64	3.95	4.3	4.51	4.88	5.03	5.34
16.5	0.239	0.11	2.99	3.14	3.23	3.46	3.61	3.91	4.25	4.44	4.79	4.94	5.23	-0.308	0.123	2.98	3.14	3.22	3.46	3.61	3.92	4.27	4.47	4.83	4.98	5.29
17	0.23	0.11	2.97	3.12	3.21	3.44	3.58	3.88	4.21	4.4	4.74	4.89	5.17	-0.318	0.122	2.97	3.13	3.21	3.45	3.6	3.9	4.24	4.44	4.8	4.95	5.25
17.5	0.221	0.11	2.96	3.1	3.19	3.41	3.56	3.85	4.17	4.36	4.69	4.84	5.12	-0.328	0.121	2.97	3.12	3.2	3.43	3.58	3.88	4.22	4.41	4.77	4.92	5.22
18	0.212	0.109	2.94	3.09	3.17	3.39	3.53	3.82	4.13	4.32	4.65	4.79	5.06	-0.338	0.12	2.96	3.11	3.19	3.42	3.57	3.87	4.2	4.39	4.74	4.89	5.19

Part 3. Body composition by segments with Dual X-ray absorptiometry.

Supplementary Table 27. Smoothed percentiles for total fat mass for female and male 5 to 18 years.

DXA Total Fat Mass (kg)

Age	Female LMS parameters and centiles													Male LMS parameters and centiles												
	L	S	1th	3th	5th	15th	25th	50th (M)	75th	85th	95th	97th	99th	L	S	1th	3th	5th	15th	25th	50th (M)	75th	85th	95th	97th	99th
5	-1.134	0.232	3.28	3.5	3.63	4.03	4.32	4.99	5.92	6.6	8.21	9.09	11.46	-0.576	0.265	2.79	2.97	3.08	3.44	3.74	4.52	5.59	6.23	7.39	7.87	8.85
5.5	-1.042	0.245	3.42	3.67	3.82	4.27	4.59	5.35	6.41	7.19	9.02	10.02	12.7	-0.546	0.278	2.74	2.93	3.04	3.42	3.73	4.55	5.68	6.36	7.6	8.12	9.17
6	-0.958	0.259	3.56	3.84	4.01	4.51	4.88	5.73	6.94	7.82	9.91	11.05	14.08	-0.521	0.292	2.85	3.05	3.18	3.59	3.93	4.85	6.13	6.91	8.33	8.93	10.15
6.5	-0.88	0.275	3.68	4	4.18	4.74	5.15	6.12	7.49	8.49	10.89	12.19	15.66	-0.499	0.309	3.03	3.26	3.4	3.87	4.26	5.31	6.81	7.73	9.43	10.14	11.62
7	-0.809	0.292	3.78	4.12	4.33	4.96	5.41	6.5	8.06	9.2	11.94	13.44	17.43	-0.48	0.327	3.24	3.5	3.67	4.2	4.64	5.87	7.65	8.74	10.8	11.68	13.49
7.5	-0.742	0.31	3.87	4.24	4.47	5.16	5.67	6.89	8.64	9.94	13.06	14.77	19.3	-0.463	0.346	3.46	3.75	3.93	4.54	5.05	6.47	8.56	9.87	12.35	13.42	15.66
8	-0.679	0.326	3.96	4.37	4.62	5.38	5.95	7.3	9.25	10.7	14.19	16.1	21.13	-0.449	0.364	3.67	3.99	4.2	4.88	5.45	7.07	9.49	11.03	13.98	15.26	17.96
8.5	-0.621	0.338	4.09	4.55	4.82	5.66	6.28	7.77	9.93	11.53	15.35	17.42	22.82	-0.436	0.379	3.88	4.23	4.45	5.21	5.84	7.66	10.42	12.19	15.61	17.11	20.28
9	-0.565	0.346	4.29	4.78	5.08	6	6.68	8.33	10.7	12.43	16.56	18.76	24.4	-0.424	0.392	4.07	4.45	4.7	5.52	6.21	8.22	11.3	13.29	17.17	18.89	22.52
9.5	-0.513	0.351	4.54	5.08	5.41	6.43	7.18	8.98	11.56	13.43	17.82	20.12	25.9	-0.414	0.402	4.25	4.66	4.93	5.81	6.56	8.75	12.13	14.32	18.61	20.52	24.58
10	-0.464	0.353	4.84	5.44	5.81	6.93	7.75	9.71	12.5	14.5	19.1	21.47	27.3	-0.404	0.409	4.43	4.87	5.15	6.09	6.89	9.24	12.88	15.25	19.9	21.97	26.38
10.5	-0.416	0.352	5.19	5.85	6.24	7.47	8.36	10.49	13.47	15.58	20.35	22.76	28.56	-0.395	0.414	4.59	5.05	5.35	6.34	7.19	9.67	13.53	16.06	21.01	23.21	27.9
11	-0.371	0.349	5.57	6.29	6.73	8.06	9.03	11.32	14.47	16.68	21.57	23.99	29.71	-0.387	0.417	4.74	5.22	5.52	6.56	7.44	10.04	14.09	16.73	21.9	24.21	29.1
11.5	-0.328	0.344	6	6.79	7.27	8.71	9.76	12.2	15.53	17.82	22.8	25.23	30.84	-0.38	0.419	4.86	5.35	5.67	6.74	7.66	10.35	14.54	17.27	22.61	24.99	30.03
12	-0.287	0.337	6.48	7.34	7.86	9.42	10.55	13.15	16.64	19	24.06	26.48	31.98	-0.373	0.42	4.96	5.47	5.79	6.9	7.84	10.6	14.9	17.69	23.16	25.58	30.71
12.5	-0.248	0.33	7	7.93	8.49	10.18	11.38	14.13	17.77	20.21	25.32	27.73	33.12	-0.367	0.42	5.05	5.57	5.9	7.03	7.99	10.81	15.2	18.04	23.59	26.04	31.23
13	-0.21	0.323	7.53	8.53	9.14	10.94	12.21	15.11	18.88	21.37	26.52	28.92	34.19	-0.361	0.42	5.13	5.66	6	7.15	8.13	11	15.46	18.34	23.95	26.42	31.63
13.5	-0.173	0.315	8.04	9.12	9.76	11.67	13.02	16.04	19.92	22.45	27.62	29.99	35.14	-0.356	0.42	5.21	5.75	6.09	7.26	8.26	11.18	15.69	18.61	24.26	26.75	31.97
14	-0.138	0.308	8.52	9.66	10.34	12.35	13.75	16.88	20.85	23.41	28.56	30.9	35.93	-0.351	0.419	5.29	5.84	6.19	7.37	8.39	11.35	15.91	18.86	24.55	27.04	32.27
14.5	-0.104	0.302	8.95	10.15	10.86	12.95	14.4	17.62	21.64	24.22	29.34	31.63	36.53	-0.346	0.418	5.37	5.92	6.27	7.48	8.51	11.51	16.13	19.09	24.82	27.32	32.56
15	-0.071	0.296	9.31	10.56	11.3	13.46	14.95	18.23	22.3	24.87	29.93	32.19	36.95	-0.342	0.417	5.44	6	6.36	7.58	8.63	11.66	16.33	19.33	25.08	27.59	32.83
15.5	-0.04	0.291	9.6	10.9	11.65	13.87	15.4	18.72	22.8	25.36	30.35	32.55	37.18	-0.338	0.417	5.51	6.08	6.45	7.69	8.74	11.82	16.53	19.55	25.34	27.86	33.11
16	-0.009	0.286	9.82	11.15	11.93	14.19	15.74	19.08	23.15	25.69	30.59	32.74	37.22	-0.334	0.416	5.58	6.16	6.53	7.79	8.85	11.97	16.73	19.78	25.6	28.13	33.39
16.5	0.021	0.282	9.98	11.33	12.12	14.41	15.97	19.32	23.36	25.86	30.67	32.76	37.09	-0.33	0.416	5.65	6.24	6.61	7.88	8.96	12.11	16.93	20	25.85	28.39	33.68
17	0.05	0.278	10.07	11.44	12.24	14.54	16.1	19.45	23.45	25.9	30.59	32.62	36.8	-0.327	0.415	5.71	6.31	6.69	7.97	9.07	12.26	17.12	20.21	26.11	28.66	33.96
17.5	0.078	0.275	10.1	11.49	12.29	14.6	16.16	19.48	23.42	25.82	30.39	32.35	36.37	-0.323	0.415	5.77	6.38	6.76	8.06	9.17	12.39	17.3	20.42	26.36	28.93	34.25
18	0.105	0.272	10.1	11.49	12.29	14.6	16.15	19.44	23.31	25.66	30.09	31.98	35.85	-0.32	0.415	5.83	6.44	6.83	8.15	9.27	12.53	17.48	20.63	26.61	29.19	34.54

Supplementary Table 28. Smoothed percentiles for right arm fat mass for female and male 5 to 18 years.

DXA right arm fat mass (g)

Age (years)	Female LMS parameters and percentile													Male LMS parameters and percentile												
	L	S	1th	3th	5th	15th	25th	50th (M)	75th	85th	95th	97th	99th	L	S	1th	3th	5th	15th	25th	50th (M)	75th	85th	95th	97th	99th
5	-1.008	0.189	239.6	254.5	263.1	288.4	305.9	344.8	395.2	428.9	500.7	535.5	616.5	-0.715	0.272	184.4	193.7	199.7	220.4	238.4	291.5	368.6	413.6	490.2	520.3	578.4
5.5	-0.925	0.207	241.2	257.6	267.1	295.3	314.9	359.0	416.8	455.9	540.0	581.3	678.3	-0.695	0.25	197.2	206.9	213.0	233.9	251.8	302.9	374.6	415.4	483.5	509.8	560.0
6	-0.849	0.225	243.2	261.3	271.9	303.2	325.2	375.0	441.2	486.3	584.7	633.4	749.1	-0.678	0.244	212.7	223.2	229.9	252.3	271.3	325.2	399.7	441.8	512.2	539.4	591.3
6.5	-0.779	0.244	245.3	265.1	276.8	311.6	336.2	392.5	468.2	520.3	635.4	692.9	830.9	-0.664	0.251	228.2	239.9	247.3	272.4	293.6	353.4	436.6	484.2	564.5	596.0	656.4
7	-0.714	0.264	247.1	268.8	281.7	320.2	347.7	411.1	497.4	557.4	691.3	758.9	922.4	-0.651	0.268	241.7	255.1	263.5	292.0	316.1	384.7	481.8	538.4	636.1	675.1	751.1
7.5	-0.653	0.283	249.6	273.3	287.4	329.9	360.4	431.4	528.9	597.3	751.2	829.4	1019.6	-0.64	0.289	253.2	268.3	277.8	310.2	337.7	416.9	531.8	600.5	722.3	772.1	871.0
8	-0.597	0.299	254.0	279.8	295.2	341.9	375.5	454.3	563.1	639.9	812.9	901.0	1115.4	-0.631	0.31	263.4	280.2	290.8	327.1	358.1	448.3	582.5	664.8	814.6	877.2	1004.3
8.5	-0.544	0.311	261.3	289.4	306.2	357.3	394.3	480.8	600.7	685.2	875.4	971.8	1205.1	-0.622	0.329	272.7	291.2	302.9	342.9	377.2	478.1	631.5	727.6	906.9	983.4	1141.6
9	-0.494	0.319	272.1	302.8	321.1	376.9	417.3	511.8	642.4	734.0	938.7	1041.6	1288.1	-0.615	0.344	281.4	301.3	314.0	357.4	394.8	505.4	676.3	785.4	993.2	1083.5	1273.2
9.5	-0.446	0.323	286.2	319.6	339.6	400.4	444.3	546.9	687.7	785.8	1002.5	1110.3	1365.0	-0.608	0.355	289.8	311.0	324.5	370.9	410.9	529.8	715.7	835.8	1068.2	1170.6	1388.1
10	-0.402	0.324	302.7	339.1	360.8	426.8	474.3	584.8	735.1	838.8	1065.2	1176.3	1435.0	-0.602	0.362	298.0	320.4	334.6	383.5	425.6	551.2	748.7	877.4	1128.7	1240.5	1479.7
10.5	-0.359	0.323	320.8	360.1	383.6	454.8	505.8	623.7	782.5	890.9	1124.2	1237.2	1496.4	-0.597	0.366	305.6	328.9	343.8	394.7	438.5	569.2	775.1	909.9	1174.6	1292.9	1547.2
11	-0.318	0.32	340.1	382.5	407.8	484.0	538.4	663.4	829.5	941.7	1179.9	1293.6	1550.9	-0.592	0.367	312.1	336.2	351.6	404.1	449.3	583.6	795.4	934.2	1207.7	1330.3	1594.4
11.5	-0.279	0.316	361.1	406.7	433.7	515.1	572.8	704.5	877.4	992.9	1234.6	1348.4	1602.7	-0.587	0.368	317.3	342.0	357.7	411.5	457.7	594.6	810.3	951.8	1230.8	1356.1	1626.4
12	-0.242	0.311	383.6	432.4	461.3	547.7	608.8	747.0	926.3	1044.6	1289.1	1402.8	1653.7	-0.583	0.368	321.3	346.6	362.6	417.3	464.2	603.0	821.2	964.2	1246.7	1373.5	1647.4
12.5	-0.206	0.306	407.1	459.1	489.8	581.4	645.8	790.2	975.4	1096.3	1343.1	1456.5	1704.0	-0.579	0.367	324.7	350.3	366.6	422.1	469.6	609.7	829.3	973.3	1257.5	1385.2	1660.9
13	-0.172	0.3	430.5	485.8	518.3	614.9	682.4	832.6	1023.2	1146.4	1394.9	1507.9	1752.0	-0.576	0.366	327.6	353.6	370.1	426.2	474.1	615.2	835.8	980.2	1265.3	1393.3	1669.7
13.5	-0.139	0.295	453.2	511.5	545.8	647.1	717.4	873.1	1068.3	1193.4	1443.0	1555.5	1796.1	-0.572	0.365	330.4	356.7	373.4	430.1	478.4	620.2	841.4	986.0	1271.4	1399.5	1675.9
14	-0.107	0.29	474.3	535.5	571.5	677.1	750.0	910.4	1109.7	1236.1	1486.3	1598.0	1835.2	-0.569	0.364	333.2	359.9	376.7	433.9	482.6	625.1	846.8	991.5	1276.9	1404.9	1680.9
14.5	-0.076	0.286	493.3	557.2	594.6	704.1	779.4	944.0	1146.4	1274.0	1524.1	1634.8	1868.4	-0.566	0.363	336.2	363.1	380.2	437.9	487.0	630.1	852.3	997.1	1282.4	1410.3	1685.9
15	-0.047	0.282	509.7	576.1	614.8	727.8	805.2	973.2	1178.2	1306.4	1555.9	1665.7	1895.6	-0.564	0.362	339.3	366.5	383.7	442.0	491.4	635.3	858.0	1002.9	1288.3	1416.1	1691.3
15.5	-0.018	0.279	523.3	591.8	631.7	747.8	826.8	997.7	1204.6	1333.1	1581.5	1690.1	1916.2	-0.561	0.36	342.4	369.9	387.3	446.1	495.9	640.6	863.9	1009.1	1294.8	1422.6	1697.7
16	0.01	0.276	533.9	604.2	645.1	763.6	844.1	1017.1	1225.2	1353.6	1600.3	1707.5	1929.7	-0.559	0.359	345.5	373.4	391.0	450.3	500.5	646.0	870.1	1015.7	1301.8	1429.8	1705.1
16.5	0.037	0.274	541.3	613.1	654.8	775.3	856.8	1031.3	1239.7	1367.7	1612.1	1717.7	1935.7	-0.557	0.359	348.7	376.8	394.6	454.5	505.1	651.5	876.5	1022.6	1309.5	1437.8	1713.5
17	0.063	0.272	545.6	618.6	660.9	782.8	865.0	1040.2	1248.2	1375.3	1616.7	1720.5	1933.9	-0.554	0.358	351.7	380.2	398.1	458.7	509.7	657.0	883.1	1029.7	1317.6	1446.3	1722.9
17.5	0.089	0.27	547.1	621.0	663.7	786.5	869.0	1044.2	1251.0	1376.7	1614.4	1716.1	1924.5	-0.552	0.357	354.7	383.4	401.6	462.7	514.1	662.4	889.6	1036.9	1326.1	1455.3	1732.9
18	0.113	0.268	546.3	620.8	663.8	787.0	869.5	1044.1	1249.0	1373.1	1606.4	1705.9	1908.9	-0.551	0.357	357.5	386.6	404.9	466.5	518.4	667.7	896.1	1044.2	1334.8	1464.7	1743.6

Supplementary Table 29. Smoothed percentiles for right leg fat mass for female and male 5 to 18 years.

DXA right leg fat mass (g)

Age (years)	Female LMS parameters and percentile													Male LMS parameters and percentile												
	L	S	1th	3th	5th	15th	25th	50th (M)	75th	85th	95th	97th	99th	L	S	1th	3th	5th	15th	25th	50th (M)	75th	85th	95th	97th	99th
5	-0.917	0.221	640.5	686.3	713.2	792.8	848.7	976.0	1145.6	1262.0	1518.5	1646.9	1955.8	-0.301	0.264	527.7	566.2	589.8	665.7	725.5	881.1	1083.0	1196.7	1390.9	1467.6	1615.9
5.5	-0.857	0.235	664.0	714.7	744.5	833.2	895.9	1039.3	1232.1	1365.4	1661.2	1810.2	2171.2	-0.299	0.279	518.8	559.4	584.4	664.4	727.5	891.8	1107.9	1231.6	1447.0	1533.5	1702.7
6	-0.803	0.249	688.4	744.2	777.2	875.7	945.7	1106.8	1325.1	1477.1	1816.7	1988.8	2408.1	-0.297	0.293	531.4	575.8	603.0	690.6	759.6	939.7	1179.4	1319.0	1566.4	1667.2	1866.8
6.5	-0.754	0.263	712.3	773.6	810.0	919.1	996.9	1177.2	1423.6	1596.2	1984.8	2182.9	2668.2	-0.295	0.306	562.1	611.8	642.4	740.7	818.3	1021.1	1294.1	1455.6	1746.8	1867.3	2108.4
7	-0.707	0.276	736.7	804.0	843.9	964.3	1050.7	1251.8	1529.0	1724.4	2167.1	2394.1	2952.7	-0.293	0.319	606.1	662.6	697.5	809.5	897.9	1129.5	1444.6	1633.8	1980.4	2125.7	2419.9
7.5	-0.665	0.29	764.4	838.0	881.9	1014.6	1110.1	1334.0	1644.5	1864.5	2365.5	2623.4	3259.9	-0.291	0.331	657.0	721.3	760.9	888.6	989.2	1253.6	1616.7	1837.8	2248.8	2423.5	2780.7
8	-0.624	0.301	797.4	877.9	926.0	1072.0	1177.5	1425.4	1770.9	2016.5	2576.9	2865.7	3578.3	-0.29	0.342	709.6	782.1	826.9	971.0	1084.6	1383.4	1797.3	2052.4	2533.5	2740.3	3167.6
8.5	-0.587	0.31	838.2	926.2	978.9	1139.2	1255.3	1528.7	1910.2	2181.5	2799.9	3117.8	3899.3	-0.288	0.351	760.6	841.3	891.1	1051.7	1178.3	1511.2	1975.9	2265.5	2818.7	3059.2	3560.5
9	-0.551	0.316	888.4	984.7	1042.5	1218.4	1345.9	1646.1	2064.7	2361.6	3035.3	3379.8	4220.9	-0.287	0.359	808.8	897.5	952.3	1128.9	1268.0	1633.9	2147.6	2471.0	3095.9	3370.2	3947.0
9.5	-0.518	0.319	948.0	1053.6	1116.9	1309.6	1449.2	1777.6	2233.7	2555.7	3281.5	3649.8	4540.5	-0.286	0.366	855.6	952.1	1011.8	1204.1	1355.4	1752.8	2313.4	2669.5	3364.6	3672.4	4324.4
10	-0.486	0.321	1015.3	1130.7	1199.9	1410.2	1562.4	1919.3	2412.3	2758.2	3531.0	3919.4	4848.8	-0.285	0.371	903.3	1007.6	1072.2	1279.9	1443.2	1871.3	2477.1	2864.8	3628.1	3968.8	4695.0
10.5	-0.455	0.32	1087.8	1213.3	1288.5	1516.7	1681.4	2066.2	2593.8	2961.2	3773.7	4177.8	5133.4	-0.283	0.374	949.0	1060.9	1130.0	1352.5	1526.9	1983.1	2630.1	3046.6	3873.0	4244.2	5040.1
11	-0.426	0.318	1165.7	1301.6	1382.9	1629.1	1806.2	2217.8	2777.5	3164.0	4009.3	4425.0	5396.3	-0.282	0.377	986.9	1105.3	1178.4	1413.2	1596.9	2075.9	2755.9	3195.9	4074.5	4471.4	5326.5
11.5	-0.399	0.315	1251.2	1398.0	1485.6	1750.2	1939.9	2378.2	2968.6	3372.6	4246.1	4670.8	5651.2	-0.281	0.378	1012.6	1135.8	1211.8	1455.6	1645.9	2140.3	2842.1	3298.3	4214.1	4629.8	5529.2
12	-0.372	0.31	1344.2	1502.4	1596.6	1880.1	2082.5	2547.3	3167.3	3587.6	4485.8	4917.6	5902.9	-0.28	0.379	1025.0	1151.2	1229.1	1478.2	1672.2	2174.2	2886.4	3351.0	4288.3	4715.6	5643.4
12.5	-0.347	0.305	1442.5	1612.2	1713.0	2015.6	2230.5	2721.1	3369.0	3804.2	4723.7	5160.9	6147.8	-0.279	0.379	1027.6	1155.4	1234.2	1485.9	1681.3	2184.9	2898.8	3365.9	4312.3	4745.4	5688.7
13	-0.323	0.3	1540.5	1721.5	1828.8	2149.5	2376.2	2890.7	3563.4	4011.1	4947.2	5387.8	6372.3	-0.278	0.379	1024.2	1152.7	1231.9	1484.4	1679.8	2181.3	2891.3	3357.2	4304.7	4739.6	5689.9
13.5	-0.299	0.295	1633.4	1824.8	1938.0	2275.2	2512.5	3047.7	3740.9	4198.5	5145.6	5587.2	6564.8	-0.277	0.379	1018.7	1147.6	1226.9	1479.4	1674.2	2172.2	2876.1	3339.1	4283.9	4719.0	5672.1
14	-0.276	0.29	1716.8	1917.4	2035.8	2387.2	2633.5	3185.7	3894.7	4358.9	5311.1	5751.3	6717.6	-0.277	0.378	1013.3	1142.5	1222.0	1474.4	1668.5	2162.8	2860.4	3320.3	4261.9	4696.8	5651.7
14.5	-0.255	0.285	1788.2	1996.6	2119.3	2482.5	2735.9	3301.2	4021.1	4489.0	5440.7	5877.3	6828.5	-0.276	0.378	1009.0	1138.6	1218.2	1470.7	1664.4	2155.6	2847.7	3305.1	4244.4	4679.3	5636.9
15	-0.234	0.281	1846.4	2061.3	2187.5	2559.9	2818.8	3393.5	4119.7	4588.6	5535.3	5966.4	6899.6	-0.275	0.378	1006.1	1136.4	1216.3	1469.4	1662.9	2152.0	2840.2	3296.1	4235.2	4671.3	5633.6
15.5	-0.213	0.277	1891.3	2111.1	2240.1	2619.4	2882.1	3462.6	4191.2	4658.9	5596.4	6020.7	6933.7	-0.274	0.378	1004.9	1136.1	1216.5	1470.6	1664.6	2152.8	2839.1	3294.9	4236.8	4675.4	5645.8
16	-0.193	0.274	1923.3	2146.8	2277.6	2661.5	2926.6	3509.8	4237.2	4701.4	5626.5	6042.7	6933.4	-0.273	0.378	1005.5	1137.8	1218.8	1474.8	1669.6	2158.5	2845.2	3302.5	4250.6	4693.5	5675.7
16.5	-0.174	0.271	1943.3	2169.2	2301.3	2687.8	2953.8	3536.9	4259.8	4718.9	5628.4	6035.5	6902.6	-0.273	0.379	1007.2	1140.9	1222.8	1481.1	1677.3	2168.2	2857.4	3317.6	4275.3	4724.0	5722.1
17	-0.156	0.269	1953.2	2180.4	2313.0	2700.3	2966.1	3546.6	4262.4	4714.8	5606.4	6003.6	6845.7	-0.272	0.38	1009.6	1144.9	1227.8	1488.8	1686.7	2180.7	2874.2	3338.7	4309.0	4765.1	5782.8
17.5	-0.138	0.266	1954.6	2182.3	2314.9	2701.5	2966.1	3542.0	4248.4	4692.9	5564.6	5951.2	6767.6	-0.271	0.381	1012.0	1149.0	1232.9	1497.0	1697.0	2194.8	2893.9	3363.8	4349.2	4814.1	5854.5
18	-0.12	0.264	1950.0	2177.3	2309.6	2694.3	2957.0	3526.7	4222.2	4658.0	5508.7	5884.4	6674.7	-0.271	0.383	1013.7	1152.5	1237.5	1504.7	1706.8	2208.8	2914.4	3390.2	4392.4	4866.9	5932.5

Supplementary Table 30. Smoothed percentiles for truncal fat mass for female and male 5 to 18 years.

DXA Truncal fat mass (g)

Age (y)	Female LMS parameters and percentile													Male LMS parameters and percentile												
	L	S	1th	3th	5th	15th	25th	50th (M)	75th	85th	95th	97th	99th	L	S	1th	3th	5th	15th	25th	50th (M)	75th	85th	95th	97th	99th
5	-0.731	0.35	919.6	1017.8	1077.4	1262.7	1401.1	1741.9	2257.0	2654.7	3675.6	4271.8	5990.9	-0.317	0.367	647.5	716.7	759.7	899.6	1011.3	1311.0	1739.4	2010.7	2536.7	2768.4	3257.0
5.5	-0.661	0.359	1004.1	1117.2	1186.0	1400.1	1559.9	1953.1	2543.4	2994.8	4132.4	4781.7	6591.7	-0.298	0.39	716.2	796.5	846.8	1012.0	1145.9	1512.0	2045.7	2386.3	3049.5	3342.3	3961.2
6	-0.598	0.371	1078.3	1206.9	1285.2	1529.5	1712.2	2161.5	2833.8	3344.5	4614.5	5327.3	7265.5	-0.282	0.413	783.2	875.1	932.9	1125.1	1283.1	1723.0	2376.3	2796.8	3618.9	3983.0	4754.0
6.5	-0.54	0.388	1132.3	1276.7	1365.0	1641.5	1849.2	2362.1	3132.0	3716.8	5164.9	5971.8	8139.0	-0.266	0.436	847.9	951.8	1017.7	1238.7	1422.9	1944.8	2734.6	3247.4	4255.1	4702.9	5653.3
7	-0.486	0.412	1163.2	1323.7	1422.4	1733.7	1969.3	2556.2	3446.1	4126.3	5818.3	6762.5	9293.4	-0.252	0.46	910.2	1026.7	1100.9	1352.5	1564.9	2177.5	3122.2	3741.2	4964.3	5509.8	6670.4
7.5	-0.435	0.44	1177.6	1354.2	1463.5	1811.2	2076.8	2745.3	3771.9	4563.5	6546.3	7657.1	10637.4	-0.239	0.482	970.1	1099.3	1182.2	1465.8	1708.1	2418.7	3534.7	4272.6	5738.4	6394.4	7793.5
8	-0.388	0.464	1191.1	1383.9	1504.0	1888.6	2184.8	2936.4	4100.9	5003.2	7267.5	8534.5	11917.3	-0.227	0.503	1028.7	1170.8	1262.4	1578.4	1851.6	2664.9	3963.1	4828.2	6553.8	7328.1	8981.8
8.5	-0.344	0.481	1221.9	1432.5	1564.1	1987.6	2315.2	3148.9	4441.7	5440.8	7928.9	9307.0	12933.7	-0.215	0.522	1087.0	1241.8	1342.0	1690.6	1994.7	2912.5	4397.6	5393.1	7383.8	8277.8	10188.0
9	-0.302	0.488	1281.6	1513.1	1657.9	2124.7	2485.7	3402.5	4813.2	5892.5	8537.0	9975.6	13679.2	-0.204	0.537	1144.6	1311.7	1420.3	1800.6	2135.3	3156.4	4826.9	5951.1	8200.3	9210.0	11365.1
9.5	-0.263	0.488	1373.1	1629.3	1789.6	2305.4	2702.9	3705.2	5226.3	6372.3	9119.7	10581.2	14248.8	-0.194	0.549	1201.3	1380.3	1497.0	1907.7	2271.7	3392.5	5240.6	6486.7	8976.6	10092.1	12467.8
10	-0.226	0.481	1493.9	1778.8	1956.6	2526.5	2962.9	4052.4	5676.2	6877.4	9688.2	11147.9	14717.8	-0.185	0.558	1257.3	1447.4	1571.7	2011.1	2402.8	3617.1	5630.0	6987.0	9690.2	10897.2	13459.3
10.5	-0.19	0.47	1640.4	1957.0	2153.9	2781.3	3258.0	4433.6	6150.2	7395.4	10238.1	11679.8	15120.0	-0.176	0.565	1312.0	1512.5	1643.9	2109.8	2527.0	3827.1	5988.5	7442.7	10326.7	11608.5	14318.0
11	-0.156	0.457	1813.3	2164.6	2382.3	3070.9	3589.1	4850.4	6652.9	7934.9	10792.5	12209.6	15516.0	-0.167	0.569	1364.5	1574.6	1712.4	2202.5	2642.9	4020.4	6312.1	7849.2	10880.1	12220.0	15038.9
11.5	-0.124	0.442	2015.4	2405.0	2645.1	3398.8	3960.3	5308.1	7193.2	8508.3	11374.8	12766.9	15950.5	-0.159	0.571	1415.1	1633.9	1777.6	2289.6	2750.8	4197.1	6601.1	8206.6	11352.1	12734.3	15627.1
12	-0.093	0.426	2248.8	2680.1	2944.4	3767.1	4373.7	5809.2	7775.2	9121.8	11996.4	13366.2	16443.3	-0.151	0.571	1464.7	1691.5	1840.5	2372.4	2852.4	4359.4	6858.6	8518.9	11748.7	13158.7	16093.5
12.5	-0.063	0.411	2507.2	2982.4	3271.9	4165.6	4817.6	6339.7	8383.1	9758.9	12641.0	13991.3	16977.2	-0.143	0.57	1515.1	1749.4	1903.4	2453.5	2950.5	4511.2	7089.9	8792.6	12079.0	13503.9	16452.5
13	-0.034	0.396	2779.8	3299.5	3614.4	4578.2	5274.3	6878.8	8993.2	10394.9	13282.2	14614.7	17521.5	-0.136	0.568	1567.6	1809.0	1967.8	2534.9	3047.6	4656.2	7300.8	9035.1	12354.8	13783.8	16723.0
13.5	-0.007	0.382	3053.9	3616.9	3956.2	4986.7	5724.0	7403.5	9580.1	11003.0	13891.1	15206.6	18042.8	-0.129	0.564	1622.9	1871.4	2034.8	2618.2	3145.5	4797.4	7497.1	9254.4	12589.1	14013.9	16926.6
14	0.02	0.37	3315.9	3919.6	4281.6	5373.3	6147.5	7892.9	10121.2	11560.1	14443.4	15741.8	18513.3	-0.123	0.559	1681.2	1936.7	2104.5	2703.7	3244.9	4936.4	7682.6	9456.3	12792.6	14207.3	17081.9
14.5	0.046	0.359	3553.3	4193.8	4576.2	5721.9	6528.3	8329.2	10598.5	12048.3	14921.1	16202.3	18913.5	-0.116	0.553	1742.1	2004.5	2176.7	2791.2	3345.7	5074.1	7860.3	9645.5	12973.9	14374.7	17204.2
15	0.07	0.351	3756.1	4428.9	4829.0	6021.1	6854.5	8700.7	11000.8	12456.7	15314.3	16578.0	19232.9	-0.11	0.547	1805.4	2074.5	2251.1	2880.5	3447.9	5211.0	8032.3	9825.5	13140.0	14524.9	17305.8
15.5	0.094	0.344	3917.7	4618.0	5033.1	6263.9	7119.5	9001.6	11323.6	12781.9	15620.7	16867.1	19469.5	-0.104	0.541	1870.5	2146.4	2327.3	2971.4	3551.2	5347.4	8200.4	9999.5	13296.8	14664.7	17396.3
16	0.117	0.339	4034.3	4757.1	5184.3	6446.2	7319.1	9228.0	11563.5	13020.2	15836.3	17065.2	19617.4	-0.099	0.534	1937.0	2219.7	2404.8	3063.4	3655.4	5483.7	8366.4	10170.4	13449.6	14800.8	17484.7
16.5	0.14	0.335	4104.9	4845.1	5281.6	6566.6	7451.9	9377.8	11717.4	13168.1	15955.7	17165.7	19667.2	-0.093	0.528	2004.6	2294.0	2483.3	3156.2	3760.4	5620.4	8532.2	10340.9	13603.3	14938.8	17578.3
17	0.162	0.333	4131.7	4884.3	5327.4	6627.9	7520.7	9454.6	11789.2	13229.5	15982.4	17171.8	19620.9	-0.088	0.522	2072.9	2368.9	2562.5	3249.8	3866.1	5757.6	8698.8	10513.0	13761.0	15082.5	17681.8
17.5	0.183	0.332	4120.9	4881.9	5329.2	6639.1	7535.8	9470.6	11793.7	13220.3	15934.8	17102.8	19499.4	-0.082	0.517	2141.6	2444.4	2642.2	3343.9	3972.3	5895.7	8867.1	10687.7	13924.6	15233.9	17797.7
18	0.203	0.332	4079.5	4845.7	5295.5	6610.4	7508.1	9439.0	11746.3	13157.7	15832.1	16978.7	19324.1	-0.077	0.511	2210.7	2520.1	2722.2	3438.3	4079.0	6034.6	9037.5	10865.8	14094.8	15393.8	17926.8

Supplementary Table 31. Smoothed percentiles for android fat mass for female and male 5 to 18 years.

DXA Android fat mass (g)

Age (y)	Female LMS parameters and percentile													Male LMS parameters and percentile												
	L	S	1th	3th	5th	15th	25th	50th (M)	75th	85th	95th	97th	99th	L	S	1th	3th	5th	15th	25th	50th (M)	75th	85th	95th	97th	99th
5	-0.231	0.48	79.8	94.9	104.4	134.6	157.8	215.6	301.9	365.9	515.9	593.9	785.3	0.844	0.401	39.1	69.1	86.7	135.4	166.0	225.4	287.5	321.8	381.0	404.5	449.6
5.5	-0.233	0.488	87.6	104.5	115.0	148.8	174.8	240.1	338.2	411.3	584.1	674.6	897.9	0.292	0.449	55.7	73.5	84.4	117.6	141.3	193.9	259.2	300.0	378.5	412.6	482.8
6	-0.233	0.5	94.5	113.1	124.7	162.2	191.3	264.6	376.0	459.8	659.7	765.3	1028.3	-0.041	0.49	67.3	83.0	92.8	124.1	147.7	205.1	286.0	342.5	465.2	524.5	658.8
6.5	-0.233	0.519	99.6	119.8	132.5	173.9	206.1	288.6	415.7	512.6	747.1	872.8	1190.0	-0.218	0.527	74.2	89.6	99.4	131.2	156.1	219.9	318.3	393.2	574.4	671.3	914.9
7	-0.23	0.543	103.1	124.9	138.7	184.1	219.8	312.4	458.0	570.8	849.0	1000.6	1389.4	-0.31	0.563	80.8	97.0	107.4	141.8	169.3	242.5	363.2	461.5	722.3	875.1	1301.5
7.5	-0.225	0.568	105.9	129.3	144.3	193.8	233.3	336.9	503.1	633.8	962.4	1144.4	1618.6	-0.331	0.598	87.1	105.1	116.7	155.7	187.4	273.7	422.0	547.7	899.9	1117.8	1766.2
8	-0.218	0.59	109.3	134.5	150.6	204.6	248.0	363.2	550.6	699.9	1080.1	1293.1	1854.2	-0.294	0.631	91.9	112.5	125.9	171.2	208.5	311.3	490.6	643.7	1074.4	1340.7	2128.3
8.5	-0.208	0.604	114.7	142.0	159.5	218.5	266.2	393.5	602.4	769.6	1197.8	1438.6	2074.8	-0.24	0.66	95.8	119.4	134.9	187.9	231.9	353.9	566.7	747.1	1244.6	1544.8	2401.8
9	-0.197	0.61	123.0	152.8	172.1	237.0	289.6	430.2	660.8	845.2	1316.0	1579.7	2273.0	-0.195	0.684	99.3	126.0	143.6	204.4	255.3	397.2	644.2	851.9	1414.4	1746.5	2667.6
9.5	-0.183	0.61	134.3	167.4	188.8	260.8	319.0	474.2	727.2	928.2	1436.0	1717.5	2449.5	-0.164	0.703	103.2	132.8	152.4	220.7	278.2	439.2	719.2	953.8	1581.9	1947.9	2945.6
10	-0.166	0.604	148.6	185.6	209.5	289.6	354.2	525.4	801.0	1017.5	1556.1	1850.3	2603.0	-0.15	0.716	108.0	140.1	161.5	236.4	299.7	477.5	788.0	1048.1	1742.9	2146.2	3239.3
10.5	-0.147	0.594	165.6	207.0	233.8	323.1	394.6	582.4	880.1	1110.3	1672.3	1973.7	2730.1	-0.147	0.724	113.1	147.1	169.9	249.7	317.5	508.7	844.2	1126.2	1883.0	2323.8	3521.9
11	-0.126	0.581	185.1	231.7	261.6	361.0	440.0	645.0	963.9	1206.2	1784.9	2089.0	2836.4	-0.152	0.728	117.1	152.4	175.9	258.8	329.2	528.7	880.8	1178.4	1982.9	2454.6	3746.8
11.5	-0.102	0.566	207.7	260.1	293.6	404.2	491.3	714.2	1054.0	1307.2	1898.1	2202.1	2933.5	-0.159	0.73	120.1	155.9	179.8	264.1	335.8	539.3	900.5	1207.5	2044.0	2538.5	3904.9
12	-0.076	0.549	233.3	292.2	329.7	452.5	548.2	789.9	1150.0	1413.2	2013.2	2315.3	3027.6	-0.168	0.729	122.5	158.7	182.8	267.7	339.9	545.3	911.0	1223.3	2080.1	2590.0	4011.1
12.5	-0.048	0.532	261.5	327.5	369.3	505.1	609.9	870.5	1250.3	1522.4	2129.0	2428.3	3120.3	-0.174	0.727	125.0	161.4	185.7	271.1	343.7	550.1	918.4	1233.7	2102.4	2622.0	4078.3
13	-0.019	0.515	291.2	364.8	411.1	560.3	674.2	953.4	1351.4	1631.3	2242.0	2537.7	3209.4	-0.178	0.723	127.9	164.8	189.3	275.6	348.8	556.9	927.8	1245.4	2121.1	2645.6	4118.3
13.5	0.01	0.5	321.4	402.6	453.5	616.1	738.8	1035.5	1449.8	1735.9	2348.3	2639.7	3291.3	-0.179	0.719	131.4	169.1	194.2	282.0	356.5	567.6	942.6	1262.8	2143.0	2668.7	4140.9
14	0.04	0.485	350.7	439.5	494.9	670.3	801.4	1114.0	1541.9	1832.7	2444.1	2730.6	3362.3	-0.176	0.714	135.7	174.6	200.4	290.7	367.1	582.9	964.0	1287.7	2171.2	2695.3	4152.3
14.5	0.07	0.472	378.0	474.2	533.8	721.1	859.7	1186.1	1625.0	1918.7	2526.8	2807.8	3419.9	-0.168	0.709	140.6	181.0	207.8	301.4	380.4	602.4	991.5	1319.4	2205.0	2725.0	4154.3
15	0.1	0.46	402.3	505.3	568.8	766.9	912.1	1250.1	1697.0	1992.1	2594.5	2869.6	3462.4	-0.157	0.703	145.9	188.1	216.1	313.8	395.9	625.6	1024.4	1357.1	2243.8	2757.7	4150.0
15.5	0.129	0.449	422.7	531.9	598.9	806.4	957.2	1304.3	1756.3	2051.3	2645.9	2914.5	3488.2	-0.143	0.698	151.4	195.8	225.3	327.7	413.6	652.4	1062.3	1400.4	2287.6	2794.0	4143.1
16	0.158	0.44	438.8	553.4	623.5	838.9	994.1	1347.8	1802.0	2095.2	2679.7	2941.3	3496.0	-0.126	0.693	157.2	204.0	235.1	342.9	433.1	682.1	1104.5	1448.6	2336.1	2834.4	4138.7
16.5	0.186	0.432	450.2	569.6	642.3	864.0	1022.6	1380.3	1833.8	2123.6	2696.1	2950.2	3485.6	-0.108	0.688	163.0	212.5	245.4	359.2	454.0	714.3	1150.1	1500.6	2389.1	2879.8	4141.4
17	0.215	0.425	457.3	580.7	655.5	882.3	1043.1	1402.6	1852.9	2138.1	2696.7	2943.0	3459.2	-0.088	0.684	168.8	221.2	256.0	376.2	476.0	748.2	1198.2	1555.5	2446.1	2930.1	4153.5
17.5	0.243	0.418	460.2	587.2	663.8	894.3	1056.5	1415.8	1860.7	2140.2	2683.8	2922.3	3419.8	-0.068	0.68	174.6	230.0	266.8	393.8	498.9	783.5	1248.3	1612.8	2506.7	2985.1	4175.5
18	0.271	0.412	459.8	590.0	668.0	901.5	1064.4	1422.0	1860.2	2133.5	2661.8	2892.5	3372.5	-0.049	0.676	180.3	238.9	277.8	411.8	522.3	819.8	1299.8	1671.7	2570.2	3044.2	4206.9

Supplementary Table 32. Smoothed percentiles for gynoid fat mass for female and male 5 to 18 years.

DXA Gynoid fat mass (g)

Age (y)	Female LMS parameters and percentile													Male LMS parameters and percentile												
	L	S	1th	3th	5th	15th	25th	50th (M)	75th	85th	95th	97th	99th	L	S	1th	3th	5th	15th	25th	50th (M)	75th	85th	95th	97th	99th
5	-0.805	0.256	481.9	521.7	545.3	616.1	666.5	783.3	943.1	1055.2	1308.7	1438.7	1759.5	0.597	0.303	297.7	370.1	411.1	524.4	597.0	742.3	900.1	989.7	1148.0	1212.0	1336.5
5.5	-0.736	0.269	499.0	543.1	569.3	647.9	704.2	835.0	1014.5	1140.8	1426.3	1572.5	1932.0	0.431	0.322	294.1	360.2	398.3	506.7	578.3	726.4	893.9	992.0	1170.2	1243.8	1389.9
6	-0.673	0.282	519.0	567.9	596.9	684.6	747.5	894.2	1096.3	1238.7	1560.5	1725.0	2128.1	0.282	0.34	310.2	374.9	412.9	523.4	598.5	758.4	947.0	1061.0	1274.3	1364.8	1548.1
6.5	-0.615	0.296	540.1	594.1	626.4	724.1	794.4	959.1	1187.0	1347.8	1711.4	1897.0	2350.4	0.149	0.356	338.7	405.0	444.3	561.1	642.2	820.2	1038.6	1174.9	1438.4	1553.3	1791.7
7	-0.562	0.31	560.7	620.4	656.2	764.8	843.3	1027.8	1284.2	1465.5	1875.6	2084.7	2594.1	0.036	0.371	374.5	444.0	485.7	611.2	700.2	900.4	1155.3	1319.4	1647.2	1794.3	2107.5
7.5	-0.512	0.324	581.8	647.5	687.0	807.2	894.5	1100.3	1387.1	1590.2	2049.4	2283.1	2849.9	-0.053	0.385	413.9	487.8	532.5	668.7	766.7	992.1	1288.2	1484.3	1888.0	2074.4	2481.2
8	-0.465	0.335	605.1	677.1	720.5	853.0	949.5	1177.4	1495.3	1720.3	2227.5	2484.5	3104.1	-0.117	0.396	454.4	533.7	581.9	730.2	838.3	1090.7	1431.1	1661.7	2149.0	2379.3	2893.6
8.5	-0.421	0.344	633.9	712.9	760.6	906.3	1012.6	1263.6	1613.1	1859.6	2412.1	2690.1	3354.5	-0.16	0.406	494.6	580.0	632.2	793.7	912.4	1193.2	1579.3	1845.6	2420.1	2696.9	3326.6
9	-0.38	0.35	671.2	758.1	810.5	971.1	1088.0	1363.8	1746.1	2014.2	2610.0	2906.9	3609.2	-0.188	0.415	532.4	624.2	680.3	855.2	984.6	1293.5	1724.6	2025.9	2686.2	3009.1	3754.3
9.5	-0.341	0.353	717.9	813.8	871.7	1048.7	1177.4	1479.9	1896.6	2186.6	2824.3	3138.8	3873.6	-0.204	0.423	567.3	665.4	725.5	913.6	1053.5	1389.8	1864.0	2198.6	2940.1	3306.7	4161.4
10	-0.304	0.353	773.3	879.3	943.1	1138.1	1279.5	1610.4	2062.3	2374.0	3051.7	3382.0	4144.4	-0.211	0.429	598.8	703.0	767.1	968.1	1118.1	1480.4	1995.0	2360.4	3176.4	3582.6	4535.8
10.5	-0.268	0.352	836.2	953.0	1023.2	1237.3	1392.0	1752.0	2238.6	2571.1	3285.1	3628.9	4412.7	-0.212	0.434	625.5	735.6	803.3	1016.3	1175.6	1561.7	2112.6	2505.2	3385.6	3825.4	4861.4
11	-0.235	0.348	907.5	1036.0	1113.2	1347.5	1516.1	1905.7	2426.7	2779.0	3526.0	3881.4	4681.7	-0.206	0.438	645.2	760.2	831.1	1054.4	1221.6	1627.7	2208.3	2622.8	3553.2	4018.5	5115.0
11.5	-0.203	0.343	990.2	1131.6	1216.3	1472.5	1655.9	2076.6	2632.6	3004.7	3783.5	4149.6	4964.3	-0.196	0.442	657.2	776.4	849.9	1081.5	1255.2	1677.1	2280.4	2710.7	3675.6	4157.4	5290.5
12	-0.172	0.337	1084.2	1239.7	1332.6	1612.2	1811.2	2264.3	2855.9	3247.6	4057.3	4433.4	5261.4	-0.182	0.445	662.8	785.3	860.9	1099.2	1278.0	1712.2	2331.8	2772.7	3758.0	4248.1	5396.2
12.5	-0.142	0.331	1187.3	1357.6	1459.0	1763.0	1978.0	2463.7	3090.4	3500.8	4339.3	4724.5	5563.7	-0.164	0.448	663.9	789.2	866.6	1110.7	1293.8	1737.6	2369.0	2816.6	3811.7	4304.1	5451.2
13	-0.114	0.324	1293.7	1479.0	1588.9	1916.8	2147.3	2663.9	3322.7	3749.8	4612.6	5004.9	5851.4	-0.144	0.45	662.9	790.9	870.0	1119.4	1306.3	1758.7	2399.3	2851.4	3849.9	4340.8	5476.8
13.5	-0.086	0.317	1398.0	1597.4	1715.4	2065.6	2310.2	2854.5	3540.9	3981.6	4862.7	5259.6	6108.5	-0.122	0.452	661.0	791.8	872.7	1127.6	1318.4	1779.0	2427.9	2883.2	3881.6	4368.9	5488.4
14	-0.06	0.31	1495.2	1707.6	1832.8	2202.8	2459.8	3027.4	3735.8	4186.6	5079.4	5478.0	6324.2	-0.099	0.454	659.1	793.0	875.8	1136.6	1331.5	1800.6	2457.7	2915.8	3912.5	4395.3	5495.8
14.5	-0.035	0.303	1582.0	1805.8	1937.3	2324.2	2591.5	3177.8	3902.4	4359.8	5257.8	5655.7	6494.4	-0.074	0.456	657.7	795.0	879.9	1147.2	1346.7	1825.1	2490.8	2951.9	3946.8	4424.8	5505.9
15	-0.01	0.298	1656.6	1890.1	2026.9	2427.6	2703.1	3303.7	4039.3	4500.2	5398.0	5793.0	6620.4	-0.049	0.457	656.8	798.0	885.2	1159.8	1364.3	1852.9	2528.0	2992.5	3986.0	4459.6	5521.8
15.5	0.014	0.293	1717.9	1959.3	2100.4	2512.2	2793.8	3404.4	4146.1	4607.7	5500.5	5890.8	6704.0	-0.022	0.459	656.5	801.9	891.8	1174.3	1384.3	1884.1	2569.7	3038.0	4031.2	4500.7	5545.6
16	0.037	0.288	1765.7	2013.4	2157.8	2577.6	2863.5	3480.1	4223.5	4683.3	5566.8	5950.9	6747.1	0.004	0.46	656.7	806.7	899.5	1190.7	1406.7	1918.6	2615.7	3088.5	4082.6	4548.7	5578.3
16.5	0.059	0.284	1800.3	2052.6	2199.3	2624.5	2912.9	3531.8	4272.8	4728.5	5599.2	5975.7	6752.7	0.031	0.461	657.3	812.2	908.1	1208.7	1431.1	1956.1	2665.7	3143.5	4139.8	4603.4	5619.7
17	0.08	0.28	1822.7	2078.1	2226.2	2654.3	2943.6	3561.4	4296.5	4746.2	5600.7	5968.5	6724.3	0.057	0.462	658.0	818.3	917.4	1227.9	1457.2	1995.9	2718.8	3202.3	4202.2	4663.9	5669.4
17.5	0.101	0.277	1834.4	2091.4	2240.3	2669.0	2957.7	3571.5	4297.5	4739.4	5575.0	5933.2	6666.3	0.082	0.464	658.9	824.7	927.3	1248.2	1484.5	2037.6	2774.6	3264.2	4269.0	4729.8	5726.8
18	0.122	0.274	1837.8	2095.4	2244.3	2671.8	2958.8	3566.4	4281.0	4714.0	5529.0	5876.8	6586.4	0.106	0.465	659.8	831.3	937.4	1269.0	1512.7	2080.7	2832.2	3328.3	4339.3	4799.9	5790.3

Supplementary Table 33. Smoothed percentiles for total FM% by DXA for female and male 5 to 18 years.

DXA Total body fat percentage (%)

Age (years)	Female LMS parameters and percentile													Male LMS parameters and percentile												
	L	S	1th	3th	5th	15th	25th	50th (M)	75th	85th	95th	97th	99th	L	S	1th	3th	5th	15th	25th	50th (M)	75th	85th	95th	97th	99th
5	-0.936	0.149	20.78	21.87	22.50	24.29	25.49	28.07	31.20	33.18	37.11	38.88	42.73	0.153	0.171	17.52	18.70	19.39	21.37	22.74	25.78	29.15	30.94	33.87	35.00	37.11
5.5	-0.766	0.153	20.83	22.00	22.67	24.57	25.85	28.55	31.81	33.83	37.80	39.57	43.33	0.079	0.179	16.60	17.71	18.35	20.26	21.60	24.64	28.07	29.88	32.86	34.00	36.15
6	-0.61	0.159	20.79	22.05	22.77	24.80	26.15	29.00	32.40	34.49	38.54	40.32	44.07	0.012	0.186	16.36	17.45	18.09	19.99	21.36	24.50	28.10	30.01	33.15	34.35	36.61
6.5	-0.467	0.166	20.64	22.00	22.77	24.94	26.39	29.43	33.01	35.20	39.39	41.21	45.02	-0.044	0.194	16.52	17.61	18.25	20.20	21.62	24.94	28.79	30.85	34.21	35.50	37.93
7	-0.335	0.174	20.39	21.85	22.68	25.02	26.57	29.82	33.62	35.93	40.31	42.21	46.11	-0.09	0.2	16.98	18.10	18.76	20.78	22.28	25.84	30.03	32.26	35.93	37.33	39.97
7.5	-0.211	0.184	20.06	21.64	22.53	25.06	26.72	30.19	34.23	36.66	41.25	43.21	47.24	-0.126	0.207	17.51	18.66	19.34	21.46	23.04	26.86	31.41	33.84	37.83	39.35	42.22
8	-0.096	0.192	19.72	21.42	22.38	25.08	26.86	30.55	34.80	37.35	42.10	44.12	48.23	-0.151	0.213	17.87	19.04	19.74	21.93	23.59	27.64	32.51	35.12	39.39	41.02	44.09
8.5	0.012	0.199	19.42	21.23	22.26	25.13	27.01	30.88	35.31	37.94	42.80	44.84	48.97	-0.167	0.219	18.03	19.21	19.93	22.17	23.88	28.13	33.27	36.03	40.53	42.25	45.48
9	0.114	0.203	19.20	21.12	22.20	25.22	27.18	31.20	35.75	38.42	43.31	45.35	49.43	-0.177	0.225	18.07	19.27	19.99	22.28	24.04	28.44	33.83	36.71	41.42	43.21	46.57
9.5	0.211	0.205	19.07	21.09	22.22	25.36	27.39	31.51	36.10	38.78	43.62	45.63	49.61	-0.182	0.231	18.08	19.29	20.02	22.35	24.15	28.71	34.33	37.33	42.23	44.09	47.57
10	0.303	0.204	19.06	21.16	22.33	25.56	27.63	31.80	36.39	39.03	43.77	45.71	49.54	-0.184	0.236	18.14	19.36	20.10	22.47	24.32	29.05	34.91	38.04	43.13	45.05	48.66
10.5	0.39	0.201	19.15	21.32	22.52	25.82	27.91	32.08	36.61	39.19	43.78	45.64	49.29	-0.183	0.242	18.12	19.35	20.10	22.51	24.41	29.28	35.34	38.59	43.85	45.83	49.53
11	0.473	0.196	19.33	21.55	22.78	26.12	28.22	32.35	36.79	39.30	43.71	45.48	48.94	-0.179	0.247	17.86	19.08	19.83	22.24	24.15	29.10	35.29	38.60	43.94	45.95	49.69
11.5	0.552	0.191	19.57	21.83	23.08	26.44	28.53	32.61	36.94	39.37	43.59	45.29	48.56	-0.174	0.252	17.29	18.48	19.22	21.59	23.49	28.42	34.61	37.92	43.24	45.24	48.95
12	0.628	0.186	19.84	22.14	23.40	26.76	28.84	32.87	37.08	39.42	43.48	45.09	48.19	-0.166	0.256	16.47	17.62	18.33	20.63	22.47	27.31	33.40	36.65	41.85	43.80	47.41
12.5	0.701	0.181	20.12	22.46	23.72	27.09	29.15	33.11	37.22	39.48	43.37	44.91	47.86	-0.156	0.261	15.53	16.62	17.30	19.51	21.28	25.97	31.89	35.04	40.07	41.95	45.42
13	0.772	0.176	20.40	22.77	24.04	27.40	29.45	33.34	37.35	39.54	43.28	44.76	47.57	-0.145	0.265	14.58	15.62	16.26	18.37	20.08	24.59	30.31	33.35	38.19	39.98	43.29
13.5	0.839	0.171	20.68	23.07	24.35	27.70	29.73	33.57	37.48	39.61	43.23	44.64	47.34	-0.132	0.27	13.72	14.70	15.32	17.33	18.98	23.34	28.87	31.80	36.45	38.17	41.33
14	0.904	0.167	20.93	23.35	24.64	27.99	30.01	33.79	37.62	39.69	43.19	44.56	47.15	-0.119	0.274	12.99	13.93	14.52	16.46	18.05	22.28	27.66	30.50	34.99	36.65	39.68
14.5	0.967	0.163	21.17	23.61	24.91	28.26	30.26	34.01	37.76	39.78	43.19	44.51	47.01	-0.105	0.278	12.40	13.31	13.88	15.77	17.32	21.46	26.73	29.51	33.87	35.48	38.41
15	1.027	0.16	21.37	23.85	25.15	28.51	30.51	34.21	37.91	39.89	43.21	44.50	46.92	-0.091	0.282	11.96	12.85	13.41	15.25	16.78	20.87	26.08	28.82	33.10	34.67	37.53
15.5	1.086	0.158	21.55	24.06	25.38	28.75	30.74	34.42	38.06	40.01	43.26	44.51	46.87	-0.078	0.286	11.63	12.51	13.06	14.89	16.40	20.47	25.66	28.38	32.63	34.17	36.98
16	1.143	0.155	21.71	24.25	25.58	28.97	30.96	34.61	38.22	40.13	43.32	44.55	46.85	-0.064	0.289	11.40	12.27	12.82	14.64	16.15	20.24	25.44	28.17	32.39	33.93	36.71
16.5	1.198	0.154	21.84	24.42	25.77	29.17	31.16	34.81	38.38	40.26	43.40	44.60	46.86	-0.051	0.293	11.25	12.12	12.66	14.49	16.01	20.13	25.37	28.12	32.35	33.88	36.65
17	1.251	0.152	21.95	24.57	25.94	29.37	31.36	34.99	38.53	40.40	43.49	44.68	46.89	-0.038	0.296	11.15	12.02	12.56	14.40	15.94	20.11	25.42	28.20	32.46	33.99	36.76
17.5	1.303	0.15	22.05	24.72	26.09	29.55	31.55	35.18	38.69	40.54	43.59	44.76	46.93	-0.025	0.3	11.09	11.97	12.52	14.37	15.93	20.17	25.57	28.38	32.68	34.23	37.01
18	1.353	0.149	22.14	24.85	26.25	29.73	31.73	35.35	38.85	40.68	43.69	44.84	46.98	-0.013	0.303	11.07	11.95	12.51	14.39	15.97	20.29	25.80	28.65	33.01	34.57	37.38

Supplementary Table 34. Smoothed percentiles for truncal fat percentage by DXA for female and male 5 to 18 years.

DXA Truncal fat percentage (%)

Age (years)	Female LMS parameters and percentile													Male LMS parameters and percentile												
	L	S	1th	3th	5th	15th	25th	50th (M)	75th	85th	95th	97th	99th	L	S	1th	3th	5th	15th	25th	50th (M)	75th	85th	95th	97th	99th
5	-0.15	0.246	14.07	15.57	16.45	18.97	20.67	24.35	28.80	31.57	36.95	39.31	44.27	0.098	0.28	11.04	12.37	13.15	15.50	17.17	21.01	25.60	28.22	32.87	34.75	38.46
5.5	-0.084	0.245	14.38	15.97	16.88	19.52	21.30	25.09	29.64	32.44	37.81	40.15	45.00	0.111	0.29	10.36	11.61	12.35	14.60	16.23	20.07	24.68	27.29	31.81	33.62	37.15
6	-0.025	0.248	14.56	16.23	17.20	19.97	21.83	25.79	30.49	33.36	38.84	41.20	46.07	0.122	0.299	10.18	11.40	12.13	14.39	16.06	20.05	24.89	27.58	32.19	34.01	37.52
6.5	0.029	0.256	14.52	16.30	17.33	20.27	22.25	26.45	31.41	34.44	40.18	42.64	47.70	0.133	0.309	10.33	11.57	12.32	14.66	16.42	20.70	25.92	28.79	33.63	35.52	39.12
7	0.078	0.268	14.28	16.18	17.28	20.44	22.56	27.07	32.40	35.65	41.78	44.41	49.80	0.142	0.317	10.70	11.99	12.78	15.25	17.14	21.81	27.52	30.64	35.82	37.83	41.61
7.5	0.124	0.283	13.94	15.96	17.14	20.53	22.81	27.67	33.40	36.89	43.48	46.30	52.06	0.151	0.326	11.13	12.47	13.30	15.92	17.94	23.03	29.29	32.68	38.24	40.36	44.35
8	0.168	0.295	13.61	15.76	17.01	20.62	23.06	28.23	34.35	38.05	45.04	48.02	54.09	0.16	0.333	11.47	12.87	13.72	16.47	18.62	24.10	30.88	34.51	40.41	42.64	46.80
8.5	0.21	0.303	13.41	15.67	16.99	20.79	23.35	28.78	35.16	39.02	46.25	49.32	55.56	0.168	0.341	11.74	13.17	14.05	16.91	19.17	25.00	32.24	36.10	42.29	44.61	48.90
9	0.253	0.306	13.38	15.75	17.12	21.06	23.71	29.30	35.82	39.74	47.03	50.11	56.34	0.176	0.347	11.96	13.41	14.32	17.27	19.64	25.79	33.47	37.52	43.98	46.37	50.78
9.5	0.295	0.302	13.54	15.99	17.40	21.45	24.15	29.80	36.33	40.22	47.41	50.42	56.48	0.183	0.353	12.13	13.61	14.53	17.56	20.01	26.47	34.54	38.78	45.46	47.91	52.41
10	0.34	0.295	13.87	16.38	17.83	21.93	24.65	30.29	36.71	40.51	47.45	50.34	56.11	0.189	0.359	12.24	13.73	14.67	17.76	20.29	27.01	35.42	39.81	46.68	49.18	53.74
10.5	0.386	0.284	14.33	16.90	18.36	22.49	25.20	30.75	37.00	40.65	47.27	50.00	55.43	0.196	0.364	12.23	13.72	14.66	17.79	20.36	27.26	35.92	40.42	47.38	49.91	54.47
11	0.434	0.272	14.88	17.49	18.96	23.09	25.77	31.20	37.24	40.72	46.99	49.55	54.61	0.202	0.369	12.04	13.51	14.43	17.53	20.11	27.07	35.82	40.35	47.30	49.80	54.29
11.5	0.485	0.26	15.45	18.10	19.58	23.70	26.34	31.64	37.45	40.77	46.69	49.10	53.81	0.208	0.373	11.67	13.09	13.99	17.02	19.56	26.46	35.15	39.63	46.44	48.87	53.23
12	0.537	0.249	16.03	18.70	20.20	24.29	26.89	32.07	37.66	40.83	46.43	48.69	53.09	0.213	0.376	11.19	12.54	13.41	16.33	18.80	25.54	34.06	38.42	45.01	47.35	51.51
12.5	0.591	0.239	16.58	19.29	20.79	24.86	27.43	32.48	37.88	40.91	46.22	48.35	52.49	0.219	0.38	10.67	11.96	12.78	15.58	17.96	24.50	32.78	37.00	43.33	45.56	49.51
13	0.646	0.229	17.10	19.83	21.34	25.40	27.94	32.88	38.10	41.02	46.08	48.11	52.00	0.224	0.383	10.17	11.39	12.17	14.86	17.15	23.49	31.50	35.58	41.64	43.76	47.51
13.5	0.701	0.221	17.57	20.34	21.85	25.91	28.42	33.27	38.35	41.16	46.01	47.94	51.64	0.229	0.385	9.74	10.90	11.65	14.22	16.44	22.58	30.36	34.30	40.12	42.15	45.70
14	0.757	0.214	17.99	20.80	22.33	26.38	28.87	33.65	38.60	41.33	46.00	47.85	51.38	0.234	0.387	9.38	10.50	11.22	13.70	15.85	21.83	29.42	33.24	38.85	40.79	44.19
14.5	0.813	0.209	18.35	21.21	22.75	26.82	29.30	34.02	38.87	41.52	46.05	47.83	51.22	0.238	0.388	9.11	10.19	10.88	13.29	15.39	21.25	28.68	32.41	37.85	39.72	42.98
15	0.869	0.204	18.67	21.58	23.14	27.23	29.70	34.39	39.15	41.74	46.15	47.87	51.14	0.243	0.39	8.91	9.96	10.63	12.98	15.04	20.82	28.14	31.80	37.10	38.92	42.06
15.5	0.924	0.2	18.93	21.90	23.48	27.61	30.08	34.74	39.44	41.99	46.29	47.96	51.14	0.247	0.391	8.78	9.80	10.46	12.77	14.81	20.53	27.78	31.39	36.59	38.36	41.41
16	0.978	0.197	19.14	22.17	23.79	27.95	30.44	35.09	39.74	42.25	46.47	48.11	51.20	0.251	0.391	8.70	9.70	10.35	12.64	14.66	20.36	27.57	31.16	36.28	38.02	41.00
16.5	1.032	0.194	19.29	22.41	24.05	28.27	30.78	35.42	40.05	42.53	46.68	48.29	51.32	0.255	0.392	8.68	9.67	10.31	12.58	14.60	20.30	27.51	31.09	36.16	37.87	40.80
17	1.085	0.192	19.41	22.61	24.29	28.57	31.10	35.76	40.36	42.82	46.92	48.50	51.47	0.259	0.392	8.69	9.68	10.32	12.59	14.61	20.34	27.58	31.15	36.21	37.90	40.79
17.5	1.137	0.19	19.48	22.78	24.50	28.85	31.40	36.08	40.68	43.11	47.17	48.73	51.65	0.263	0.392	8.75	9.73	10.37	12.65	14.68	20.46	27.76	31.36	36.41	38.09	40.96
18	1.188	0.189	19.54	22.93	24.69	29.12	31.70	36.40	40.99	43.41	47.43	48.97	51.85	0.267	0.392	8.84	9.82	10.46	12.76	14.82	20.67	28.06	31.69	36.76	38.44	41.30

Supplementary Table 35. Smoothed percentiles for total lean mass for female and male 5 to 18 years.

DXA Total Lean Mass (kg)

Age	Female LMS parameters modeled percentile													Male LMS parameters modeled percentile												
	L	S	1th	3th	5th	15th	25th	50th (M)	75th	85th	95th	97th	99th	L	S	1th	3th	5th	15th	25th	50th (M)	75th	85th	95th	97th	99th
5	-1.136	0.120	9.09	9.49	9.71	10.35	10.77	11.67	12.75	13.42	14.74	15.33	16.59	1.648	0.122	8.88	9.78	10.24	11.35	11.96	13.03	14.07	14.63	15.59	15.96	16.67
5.5	-1.064	0.114	9.93	10.34	10.57	11.22	11.64	12.54	13.58	14.22	15.45	15.99	17.12	1.161	0.122	9.79	10.57	10.98	12.02	12.62	13.73	14.83	15.44	16.49	16.91	17.70
6	-0.999	0.112	10.62	11.04	11.28	11.95	12.39	13.29	14.34	14.98	16.19	16.71	17.79	0.691	0.123	10.60	11.31	11.70	12.71	13.33	14.50	15.72	16.40	17.59	18.06	18.98
6.5	-0.938	0.114	11.09	11.54	11.80	12.52	12.99	13.96	15.09	15.77	17.06	17.62	18.78	0.219	0.125	11.38	12.06	12.43	13.43	14.06	15.31	16.65	17.41	18.75	19.30	20.36
7	-0.882	0.120	11.41	11.92	12.21	13.01	13.54	14.64	15.92	16.70	18.18	18.82	20.17	-0.220	0.123	12.22	12.85	13.21	14.18	14.81	16.10	17.53	18.35	19.82	20.43	21.63
7.5	-0.830	0.128	11.76	12.32	12.64	13.54	14.13	15.38	16.84	17.73	19.45	20.20	21.77	-0.574	0.121	13.01	13.62	13.96	14.92	15.55	16.87	18.37	19.24	20.81	21.46	22.77
8	-0.781	0.134	12.23	12.84	13.19	14.18	14.83	16.20	17.81	18.80	20.70	21.53	23.28	-0.830	0.122	13.72	14.33	14.67	15.64	16.30	17.69	19.29	20.23	21.93	22.64	24.08
8.5	-0.735	0.139	12.83	13.50	13.87	14.94	15.64	17.11	18.85	19.91	21.96	22.85	24.73	-0.973	0.126	14.34	14.96	15.32	16.35	17.04	18.56	20.33	21.37	23.27	24.07	25.68
9	-0.692	0.145	13.50	14.22	14.63	15.78	16.54	18.14	20.02	21.17	23.39	24.35	26.37	-0.991	0.133	14.90	15.57	15.96	17.07	17.83	19.51	21.49	22.65	24.77	25.66	27.46
9.5	-0.651	0.151	14.17	14.96	15.41	16.68	17.52	19.29	21.38	22.67	25.13	26.22	28.48	-0.888	0.139	15.48	16.21	16.63	17.85	18.69	20.56	22.77	24.05	26.36	27.33	29.27
10	-0.613	0.159	14.82	15.70	16.20	17.62	18.57	20.57	22.94	24.41	27.24	28.49	31.11	-0.695	0.145	16.11	16.92	17.38	18.73	19.67	21.75	24.19	25.59	28.07	29.08	31.10
10.5	-0.576	0.165	15.48	16.46	17.02	18.62	19.68	21.95	24.66	26.34	29.62	31.06	34.12	-0.444	0.152	16.77	17.68	18.20	19.72	20.77	23.11	25.80	27.31	29.94	31.00	33.07
11	-0.541	0.170	16.22	17.31	17.93	19.69	20.88	23.41	26.45	28.34	32.04	33.68	37.16	-0.166	0.158	17.46	18.49	19.09	20.82	22.01	24.64	27.62	29.26	32.04	33.14	35.25
11.5	-0.507	0.170	17.13	18.30	18.97	20.88	22.17	24.90	28.18	30.22	34.20	35.97	39.70	0.107	0.164	18.22	19.40	20.08	22.06	23.41	26.37	29.67	31.43	34.38	35.52	37.68
12	-0.475	0.167	18.22	19.46	20.17	22.18	23.52	26.36	29.75	31.84	35.90	37.68	41.43	0.348	0.167	19.12	20.48	21.25	23.48	25.00	28.31	31.92	33.81	36.91	38.09	40.31
12.5	-0.444	0.161	19.43	20.71	21.44	23.49	24.86	27.73	31.11	33.17	37.13	38.85	42.44	0.540	0.168	20.30	21.81	22.68	25.15	26.83	30.45	34.34	36.34	39.57	40.78	43.02
13	-0.414	0.154	20.67	21.97	22.70	24.76	26.12	28.95	32.25	34.24	38.01	39.63	42.98	0.672	0.165	21.81	23.45	24.39	27.05	28.86	32.74	36.85	38.94	42.25	43.48	45.75
13.5	-0.386	0.147	21.84	23.14	23.88	25.92	27.26	30.03	33.20	35.10	38.66	40.18	43.29	0.741	0.160	23.63	25.36	26.34	29.14	31.03	35.09	39.36	41.50	44.88	46.12	48.40
14	-0.358	0.140	22.89	24.18	24.91	26.94	28.25	30.94	34.01	35.82	39.19	40.62	43.51	0.754	0.154	25.66	27.42	28.43	31.29	33.23	37.39	41.75	43.92	47.33	48.58	50.85
14.5	-0.332	0.134	23.76	25.06	25.78	27.78	29.08	31.71	34.68	36.43	39.65	41.01	43.74	0.722	0.147	27.70	29.46	30.46	33.33	35.27	39.47	43.87	46.05	49.45	50.70	52.96
15	-0.306	0.130	24.45	25.75	26.47	28.46	29.74	32.34	35.25	36.96	40.08	41.39	44.01	0.657	0.140	29.60	31.32	32.30	35.13	37.06	41.24	45.63	47.81	51.19	52.42	54.66
15.5	-0.282	0.127	24.96	26.26	26.99	28.98	30.26	32.85	35.73	37.41	40.48	41.76	44.33	0.569	0.134	31.24	32.91	33.87	36.63	38.54	42.68	47.05	49.22	52.57	53.79	56.01
16	-0.258	0.125	25.31	26.62	27.35	29.36	30.65	33.25	36.13	37.82	40.88	42.15	44.69	0.467	0.130	32.56	34.19	35.12	37.82	39.69	43.80	48.16	50.31	53.65	54.87	57.07
16.5	-0.234	0.124	25.50	26.84	27.58	29.62	30.92	33.55	36.46	38.16	41.24	42.52	45.07	0.354	0.127	33.60	35.18	36.09	38.74	40.59	44.68	49.04	51.20	54.54	55.76	57.97
17	-0.212	0.124	25.59	26.95	27.70	29.77	31.10	33.76	36.72	38.44	41.55	42.84	45.42	0.235	0.126	34.40	35.94	36.83	39.45	41.29	45.39	49.79	51.97	55.36	56.60	58.84
17.5	-0.190	0.124	25.59	26.97	27.74	29.84	31.19	33.90	36.90	38.64	41.80	43.11	45.72	0.110	0.126	35.00	36.52	37.40	40.01	41.84	45.99	50.47	52.71	56.18	57.44	59.75
18	-0.169	0.125	25.53	26.93	27.71	29.85	31.21	33.96	37.00	38.76	41.96	43.29	45.93	-0.020	0.127	35.48	36.98	37.85	40.45	42.31	46.52	51.13	53.43	57.02	58.33	60.72

Supplementary Table 36. Smoothed percentiles for lean mass index (LMI) for female and male 5 to 18 years.

DXA Lean mass index (kg/m2)

Age (years)	Female LMS parameters and percentile													Male LMS parameters and percentile												
	L	S	1th	3th	5th	15th	25th	50th (M)	75th	85th	95th	97th	99th	L	S	1th	3th	5th	15th	25th	50th (M)	75th	85th	95th	97th	99th
5	-2.364	0.061	9.08	9.21	9.29	9.52	9.70	10.14	10.65	10.91	11.33	11.48	11.76	-1.926	0.058	9.58	9.79	9.90	10.20	10.40	10.80	11.24	11.50	11.99	12.19	12.60
5.5	-2.27	0.062	9.27	9.40	9.48	9.73	9.92	10.38	10.91	11.19	11.62	11.78	12.08	-1.773	0.06	9.65	9.86	9.98	10.31	10.52	10.94	11.41	11.69	12.20	12.42	12.86
6	-2.185	0.064	9.37	9.51	9.60	9.86	10.05	10.53	11.09	11.38	11.84	12.01	12.33	-1.634	0.063	9.69	9.91	10.04	10.38	10.60	11.05	11.55	11.84	12.39	12.61	13.08
6.5	-2.107	0.067	9.36	9.51	9.61	9.88	10.09	10.59	11.17	11.48	11.97	12.16	12.51	-1.506	0.066	9.68	9.92	10.05	10.41	10.64	11.11	11.63	11.94	12.51	12.75	13.23
7	-2.035	0.071	9.32	9.48	9.58	9.87	10.09	10.62	11.25	11.57	12.11	12.31	12.70	-1.387	0.069	9.64	9.89	10.03	10.40	10.65	11.13	11.68	12.00	12.59	12.83	13.33
7.5	-1.968	0.076	9.26	9.44	9.54	9.85	10.09	10.65	11.32	11.67	12.26	12.49	12.92	-1.276	0.071	9.59	9.85	9.99	10.38	10.63	11.14	11.70	12.03	12.64	12.89	13.41
8	-1.904	0.081	9.21	9.39	9.50	9.83	10.08	10.67	11.38	11.77	12.40	12.65	13.13	-1.173	0.073	9.56	9.83	9.98	10.39	10.65	11.17	11.76	12.10	12.73	12.99	13.52
8.5	-1.845	0.085	9.17	9.36	9.48	9.83	10.09	10.72	11.46	11.87	12.56	12.83	13.34	-1.076	0.076	9.57	9.85	10.01	10.43	10.71	11.25	11.86	12.21	12.86	13.13	13.68
9	-1.789	0.089	9.17	9.37	9.49	9.86	10.14	10.79	11.58	12.01	12.75	13.04	13.60	-0.984	0.078	9.61	9.91	10.07	10.51	10.79	11.36	11.99	12.36	13.03	13.32	13.88
9.5	-1.737	0.093	9.19	9.41	9.54	9.93	10.22	10.90	11.73	12.19	12.98	13.29	13.90	-0.897	0.08	9.67	9.98	10.15	10.61	10.90	11.49	12.15	12.53	13.23	13.52	14.10
10	-1.686	0.097	9.24	9.47	9.61	10.02	10.32	11.04	11.92	12.41	13.25	13.59	14.25	-0.815	0.082	9.75	10.07	10.25	10.73	11.04	11.65	12.34	12.73	13.45	13.75	14.36
10.5	-1.639	0.1	9.32	9.56	9.70	10.13	10.45	11.21	12.13	12.65	13.55	13.91	14.63	-0.737	0.085	9.86	10.19	10.38	10.88	11.20	11.85	12.56	12.97	13.72	14.03	14.65
11	-1.593	0.103	9.42	9.68	9.83	10.28	10.61	11.40	12.37	12.91	13.86	14.25	15.02	-0.662	0.087	9.99	10.34	10.54	11.07	11.40	12.07	12.82	13.24	14.02	14.35	14.99
11.5	-1.55	0.105	9.58	9.84	10.00	10.47	10.81	11.63	12.63	13.19	14.18	14.58	15.38	-0.591	0.089	10.16	10.53	10.73	11.28	11.64	12.34	13.11	13.56	14.37	14.71	15.37
12	-1.508	0.105	9.77	10.05	10.21	10.69	11.05	11.88	12.89	13.47	14.47	14.89	15.70	-0.523	0.091	10.35	10.73	10.95	11.53	11.90	12.64	13.45	13.92	14.76	15.11	15.80
12.5	-1.469	0.104	9.99	10.27	10.44	10.93	11.29	12.14	13.15	13.73	14.74	15.15	15.97	-0.458	0.093	10.56	10.97	11.19	11.81	12.19	12.97	13.82	14.30	15.18	15.55	16.27
13	-1.43	0.102	10.20	10.49	10.66	11.17	11.53	12.38	13.39	13.96	14.96	15.37	16.17	-0.395	0.094	10.79	11.22	11.46	12.11	12.51	13.33	14.21	14.72	15.64	16.02	16.77
13.5	-1.393	0.1	10.41	10.71	10.88	11.39	11.75	12.60	13.60	14.16	15.15	15.55	16.34	-0.334	0.096	11.04	11.49	11.74	12.42	12.85	13.70	14.63	15.17	16.13	16.52	17.30
14	-1.358	0.098	10.60	10.90	11.07	11.59	11.95	12.79	13.78	14.33	15.30	15.70	16.47	-0.276	0.098	11.29	11.76	12.03	12.74	13.19	14.09	15.06	15.62	16.61	17.02	17.83
14.5	-1.324	0.096	10.76	11.06	11.24	11.76	12.12	12.95	13.93	14.48	15.44	15.82	16.58	-0.22	0.1	11.52	12.02	12.29	13.04	13.51	14.45	15.46	16.04	17.08	17.51	18.34
15	-1.29	0.095	10.89	11.20	11.38	11.90	12.26	13.09	14.07	14.61	15.55	15.93	16.68	-0.165	0.102	11.72	12.24	12.53	13.31	13.80	14.78	15.83	16.44	17.51	17.95	18.81
15.5	-1.258	0.094	10.99	11.31	11.49	12.01	12.38	13.21	14.18	14.72	15.66	16.03	16.78	-0.113	0.103	11.88	12.43	12.73	13.55	14.06	15.07	16.16	16.79	17.89	18.35	19.23
16	-1.227	0.093	11.07	11.39	11.57	12.10	12.47	13.30	14.27	14.81	15.74	16.12	16.87	-0.062	0.105	12.02	12.59	12.90	13.74	14.28	15.32	16.45	17.09	18.23	18.70	19.61
16.5	-1.197	0.093	11.12	11.44	11.63	12.16	12.54	13.37	14.34	14.87	15.81	16.19	16.94	-0.013	0.107	12.13	12.72	13.04	13.92	14.47	15.55	16.71	17.37	18.54	19.01	19.94
17	-1.168	0.093	11.15	11.47	11.66	12.20	12.58	13.41	14.38	14.92	15.86	16.24	16.99	0.035	0.109	12.22	12.83	13.17	14.07	14.63	15.75	16.94	17.62	18.82	19.30	20.25
17.5	-1.14	0.093	11.15	11.48	11.67	12.22	12.59	13.43	14.40	14.94	15.88	16.27	17.01	0.082	0.11	12.30	12.93	13.27	14.21	14.79	15.93	17.16	17.85	19.07	19.57	20.54
18	-1.112	0.094	11.14	11.47	11.66	12.21	12.59	13.43	14.40	14.94	15.88	16.27	17.02	0.127	0.112	12.37	13.02	13.38	14.34	14.94	16.11	17.37	18.08	19.32	19.83	20.81

Supplementary Table 37. Smoothed percentiles for right arm lean mass for female and male 5 to 18 years.

DXA right arm lean mass (g)

Age (years)	Female LMS parameters and percentile													Male LMS parameters and percentile												
	L	S	1th	3th	5th	15th	25th	50th (M)	75th	85th	95th	97th	99th	L	S	1th	3th	5th	15th	25th	50th (M)	75th	85th	95th	97th	99th
5	-0.668	0.195	313.4	335.5	348.3	385.2	410.5	465.5	534.0	578.0	667.2	708.1	797.8	-0.067	0.151	380.5	406.5	421.0	461.0	486.7	538.7	596.8	630.7	692.3	718.0	769.1
5.5	-0.632	0.161	367.8	390.3	403.1	439.5	463.7	515.2	576.8	614.9	689.0	721.8	791.0	-0.032	0.153	400.2	428.1	443.6	486.6	514.1	569.8	631.8	667.9	733.4	760.6	814.7
6	-0.599	0.144	412.5	435.5	448.5	485.0	509.1	559.3	617.9	653.6	721.4	750.8	811.7	0	0.155	430.2	460.9	478.0	525.2	555.4	616.4	684.1	723.4	794.8	824.3	883.0
6.5	-0.569	0.14	441.7	465.9	479.7	518.1	543.4	595.8	656.7	693.5	763.1	793.0	854.9	0.03	0.156	462.1	495.8	514.6	566.2	599.3	666.0	739.8	782.7	860.2	892.2	955.9
7	-0.541	0.147	461.0	487.6	502.6	544.9	572.7	630.6	698.1	739.0	816.7	850.2	919.6	0.057	0.158	490.9	527.5	547.9	604.0	639.9	712.2	792.2	838.6	922.3	956.8	1025.4
7.5	-0.515	0.158	476.1	505.7	522.5	569.9	601.3	667.1	744.6	791.9	882.3	921.6	1003.5	0.083	0.162	515.8	555.5	577.6	638.4	677.3	755.7	842.3	892.4	982.9	1020.3	1094.3
8	-0.49	0.17	491.5	524.2	542.9	595.9	631.1	705.7	794.1	848.6	953.5	999.4	1095.7	0.106	0.166	539.2	582.3	606.3	672.5	714.8	800.1	894.5	949.1	1047.6	1088.2	1168.7
8.5	-0.467	0.18	511.1	547.0	567.6	626.2	665.3	748.5	847.9	909.3	1028.5	1080.9	1191.2	0.129	0.172	563.3	610.3	636.6	709.0	755.3	848.7	952.1	1012.0	1120.0	1164.5	1252.8
9	-0.446	0.188	534.9	574.4	597.1	661.8	705.3	798.0	909.4	978.6	1113.4	1173.0	1298.7	0.15	0.177	589.0	640.5	669.2	748.5	799.3	901.9	1015.5	1081.3	1200.1	1249.0	1346.1
9.5	-0.425	0.198	560.8	604.5	629.7	701.9	750.5	854.9	981.2	1060.2	1214.9	1283.6	1429.4	0.17	0.183	616.0	672.2	703.7	790.4	846.1	958.5	1083.1	1155.3	1285.6	1339.4	1445.9
10	-0.406	0.211	587.6	636.3	664.3	745.3	800.1	918.6	1063.2	1154.4	1334.1	1414.5	1585.9	0.189	0.189	644.7	706.1	740.5	835.4	896.4	1019.6	1156.3	1235.6	1378.7	1437.7	1554.6
10.5	-0.388	0.222	617.1	671.0	702.3	792.7	854.4	988.3	1153.2	1257.7	1465.4	1558.7	1759.0	0.207	0.195	676.1	743.3	781.0	885.1	952.0	1087.5	1237.8	1325.0	1482.5	1547.4	1676.2
11	-0.37	0.229	653.8	712.9	747.3	846.9	915.0	1063.4	1246.8	1363.5	1595.7	1700.5	1925.4	0.224	0.2	712.1	785.8	827.2	941.8	1015.6	1164.9	1330.8	1427.1	1601.0	1672.7	1814.8
11.5	-0.353	0.228	703.0	766.8	803.8	911.0	984.1	1143.1	1338.8	1462.9	1709.1	1819.7	2056.4	0.241	0.206	755.6	836.9	882.5	1009.0	1090.5	1255.6	1439.0	1545.5	1737.8	1817.1	1974.2
12	-0.337	0.22	763.7	831.2	870.2	982.7	1059.0	1223.8	1424.8	1551.0	1799.3	1909.8	2144.7	0.256	0.209	809.9	899.6	950.0	1089.7	1179.8	1362.2	1564.8	1682.4	1894.6	1982.0	2155.2
12.5	-0.322	0.207	831.2	901.3	941.7	1057.3	1135.0	1301.4	1501.5	1625.9	1867.4	1973.8	2197.7	0.271	0.211	877.8	976.7	1032.3	1186.1	1285.1	1485.6	1708.0	1836.8	2069.1	2164.7	2354.0
13	-0.307	0.194	898.6	970.3	1011.4	1128.2	1206.1	1371.2	1567.0	1687.3	1918.2	2018.9	2228.5	0.286	0.21	960.5	1068.9	1129.7	1297.8	1405.9	1624.3	1865.9	2005.6	2257.0	2360.3	2564.4
13.5	-0.293	0.182	960.1	1032.6	1074.0	1190.8	1268.2	1430.7	1621.1	1736.9	1956.8	2051.7	2247.7	0.3	0.206	1056.1	1173.9	1239.9	1421.9	1538.6	1773.7	2033.0	2182.5	2450.9	2560.9	2778.0
14	-0.279	0.172	1011.9	1085.0	1126.4	1243.0	1319.7	1479.7	1665.2	1777.1	1987.9	2078.1	2263.4	0.313	0.202	1160.1	1286.7	1357.4	1552.1	1676.6	1926.5	2200.9	2358.6	2641.0	2756.5	2984.0
14.5	-0.266	0.165	1052.8	1126.3	1167.9	1284.4	1360.7	1519.0	1701.2	1810.5	2015.0	2102.1	2280.0	0.326	0.196	1264.6	1398.8	1473.7	1679.0	1809.9	2071.8	2358.0	2522.1	2815.0	2934.4	3169.3
15	-0.253	0.161	1083.2	1157.4	1199.2	1316.2	1392.6	1550.5	1731.4	1839.4	2040.7	2126.2	2300.2	0.339	0.19	1362.8	1503.3	1581.4	1795.1	1930.9	2201.7	2496.5	2664.9	2964.7	3086.6	3326.0
15.5	-0.241	0.159	1104.4	1179.4	1221.7	1339.8	1416.8	1575.4	1756.8	1864.8	2065.5	2150.6	2323.3	0.351	0.185	1452.1	1597.6	1678.3	1898.5	2038.0	2315.4	2616.2	2787.5	3091.7	3215.2	3457.1
16	-0.229	0.159	1118.4	1194.4	1237.2	1356.7	1434.5	1594.7	1777.4	1886.0	2087.7	2173.0	2346.1	0.363	0.18	1531.0	1680.6	1763.4	1988.8	2131.3	2413.7	2718.9	2892.3	3199.4	3323.9	3567.2
16.5	-0.217	0.159	1127.2	1204.1	1247.5	1368.3	1446.9	1608.6	1792.9	1902.4	2105.3	2191.1	2364.9	0.374	0.177	1599.9	1753.0	1837.6	2067.4	2212.3	2498.9	2807.6	2982.7	3292.0	3417.2	3661.5
17	-0.206	0.159	1133.0	1210.5	1254.1	1375.7	1454.8	1617.3	1802.1	1911.9	2115.0	2200.8	2374.4	0.385	0.173	1660.1	1816.3	1902.5	2136.4	2283.5	2573.8	2885.9	3062.5	3374.0	3499.8	3745.2
17.5	-0.195	0.158	1137.5	1215.0	1258.7	1380.0	1458.9	1620.7	1804.4	1913.2	2114.3	2199.1	2370.4	0.396	0.171	1712.3	1871.4	1959.2	2196.8	2346.1	2640.2	2955.5	3133.6	3447.4	3574.0	3820.6
18	-0.184	0.155	1141.8	1218.8	1262.0	1382.2	1460.1	1619.6	1800.2	1906.9	2103.6	2186.3	2353.2	0.406	0.169	1756.7	1918.6	2007.7	2248.8	2400.0	2697.4	3015.7	3195.3	3511.1	3638.4	3886.1

Supplementary Table 38. Smoothed percentiles for right leg lean mass for female and male 5 to 18 years.

DXA right leg lean mass (g)

Age (years)	Female LMS parameters and percentile													Male LMS parameters and percentile												
	L	S	1th	3th	5th	15th	25th	50th (M)	75th	85th	95th	97th	99th	L	S	1th	3th	5th	15th	25th	50th (M)	75th	85th	95th	97th	99th
5	-0.827	0.178	1150.4	1222.3	1263.8	1383.7	1465.4	1643.4	1865.4	2008.5	2299.7	2434.2	2731.6	-0.835	0.138	1403.6	1474.8	1515.2	1629.4	1705.1	1864.8	2054.3	2171.2	2397.7	2497.6	2708.5
5.5	-0.763	0.16	1303.2	1379.2	1422.7	1546.9	1630.1	1808.1	2023.5	2158.7	2425.1	2544.5	2800.6	-0.724	0.133	1493.9	1569.4	1612.1	1731.9	1810.7	1975.1	2167.1	2283.8	2506.3	2602.9	2803.7
6	-0.704	0.151	1427.9	1508.6	1554.5	1684.6	1771.1	1954	2171.6	2306.1	2566.8	2681.7	2924.3	-0.622	0.132	1607	1689.6	1736.2	1866.4	1951.5	2128	2331.9	2454.7	2686.4	2785.9	2990.9
6.5	-0.651	0.152	1518.5	1606.1	1655.9	1796.9	1890.4	2087.6	2321.4	2465.2	2742.7	2864.5	3120.3	-0.53	0.134	1716.9	1808.2	1859.5	2002.7	2096.1	2288.8	2510.1	2642.5	2890.7	2996.6	3213.5
7	-0.601	0.16	1594	1691.5	1747.1	1904.7	2009.6	2231.5	2495.3	2658.2	2973.3	3111.8	3403.4	-0.444	0.138	1811.8	1913	1969.8	2128.3	2231.4	2443.8	2686.6	2831.5	3101.8	3216.7	3451
7.5	-0.555	0.171	1671.5	1781	1843.6	2021.9	2140.9	2394	2696.8	2884.6	3249.6	3410.7	3751.1	-0.363	0.144	1899.1	2012.3	2076	2253.6	2369.1	2606.9	2878.5	3040.3	3341.4	3469.2	3729.1
8	-0.511	0.179	1762	1884	1953.9	2153.2	2286.7	2571.2	2912.9	3125.4	3539.2	3722.2	4109.3	-0.286	0.153	1990.6	2119	2191.3	2393	2524.4	2795.1	3104.2	3288.3	3630.7	3775.7	4070.6
8.5	-0.47	0.184	1869.8	2004.3	2081.3	2301.4	2448.9	2763.4	3141.2	3376	3833	4034.9	4461.5	-0.211	0.163	2087.5	2233.6	2316	2546.1	2696.2	3005.4	3358.6	3568.8	3959.4	4124.6	4460.1
9	-0.432	0.189	1987.4	2135.6	2220.5	2463.2	2626	2973.3	3390.2	3649.2	4152.8	4375	4843.8	-0.136	0.171	2190.5	2355.8	2449	2709.3	2879	3228.6	3627.2	3864	4302.8	4488	4862.9
9.5	-0.396	0.196	2104.4	2268.5	2362.8	2632.5	2813.7	3200.9	3666.8	3956.5	4520.3	4769.2	5294.4	-0.062	0.177	2305.6	2490.5	2594.8	2885.6	3074.9	3463.7	3905.1	4166.3	4648.1	4850.5	5258.8
10	-0.361	0.205	2219.7	2402	2506.9	2807.9	3010.6	3445	3969.4	4296.3	4933.7	5215.6	5810.8	0.01	0.182	2434.5	2640.5	2756.5	3079.5	3289.1	3718.2	4202.5	4487.7	5010.8	5229.5	5668.7
10.5	-0.329	0.213	2342	2543.5	2659.7	2993.6	3219	3703	4288.7	4654.3	5368.2	5684.1	6351.5	0.079	0.186	2574.8	2804.1	2933.1	3291.1	3522.8	3995.1	4525.1	4835.5	5401.8	5637.4	6108.3
11	-0.297	0.216	2485.9	2706.1	2833.1	3198	3444.4	3973.3	4612.4	5010.9	5787.3	6130.2	6853.1	0.143	0.189	2724.3	2979.2	3122.3	3518.7	3774.4	4293.6	4872.8	5210.3	5822.9	6076.5	6581.4
11.5	-0.267	0.214	2665.4	2902.1	3038.3	3428.9	3691.6	4253.2	4927.7	5345.8	6155.4	6510.9	7256.3	0.201	0.192	2887.9	3170.2	3328.4	3765.6	4046.8	4615.3	5245.9	5611.7	6272.2	6544.5	7084.4
12	-0.239	0.206	2876.7	3126.6	3269.8	3678.6	3951.9	4531.9	5221.1	5644.5	6456.9	6810.5	7546.3	0.251	0.194	3075.4	3386.1	3559.9	4038.8	4345.6	4963.7	5645.3	6038.8	6746	7036.3	7609.9
12.5	-0.211	0.195	3106.7	3365.9	3513.8	3932.9	4211.1	4795.9	5482.1	5899.3	6690.9	7032.2	7736	0.294	0.193	3302.1	3640.6	3829.5	4348.3	4679.4	5343.4	6071.3	6489.5	7237.8	7543.6	8146
13	-0.185	0.183	3337.4	3602	3752.2	4175	4453.4	5033.1	5704.4	6108.2	6866.2	7189.7	7851.5	0.328	0.189	3575.4	3939.7	4142.4	4697	5049.6	5753.3	6520.1	6958.5	7739.5	8057.6	8681.9
13.5	-0.16	0.172	3553.3	3820.4	3971.2	4393	4668.5	5237.3	5888	6275.7	6996.1	7301	7919.7	0.354	0.184	3891.4	4278.4	4493	5078.1	5448.5	6184.3	6981.5	7435.4	8240.5	8567.2	9206.8
14	-0.135	0.162	3744.3	4011.9	4162.4	4581	4852.6	5409	6039	6411.1	7096.9	7385	7966	0.373	0.177	4233.4	4638.7	4862.9	5471.9	5855.8	6615.3	7433.9	7898.1	8718.6	9050.6	9698.9
14.5	-0.112	0.154	3903.1	4170.9	4321	4736.6	5004.7	5550.7	6163.7	6523.4	7181.9	7456.8	8008.6	0.385	0.17	4569.5	4988	5218.8	5843.9	6236.5	7010.7	7841.2	8310.6	9137.9	9471.8	10122.5
15	-0.089	0.149	4025.4	4294.3	4444.6	4859.3	5125.8	5666	6268.7	6620.7	7261.8	7528.3	8061.2	0.393	0.163	4872	5298.4	5533	6166.9	6563.9	7344.3	8178.6	8648.9	9475.7	9808.7	10456.8
15.5	-0.067	0.146	4110.9	4382.4	4533.8	4950.7	5218	5758	6358.1	6707.3	7341.3	7604.1	8128.1	0.397	0.157	5126.8	5557.3	5793.8	6431.3	6829.7	7611.2	8444.2	8912.9	9735.5	10066.3	10709.4
16	-0.046	0.146	4162.2	4437.8	4591.4	5013.7	5284	5829.2	6433.5	6784.4	7420.2	7683.2	8206.9	0.397	0.153	5326.2	5758.4	5995.6	6634	7032.4	7812.6	8642.8	9109.3	9927	10255.6	10893.8
16.5	-0.025	0.147	4184	4465	4621.5	5051.7	5326.9	5881.4	6495.3	6851.4	7495.7	7762	8291.7	0.395	0.15	5474.5	5907.9	6145.6	6784.8	7183.4	7963.3	8792.4	9257.9	10073.5	10401.1	11037.1
17	-0.005	0.149	4184.5	4471.2	4630.9	5069.6	5350.2	5915.5	6540.8	6903.3	7558.7	7829.4	8367.4	0.39	0.148	5580.5	6016.2	6255.1	6897.5	7298	8081.5	8914.2	9381.7	10200.8	10529.7	11168.4
17.5	0.015	0.151	4171.9	4463.5	4626	5072.4	5357.8	5932.5	6567.9	6936.1	7601.3	7875.8	8421.2	0.384	0.148	5653	6093	6334.3	6983.3	7388.1	8180.3	9022.8	9496	10325.3	10658.5	11305.6
18	0.034	0.153	4152.2	4447.8	4612.4	5064.6	5353.7	5935.5	6578.1	6950.2	7621.9	7898.9	8448.8	0.377	0.149	5700.3	6146.4	6391.2	7050.1	7461.4	8267	9124.8	9607	10452.8	10792.9	11453.6

Supplementary Table 39. Smoothed percentiles for trunk lean mass for female and male 5 to 18 years.

DXA Truncal lean mass (g)

Age	Female LMS parameters and percentile													Male LMS parameters and percentile												
	L	S	1th	3th	5th	15th	25th	50th (M)	75th	85th	95th	97th	99th	L	S	1th	3th	5th	15th	25th	50th (M)	75th	85th	95th	97th	99th
5	-0.653	0.124	4172	4374	4487	4804	5010	5435	5924	6217	6766	7001	7484	0.361	0.138	4195	4493	4656	5094	5366	5899	6464	6781	7335	7558	7990
5.5	-0.566	0.107	4587	4785	4895	5199	5394	5790	6233	6493	6970	7170	7573	0.362	0.122	4654	4941	5098	5516	5775	6279	6809	7105	7621	7827	8227
6	-0.487	0.102	4860	5064	5177	5487	5684	6081	6521	6777	7242	7435	7821	0.319	0.111	5140	5422	5576	5987	6240	6733	7250	7539	8042	8243	8632
6.5	-0.414	0.108	5004	5228	5353	5694	5912	6351	6837	7120	7634	7848	8275	0.189	0.105	5563	5841	5993	6400	6652	7143	7664	7955	8466	8671	9069
7	-0.346	0.120	5108	5365	5508	5901	6153	6662	7230	7562	8168	8421	8927	-0.006	0.103	5899	6175	6326	6733	6988	7489	8027	8331	8868	9086	9512
7.5	-0.283	0.133	5228	5522	5686	6140	6432	7026	7691	8082	8798	9098	9700	-0.220	0.105	6146	6425	6580	6999	7263	7790	8365	8694	9284	9526	10004
8	-0.225	0.142	5386	5716	5901	6412	6742	7414	8170	8615	9431	9773	10461	-0.417	0.111	6377	6670	6832	7279	7565	8141	8781	9155	9835	10118	10685
8.5	-0.169	0.148	5600	5963	6166	6728	7090	7829	8659	9147	10042	10416	11168	-0.566	0.119	6612	6926	7102	7590	7906	8552	9285	9719	10525	10866	11558
9	-0.117	0.153	5856	6252	6475	7090	7486	8293	9198	9730	10701	11107	11920	-0.637	0.128	6842	7183	7376	7912	8262	8987	9821	10322	11266	11670	12502
9.5	-0.068	0.159	6124	6563	6809	7490	7929	8822	9824	10411	11484	11931	12827	-0.621	0.135	7077	7449	7659	8247	8633	9435	10364	10926	11989	12447	13393
10	-0.021	0.167	6399	6890	7165	7927	8419	9421	10545	11204	12407	12909	13913	-0.539	0.142	7325	7735	7966	8614	9039	9922	10946	11563	12730	13231	14266
10.5	0.024	0.175	6700	7249	7557	8411	8964	10089	11352	12092	13444	14006	15133	-0.413	0.148	7593	8050	8309	9032	9504	10483	11611	12288	13558	14101	15212
11	0.066	0.180	7075	7681	8022	8967	9577	10819	12210	13024	14507	15124	16355	-0.260	0.156	7882	8403	8697	9516	10050	11149	12404	13151	14540	15128	16322
11.5	0.106	0.179	7580	8235	8602	9616	10269	11593	13066	13925	15481	16125	17407	-0.094	0.164	8203	8805	9143	10083	10691	11936	13340	14168	15689	16326	17607
12	0.145	0.171	8203	8891	9275	10332	11008	12369	13871	14740	16305	16949	18224	0.072	0.171	8581	9280	9671	10751	11445	12852	14418	15329	16983	17668	19031
12.5	0.183	0.161	8892	9597	9989	11060	11741	13101	14587	15439	16963	17586	18815	0.224	0.175	9074	9878	10325	11551	12332	13896	15610	16595	18358	19080	20503
13	0.218	0.150	9571	10281	10673	11739	12412	13745	15188	16010	17469	18062	19225	0.352	0.174	9732	10636	11136	12494	13351	15047	16878	17917	19755	20500	21955
13.5	0.253	0.139	10185	10891	11280	12329	12987	14282	15672	16458	17845	18406	19501	0.444	0.168	10574	11559	12100	13560	14473	16263	18169	19241	21120	21874	23340
14	0.286	0.131	10709	11407	11789	12818	13459	14715	16052	16804	18125	18657	19691	0.497	0.160	11564	12600	13166	14685	15629	17465	19405	20488	22375	23129	24588
14.5	0.318	0.124	11128	11818	12196	13206	13834	15057	16352	17077	18345	18853	19840	0.512	0.151	12597	13649	14222	15755	16702	18541	20473	21548	23416	24161	25599
15	0.349	0.120	11439	12127	12502	13504	14125	15329	16599	17308	18543	19037	19993	0.491	0.141	13568	14605	15171	16678	17610	19415	21309	22363	24192	24922	26329
15.5	0.379	0.118	11651	12344	12721	13727	14348	15551	16815	17519	18743	19231	20175	0.442	0.133	14421	15428	15976	17440	18344	20099	21943	22970	24756	25469	26846
16	0.408	0.117	11781	12487	12871	13892	14522	15741	17017	17727	18959	19450	20397	0.370	0.126	15129	16099	16628	18042	18919	20622	22419	23422	25172	25872	27227
16.5	0.436	0.119	11845	12571	12965	14013	14659	15906	17210	17934	19190	19690	20653	0.281	0.121	15701	16636	17148	18518	19370	21032	22794	23782	25512	26207	27556
17	0.463	0.121	11862	12610	13016	14095	14758	16039	17377	18119	19404	19915	20899	0.180	0.117	16160	17069	17567	18906	19742	21380	23128	24113	25847	26546	27909
17.5	0.489	0.123	11850	12619	13035	14142	14822	16134	17502	18260	19570	20091	21093	0.070	0.116	16529	17419	17908	19229	20057	21689	23445	24440	26202	26917	28316
18	0.515	0.125	11828	12612	13038	14165	14858	16191	17579	18347	19674	20201	21213	-0.046	0.115	16816	17692	18175	19485	20311	21950	23727	24743	26553	27292	28746

Supplementary Table 40. Smoothed percentiles for appendicular lean mass for female and male 5 to 18 years.

DXA Appendicular lean mass (kg)

Age (years)	Female LMS parameters and percentile													Male LMS parameters and percentile												
	L	S	1th	3th	5th	15th	25th	50th (M)	75th	85th	95th	97th	99th	L	S	1th	3th	5th	15th	25th	50th (M)	75th	85th	95th	97th	99th
5	-1.096	0.185	2.95	3.13	3.23	3.53	3.74	4.20	4.80	5.21	6.08	6.51	7.52	-0.742	0.133	3.49	3.67	3.77	4.05	4.23	4.61	5.06	5.34	5.86	6.08	6.56
5.5	-1.01	0.163	3.37	3.55	3.66	3.97	4.18	4.64	5.21	5.59	6.34	6.70	7.48	-0.634	0.133	3.79	3.99	4.10	4.41	4.61	5.03	5.51	5.81	6.36	6.60	7.09
6	-0.931	0.151	3.70	3.90	4.01	4.34	4.55	5.02	5.58	5.94	6.65	6.98	7.67	-0.534	0.134	4.10	4.32	4.44	4.78	5.01	5.47	6.00	6.32	6.91	7.17	7.69
6.5	-0.858	0.149	3.95	4.16	4.28	4.62	4.85	5.35	5.94	6.31	7.04	7.37	8.07	-0.442	0.136	4.38	4.62	4.76	5.14	5.39	5.89	6.47	6.82	7.46	7.74	8.29
7	-0.791	0.156	4.14	4.38	4.51	4.89	5.15	5.69	6.35	6.77	7.58	7.94	8.72	-0.357	0.14	4.62	4.89	5.04	5.46	5.73	6.29	6.93	7.30	8.00	8.30	8.89
7.5	-0.728	0.166	4.33	4.59	4.74	5.18	5.47	6.09	6.84	7.32	8.26	8.68	9.59	-0.278	0.145	4.84	5.14	5.31	5.78	6.08	6.69	7.39	7.81	8.58	8.90	9.55
8	-0.669	0.177	4.53	4.83	5.00	5.48	5.81	6.52	7.39	7.93	9.02	9.51	10.57	-0.205	0.153	5.07	5.40	5.59	6.11	6.45	7.14	7.93	8.39	9.24	9.60	10.33
8.5	-0.614	0.186	4.76	5.09	5.28	5.83	6.20	6.99	7.97	8.59	9.82	10.38	11.58	-0.135	0.16	5.31	5.68	5.90	6.48	6.86	7.64	8.52	9.04	10.00	10.40	11.21
9	-0.562	0.194	5.02	5.39	5.60	6.21	6.62	7.51	8.60	9.30	10.68	11.30	12.64	-0.07	0.168	5.56	5.98	6.22	6.88	7.30	8.17	9.16	9.74	10.80	11.25	12.14
9.5	-0.513	0.202	5.30	5.71	5.94	6.62	7.08	8.07	9.29	10.07	11.61	12.31	13.81	-0.008	0.174	5.84	6.31	6.57	7.30	7.77	8.74	9.83	10.47	11.64	12.12	13.10
10	-0.466	0.21	5.59	6.04	6.30	7.05	7.56	8.67	10.04	10.91	12.64	13.42	15.09	0.051	0.179	6.15	6.67	6.97	7.78	8.30	9.37	10.56	11.26	12.54	13.07	14.14
10.5	-0.422	0.216	5.90	6.40	6.68	7.52	8.08	9.31	10.83	11.79	13.70	14.56	16.40	0.107	0.183	6.50	7.08	7.40	8.30	8.88	10.06	11.37	12.14	13.53	14.11	15.26
11	-0.38	0.219	6.27	6.81	7.13	8.03	8.65	9.99	11.63	12.66	14.71	15.63	17.59	0.16	0.188	6.88	7.52	7.88	8.88	9.52	10.82	12.26	13.10	14.62	15.25	16.49
11.5	-0.339	0.216	6.72	7.31	7.65	8.62	9.27	10.69	12.41	13.49	15.60	16.54	18.53	0.211	0.191	7.31	8.02	8.42	9.52	10.22	11.65	13.23	14.14	15.79	16.47	17.81
12	-0.3	0.207	7.27	7.89	8.24	9.26	9.94	11.40	13.14	14.22	16.32	17.24	19.17	0.26	0.193	7.79	8.58	9.02	10.23	11.00	12.56	14.27	15.26	17.03	17.76	19.20
12.5	-0.263	0.195	7.86	8.50	8.86	9.91	10.60	12.06	13.79	14.85	16.87	17.75	19.57	0.307	0.192	8.37	9.23	9.71	11.03	11.87	13.55	15.39	16.44	18.33	19.10	20.61
13	-0.228	0.183	8.44	9.10	9.47	10.52	11.22	12.67	14.35	15.37	17.30	18.12	19.81	0.352	0.189	9.06	10.00	10.52	11.93	12.83	14.63	16.57	17.68	19.66	20.46	22.03
13.5	-0.193	0.171	8.98	9.65	10.02	11.07	11.76	13.18	14.82	15.79	17.61	18.39	19.96	0.395	0.184	9.85	10.85	11.41	12.92	13.87	15.76	17.79	18.95	20.99	21.81	23.43
14	-0.16	0.161	9.46	10.12	10.50	11.55	12.22	13.62	15.20	16.13	17.86	18.59	20.06	0.436	0.178	10.69	11.76	12.34	13.93	14.93	16.89	18.99	20.18	22.26	23.10	24.73
14.5	-0.128	0.154	9.85	10.52	10.89	11.93	12.61	13.98	15.51	16.42	18.08	18.77	20.16	0.477	0.172	11.51	12.63	13.24	14.89	15.92	17.94	20.08	21.28	23.39	24.23	25.87
15	-0.098	0.149	10.15	10.82	11.20	12.24	12.91	14.27	15.78	16.67	18.28	18.96	20.30	0.515	0.166	12.26	13.42	14.05	15.74	16.79	18.84	21.00	22.21	24.31	25.15	26.77
15.5	-0.068	0.146	10.36	11.04	11.42	12.47	13.15	14.51	16.02	16.90	18.50	19.16	20.48	0.553	0.16	12.90	14.08	14.72	16.44	17.51	19.57	21.73	22.94	25.02	25.85	27.45
16	-0.039	0.146	10.49	11.19	11.58	12.64	13.32	14.70	16.22	17.11	18.71	19.37	20.69	0.589	0.155	13.42	14.62	15.27	17.01	18.08	20.15	22.31	23.50	25.57	26.39	27.96
16.5	-0.011	0.147	10.55	11.27	11.66	12.75	13.44	14.84	16.39	17.29	18.91	19.57	20.90	0.625	0.152	13.84	15.06	15.72	17.48	18.56	20.63	22.78	23.97	26.02	26.83	28.39
17	0.016	0.149	10.56	11.29	11.69	12.80	13.51	14.94	16.52	17.43	19.07	19.75	21.10	0.659	0.15	14.19	15.43	16.10	17.89	18.97	21.06	23.22	24.41	26.45	27.26	28.81
17.5	0.043	0.151	10.52	11.26	11.68	12.81	13.54	14.99	16.60	17.53	19.20	19.89	21.25	0.692	0.148	14.49	15.77	16.45	18.27	19.37	21.48	23.66	24.86	26.91	27.72	29.27
18	0.068	0.154	10.45	11.21	11.63	12.79	13.53	15.01	16.64	17.59	19.28	19.98	21.37	0.724	0.148	14.78	16.09	16.79	18.65	19.78	21.93	24.14	25.35	27.43	28.24	29.80

Supplementary Table 41. Smoothed percentiles for right arm bone mineral content for female and male 5 to 18 years.

DXA right arm bone mineral content (g)

Age (years)	Female LMS parameters and percentile													Male LMS parameters and percentile												
	L	S	1th	3th	5th	15th	25th	50th (M)	75th	85th	95th	97th	99th	L	S	1th	3th	5th	15th	25th	50th (M)	75th	85th	95th	97th	99th
5	0.483	0.16	19.7	21.4	22.4	24.9	26.5	29.7	32.9	34.8	38	39.3	41.8	-0.43	0.131	23.4	24.7	25.4	27.3	28.6	31.2	34.2	35.9	39.1	40.5	43.3
5.5	0.463	0.15	22	23.8	24.8	27.4	29	32.2	35.6	37.4	40.7	42	44.5	-0.368	0.135	23.8	25.1	25.9	27.9	29.3	32	35.1	37	40.4	41.8	44.8
6	0.445	0.147	24	25.8	26.9	29.7	31.4	34.7	38.3	40.3	43.7	45.1	47.8	-0.311	0.139	25.6	27.1	28	30.3	31.8	34.9	38.4	40.5	44.3	45.9	49.1
6.5	0.428	0.151	25.5	27.6	28.7	31.7	33.6	37.3	41.2	43.4	47.2	48.7	51.7	-0.258	0.143	27.9	29.6	30.6	33.2	34.9	38.4	42.4	44.7	49	50.8	54.5
7	0.412	0.159	26.9	29.2	30.4	33.8	35.9	40.1	44.5	47	51.4	53.1	56.5	-0.209	0.148	29.9	31.8	32.9	35.8	37.8	41.7	46.1	48.7	53.4	55.5	59.5
7.5	0.398	0.168	28.2	30.7	32.1	36	38.4	43.1	48.1	51	56	58	62	-0.164	0.152	31.7	33.8	35	38.3	40.4	44.8	49.6	52.5	57.8	60	64.5
8	0.384	0.177	29.5	32.3	33.9	38.2	40.9	46.2	51.9	55.1	60.9	63.2	67.7	-0.122	0.157	33.8	36.1	37.4	41.1	43.4	48.3	53.7	56.9	62.8	65.2	70.1
8.5	0.372	0.184	31.1	34.2	36	40.7	43.7	49.6	56	59.7	66.1	68.7	73.8	-0.082	0.162	36	38.6	40	44.1	46.8	52.1	58.2	61.7	68.3	71	76.5
9	0.36	0.19	33.2	36.6	38.5	43.8	47.1	53.7	60.8	64.9	72.1	75.1	80.8	-0.045	0.167	38.1	41	42.7	47.2	50.1	56	62.7	66.7	73.8	76.9	82.9
9.5	0.348	0.194	35.7	39.5	41.6	47.4	51.1	58.5	66.5	71	79.2	82.5	89	-0.009	0.171	40.2	43.4	45.2	50.1	53.3	59.8	67.1	71.4	79.3	82.6	89.1
10	0.337	0.198	38.7	42.9	45.3	51.7	55.8	63.9	72.9	78	87.2	90.9	98.2	0.025	0.175	42.4	45.9	47.8	53.2	56.7	63.8	71.7	76.4	84.9	88.5	95.6
10.5	0.327	0.201	42.2	46.8	49.4	56.5	61	70.1	80	85.7	95.9	100.1	108.3	0.057	0.178	44.8	48.6	50.7	56.6	60.4	68.1	76.8	81.8	91.1	94.9	102.6
11	0.317	0.201	46.3	51.3	54.1	61.9	66.8	76.8	87.7	94	105.2	109.8	118.8	0.087	0.18	47.6	51.8	54.1	60.5	64.6	73.1	82.5	87.9	97.9	102.1	110.3
11.5	0.308	0.198	51.1	56.5	59.6	67.9	73.3	84	95.7	102.5	114.5	119.5	129.2	0.116	0.182	50.9	55.4	58	65	69.5	78.7	88.9	94.9	105.7	110.1	119
12	0.299	0.192	56.5	62.2	65.5	74.3	80	91.3	103.7	110.8	123.6	128.8	139	0.144	0.183	54.8	59.8	62.6	70.3	75.2	85.2	96.3	102.8	114.5	119.3	128.9
12.5	0.29	0.185	62.2	68.2	71.6	80.8	86.7	98.4	111.2	118.6	131.7	137.1	147.6	0.171	0.183	59.6	65.1	68.1	76.6	82	92.9	105	112	124.7	129.9	140.2
13	0.282	0.176	67.8	74	77.5	86.9	92.9	104.9	117.9	125.3	138.6	144	154.6	0.197	0.183	65.4	71.4	74.8	84	89.9	101.9	115	122.7	136.4	142	153.2
13.5	0.274	0.168	73.1	79.4	82.9	92.4	98.5	110.4	123.4	130.9	144.1	149.4	160	0.221	0.181	72.1	78.7	82.4	92.5	99	112	126.3	134.6	149.4	155.4	167.4
14	0.266	0.159	77.9	84.2	87.7	97.2	103.2	115.1	127.9	135.2	148.2	153.5	163.8	0.245	0.178	79.4	86.6	90.7	101.7	108.8	122.9	138.3	147.2	163	169.5	182.3
14.5	0.259	0.152	82	88.3	91.7	101.1	107.1	118.8	131.4	138.6	151.3	156.5	166.5	0.268	0.175	86.7	94.5	98.8	110.7	118.3	133.3	149.7	159.1	175.9	182.7	196.1
15	0.252	0.146	85.4	91.7	95.1	104.4	110.2	121.7	134.1	141.2	153.6	158.6	168.5	0.291	0.171	93.3	101.6	106.2	118.8	126.7	142.6	159.7	169.5	186.9	194	207.9
15.5	0.245	0.141	88.2	94.4	97.8	107	112.7	124.1	136.2	143.1	155.3	160.3	169.9	0.312	0.168	99.3	108	112.8	125.9	134.1	150.5	168.2	178.3	196.2	203.4	217.6
16	0.238	0.137	90.5	96.6	99.9	109	114.7	125.9	137.9	144.7	156.7	161.5	171	0.333	0.164	104.5	113.5	118.5	132	140.5	157.3	175.4	185.7	203.8	211.1	225.4
16.5	0.232	0.134	92.2	98.2	101.6	110.6	116.2	127.3	139.2	145.9	157.7	162.5	171.9	0.353	0.161	109.3	118.6	123.7	137.5	146.2	163.3	181.7	192	210.3	217.7	232.1
17	0.226	0.131	93.4	99.4	102.8	111.7	117.3	128.3	140	146.7	158.4	163.2	172.4	0.373	0.158	113.9	123.4	128.7	142.8	151.6	169	187.6	198.1	216.5	223.9	238.3
17.5	0.219	0.13	94.3	100.3	103.5	112.4	118	128.9	140.5	147.1	158.8	163.5	172.7	0.392	0.155	118.5	128.2	133.6	147.9	156.9	174.6	193.3	203.9	222.4	229.9	244.4
18	0.213	0.129	94.8	100.8	104	112.8	118.4	129.2	140.8	147.3	158.9	163.5	172.6	0.41	0.152	122.7	132.7	138.1	152.7	161.9	179.7	198.6	209.3	227.9	235.4	249.9

Supplementary Table 42. Smoothed percentiles for right leg bone mineral content for female and male 5 to 18 years.

DXA right leg bone mineral content (g)

Age (years)	Female LMS parameters and percentile													Male LMS parameters and percentile												
	L	S	1th	3th	5th	15th	25th	50th (M)	75th	85th	95th	97th	99th	L	S	1th	3th	5th	15th	25th	50th (M)	75th	85th	95th	97th	99th
5	0.249	0.16	55.3	59.8	62.2	69.0	73.2	81.7	90.8	96.0	105.3	109.1	116.5	0.331	0.149	57.3	61.7	64.1	70.7	74.8	82.8	91.4	96.3	104.9	108.3	115.0
5.5	0.253	0.164	60.6	65.7	68.5	76.1	81.0	90.6	101.1	107.0	117.7	122.0	130.5	0.045	0.147	62.9	67.2	69.6	76.2	80.4	88.8	98.1	103.4	113.1	117.0	124.8
6	0.256	0.17	65.6	71.3	74.5	83.1	88.6	99.5	111.4	118.1	130.3	135.2	144.9	-0.236	0.147	70.8	75.2	77.7	84.6	89.1	98.2	108.6	114.7	125.9	130.6	140.1
6.5	0.259	0.176	70.5	76.9	80.5	90.2	96.4	108.8	122.3	130.0	143.8	149.4	160.5	-0.49	0.147	79.6	84.2	86.9	94.2	99.1	109.1	120.8	127.9	141.2	146.9	158.7
7	0.262	0.183	75.9	83.1	87.1	98.1	105.1	119.1	134.5	143.3	159.1	165.6	178.3	-0.687	0.149	88.2	93.2	96.0	103.9	109.2	120.4	133.6	141.7	157.4	164.3	178.8
7.5	0.265	0.189	81.8	89.8	94.3	106.7	114.6	130.4	147.8	157.9	175.8	183.2	197.6	-0.825	0.153	96.7	102.1	105.1	113.8	119.6	132.0	147.0	156.4	174.9	183.2	200.9
8	0.268	0.193	88.0	97.0	102.0	115.7	124.5	142.2	161.7	172.9	193.0	201.2	217.5	-0.919	0.157	105.5	111.3	114.6	124.2	130.7	144.6	161.7	172.5	194.3	204.2	225.9
8.5	0.27	0.197	95.0	104.9	110.4	125.5	135.3	154.8	176.3	188.8	211.0	220.2	238.2	-0.948	0.163	114.4	120.8	124.5	135.1	142.3	158.0	177.4	189.8	215.1	226.7	252.4
9	0.272	0.198	103.3	114.1	120.1	136.8	147.5	169.0	192.7	206.4	230.9	241.0	260.9	-0.891	0.168	123.3	130.5	134.6	146.6	154.7	172.3	194.1	208.1	236.4	249.5	278.2
9.5	0.274	0.199	113.0	124.9	131.5	149.8	161.5	185.2	211.2	226.2	253.2	264.2	286.0	-0.748	0.172	132.5	140.8	145.5	159.1	168.3	188.0	212.3	227.7	258.4	272.3	302.5
10	0.276	0.198	124.1	137.1	144.4	164.4	177.3	203.1	231.6	248.0	277.5	289.6	313.4	-0.53	0.176	142.1	151.8	157.3	172.9	183.4	205.8	232.5	249.2	281.5	295.8	326.1
10.5	0.278	0.197	136.4	150.6	158.6	180.4	194.4	222.6	253.6	271.4	303.4	316.6	342.4	-0.254	0.179	151.9	163.4	169.9	188.2	200.3	225.5	254.9	272.6	306.1	320.4	349.9
11	0.28	0.194	149.9	165.3	173.9	197.6	212.7	243.1	276.5	295.7	330.2	344.3	372.1	0.059	0.181	161.5	175.3	183.1	204.8	218.8	247.2	279.2	297.9	332.0	346.2	374.6
11.5	0.282	0.191	164.3	180.9	190.1	215.5	231.7	264.1	299.8	320.3	356.9	371.9	401.5	0.378	0.182	170.5	187.4	196.8	222.4	238.6	270.6	305.1	324.7	359.4	373.5	401.0
12	0.284	0.187	178.9	196.5	206.3	233.2	250.3	284.6	322.2	343.7	382.2	398.0	428.9	0.669	0.183	179.1	199.8	211.1	241.2	259.7	295.3	332.5	353.0	388.4	402.4	429.4
12.5	0.285	0.182	193.1	211.5	221.8	249.9	267.7	303.5	342.5	364.8	404.7	420.9	452.9	0.9	0.182	188.4	213.3	226.6	261.3	282.1	321.4	361.2	382.7	419.2	433.4	460.4
13	0.287	0.178	205.9	225.0	235.6	264.6	283.0	319.7	359.6	382.5	423.2	439.8	472.4	1.048	0.181	200.0	228.8	244.0	282.9	305.9	348.6	391.0	413.7	451.7	466.4	494.1
13.5	0.288	0.173	217.1	236.7	247.5	277.0	295.7	332.9	373.4	396.5	437.5	454.2	487.0	1.106	0.179	215.4	247.0	263.5	305.8	330.6	376.4	421.5	445.5	485.6	501.1	530.1
14	0.29	0.168	226.7	246.4	257.3	287.1	305.9	343.4	383.9	407.0	448.0	464.7	497.4	1.081	0.177	234.3	267.3	284.6	329.0	355.1	403.4	451.3	476.8	519.5	535.9	566.9
14.5	0.291	0.164	234.6	254.4	265.4	295.2	314.0	351.4	391.7	414.7	455.4	472.0	504.3	0.994	0.174	254.7	287.8	305.4	350.6	377.6	427.8	478.1	505.0	550.4	568.0	601.3
15	0.292	0.16	241.2	261.0	272.0	301.7	320.4	357.5	397.5	420.3	460.5	476.9	508.8	0.864	0.172	274.5	306.8	324.1	369.3	396.5	448.0	500.3	528.6	576.8	595.6	631.3
15.5	0.294	0.156	246.7	266.4	277.3	306.9	325.5	362.2	401.8	424.2	464.0	480.1	511.5	0.707	0.169	292.5	323.6	340.4	384.9	412.1	464.2	518.0	547.7	598.5	618.6	657.0
16	0.295	0.153	251.2	270.8	281.7	311.0	329.4	365.8	404.9	427.0	466.2	482.0	512.9	0.535	0.167	308.2	337.9	354.1	397.5	424.4	476.8	532.0	562.7	616.3	637.6	678.9
16.5	0.296	0.15	254.9	274.4	285.2	314.2	332.5	368.4	407.0	428.8	467.4	483.0	513.4	0.355	0.165	321.9	350.1	365.7	407.9	434.4	486.8	543.1	574.9	631.1	653.9	698.2
17	0.297	0.147	258.0	277.3	287.9	316.7	334.7	370.2	408.3	429.8	467.8	483.1	513.0	0.173	0.164	333.8	360.8	375.8	416.8	442.9	495.2	552.5	585.5	644.5	668.7	716.4
17.5	0.299	0.145	260.3	279.4	290.0	318.4	336.2	371.2	408.7	429.9	467.3	482.3	511.7	-0.009	0.163	344.5	370.3	384.7	424.7	450.4	502.6	561.0	595.0	657.1	682.8	734.3
18	0.3	0.142	262.1	281.0	291.4	319.5	337.1	371.6	408.6	429.4	466.1	480.9	509.7	-0.188	0.162	353.9	378.7	392.7	431.6	456.9	509.0	568.4	603.6	668.8	696.3	752.0

Supplementary Table 43. Smoothed percentiles for truncal bone mineral content for female and male 5 to 18 years.

DXA Truncal lean mass (g)

Age	Female LMS parameters and percentile													Male LMS parameters and percentile												
	L	S	1th	3th	5th	15th	25th	50th (M)	75th	85th	95th	97th	99th	L	S	1th	3th	5th	15th	25th	50th (M)	75th	85th	95th	97th	99th
5	-0.653	0.124	4172	4374	4487	4804	5010	5435	5924	6217	6766	7001	7484	0.361	0.138	4195	4493	4656	5094	5366	5899	6464	6781	7335	7558	7990
5.5	-0.566	0.107	4587	4785	4895	5199	5394	5790	6233	6493	6970	7170	7573	0.362	0.122	4654	4941	5098	5516	5775	6279	6809	7105	7621	7827	8227
6	-0.487	0.102	4860	5064	5177	5487	5684	6081	6521	6777	7242	7435	7821	0.319	0.111	5140	5422	5576	5987	6240	6733	7250	7539	8042	8243	8632
6.5	-0.414	0.108	5004	5228	5353	5694	5912	6351	6837	7120	7634	7848	8275	0.189	0.105	5563	5841	5993	6400	6652	7143	7664	7955	8466	8671	9069
7	-0.346	0.120	5108	5365	5508	5901	6153	6662	7230	7562	8168	8421	8927	-0.006	0.103	5899	6175	6326	6733	6988	7489	8027	8331	8868	9086	9512
7.5	-0.283	0.133	5228	5522	5686	6140	6432	7026	7691	8082	8798	9098	9700	-0.220	0.105	6146	6425	6580	6999	7263	7790	8365	8694	9284	9526	10004
8	-0.225	0.142	5386	5716	5901	6412	6742	7414	8170	8615	9431	9773	10461	-0.417	0.111	6377	6670	6832	7279	7565	8141	8781	9155	9835	10118	10685
8.5	-0.169	0.148	5600	5963	6166	6728	7090	7829	8659	9147	10042	10416	11168	-0.566	0.119	6612	6926	7102	7590	7906	8552	9285	9719	10525	10866	11558
9	-0.117	0.153	5856	6252	6475	7090	7486	8293	9198	9730	10701	11107	11920	-0.637	0.128	6842	7183	7376	7912	8262	8987	9821	10322	11266	11670	12502
9.5	-0.068	0.159	6124	6563	6809	7490	7929	8822	9824	10411	11484	11931	12827	-0.621	0.135	7077	7449	7659	8247	8633	9435	10364	10926	11989	12447	13393
10	-0.021	0.167	6399	6890	7165	7927	8419	9421	10545	11204	12407	12909	13913	-0.539	0.142	7325	7735	7966	8614	9039	9922	10946	11563	12730	13231	14266
10.5	0.024	0.175	6700	7249	7557	8411	8964	10089	11352	12092	13444	14006	15133	-0.413	0.148	7593	8050	8309	9032	9504	10483	11611	12288	13558	14101	15212
11	0.066	0.180	7075	7681	8022	8967	9577	10819	12210	13024	14507	15124	16355	-0.260	0.156	7882	8403	8697	9516	10050	11149	12404	13151	14540	15128	16322
11.5	0.106	0.179	7580	8235	8602	9616	10269	11593	13066	13925	15481	16125	17407	-0.094	0.164	8203	8805	9143	10083	10691	11936	13340	14168	15689	16326	17607
12	0.145	0.171	8203	8891	9275	10332	11008	12369	13871	14740	16305	16949	18224	0.072	0.171	8581	9280	9671	10751	11445	12852	14418	15329	16983	17668	19031
12.5	0.183	0.161	8892	9597	9989	11060	11741	13101	14587	15439	16963	17586	18815	0.224	0.175	9074	9878	10325	11551	12332	13896	15610	16595	18358	19080	20503
13	0.218	0.150	9571	10281	10673	11739	12412	13745	15188	16010	17469	18062	19225	0.352	0.174	9732	10636	11136	12494	13351	15047	16878	17917	19755	20500	21955
13.5	0.253	0.139	10185	10891	11280	12329	12987	14282	15672	16458	17845	18406	19501	0.444	0.168	10574	11559	12100	13560	14473	16263	18169	19241	21120	21874	23340
14	0.286	0.131	10709	11407	11789	12818	13459	14715	16052	16804	18125	18657	19691	0.497	0.160	11564	12600	13166	14685	15629	17465	19405	20488	22375	23129	24588
14.5	0.318	0.124	11128	11818	12196	13206	13834	15057	16352	17077	18345	18853	19840	0.512	0.151	12597	13649	14222	15755	16702	18541	20473	21548	23416	24161	25599
15	0.349	0.120	11439	12127	12502	13504	14125	15329	16599	17308	18543	19037	19993	0.491	0.141	13568	14605	15171	16678	17610	19415	21309	22363	24192	24922	26329
15.5	0.379	0.118	11651	12344	12721	13727	14348	15551	16815	17519	18743	19231	20175	0.442	0.133	14421	15428	15976	17440	18344	20099	21943	22970	24756	25469	26846
16	0.408	0.117	11781	12487	12871	13892	14522	15741	17017	17727	18959	19450	20397	0.370	0.126	15129	16099	16628	18042	18919	20622	22419	23422	25172	25872	27227
16.5	0.436	0.119	11845	12571	12965	14013	14659	15906	17210	17934	19190	19690	20653	0.281	0.121	15701	16636	17148	18518	19370	21032	22794	23782	25512	26207	27556
17	0.463	0.121	11862	12610	13016	14095	14758	16039	17377	18119	19404	19915	20899	0.180	0.117	16160	17069	17567	18906	19742	21380	23128	24113	25847	26546	27909
17.5	0.489	0.123	11850	12619	13035	14142	14822	16134	17502	18260	19570	20091	21093	0.070	0.116	16529	17419	17908	19229	20057	21689	23445	24440	26202	26917	28316
18	0.515	0.125	11828	12612	13038	14165	14858	16191	17579	18347	19674	20201	21213	-0.046	0.115	16816	17692	18175	19485	20311	21950	23727	24743	26553	27292	28746

Supplementary Table 44. Smoothed percentiles for total body fat free mass (kg) for female and male 5 to 18 years.

DXA Fat free mass (kg)

Age (years)	Female LMS parameters and percentile													Male LMS parameters and percentile												
	L	S	1th	3th	5th	15th	25th	50th (M)	75th	85th	95th	97th	99th	L	S	1th	3th	5th	15th	25th	50th (M)	75th	85th	95th	97th	99th
5	-1.326	0.133	9.49	9.91	10.15	10.83	11.30	12.30	13.53	14.32	15.92	16.66	18.31	-0.904	0.125	10.17	10.63	10.90	11.64	12.13	13.16	14.36	15.10	16.52	17.14	18.44
5.5	-1.211	0.12	10.43	10.86	11.11	11.81	12.27	13.26	14.43	15.16	16.60	17.24	18.61	-0.795	0.113	11.13	11.62	11.89	12.64	13.13	14.14	15.30	16.00	17.31	17.86	19.01
6	-1.107	0.113	11.14	11.59	11.85	12.57	13.04	14.03	15.20	15.91	17.29	17.89	19.16	-0.696	0.107	12.07	12.57	12.85	13.64	14.14	15.17	16.33	17.02	18.29	18.83	19.92
6.5	-1.01	0.115	11.60	12.09	12.36	13.13	13.64	14.70	15.93	16.68	18.12	18.75	20.06	-0.604	0.103	12.87	13.40	13.70	14.52	15.04	16.10	17.29	17.99	19.27	19.80	20.88
7	-0.921	0.122	11.98	12.52	12.82	13.68	14.25	15.43	16.81	17.65	19.27	19.98	21.46	-0.519	0.103	13.53	14.10	14.41	15.28	15.83	16.94	18.18	18.90	20.22	20.76	21.86
7.5	-0.837	0.133	12.36	12.97	13.31	14.28	14.92	16.27	17.86	18.84	20.72	21.54	23.27	-0.441	0.106	14.04	14.66	15.00	15.94	16.54	17.74	19.08	19.85	21.27	21.85	23.03
8	-0.76	0.143	12.77	13.45	13.84	14.93	15.65	17.18	18.99	20.11	22.26	23.21	25.21	-0.367	0.113	14.52	15.20	15.59	16.63	17.30	18.65	20.15	21.02	22.60	23.26	24.58
8.5	-0.687	0.151	13.26	14.02	14.44	15.66	16.46	18.16	20.18	21.42	23.83	24.89	27.12	-0.297	0.122	14.98	15.76	16.19	17.38	18.15	19.69	21.40	22.41	24.23	24.99	26.51
9	-0.618	0.157	13.84	14.67	15.14	16.47	17.36	19.23	21.46	22.83	25.47	26.64	29.08	-0.232	0.131	15.46	16.33	16.82	18.16	19.02	20.76	22.71	23.84	25.91	26.77	28.50
9.5	-0.552	0.163	14.47	15.38	15.90	17.37	18.35	20.42	22.87	24.38	27.29	28.57	31.25	-0.17	0.137	16.03	16.99	17.53	19.00	19.95	21.87	24.01	25.26	27.54	28.48	30.38
10	-0.491	0.17	15.15	16.15	16.73	18.35	19.43	21.72	24.43	26.10	29.32	30.72	33.67	-0.112	0.142	16.70	17.76	18.34	19.96	21.00	23.09	25.42	26.78	29.24	30.27	32.31
10.5	-0.432	0.175	15.89	17.00	17.63	19.43	20.62	23.14	26.13	27.96	31.49	33.03	36.24	-0.056	0.147	17.42	18.58	19.22	21.00	22.14	24.45	27.00	28.49	31.19	32.31	34.53
11	-0.376	0.178	16.79	18.00	18.69	20.64	21.94	24.67	27.90	29.88	33.65	35.30	38.71	-0.003	0.154	18.14	19.43	20.15	22.14	23.41	25.98	28.83	30.48	33.49	34.73	37.20
11.5	-0.322	0.176	17.89	19.19	19.93	22.02	23.40	26.29	29.68	31.74	35.64	37.33	40.81	0.048	0.163	18.90	20.35	21.16	23.39	24.82	27.71	30.91	32.78	36.16	37.55	40.33
12	-0.271	0.169	19.20	20.57	21.35	23.53	24.96	27.93	31.37	33.44	37.32	38.98	42.38	0.097	0.17	19.79	21.41	22.32	24.82	26.42	29.65	33.24	35.33	39.10	40.66	43.75
12.5	-0.222	0.16	20.62	22.03	22.83	25.05	26.50	29.48	32.88	34.91	38.66	40.25	43.48	0.144	0.174	20.95	22.74	23.74	26.50	28.27	31.83	35.77	38.05	42.16	43.86	47.22
13	-0.174	0.15	22.00	23.44	24.24	26.47	27.91	30.86	34.17	36.12	39.69	41.19	44.21	0.188	0.173	22.50	24.45	25.53	28.51	30.40	34.22	38.41	40.83	45.17	46.95	50.46
13.5	-0.129	0.14	23.27	24.71	25.51	27.73	29.15	32.02	35.21	37.08	40.46	41.87	44.67	0.232	0.168	24.42	26.49	27.64	30.78	32.77	36.75	41.09	43.58	48.02	49.83	53.39
14	-0.085	0.132	24.36	25.79	26.59	28.78	30.17	32.97	36.05	37.83	41.03	42.36	44.98	0.273	0.159	26.59	28.75	29.94	33.18	35.23	39.28	43.67	46.16	50.59	52.38	55.90
14.5	-0.043	0.125	25.23	26.67	27.46	29.63	31.00	33.73	36.71	38.43	41.50	42.76	45.25	0.313	0.15	28.77	30.97	32.19	35.47	37.53	41.58	45.93	48.38	52.71	54.46	57.86
15	-0.002	0.121	25.90	27.34	28.13	30.28	31.64	34.34	37.27	38.94	41.92	43.14	45.54	0.352	0.14	30.76	32.99	34.20	37.48	39.52	43.51	47.75	50.14	54.31	55.99	59.25
15.5	0.038	0.119	26.37	27.82	28.62	30.79	32.15	34.84	37.75	39.41	42.35	43.55	45.90	0.389	0.132	32.47	34.68	35.89	39.12	41.13	45.03	49.14	51.45	55.46	57.07	60.18
16	0.076	0.119	26.67	28.15	28.96	31.16	32.54	35.26	38.19	39.85	42.80	44.00	46.34	0.426	0.125	33.83	36.03	37.22	40.41	42.38	46.19	50.19	52.42	56.29	57.83	60.81
16.5	0.113	0.12	26.82	28.33	29.16	31.41	32.81	35.58	38.56	40.25	43.23	44.44	46.80	0.461	0.121	34.89	37.09	38.28	41.44	43.39	47.15	51.08	53.25	57.02	58.52	61.41
17	0.149	0.121	26.84	28.40	29.25	31.55	32.99	35.83	38.87	40.59	43.62	44.85	47.25	0.495	0.118	35.71	37.93	39.13	42.31	44.26	48.02	51.94	54.10	57.84	59.32	62.17
17.5	0.184	0.124	26.77	28.37	29.24	31.61	33.08	35.99	39.10	40.86	43.95	45.21	47.65	0.528	0.118	36.35	38.61	39.84	43.08	45.07	48.89	52.85	55.03	58.80	60.30	63.16
18	0.218	0.126	26.62	28.26	29.16	31.59	33.10	36.08	39.26	41.05	44.22	45.49	47.98	0.56	0.119	36.84	39.19	40.46	43.81	45.86	49.80	53.87	56.12	59.98	61.51	64.44

Supplementary figure 3. Body composition 3-C model by DXA at different Tanner puberal stages by sex.

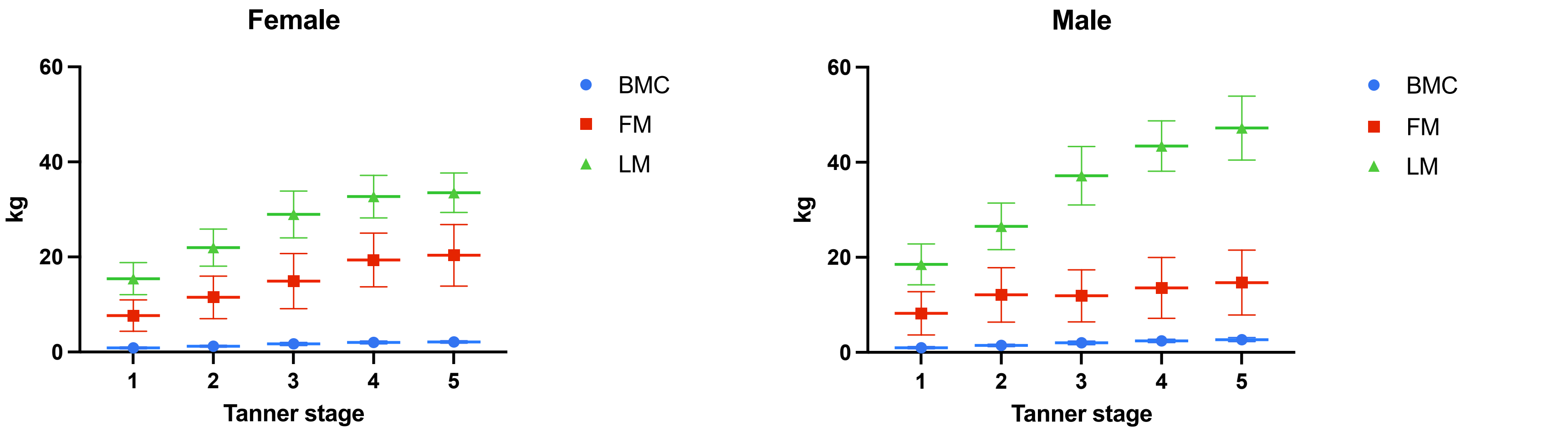

Supplementary figure 4. Comparison between the behavior of reference values for FM% by DXA published in other studies.

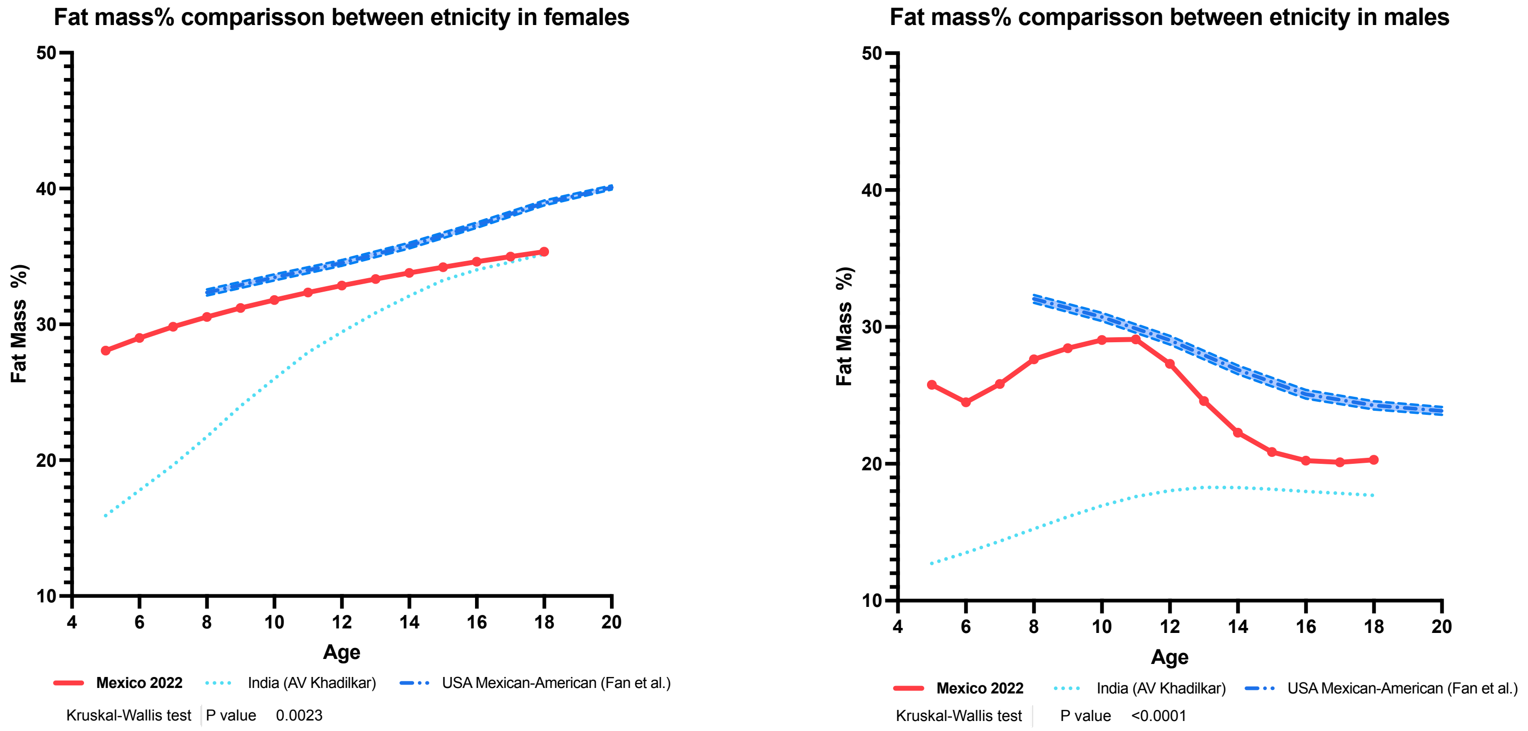

Supplement: Supplementary file 1 — Supplementary Material [file 41430_2023_1352_MOESM1_ESM.pdf]
